# Raising the HOMO level of the [*closo*-B_10_H_10_]^2−^ anion: apical alkyl derivatives for modern materials

**DOI:** 10.1039/d5sc08516k

**Published:** 2025-11-24

**Authors:** Rafał Jakubowski, Kehinde Ogunmola, Oleksandr Hietsoi, Andrienne C. Friedli, Kevin H. Shaughnessy, Piotr Kaszyński

**Affiliations:** a Centre of Molecular and Macromolecular Studies, Polish Academy of Sciences 90-363 Łódź Poland piotr.kaszynski@cbmm.lodz.pl; b Department of Chemistry, Middle Tennessee State University Murfreesboro TN 37132 USA; c Department of Chemistry and Biochemistry, The University of Alabama Tuscaloosa AL 35487 USA; d Faculty of Chemistry, University of Łódź 91-403 Łódź Poland

## Abstract

The HOMO energy of the [*closo*-B_10_H_10_]^2−^ anion is increased by placing one or two alkyl groups at the apical positions of the cluster. To accomplish this, a Pd(0) catalysed C–B cross coupling reaction of RZnCl and mono- or diiodo cluster derivatives was developed. DFT results show that these iodo derivatives are the most challenging substrates among typical *closo*-borane iodides characterised by the highest B–I bond strength and associated the largest σ* MO energy. The most effective catalyst for the B–I coupling reaction was BrettPhosPd-G3, which gave the alkylated products in high yields. The alkyl effect on the {B_10_} cluster geometry was demonstrated with single crystal XRD and correlation analyses. The electronic properties of the mono- and dialkyl products were probed with electrochemistry and UV-vis spectroscopy of charge transfer ion pairs. The electrochemical results correlate with Hammett substituent parameters and DFT derived *E*_HOMO_, while the CT band energy is proportional to the difference between the FMO energies. Material properties of such newly available alkyl derivatives of the [*closo*-B_10_H_10_]^2−^ anion were investigated with an Fe(ii) complex and ionic LCs displaying CT behaviour.

## Abstract 2

The HOMO energy of the [*closo*-B_10_H_10_]^2−^ anion is increased by placing one or two alkyl groups at the apical positions of the cluster.

## Graphical abstract

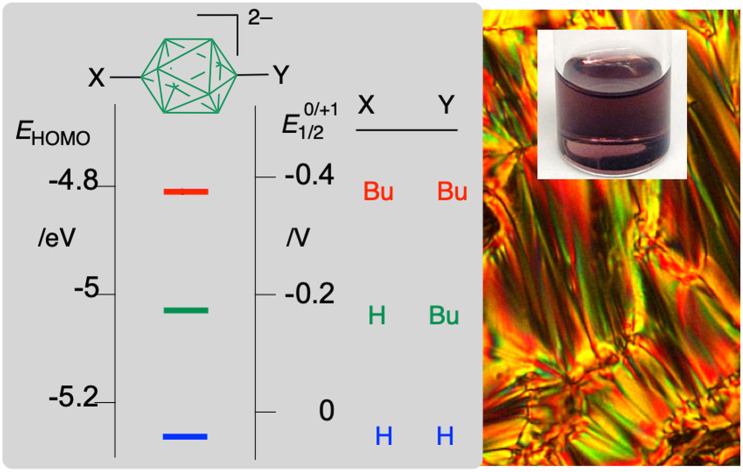

## Introduction

**Fig. 1:**
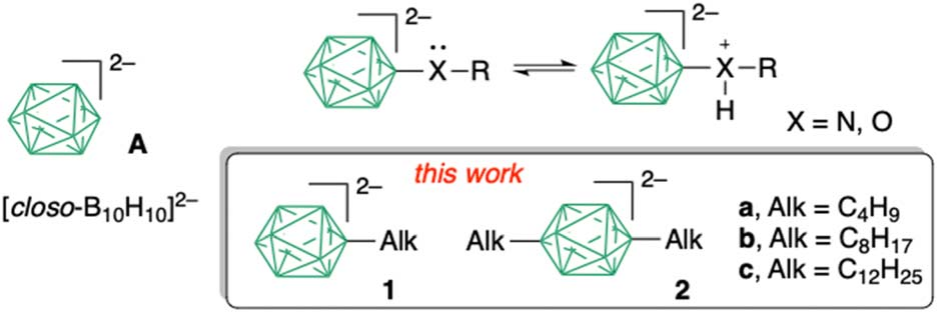
Left: [*closo*-B_10_H_10_]^2–^ (A). Right: protonation of RX derivative of [*closo*-B_10_H_10_]^2−^. In the box: apical alkyl derivatives of anion A. In the cluster each unsubstituted vertex corresponds to a B–H fragment.

*closo*-Boron clusters^1^ are unique inorganic compounds that are of increasing interest, mainly as structural elements of advanced materials and pharmacophores.^2^ Their geometry is suitable for self-organizing materials,^3^ while the σ-aromatic electronic structure^4^ can be exploited in tuning photophysical and electrochemical properties of organic materials.^5^ Of the common *closo*-boron clusters, the *closo*-decaborate dianion^6^ [*closo*-B_10_H_10_]^2−^ (A, Fig. 1) is notable for its square bipyramidal geometry with *D*_4d_ symmetry and exceptionally high HOMO energy level. These properties make the cluster particularly attractive for the design of photonic materials with low energy bandgaps and CT behaviour.^7^

The HOMO energy of the [*closo*-B_10_H_10_]^2−^ cluster responds to the electronic properties of the substituents,^7^ with electron-donating groups increasing its level. Most substituents investigated to date are electron withdrawing, while those with electron donating properties are needed for achieving the full range of tuneable electronic properties of anion A. Unfortunately, the negative charge on the [*closo*-B_10_H_10_]^2−^ cluster increases the basicity of typical π-electron-donating atoms, such as X = O and N, which leads to their ready protonation and consequent lowering of the HOMO energy level. One substituent that has a moderately negative *σ*_p_ parameter^8^ (−0.16), yet lacks basic sites, is the alkyl group. The only example of an apical alkyl derivative of anion A is the dimethyl derivative [*closo*-B_10_H_8_-1,10-Me_2_]^2−^ obtained by LiAlH_4_ reduction of the dicarbonyl derivative [*closo*-B_10_H_8_-1,10-(CO)_2_].^9^ Further exploration of this class of compounds as electron donors, liquid crystals and photonic materials with tuneable properties requires a more general method for controlled and regioselective substitution of alkyl groups onto the [*closo*-B_10_H_10_]^2−^ anion. In this report we demonstrate selective preparation of mono and di-alkyl derivatives substituted at the apical positions of [*closo*-B_10_H_10_]^2−^ anion (Fig. 1).

A potential general route to apical mono- or dialkyl-substituted [*closo*-B_10_H_10_]^2−^ anions, 1 and 2 in Fig. 1, would be palladium-catalysed alkylation and dialkylation of the previously reported iodides^10^ [*closo*-B_10_H_9_-1-I]^2−^ (3) and [*closo*-B_10_H_8_-1,10-I_2_]^2−^ (4), respectively. Palladium-catalysed B–C bond formation (alkylation, arylation and alkynylation reactions) is fairly well established for iodo derivatives of *closo*-C_2_B_10_H_12_,^11^ and to a lesser extent for anions [*closo*-1-CB_11_H_12_]^−^,^12^ [*closo*-B_12_H_12_]^2−^,^13^ and [*closo*-1-CB_9_H_10_]^−^,^14^ but all these reactions are limited in scope compared to the analogous C–C cross-coupling. For derivatives of [*closo*-B_10_H_10_]^2−^ (A) there are only three reported examples of Kumada coupling of aryl Grignard reagents with iodides 3 and 4.^7*b*,10^ No palladium-catalysed alkylation or alkynylation reactions of anion A to give apically substituted *closo*-borate derivatives have been reported to date.^9*b*^

Herein we report an efficient Pd(0)-catalysed alkyl-B cross-coupling in [*closo*-B_10_H_10_]^2−^ anion, and preparation of derivatives 1 and 2 containing three representative alkyl groups, butyl (a), octyl (b) and dodecyl (c, Fig. 1). The effect of the alkyl group on the structural and electronic properties of anion A is investigated with XRD, spectroscopic, electrochemical and DFT methods. Monoalkyl derivatives are used to prepare an Fe(ii) complex and to test for the formation of ionic liquid crystals with intermolecular charge transfer properties.

## Results and discussion

### Analysis of electrophile electronic properties

Oxidative addition of a carbon–halogen bond to a Pd(0) centre is the first step in palladium-catalyzed cross-coupling reactions.^15^ Such additions of haloarenes to Pd(0) are thermodynamically favourable leading to isolable complexes. The rate of the process decreases with increasing bond strength (rate: I > Br > Cl)^16^ and correlates with the energy of the C–X σ* molecular orbital. Oxidative addition of B–X bonds of *closo*-boranes is significantly more challenging than for aryl halide C–X bonds due to the high strength of the B–X bond and the increased energy level of the associated orbital. For example, reaction of 9-iodo-*m*-carborane with Pd(PPh_3_)_4_ in toluene at 70 °C for 3 h resulted in no observable reaction when analysed by ^31^P NMR spectroscopy.^17^ When the reaction of 9-iodo-*m*-carborane with Pd(PPh_3_)_4_ was performed in the presence of [Bu_4_N]Br for 12 h at 30 °C and then 2 hours at 55 °C, a 1 : 5 ratio of 9-bromo-*m*-carborane and 9-iodo-*m*-carborane was obtained. This result suggests that oxidative addition of the B–I bond of carborane occurs as part of a reversible endothermic step, in which the concentration of the oxidative addition product is below the ^31^P NMR detection limit. The subsequent halide exchange at the Pd centre followed by reductive elimination gives the B–Br product. Presumably, successful palladium-catalysed functionalization of iodo-*closo*-boranes proceeds through a similar reversible endothermic oxidative addition of the B–X bond to Pd(0) and trapping of the transient Pd(ii)-boryl intermediate by the coupling partner.

**Fig. 2:**
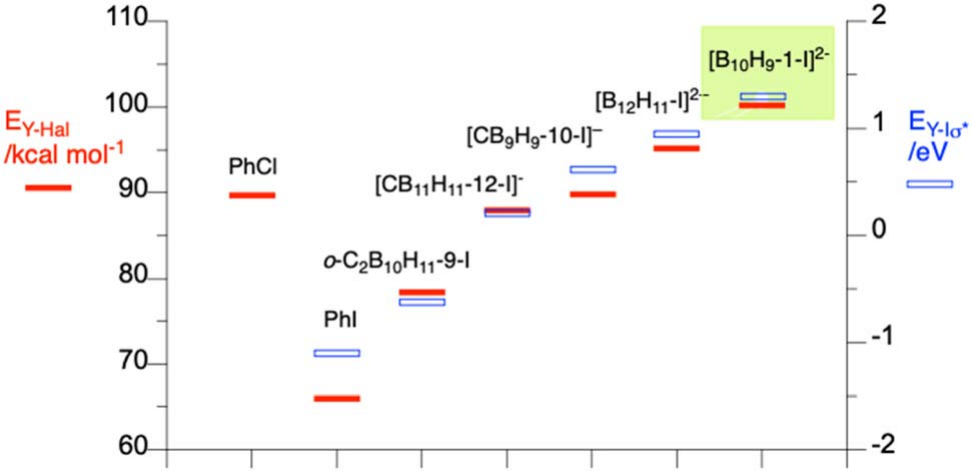
DFT (B3LYP/Def2TZVP) calculated homolytic BDE (*E*_Y-Hal_, red bars) and B/C–I σ* orbital energies (blue hollow bars) in the gas phase. For details see SI.

DFT calculations at the B3LYP/Def2TZVP level of theory demonstrate that the B–I bond of all the *closo*-borate clusters are significantly stronger (by 12–34 kcal mol^−1^) than the C–I bond of iodobenzene, and the bond strength increases with increasing negative charge on the cluster (red bars in Fig. 2). The B–I bond is also stronger for the 10-vertex clusters compared to the 12-vertex analogue (*e.g*. [*closo*-B_12_H_11_-1-I]^2−^*vs.* [*closo*-B_10_H_9_-1-I]^2−^). It should be noted that the B–I bond in the [*closo*-B_10_H_9_-1-I]^2−^ cluster is about 9 kcal mol^−1^ stronger than the C–Cl bond of chlorobenzene, which is already a demanding substrate for Pd-catalysed cross-coupling. A similar trend is found for the energy associated with the B–I σ* orbital (blue bars in Fig. 2), which would be the key orbital involved in oxidative addition. Results show that the B–I σ* energy for [*closo*-B_10_H_9_-1-I]^2−^ anion (3) is the highest in the series: it is nearly 2.4 eV higher than that of the C–I σ* in iodobenzene or 1.9 eV higher than the B–I σ* in iodocarborane. Thus, the calculated highest B–I bond strength and σ* energy in the series for 3 suggest that oxidative addition of the dianionic iodide 3 to Pd(0) centre will be particularly difficult. It was hypothesized that successful cross-coupling of derivatives of [*closo*-B_10_H_10_]^2−^ (A) would require a palladium catalyst that is highly activated towards oxidative addition and conditions to promote fast transmetalation of the transient palladium-boryl intermediate present in low concentrations.

### Synthetic method development

**Scheme 1:**
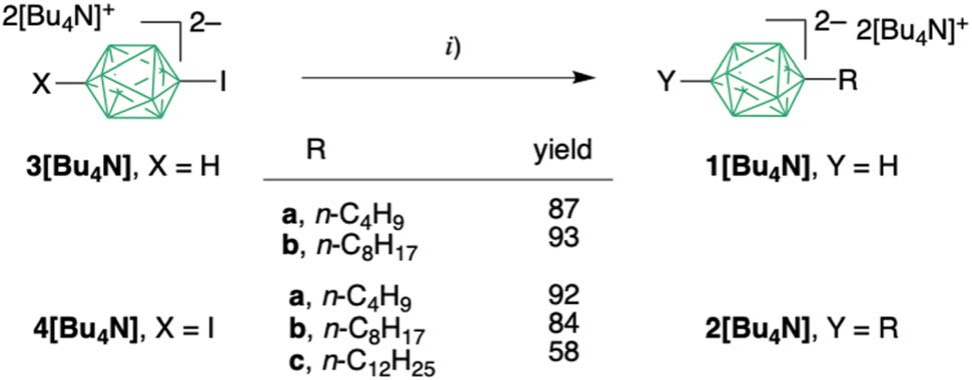
Synthesis of mono and dialkyl derivatives 1 and 2.^*a*^ Reagents and conditions: (i) RMgCl/ZnCl_2_/LiCl (10 eq per B–I), BrettPhosPd-G3 (3.75 mol% per B–I), THF, reflux, 72 h.

Initial optimization of the C–B cross-coupling reaction conditions was performed for butylzinc chloride, generated *in situ* from butylmagnesium chloride and ZnCl_2_·LiCl, and [*closo*-B_10_H_8_-1,10-I_2_][Bu_4_N]_2_ (4[Bu_4_N], Scheme 1). Palladium catalyst systems known to promote coupling with less reactive aryl halides^18^ were tried first.^19^ PEPPSI-IPr, G3-XPhos, and G3-DPPF gave no conversion to the alkylated product and produced only the hydrodeiodinated parent A in addition to unreacted iodide 4[Bu_4_N]. Byproduct A is likely formed by β-hydrogen elimination of palladium alkyl intermediates to give a boryl-palladium hydride that undergoes reductive elimination to give the B–H bond. In contrast, catalysts derived from BrettPhos or SPhos and the G3 palladacycle precatalyst gave full consumption of 4 and low yields of the desired product 2a (∼25%) accompanied by a complex mixture of boron byproducts, including 1a and the parent A. The reactions were followed by ^11^B NMR, where the characteristic peaks of the apical boron atoms were shifted from about −5 ppm in the starting iodide to about 5 ppm in the alkylated products.^19^ Full details and summary of all experiments are provided in the SI.

It was reasoned that the poor selectivity for the alkylated product with BrettPhos and SPhos may be partially due to inefficient interception of the palladium-boryl intermediate by the organozinc reagent, which allows unproductive processes to occur with the palladium-boryl species. Increasing the ratio of the organozinc reagent to the boron cluster improved the selectivity for the dialkylated product with the BrettPhos-G3 catalyst system. With a 20 : 1 mole ratio of butylzinc chloride to 4[Bu_4_N], 92% conversion to [*closo*-B_10_H_8_-1,10-Bu_2_]^2−^2a was achieved after 72 hours with only traces of [*closo*-B_10_H_10_]^2−^ and [*closo*-B_10_H_9_-1-Bu]^2−^ as byproducts based on ^11^B NMR spectroscopy.

Under these conditions, BrettPhos was the optimal ligand. Lower yields of 2a were obtained using SPhos, CPhos, or JoyPhos with the G3 palladacycle. Rigorous exclusion of oxygen in all stages of preparation of 2a by setting up the reactions under nitrogen in the glove box was critical to the success of the reaction. The subsequent work-up and purification of the products was conducted in air on the benchtop. Reactions set up under Ar on the benchtop using standard anaerobic techniques gave complex mixtures with no desired product.

Using these optimized conditions, [*closo*-B_10_H_8_-1,10-Bu_2_][Bu_4_N]_2_ (2a[Bu_4_N]) was isolated in 92% yield with >90% purity (^11^B NMR) after aqueous workup in air and purification using a short alumina column (Scheme 1). The product was isolated as an oil that solidified upon standing. The dioctyl 2b[Bu_4_N] and didodecyl 2c[Bu_4_N] analogues were isolated in 84% and 58% yield, respectively, and purity >90%. Mono-alkylated products, monobutyl 1a[Bu_4_N] and monooctyl 1b[Bu_4_N] were obtained in high yields starting from [*closo*-B_10_H_9_-1-I][Bu_4_N]_2_ (3[Bu_4_N]) and using a 10 : 1 mole ratio of the organozinc reagent to the iodide.

Attempts were made to convert the oily or waxy [Bu_4_N]^+^ salts of 1 and 2 to easier-to-handle and more oxidatively stable crystalline derivatives, such as those containing the [Et_4_N]^+^ or Cs^+^. Thus, extraction of 2a[H_3_O] to ether from 10% HCl solution of 2a[Bu_4_N] followed by treatment with [Et_4_N]Cl gave the desired 2a[Et_4_N] along with approximately 50% decomposition products, as demonstrated^19^ by complex ^11^B NMR spectra (see the SI). Attempted cation exchange using ion exchange resins gave incomplete ion exchange and partial decomposition. It was determined that all dialkylated and, to lesser extent, mono-alkylated products are acid-sensitive, possibly due to their elevated HOMO level leading to facile protonation of the cage and presumably ring opening to *nido* derivatives.^20^ Therefore, the oily [Bu_4_N]^+^ salts were used for further transformations. For crystallographic characterisation, two ion pairs of mono- and di-butyl derivatives 1a[Ph_4_P] and 2a[Ph_4_P] were obtained by metathesis of the [Bu_4_N]^+^ salts with [Ph_4_P]Cl in CH_2_Cl_2_/H_2_O system.

### Molecular and crystal structures

**Fig. 3:**
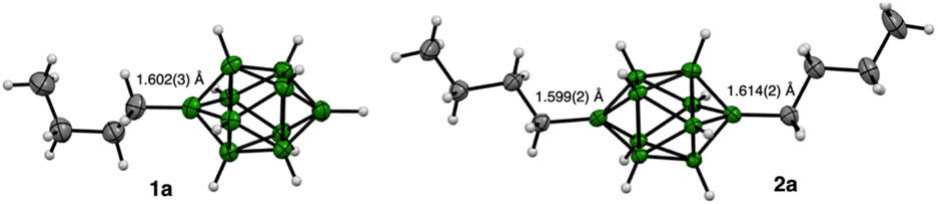
Thermal ellipsoid diagram for anions 1a and 2a (ellipsoids set at 50% probability) with the [Ph_4_P]^+^ cations and solvent molecule omitted for clarity. Angle between the alkyl group mean planes in 2a is 28.8°. For details see the SI.

Single crystals of ion pairs 1a[Ph_4_P]·MeOH (monoclinic, *P*2_1_/*n*) and 2a[Ph_4_P] (triclinic, *P*-1) suitable for XRD analysis were obtained by cooling followed by slow evaporation of hot MeOH solutions. Structural analysis revealed that in 1a the Bu group adopts a staggered orientation relative to the cage, with the B(1)–C bond length of 1.602(3) Å (Fig. 3). The C(2)–C(3) bond in the butyl chain exists in a gauche conformation. In the dibutyl derivative 2a, the alkyl groups in the all-*trans* conformation are rotated about 8° from the ideal eclipsed orientation, with B–C distances of 1.599(2) and 1.614(2) Å, respectively. These distances are well reproduced by DFT calculations (1.608 Å) and are similar to that found in a B(10)–alkyl derivative of the [*closo*-1-CB_9_H_10_]^−^ anion (1.593(3) Å).^14*a*^

Further structural analysis indicates that the Bu groups in 1a and 2a exert a significant effect on the {B_10_} cage geometry. Thus, the height of the tetragonal pyramid (1.116 Å) and the resulting B(1)⋯B(10) separation in 2a (3.757(2) Å), are the largest reported to date for apical derivatives of A, and consistent with the strong electron donating character of the apical substituent. In a series of nine [*closo*-B_10_H_8_-1,10-X_2_]^2−^ derivatives,^7*b*,21^ including 2a, both distances correlate well (*r*^2^ > 0.95) with Hammett^8^ substituent parameters *σ*_p_ giving the slope of 0.054(4) and 0.12(1) Å/*σ*_p_, respectively.^19^

### Electrochemical analysis

**Fig. 4:**
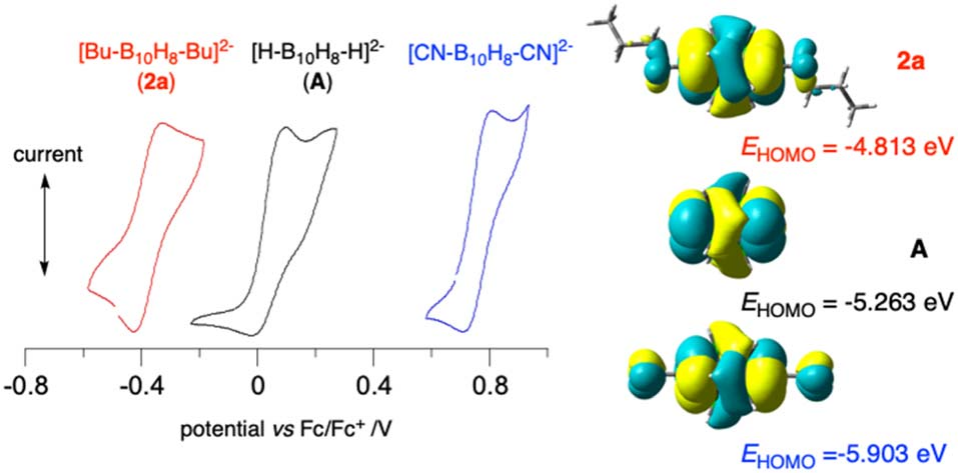
Left: cyclic voltammograms for dibutyl 2a[Bu_4_N] (red), parent A (black) and [*closo*-B_10_H_8_-1,10-(CN)_2_]^2−^ (blue). Conditions: 0.5 mM analyte in MeCN [Bu_4_N][PF_6_] (100 mM) at *ca* 22 °C, scans of 100 mV s^−1^ in the anodic direction, glassy carbon working electrode (*φ* = 1 mm), Pt counter electrode and an Ag/AgCl pseudo-reference electrode. Right: B3LYP/Def2TZVP-derived HOMO contours and energies in MeCN dielectric medium (MO Isovalue = 0.02). Full data in SI.

The effect of the alkyl groups on the level of the HOMO of the {B_10_} cage was probed with electrochemical methods. Cyclic voltammetry demonstrated that the oxidation processes in 1a and 2a recorded in MeCN are quasireversible and significantly more cathodic than those of the parent dianion A by 0.19 V and 0.42 V (Fig. 4 and ESI). This shift corresponds to lifting the HOMO of dianion A by 0.23 and 0.45 eV, respectively, or lowering the adiabatic ionisation energy by 0.30 and 0.52 eV, respectively, according to DFT calculations in MeCN dielectric medium.^19^ The measured low oxidation potential *E*_1/2_^0/+1^ of −0.376 V *vs.* the Fc/Fc^+^ couple for 2a[Bu_4_N] is consistent with the observed limited stability of this and other dialkyl derivatives 2 during storage, presumably due to air oxidation and sensitivity to acids.

**Fig. 5:**
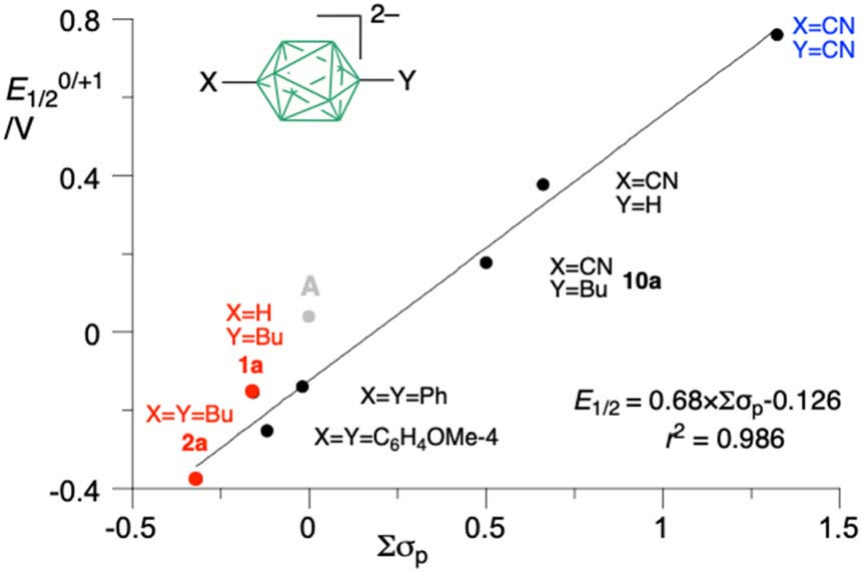
Correlation of oxidation potentials *E*_1/2_^0/+1^ (*vs.* Fc/Fc^+^) with the sum of Hammett parameters Σ*σ*_p_. The datapoint for A (grey dot) is not used for the correlation. Numerical values are shown in the SI.

Analysis of a series of eight [*closo*-B_10_H_8_-1-X-10-Y]^2−^ derivatives containing apical Bu, CN and Ar substituents demonstrates a good correlation of *E*_1/2_^0/+1^ with the sum of Hammett^8^ substituent parameters, Σ*σ*_p_ (Fig. 5), and excellent correlation with the calculated HOMO level and adiabatic ionisation energy for each anion.^19^ Assuming validity of this correlation in Fig. 5, the *E*_1/2_^0/+1^ potential of the hypothetical 1,10-dimethoxy derivative [*closo*-B_10_H_8_-1,10-(OMe)_2_]^2−^ can be predicted to be −0.49(2) V *vs.* the Fc/Fc^+^ couple, while its B(1)⋯B(10) separation would be 3.76(1) Å.

### Charge transfer complexes

**Fig. 6:**
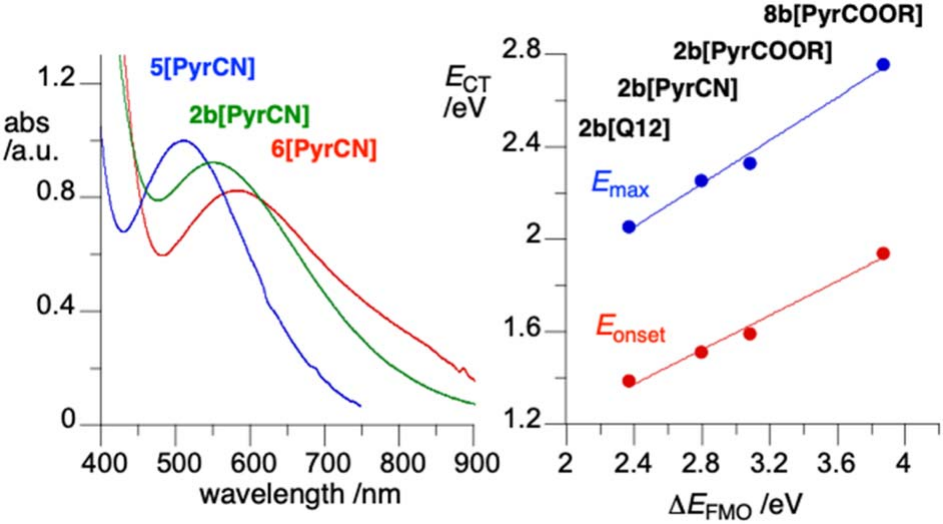
Left: UV-vis spectra for ion pairs with *N*-decyl-4-cyanopyridine (PyrCN). Right: correlation of CT energy *vs.* difference of the FMO energies (Δ*E*_FMO_ = *E*_LUMO_ − *E*_HOMO_) calculated at the CAM-B3LYP/Def2TZVP level in CH_2_Cl_2_. See Chart 1 for structures.

**Chart 1:**
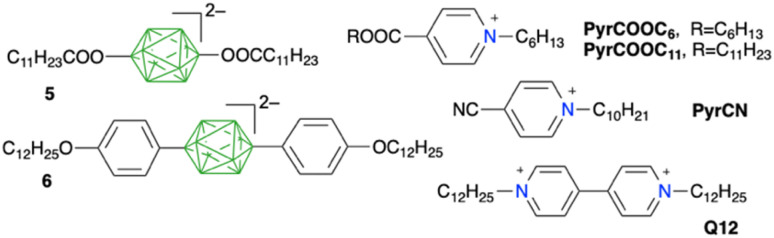


The high level of the HOMO in alkyl derivatives 2 is also evident from energies of intermolecular charge transfer (CT) bands observed in concentrated solutions of ion pairs of 2b with pyridinium cations (Fig. 6, Chart 1). Thus, as the electron-withdrawing power of the C(4) substituent on the *N*-alkylpyridinium cation increases, the LUMO energy decreases, and so does the difference, Δ*E*_FMO_, between the energies of the LUMO of pyridinium and the HOMO localized on the anion. Consequently, the energy of the intermolecular CT band decreases from 2.33 eV (532 nm) for 2b[PyrCOOC_11_] to 2.05 eV (604 nm) for 2b[Q12] (see structures in Chart 1). These experimental CT energies correlate well with the calculated Δ*E*_FMO_ values (Fig. 6), as observed previously for related ion pairs, *e.g.*5[PyrCN] and 6[PyrCN] in Chart 1.^7*b*^ As expected, the CT band of 2b[PyrCN] is positioned between those of the two analogous ion pairs, the dicarboxylate 5[PyrCN] and dialkoxyphenyl 6[PyrCN] (Fig. 6, left).

### Molecular materials

**Scheme 2:**
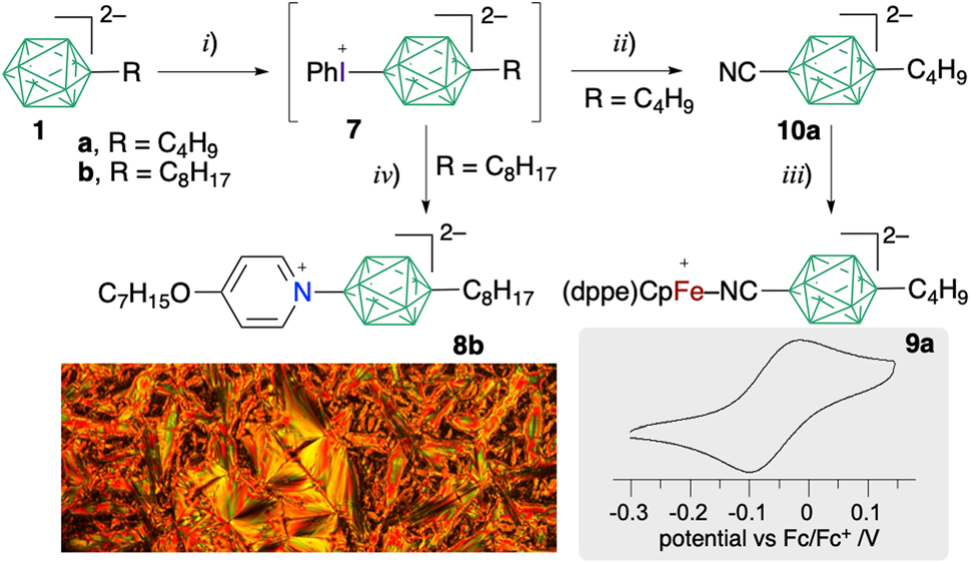
Synthesis of pyridinium derivative 8b and Fe(ii) complex 9a.^*a*^ Reagents and conditions: (i) PhI(OAc)_2_, MeCN, 0 °C 1 h, then rt 16 h, 45% for 7a and 54% yield for 7b; (ii) [Bu_4_N]CN, MeCN, 55–60 °C, 2 d, 30% yield; (iii) [(η^5^-Cp)(dppe)FeCl, CH_2_Cl_2_, reflux 16 h, 66% yield; (iv) 4-C_7_H_15_OC_5_H_4_N neat, 50 °C, 2 d, 58% yield. Below left: polarised optical microscopy of a Sm texture at 125 °C of 8b[PyrCOOC_11_]. Right: cyclic voltammogram for 9a[Bu_4_N].

Polarised light optical microscopy demonstrated that none of the investigated ion pairs of anion 2b exhibited liquid crystalline behaviour, which is in contrast to behaviour of similar ion pairs 5[PyrCN] and 6[PyrCN].^7*b*^ To induce mesogenic behaviour, the monooctyl derivative 1b was substituted with a 4-heptyloxypyridinium group using the previously developed aryliodonium method for selective activation of B–H bonds in *closo*-borates.^12*e*,22^ Thus, the monooctyl derivative 1b was reacted with PhI(OAc)_2_ in MeCN (Scheme 2) giving crude product 7b with ∼80% purity (based on ^11^B NMR). The crude product was purified by rapid chromatography using a SiO_2_ column impregnated with [Bu_4_N][HSO_4_] to give pure, moderately stable phenyliodonium derivative 7b in about 50% yield as a yellow-brownish oil.

The octyl derivative 7b[Bu_4_N] was reacted with neat 4-heptyloxypyridine to give essentially colourless product 8b[Bu_4_N] in 58% yield (Scheme 2). Exchange of the [Bu_4_N]^+^ cation for pyridinium PyrCOOC_6_ or PyrCOOC_11_ (Chart 1) gave a reddish-orange waxy solid ion pair 8b[PyrCOOC_n_], which upon heating displayed textures characteristic for a smectic A phase (Scheme 2). Both ion pairs exhibited limited thermal stabilities and slowly decomposed above 150 °C, with estimated clearing temperatures below 200 °C for *n* = 11 and about 250 °C for *n* = 6.

Lastly, the significant electron donating ability of the apical alkyl group was demonstrated in Fe(ii) complex 9a[Bu_4_N], which was obtained from [*closo*-B_10_H_8_-10-Bu-1-CN]^2−^ (10a[Bu_4_N]) prepared from phenyliodonium derivative 7a[Bu_4_N] shown in Scheme 2. Electrochemical analysis, conducted in CH_2_Cl_2_ for comparison purposes, demonstrated a quasireversible oxidation process with *E*_1/2_^0/+1^ = −0.054 V (*vs.* Fc/Fc^+^, Scheme 2), which is significantly cathodically shifted relative to the symmetric derivative [*closo*-B_10_H_8_-1,10-(CN{Fe})_2_] (0.056 V *vs.* Fc/Fc^+^)^23^ and the analogous Fe(ii) complex with pyrazinium derivative [*closo*-B_10_H_8_-1-CN{Fe}-10-Pyrazine] (0.080 V *vs.* Fc/Fc^+^).^24^ The observed trend in *E*_1/2_^0/+1^ values correlates well with the HOMO energy, as calculated with a DFT method (B3LYP/Def2SVP) for model compounds.^19^

## Conclusions

We have demonstrated an efficient method for preparation of mono- and dialkyl derivatives of [*closo*-B_10_H_10_]^2−^ anion (A) using Pd(0) catalysed B–C cross-coupling with the apical mono- and diiodo precursors, respectively. This method fills a significant void in functional derivatives of the [*closo*-B_10_H_10_]^2−^ anion and provides access to this previously unavailable class of derivatives for further studies. Results demonstrate that each apical alkyl group raises the HOMO level of the parent dianion A by about 0.22 eV. Since the effect is cumulative, dialkyl derivatives 2 approach the limit of oxidative stability in air and toward acids. The high level of the HOMO in 1 and 2 was confirmed by electrochemical and UV-vis methods. The former method demonstrated a cathodic shift of the *E*_1/2_^ox^ potential by about 0.2 V per each apical alkyl group, while the electronic absorption spectroscopy revealed low energy intermolecular CT bands in pyridinium ion pairs. Substituent effects on the geometry and electronic and redox behaviour of the {B_10_} cage correlate well with Hammett parameters *σ*_p_ and DFT results, which permits the design and fine tuning of properties. The applications of the monoalkyl derivatives 1 as an Fe(ii) complex and ionic liquid crystals demonstrates materials with controlled redox properties, self-organisation and CT behaviour.

## Author contributions

The manuscript was written through contributions of all authors, and all authors have given approval to the final version of the manuscript.

## Conflicts of interest

The authors declare no competing financial interest.

## Supplementary Material

SC-017-D5SC08516K-s001

SC-017-D5SC08516K-s002

## Data Availability

The data supporting this article have been included as part of the supporting information (SI). CCDC 2362917 1a[Ph_4_P]·MeOH and 2362918 2a[Ph_4_P] contain the supplementary crystallographic data for this paper.^25*a*,*b*^ Supplementary information (SI): data for this article, including synthetic procedures and characterisation details (NMR, UV-vis, E-chem), crystallographic data, and computational results. See DOI: https://doi.org/10.1039/d5sc08516k.

## References

[cit1] (a) E. L. Muetterties , ed., Boron Hydride Chemistry, Academic Press, New York, 1975

[cit2] (a) N. S. Hosmane and R. Eagling, in Handbook of Boron Science: With Applications in Organometallics, Catalysis, Materials and Medicine, 2019

[cit3] Kaszynski P. , in Handbook of Boron Science, eds. N. S. Hosmane and R. Eagling, World Scientific, London, 2018, vol. 3, pp. 57–114

[cit4] Poater J., Viñas C., Bennour I., Escayola S., Solà M., Teixidor F. (2020). J. Am. Chem. Soc..

[cit5] Núñez R., Tarrés M., Ferrer-Ugalde A., Fabrizi de Biani F., Teixidor F. (2016). Chem. Rev..

[cit6] Sivaev I. B., Prikaznov A. V., Naoufal D. (2010). Collect. Czech. Chem. Commun..

[cit7] Kapuściński S., Abdulmojeed M. B., Schafer T. E., Pietrzak A., Hietsoi O., Friedli A. C., Kaszyński P. (2021). Inorg. Chem. Front..

[cit8] Hansch C., Leo A., Taft R. W. (1991). Chem. Rev..

[cit9] Knoth W. H., Sauer J. C., Balthis J. H., Miller H. C., Muetterties E. L. (1967). J. Am. Chem. Soc..

[cit10] Rzeszotarska E., Novozhilova I., Kaszyński P. (2017). Inorg. Chem..

[cit11] Olid D., Núñez R., Viñas C., Teixidor F. (2013). Chem. Soc. Rev..

[cit12] Grüner B., Janoušek Z., King B. T., Woodford J. N., Wang C. H., Všetečka V., Michl J. (1999). J. Am. Chem. Soc..

[cit13] Peymann T., Knobler C. B., Hawthorne M. F. (1998). Inorg. Chem..

[cit14] Ringstrand B., Kaszynski P., Januszko A., Young, Jr. V. G. (2009). J. Mater. Chem..

[cit15] Biffis A., Centomo P., Del Zotto A., Zecca M. (2018). Chem. Rev..

[cit16] Fitton P., Rick E. A. (1971). J. Organomet. Chem..

[cit17] Marshall W. J., Young, Jr. R. J., Grushin V. V. (2001). Organometallics.

[cit18] Ingoglia B. T., Wagen C. C., Buchwald S. L. (2019). Tetrahedron.

[cit19] For details see the SI.

[cit20] Hawthorne M. F., Mavunkal I. J., Knobler C. B. (1992). J. Am. Chem. Soc..

[cit21] Jacob L., Rzeszotarska E., Pietrzak A., Young, Jr. V. G., Kaszyński P. (2020). Eur. J. Inorg. Chem..

[cit22] Kaszyński P. (2025). Aust. J. Chem..

[cit23] Guschlbauer J., Shaughnessy K. H., Pietrzak A., Chung M.-C., Sponsler M. B., Kaszyński P. (2021). Organometallics.

[cit24] Jakubowski R., Abdulmojeed M. B., Hietsoi O., Friedli A. C., Kaszynski P. (2024). Inorg. Chem..

[cit25] (a) CCDC 2362917, Experimental Crystal Structure Determination, 2025, 10.5517/ccdc.csd.cc2k9t4v

