# Supplementary material for: Raising the HOMO level of the [closo-B10H10]2− anion: apical alkyl derivatives for modern materials
Source: Chem Sci. 2025 Nov 24;17(2):1282–7. doi: 10.1039/d5sc08516k (PMC12642937; doi:10.1039/d5sc08516k)
Supplement: SC-017-D5SC08516K-s001 [file SC-017-D5SC08516K-s001.pdf]

Supplementary Information (SI) for Chemical Science.
This journal is © The Royal Society of Chemistry 2025

Electronic Supplementary Information
for

Raising the HOMO level of the [*closo*-$B_{10}H_{10}$]$^{2-}$ anion: Apical alkyl derivatives for modern materials

Rafał Jakubowski,[a,b] Kehinde Ogunmola,[c] Oleksandr Hietsoi,[a] Andrienne C. Friedli,*[a] Kevin Shaughnessy,*[c] and Piotr Kaszyński*[a,b,d]

[a] Department of Chemistry, Middle Tennessee State University, Murfreesboro, TN 37130, USA
[b] Centre of Molecular and Macromolecular Studies, Polish Academy of Sciences, 90-363 Łódź, Poland
[c] Department of Chemistry and Biochemistry, University of Alabama, Tuscaloosa, AL 35487, USA
[d] Faculty of Chemistry, University of Łódź, 91-403 Łódź, Poland

Table of Content:

1. Synthetic details ….S2
2. NMR spectra S17
3. IR spectrum of **9a[Bu$_4$N]** S44
4. XRD data collection and refinement details S45
   a) general comments S45
   b) structure solution and refinement S45
   c) data analysis S46
5. Electronic absorption spectra S50
6. Electrochemical data ….S55
7. Computational details S58
   a) general S58
   b) homolytic bond dissociation energy S59
   c) molecular geometry of anions and cations S59
   d) HOMO and ionization energies of anions S60
   e) FMO energies for model cations and anions S63
   f) models of the Fe(II) complexes S64
8. Archive for DFT results S66
9. References S81

1. Synthetic details

General. All alkylation reactions and the preparation of **10a[Bu_4N]** were assembled in a nitrogen-filled glovebox (MBraun), while all other reactions were carried out under Ar atmosphere on the benchtop. In both cases subsequent manipulations were conducted in air on the benchtop. Anhydrous $ZnCl_2$ and LiCl were obtained by drying overnight in high vacuum in an oil bath (140 °C) and immediately transferring to the glovebox. Tetrahydrofuran was taken from a Solvent Purification System prior to use in the coupling reactions. Literature procedures were used to obtain [*closo*-$B_{10}H_{10}$][Et_3NH]$_2$,[1] and iodides **3[Bu_4N]** and **4[Bu_4N]**.[2]

NMR spectra were obtained at 500 MHz (^{1}H), 126 MHz (^{13}C) and 160 MHz (^{11}B) in CD_3CN unless otherwise indicated. Chemical shifts were referenced to the solvent (^{1}H and ^{13}C: 1.94 ppm and 118.26 ppm for CD_3CN)[3] or an external sample of neat BF_3•Et_2O in CD_3CN (^{11}B, $\delta = 0.0$ ppm). ^{11}B NMR chemical shifts were obtained from {^{1}H} decoupled spectra. IR spectra were recorded for neat samples using an ATR attachment. HR mass spectrometry was conducted with the TOF-MS ES method, typically in the negative mode.

Optimization of reaction conditions for B-alkylation.

Negishi butylation of 4[Bu_4N]. In a nitrogen filled glovebox, $ZnCl_2$ (1.5 equiv. relative to *n*-BuMgCl) and LiCl (1.5 equiv. relative to *n*-BuMgCl) were added to an oven-dried 10 mL vial containing a magnetic stir bar. The vial was sealed with a septum cap. The sealed vial was removed from the glove box and dried under vacuum using a heat gun for 30 min. The dried solid was placed under nitrogen, and THF (1.5 mL) was added. The solution was stirred until a clear solution formed (ultrasonic irradiation can be used to obtain a clear solution). The resulting $ZnCl_2$•LiCl solution was cooled to 0 °C and a 2.0 M solution of *n*-butylmagnesium chloride in THF (8–20 mole eq. relative to **4[Bu_4N]**, see Table S1) was added dropwise. The resulting solution was stirred under nitrogen at room temperature for 3 h to yield *n*-butylzinc chloride.

A separate oven-dried 10 mL vial equipped with a magnetic stir bar was placed in the glove box, and charged with **4[Bu_4N]** (50 mg, 0.058 mmol) and the palladium catalyst (5–10 mol%, 1:1 Pd:ligand, see Table S1). After sealing with a septum cap, the vial was removed from the glove box and placed under positive nitrogen pressure. THF (1.5 mL) was added by a syringe and the solution was stirred for 15 min. The previously prepared *n*-butylzinc chloride solution was then

transferred into the **4[Bu_4N]**/catalyst solution by a syringe. The reaction mixture was placed in an oil bath preheated to 70 °C and stirred for 48–72 h. After completion, the reaction was cooled to 0 °C in an ice bath, opened to ambient atmosphere, and quenched with water. Solvent (THF) was removed under reduced pressure at 40 °C using a rotary evaporator. Saturated NH_4Cl solution (1 mL) was added to the remaining material. The aqueous suspension was extracted three times with CH_2Cl_2 and the extracts were dried (Na_2SO_4). After removal of the solvent under reduced pressure, the residue was dissolved in CD_3CN (*ca.* 1 mL) and analyzed by ^{11}B NMR spectroscopy. Examples of the ^{11}B NMR spectra are shown in Figures S1–S3.

Table S1. Optimization of the butylation of **4[Bu_4N].**

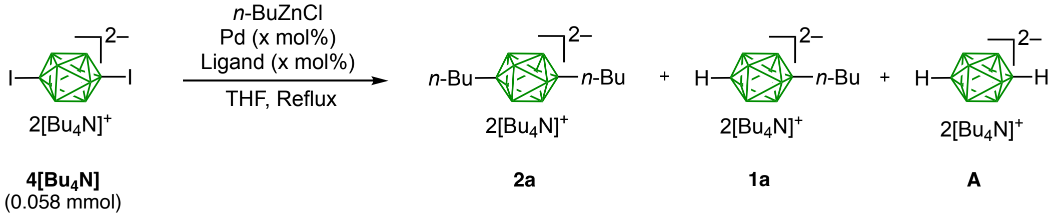

entry	Pd/L	Pd/L /mol%^{b}	RZnCl /mmol	mole ratio	time /h	**4[Bu_4N]** /% ^{c}	**2a** /% ^{c}	**1a** /% ^{c}	**A** /% ^{c}
1	G3/XPhos	7.5	0.46	8	48	64	0	0	36
2	PEPPSI-IPr	7.5	0.46	8	48	45	0	0	55
3	G3/DPPF	7.5	0.46	8	48	12	0	0	88
4	G3/SPhos	10	0.46	8	48	0	28	22	50
5	G3/BrettPhos	7.5	0.46	8	48	0	25	24	51
6	G3/BrettPhos	7.5	0.69	12	72	0	60	39	1
7	G3/BrettPhos	7.5	0.93	16	72	0	78	16	6
8	G3/BrettPhos	7.5	1.16	20	72	0	92	4	4
9	G3/CPhos	7.5	1.16	20	72	0	77	14	9
10	$Pd_2(dba)_3$/ JoyPhos	7.5	1.16	20	72	22	68	3	7
11	G3/SPhos	7.5	1.16	20	72	52	14	26	8

^{a} All reactions were performed with 0.058 mmol of **4[Bu_4N]**. ^{b} Palladium loading relative to **4[Bu_4N]**. Pd:L = 1:1. ^{c} Yields were determined from ^{11}B NMR spectra (160 MHz) of the crude reaction mixture dissolved in CD_3CN.

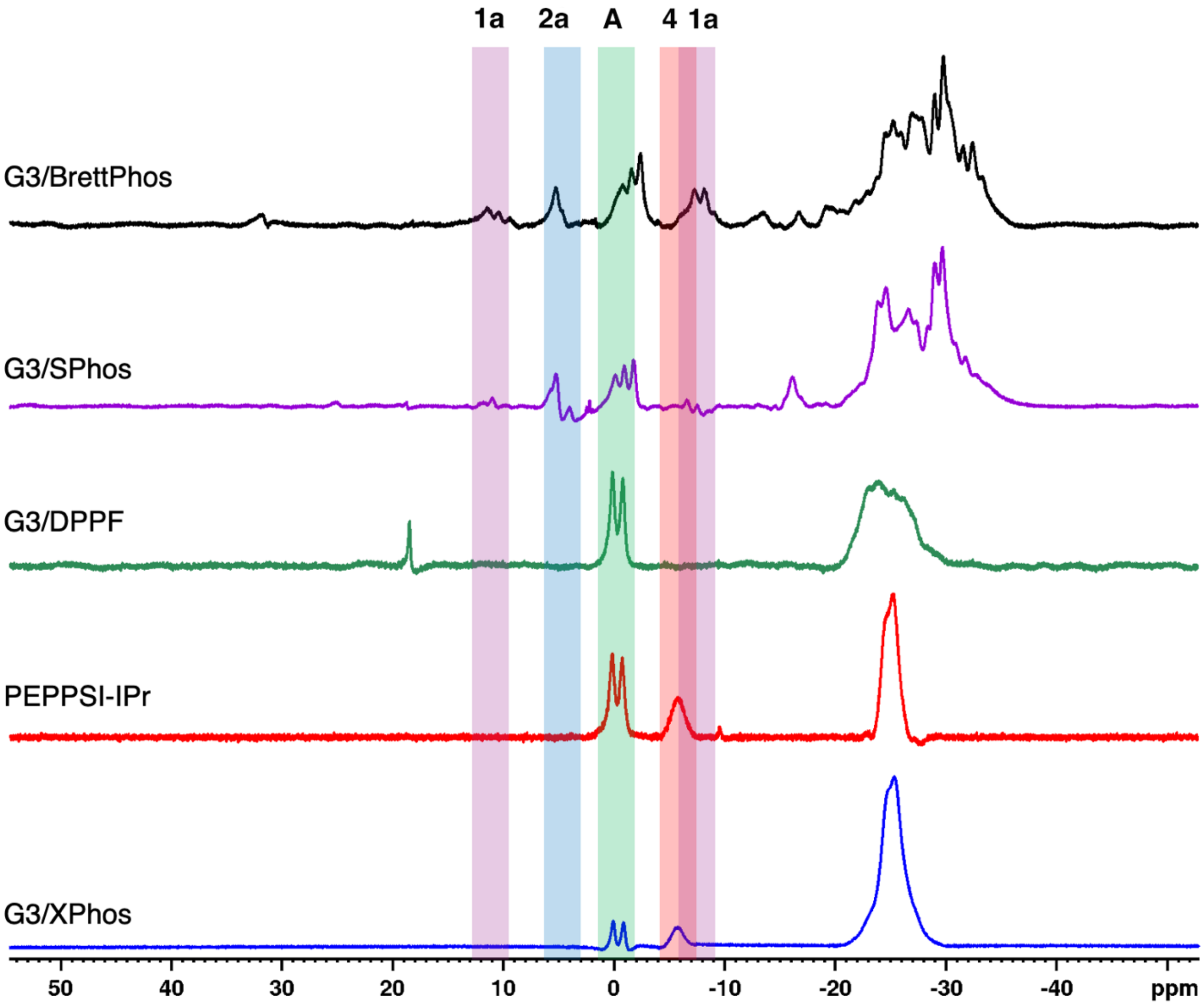

Figure S1. ^{11}B NMR spectra (160 MHz, CD_3CN) of crude reaction mixtures in the reaction of *n*-BuZnCl with **4[Bu$_4$N]** with different precatalysts. Conditions: *n*-BuZnCl:**4[Bu$_4$N]** 8:1, 7.5–10 mol% catalyst (1:1 Pd:ligand), THF, 70 °C, 72 h. Spectra correspond to Table S1, entries 1–5. Peaks corresponding to **2a**, **4**, and other by products are highlighted.

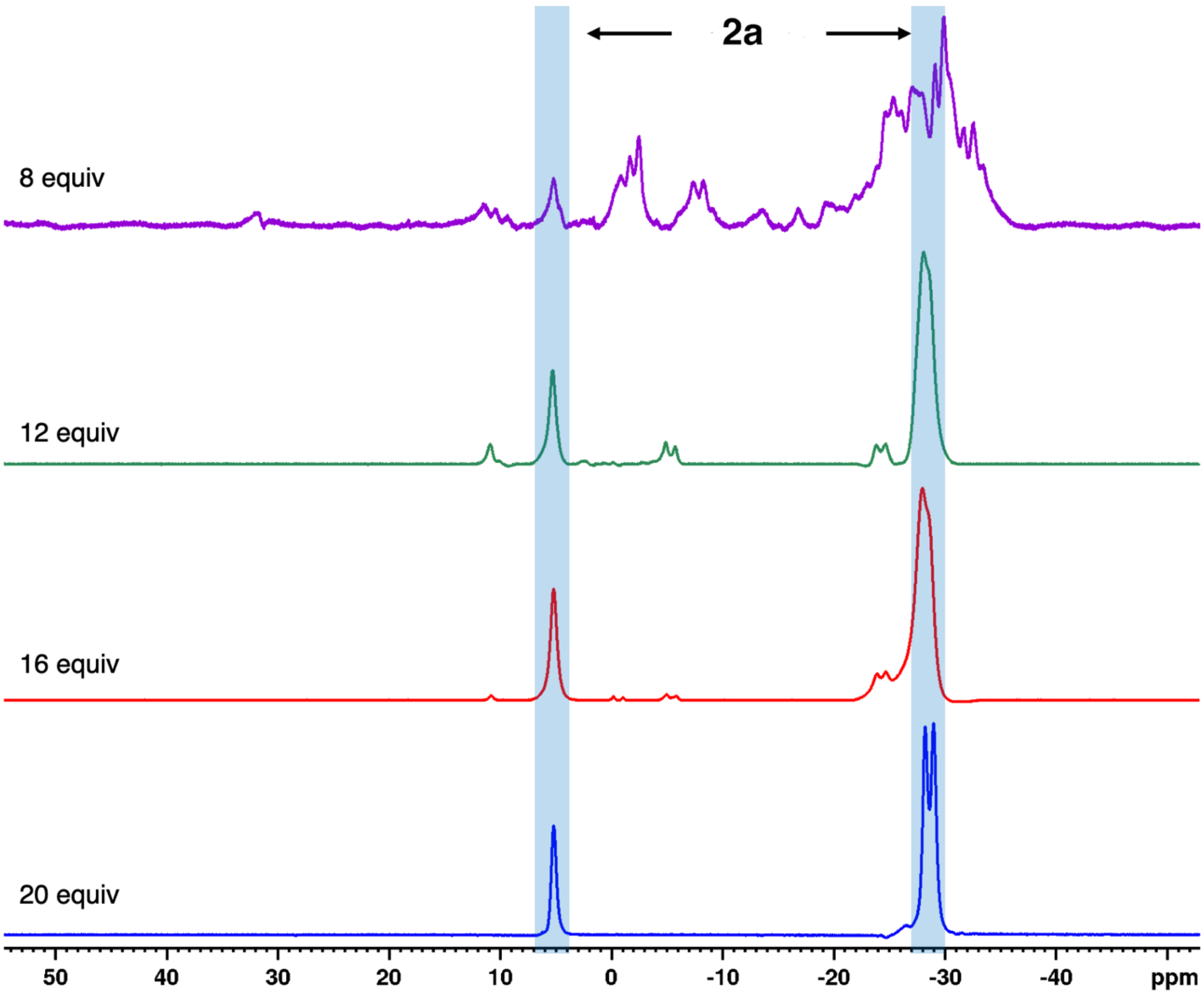

Figure S2. ^{11}B NMR spectra (160 MHz, CD_3CN) of crude reaction mixtures in the reaction of *n*-BuZnCl with **4[Bu₄N]** with different mole ratios of *n*-BuZnCl to **4[Bu₄N]**. Conditions: 7.5 mol% G3-BrettPhos, THF, 70 °C, 48–72 h. Spectra correspond to Table S1, entries 5–8. Peaks corresponding to **2a** are highlighted.

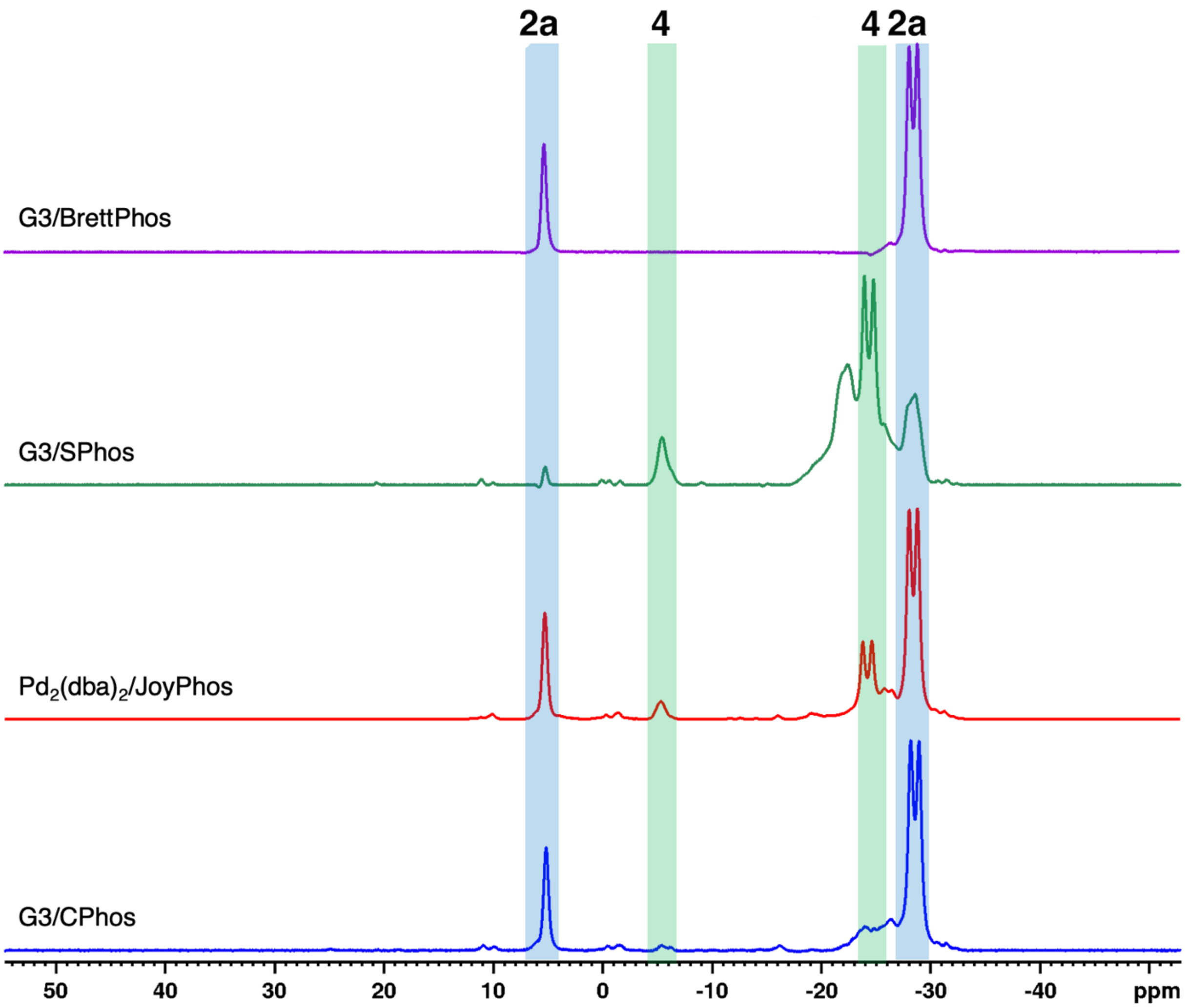

Figure S3. ^{11}B NMR spectra (160 MHz, CD_3CN) of crude reaction mixtures in the reaction of *n*-BuZnCl with **4[Bu$_4$N]**. Conditions: *n*-BuZnCl:**4[Bu$_4$N]** 20:1, 7.5 mol% catalyst (1:1 Pd:ligand), THF, 70 °C, 72 h. Spectra correspond to Table S1, entries 8–11. Peaks corresponding to **2a** and **4** are highlighted.

Preparation of [*closo*-B$_{10}$H$_8$-1,10-(Alkyl)$_2$][Bu$_4$N]$_2$ (2[Bu$_4$N]) and [*closo*-B$_{10}$H$_9$-1-Alkyl][Bu$_4$N]$_2$ (1[Bu$_4$N]). General procedure. $ZnCl_2$ and LiCl were dried overnight in high vacuum in an oil bath at 140 °C and immediately transferred to a glovebox. In the glovebox, dried $ZnCl_2$ (240 mg, 1.76 mmol) and LiCl (75 mg, 1.76 mmol) were added to a dried 10 mL vial equipped with a magnetic stir bar. THF (1.5 mL) was added and the resulting mixture was stirred until a clear solution was formed (usually 10-15 min). To the resulting solution alkylmagnesium halide solution (1.17 mmol) was added and the reaction was allowed to stir in the glovebox at room temperature for 3 h. In the glovebox, another 10 mL vial equipped with a magnetic stir bar was charged with [*closo*-B$_{10}$H$_8$-1,10-I$_2$][Bu$_4$N]$_2$ (**4[Bu$_4$N]**,[2] 50.0 mg, 0.0585 mmol) and BrettPhos Pd G3 (4.0 mg, 7.5 mol%). THF (1.5 mL) was added to the solids and the resulting mixture was

stirred for 15 min followed by addition of *n*-butylzinc chloride solution (*via* a syringe). The resulting solution was transferred to a pressure tube, closed, removed from the glovebox and stirred at 70 ^{0}C for 72 h. After this time the reaction mixture was cooled down, quenched with water in an ice bath and transferred in MeOH into a round-bottom flask. THF was evaporated and CH_2Cl_2 followed by saturated NH_4Cl solution were added. The organic phase was separated and the aquous phase was extracted with CH_2Cl_2 (2 × 10 mL). Combined organic extracts were washed with H_2O and dried (Na_2SO_4). The solvent was evaporated and the crude product was purified on a short neutral alumina plug by eluting it first with CH_2Cl_2 (elution of organic impurities), followed by MeOH. The resulting methanolic solution was concentrated to give >90% pure (based on ^{11}B NMR) [*closo*-$B_{10}H_8$-1,10-(Alkyl)$_2$][Bu_4N]$_2$ (**2[Bu_4N]**) as light brownish oily products solidifying on standing.

Monoalkyl derivatives [*closo*-$B_{10}H_9$-1-Alkyl][Bu_4N]$_2$ (**1[Bu_4N]**) were obtained according to the above procedure using [*closo*-$B_{10}H_9$-1-I][Bu_4N]$_2$ (**3[Bu_4N]**) and half ammounts of $ZnCl_2$, LiCl, BrettPhos Pd-G3, and alkylmagnesium halide.

[*closo*-$B_{10}H_9$-1-C_4H_9][Bu_4N]$_2$ (1a[Bu_4N]). Product was obtained in 87% yield (33.6 mg) from [*closo*-$B_{10}H_9$-1-I][Bu_4N]$_2$ (**3[Bu_4N]**,[2] 42.6 mg, 0.0585 mmol), *n*-butylmagnesium chloride (293 μL, 0.585 mmol, 2.0 M in THF), $ZnCl_2$ (120 mg, 0.88 mmol), LiCl (37.5 mg, 0.88 mmol) and BrettPhos Pd G3 (2.0 mg, 3.75 mol%) as a light brownish oil solidifying on standing. ^{1}H NMR (500 MHz, CD_3CN) δ 3.10 (pseudo t, J = 8.6 Hz, 16H), 2.70 (br q, J = 140 Hz, 1H), 1.83–1.74 (m, 2H), 1.66–1.48 (m, 20H), 1.36 (sext, J = 7.4 Hz, 16H), 0.98 (t, J = 7.4 Hz, 3H), 0.96 (t, J = 7.3 Hz, 24H), –0.09 (br q, J = 117 Hz, 8H); ^{13}C{^{1}H} NMR (126 MHz, CD_3CN) δ 59.2, 37.6, 27.7, 24.3, 20.3, 15.0, 13.8 (C nucleus attached to the cage was not observed); ^{11}B NMR (160 MHz, CD_3CN) δ 10.4 (s, 1B), -6.3 (d, J = 142 Hz, 1B), -28.6 (d, J = 124 Hz, 4B), -29.4 (d, J = 129 Hz, 4B); IR (ATR) ν 2959, 2874, 2432 (BH), 1477, 1382, 884, 741 cm^{-1}; HRMS (ESI, –) *m/z* calcd. for $C_4H_{18}B_{10}$: 176.2339 [M]$^+$, found: 176.2339. Anal. Calcd. for $C_{36}H_{90}B_{10}N_2$: C, 65.59; H, 13.76; N, 4.25. Found: C, 64.12; H, 13.82; N, 4.29.

[*closo*-$B_{10}H_9$-1-C_8H_{17}][Bu_4N]$_2$ (1b[Bu_4N]). Product was obtained in 93% yield (38.7 mg) from [*closo*-$B_{10}H_9$-1-I][Bu_4N]$_2$ (**3[Bu_4N]**,[2] 42.6 mg, 0.0585 mmol), *n*-octylmagnesium bromide (293 μL, 0.585 mmol, 2.0 M in Et_2O), $ZnCl_2$ (120 mg, 0.88 mmol), LiCl (37.5 mg, 0.88 mmol) and BrettPhos Pd G3 (2.0 mg, 3.75 mol%) as a light brownish oil. ^{1}H NMR (500 MHz, CD_3CN)

δ 3.11 (pseudo t, J = 8.6 Hz, 16H), 2.69 (br q, J = 140 Hz, 1H), 1.83–1.74 (m, 2H), 1.60 (quint, J = 8.0 Hz, 16H), 1.51 (quint, J = 7.3 Hz, 2H), 1.43–1.17 (m, 26H), 0.96 (t, J = 7.4 Hz, 24H), 0.90 (t, J = 6.8 Hz, 3H), –0.09 (br q, J = 115 Hz, 8H); $^{13}C\{^1H\}$ NMR (126 MHz, CD_3CN) δ 59.2, 35.3, 35.1, 32.9, 31.1, 30.5, 24.4, 23.4, 20.3, 14.5, 13.9 (C nucleus attached to the cage was not observed); ^{11}B NMR (128 MHz, CD_3CN) δ 10.3 (s, 1B), –6.1 (d, J = 140 Hz, 1B), –28.4 (d, J = 127 Hz, 4B), –29.2 (d, J = 127 Hz, 4B); IR (ATR) v 2958, 2926, 2872, 2424 (BH), 1478, 1379, 880, 732 cm^{-1}; HRMS (ESI, –) *m/z* calcd. for $C_8H_{26}B_{10}$: 232.2965 $[M]^+$, found: 232.2977. Anal. Calcd. for $C_{40}H_{98}B_{10}N_2$: C, 67.16; H, 13.81; N, 3.92. Found: C, 64.43; H, 14.09; N, 3.73.

[*closo*-$B_{10}H_8$-1,10-$(C_4H_9)_2$][Bu_4N]$_2$ (2a[Bu_4N]). Product was obtained in 92% yield (38.5 mg) from [*closo*-$B_{10}H_8$-1,10-I_2][Bu_4N]$_2$ (**4[Bu_4N]**,[2] 50.0 mg, 0.0585 mmol) and *n*-butylmagnesium chloride (585 μL, 1.17 mmol, 2.0 M in THF) as a light brownish oil solidifying on standing. ^1H NMR (500 MHz, CD_3CN) δ 3.09 (pseudo t, J = 8.5 Hz, 16H), 1.85–1.66 (m, 4H), 1.65–1.46 (m, 24H), 1.35 (sext, J = 7.4 Hz, 16H), 0.97 (t, J = 7.4 Hz, 6H), 0.96 (t, J = 7.3 Hz, 24H), 0.70– –0.70 (br m, 8H); $^{13}C\{^1H\}$ NMR (126 MHz, CD_3CN) δ 59.1, 37.9, 27.6, 24.1, 20.2, 15.0, 13.7 (C nucleus attached to the cage was not observed); ^{11}B NMR (160 MHz, CD_3CN) δ 5.2 (s, 2B), –28.5 (d, J = 120 Hz, 8B); IR (ATR) v 2956, 2874, 2438 (BH), 1475, 1382, 883, 739 cm^{-1}; HRMS (ESI, –) *m/z* calcd. for $C_8H_{26}B_{10}$: 232.2965, found: 232.2979 $[M]^+$. Anal. Calcd. for $C_{40}H_{98}B_{10}N_2$: C, 67.16; H, 13.81; N, 3.92. Found: C, 65.38; H, 14.03; N, 4.05.

[*closo*-$B_{10}H_8$-1,10-$(C_8H_{17})_2$][Bu_4N]$_2$ (2b[Bu_4N]). Product was obtained in 84% yield (40.7 mg) from [*closo*-$B_{10}H_8$-1,10-I_2][Bu_4N]$_2$ (**4[Bu_4N]**,[2] 50.0 mg, 0.0585 mmol) and *n*-octylmagnesium bromide (585 μL, 1.17 mmol, 2.0 M in Et_2O) as a light brownish oil. ^1H NMR (500 MHz, CD_3CN) δ 3.09 (pseudo t, J = 8.6 Hz, 16H), 1.80–1.72 (m, 4H), 1.67–1.46 (m, 24H), 1.44–1.16 (m, 32H), 0.96 (t, J = 7.4 Hz, 27H), 0.90 (t, J = 6.9 Hz, 3H), 0.70 – –0.70 (br m, 8H); $^{13}C\{^1H\}$ NMR (126 MHz, CD_3CN) δ 59.3 (t, J = 2.9 Hz), 35.6, 35.1, 32.9, 31.1, 30.6, 24.3, 23.5, 20.3, 14.4, 13.8 (C nucleus attached to the cage was not observed); ^{11}B NMR (160 MHz, CD_3CN) δ 6.0 (s, 2B), -27.7 (d, J = 124 Hz, 8B); IR (ATR) v 2959, 2874, 2430 (BH), 1475, 1382, 883, 739 cm^{-1}; HRMS (ESI, –) *m/z* calcd. for $C_{16}H_{42}B_{10}$: 344.4217 $[M]^+$, found: 344.4246. Anal. Calcd. for $C_{48}H_{114}B_{10}N_2$: C, 69.67; H, 13.89; N, 3.39. Found: C, 68.87; H, 13.99; N, 3.42.

[*closo*-$B_{10}H_8$-1,10-$(C_{12}H_{25})_2$][Bu_4N]$_2$ (2c[Bu_4N]). Product was obtained in 58% yield (31.9 mg) from [*closo*-$B_{10}H_8$-1,10-I_2][Bu_4N]$_2$ (**4[Bu_4N]**,[2] 50.0 mg, 0.0585 mmol) and *n*-dodecylmagnesium bromide (1.17 mL, 1.17 mmol, 1.0 M in Et_2O) as a light brownish oil. ^1H NMR

(500 MHz, CD_3CN) δ 3.12 (pseudo t, J = 8.6 Hz, 16H), 1.83–1.68 (m, 4H), 1.68–1.52 (m, 24H), 1.51–1.42 (m, 4H), 1.39–1.16 (m, 44H), 0.95 (t, J = 7.4 Hz, 24H), 0.86 (t, J = 6.6 Hz, 6H), 0.70 – –0.70 (br m, 8H); $^{13}C\{^1H\}$ NMR (126 MHz, CD_3CN) δ 59.2 (t, J = 2.9 Hz), 35.5, 35.0, 32.5, 31.1, 30.8, 30.6, 30.4, 30.3, 30.0, 24.3, 23.3, 20.2, 14.3, 13.7 (C nucleus attached to the cage was not observed); ^{11}B NMR (160 MHz, CD_3CN) δ 5.3 (s, 2B), -28.7 (d, J = 120 Hz, 8B); IR (ATR) v 2959, 2875, 2436 (BH), 1467, 1382, 883, 739 cm^{-1}; HRMS (ESI, –) *m/z* calcd. for $C_{24}H_{58}B_{10}$: 456.5469 $[M]^+$, found: 456.5506. Anal. Calcd. for $C_{56}H_{130}B_{10}N_2$: C, 71.57; H, 13.94; N, 2.98. Calcd. for $C_{56}H_{130}B_{10}N_2$•H_2O: C, 70.23; H, 13.89; N, 2.92. Found: C, 68.93; H, 14.98; N, 3.25.

Preparation of $[Ph_4P]^+$ salts for XRD characterization. To a solution of [*closo*-$B_{10}H_9$-1-C_4H_9][Bu_4N]$_2$ (**1a[Bu₄N]**, 0.05 mmol) or [*closo*-$B_{10}H_8$-1,10-(C_4H_9)$_2$][Bu_4N]$_2$ (**2a[Bu₄N]**, 0.05 mmol) in MeOH (1 mL) [Ph_4P]Cl (37.5 mg, 0.10 mmol) was added. Immediately, a yellow precipitate was formed, which was filtered off, washed with MeOH (1 mL) and dried in air. To obtain XRD quality crystals, the product was dissolved in hot MeOH, the resulting hot solution was slowly cooled and left for slow concentration.

[*closo*-$B_{10}H_9$-1-C_4H_9][Ph_4P]$_2$ (1a[Ph_4P]). Product was obtained in 46% yield (19.6 mg) from [*closo*-$B_{10}H_9$-1-C_4H_9][Bu_4N]$_2$ (**1a[Bu_4N]**, 33.0 mg, 0.05 mmol) as a yellow solid, which was recrystallized from MeOH. ^1H NMR (500 MHz, CD_3CN) δ 7.95–7.89 (m, 8H), 7.81–7.61 (m, 32H), 2.70 (br q, J = 139 Hz, 1H), 1.86–1.77 (m, 2H), 1.68–1.57 (br m, 2H), 1.52 (sext, J = 7.3 Hz, 2H), 0.96 (t, J = 7.4 Hz, 3H), –0.08 (br q, J = 121 Hz, 8H); $^{13}C\{^1H\}$ NMR (101 MHz, CD_3CN) δ 136.3 (d, J = 3.1 Hz), 135.6 (d, J = 10.5 Hz), 131.3 (d, J = 13.0 Hz), 118.8 (d, J = 90 Hz), 37.6, 27.6, 15.0 (C nucleus attached to the cage was not observed); ^{11}B NMR (128 MHz, CD_3CN) δ 10.4 (s, 1B), –6.0 (d, J = 138 Hz, 1B), –28.4 (d, J = 119 Hz, 4B), –29.2 (d, J = 118 Hz, 4B); IR (ATR) v 3058, 2916, 2429 (BH), 1584, 1435, 1208, 1104, 687 cm^{-1}. Anal. Calcd. for $C_{52}H_{58}B_{10}P_2$: C, 73.21; H, 6.85. Found: C, 73.19; H, 6.78.

[*closo*-$B_{10}H_8$-1,10-(C_4H_9)$_2$][Ph_4P]$_2$ (2a[Ph_4P]). Product was obtained in 33% yield (15.0 mg) from [*closo*-$B_{10}H_9$-1,10-(C_4H_9)$_2$][Bu_4N]$_2$ (**2a[Bu_4N]**, 35.8 mg, 0.05 mmol) and [Ph_4P]Cl (37.5 mg, 0.10 mmol) as a yellow solid, which was recrystallized from MeOH. ^1H NMR (500 MHz, CD_3CN) δ 7.92 (t, J = 7.0 Hz, 8H), 7.82–7.60 (m, 32H), 1.80–1.69 (m, 4H), 1.66–1.54 (br m, 4H), 1.50 (sext, J = 7.4 Hz, 4H), 0.95 (t, J = 7.4 Hz, 6H), 0.60– –0.70 (br m, 8H); $^{13}C\{^1H\}$ NMR (126

MHz, CD_3CN) δ 136.3 (d, J = 2.4 Hz), 135.6 (d, J = 10.2 Hz), 131.3 (d, J = 12.6 Hz), 118.8 (d, J = 90 Hz), 37.9, 27.6, 15.0 (C nucleus attached to the cage was not observed); ^{11}B NMR (160 MHz, CD_3CN) δ 5.2 (s, 2B), –28.5 (d, J = 122 Hz, 8B); IR (ATR) ν 2945, 2876, 2417 (BH), 1749, 1584, 1435, 1106, 687, 524 cm^{-1}. Anal. Calcd. for $C_{56}H_{66}B_{10}P_2$: C, 73.98; H, 7.32. Found: C, 73.62; H, 7.19.

^{11}B NMR analysis of crystalline ion pair demonstrated essentially no sample degradation after 18 months of storage under ambient conditions.

Attempted cation exchange in [*closo*-$B_{10}H_8$-1,10-$(C_4H_9)_2$][Bu_4N]$_2$ (2a[Bu_4N]). Purified dibutyl derivative **2a[Bu_4N]** (20.9 mg, 0.023 mmol) was suspended in 10% aq. HCl and the mixture was extracted 3× with diethyl ether. Water was added to the collected ether fractions, and ether was evaporated. The remaining aqueous mixture was treated with [Et_4N]Cl giving a milky suspension, which was extracted 3× with CH_2Cl_2. The extracts were combined, dried (Na_2SO_4) and evaporated. The viscous oily residue was analysed by ^{11}B{^{1}H} NMR spectroscopy showing 7 main signals (see Fig. S7), indicating cage opened product, which was not investigated further.

Preparation of pyridinium ion pairs of anion 2b. General procedure. To a solution of [*closo*-$B_{10}H_8$-1,10-$(C_8H_{17})_2$][Bu_4N]$_2$ (**2b[Bu_4N]**, 41.4 mg, 0.05 mmol) in MeOH (1 mL) appropriate pyridinium bromide (0.10 mmol) or C_{12}-paraquat bromide (0.05 mmol) was added. Immediately, a colored precipitate was formed, which was filtered off, washed with MeOH (1 mL) and dried in air.

Ion pair [*closo*-$B_{10}H_8$-1,10-$(C_8H_{17})_2$][*N,N′*-didodecyl-4,4′-bipyridinium] (2b[Q12]). Product was obtained in 55% yield (23.0 mg) from *N,N′*-didodecyl-4,4′-bipyridinium dibromide (32.7 mg, 0.05 mmol) as a dark purple solid sparingly soluble at best in all tested solvents: mp 170–171 °C (MeOH) dec; ^{1}H NMR (400 MHz, CD_3CN) δ 8.92 (br s, 4H), 8.42 (br s, 4H), 4.61 (br s, 4H), 2.13–2.08 (m, 4H), 2.06–1.98 (m, 4H), 1.80–1.69 (m, 4H), 1.65–1.54 (m, 4H), 1.54–1.42 (m, 4H), 1.41–1.04 (m, 48H), 0.96 (t, J = 7.4 Hz, 3H), 0.92–0.83 (m, 9H), 0.80– –0.70 (br m, 8H); ^{11}B NMR (128 MHz, CD_3CN) δ 5.2 (br s, 2B), –28.6 (br s, 8B); IR (ATR) ν 3566, 2920, 2430 (BH), 1744, 1636, 1443, 1377, 1175, 827 cm^{-1}. Anal. Calcd. for $C_{50}H_{100}B_{10}N_2$: C, 71.71; H, 12.04; N, 3.35. Found: C, 70.14; H, 12.36; N, 3.42.

Ion pair [*closo*-$B_{10}H_8$-1,10-(C_8H_{17})$_2$][*N*-decyl-4-cyanopyridinium]$_2$ (2b[PyrCN]). Product was obtained in 57% yield (23.8 mg) from *N*-decyl-4-cyanopyridinium bromide (32.5 mg, 0.10 mmol) as a dark burgundy solid recrystallized from MeOH: mp 148–149 °C (MeOH); ^{1}H NMR (600 MHz, CD_3CN) δ 8.93 (d, J = 6.9 Hz, 4H), 8.30 (d, J = 6.2 Hz, 4H), 4.59 (t, J = 7.6 Hz, 4H), 1.84–1.73 (m, 4H), 1.69–1.56 (m, 4H), 1.56–1.45 (m, 4H), 1.45–1.05 (m, 48H), 0.90 (t, J = 7.3 Hz, 6H), 0.88 (t, J = 7.1 Hz, 6H), 0.70– -0.70 (br m, 8H); ^{13}C{^{1}H} NMR (151 MHz, CD_3CN) δ ^{13}C{^{1}H} NMR (151 MHz, CD_3CN) δ 147.2, 132.2, 128.6, 115.1, 63.9, 35.6, 35.1, 32.9, 32.6, 32.0, 31.1, 30.6, 30.2, 30.1, 30.0, 29.6, 26.5, 23.5, 23.4, 14.5, 14.4 (C nucleus attached to the cage was not observed); ^{11}B NMR (128 MHz, CD_3CN) δ 5.4 (s, 2B), –28.4 (d, J = 123 Hz, 8B); IR (ATR) ν 2919, 2850, 2430 (BH), 1636, 1452, 1262, 1018, 834 cm^{-1}. Anal. Calcd. for $C_{48}H_{92}B_{10}N_4$: C, 69.18; H, 11.13; N, 6.72. Found: C, 68.93; H, 11.20; N, 6.69.

Ion pair [*closo*-$B_{10}H_8$-1,10-(C_8H_{17})$_2$]2[*N*-hexyl-4-($C_{11}H_{23}$OCO)pyridinium] (2b[PyrCOOC$_{11}$]). Product was obtained in 59% yield (31.5 mg) from *N*-hexyl-4-(undecyloxycarbonyl)pyridinium bromide (80.5 mg, 0.10 mmol) as a purple solid: mp 170-173° C (MeOH); ^{1}H NMR (400 MHz, CD_3CN) δ 8.91 (dd, J = 6.6, 3.2 Hz, 4H), 8.39 (d, J = 6.1 Hz, 4H), 4.61 (t, J = 7.6 Hz, 4H), 4.40 (t, J = 6.6 Hz, 4H), 2.02–1.95 (m, 4H), 1.84–1.70 (m, 8H), 1.68–1.55 (m, 4H), 1.55–1.41 (m, 8H), 1.41–1.00 (m, 56H), 0.99–0.80 (m, 18H), 0.70– –0.70 (br m, 8H); ^{13}C{^{1}H} NMR (101 MHz, CD_3CN) δ 162.9, 147.1, 145.7, 128.5, 68.2, 63.2, 35.6, 35.1, 32.9, 32.6, 32.1, 31.8, 31.1, 30.6, 30.3, 30.2, 30.0, 29.9, 29.0, 26.5, 26.2, 23.5, 23.3, 23.1, 14.5, 14.3, 14.2 (C nucleus attached to the cage was not observed); ^{11}B NMR (128 MHz, CD_3CN) δ 4.9 (s, 2B), –28.8 (d, J = 123 Hz, 8B); IR (ATR) ν 3050, 2921, 2431 (BH), 1735, 1636, 1457, 1282, 1120, 688 cm^{-1}. Anal. Calcd. for $C_{62}H_{122}B_{10}N_2O_4$: C, 69.74; H, 11.52; N, 2.62. Found: C, 68.59; H, 11.81; N, 2.90.

Preparation of [*closo*-$B_{10}H_8$-1-IPh-10-Alkyl][Bu_4N] (7[Bu_4N]). General procedure. A solution of [*closo*-$B_{10}H_9$-1-Alkyl][Bu_4N]$_2$ (**1a[Bu_4N]** or **1b[Bu_4N]**, 0.127 mmol) in dry MeCN (3 mL) under argon was cooled to 0 °C and PhI(OAc)$_2$ (51.2 mg, 0.159 mmol) was added in 3 portions every 15 min. Reaction mixture was stirred for 1 h at 0 °C and for 16 h at rt. All volatiles were removed in high vacuum avoiding excess heat (< 20 °C) and the resulting crude product was purified using SiO_2 plug (SiO_2 passivated with [Bu_4N][HSO_4], CH_2Cl_2/ MeCN, 100:1 for R = C_4H_9 or CH_2Cl_2 for R = C_8H_{17}). The eluate was concentrated using a rotevap and cold-water bath (< 20

°C) giving **7[Bu4N]** as a moderately stable yellowish oil used immediately in subsequent steps. Reactions conducted in 50% aq AcOH at 0 °C gave worse results.

[*closo*-$B_{10}H_8$-1-IPh-10-C_4H_9][Bu_4N] (7a[Bu_4N]). Product was obtained in 45% yield (35.4 mg) from [*closo*-$B_{10}H_9$-1-C_4H_9][Bu_4N]$_2$ (**1a[Bu_4N]**, 83.7 mg, 0.127 mmol) as a light brownish oil. ^{1}H NMR (400 MHz, CD_3CN) δ 8.14-8.06 (m, 2H), 7.62–7.53 (m, 1H), 7.45–7.35 (m, 2H), 3.06 (pseudo t, J = 8.6 Hz, 8H), 1.83–1.74 (m, 2H), 1.63–1.52 (m, 10H), 1.40–1.26 (m 10H), 1.02–0.93 (m, 15H), 1.90 – -0.40 (br m, 8H); ^{13}C{^{1}H} NMR (126 MHz, CD_3CN) δ 135.1, 131.5, 130.8, 104.6, 58.5 (t, J = 2.8 Hz), 36.4, 26.7, 23.8, 19.9 (t, J = 1.2 Hz), 14.8, 13.7 (C nucleus attached to the cage was not observed); ^{11}B NMR (161 MHz, CD_3CN) δ 21.5 (s, 1B), –4.2 (s, 1B), –24.0 (d, J = 137 Hz, 4B), –26.9 (d, J = 134 Hz, 4B); IR (ATR) ν 2959, 2874, 2471 (BH), 1462, 1382, 992, 881 cm^{-1}; HRMS (ESI, –) *m/z* calcd. for $C_{10}H_{21}B_{10}I$: 378.1618 [*M*-H]$^+$, found: 378.1641.

[*closo*-$B_{10}H_8$-1-IPh-10-C_8H_{17}][Bu_4N] (7b[Bu_4N]). Product was obtained in 54% yield (46.4 mg) from [*closo*-$B_{10}H_9$-1-C_8H_{17}][Bu_4N]$_2$ (**1b[Bu_4N]**, 90.8 mg, 0.127 mmol) as a light brownish oil. ^{1}H NMR (500 MHz, $CDCl_3$) δ 8.06-8.02 (m, 2H), 7.48 (t, J = 7.4 Hz, 1H), 7.30 (t, J = 7.3 Hz, 2H), 3.00 (pseudo t, J = 8.4 Hz, 8H), 1.96–1.89 (m, 2H), 1.56–1.43 (m, 10H), 1.42-1.24 (m, 18H), 0.94 (t, J = 7.2 Hz, 12H), 0.88 (t, J = 6.8 Hz, 3H), 1.85– -0.15 (br m, 8H); ^{13}C NMR (126 MHz, $CDCl_3$) δ 134.3, 131.2, 130.3, 103.6, 58.7, 34.5, 34.2, 32.3, 30.4, 29.9, 24.1, 22.9, 19.8, 14.3, 13.9 (C nucleus attached to the cage was not observed); ^{11}B NMR (161 MHz, $CDCl_3$) δ 21.5 (s, 1B), -4.6 (br s, 1B), –25.2 (br s, 4B), –28.2 (br s, 4B); IR (ATR) ν 2919, 2874, 2468 (BH), 1469, 1379, 1156, 992, 880 cm^{-1}; HRMS (ESI, –) *m/z* calcd. for $C_{14}H_{29}B_{10}I$: 434.2244 [*M*-H]$^+$, found: 434.2267.

Preparation of [*closo*-$B_{10}H_8$-1-(NC_5H_4-4-OC_7H_{15})-10-C_8H_{17}][Bu_4N] (8b[Bu_4N]). A mixture of [*closo*-$B_{10}H_8$-1-IPh-10-C_8H_{17}][Bu_4N] (**7b[Bu_4N]**, 75.0 mg, 0.105 mmol) and 4-heptyloxypyridine (0.35 mL) was stirred under argon at 50 °C for 2 d. All volatiles were removed *in vacuo* (100 °C, 0.1 mm Hg), the residue was washed with hexanes and passed through a short Florosil plug (starting with CH_2Cl_2/MeCN, 10:1 to elute all impurities, followed by CH_2Cl_2/MeCN, 4:1 to elute the product) giving **8b[Bu_4N]** in 58% yield (40.5 mg) as yellowish oil

solidifying on standing. ^{1}H NMR (400 MHz, CD_3CN) δ 9.24 (d, J = 7.4 Hz, 2H), 7.17 (d, J = 7.4 Hz, 2H), 4.25 (t, J = 6.6 Hz, 2H), 3.07 (pseudo t, J = 8.6 Hz, 8H), 1.89–1.74 (m, 4H), 1.72–1.65 (m, 2H), 1.64–1.54 (m, 8H), 1.52–1.46 (m, 2H), 1.44–1.25 (m, 24H), 0.96 (t, J = 7.3 Hz, 12H), 0.91 (t, J = 6.9 Hz, 3H), 0.90 (t, J = 6.7 Hz, 3H), 0.80– -0.50 (br m, 8H); ^{13}C{^{1}H} NMR (151 MHz, CD_3CN) δ 169.2, 150.2, 112.7, 70.9, 59.3 (t, J = 2.9 Hz), 34.9, 34.7, 32.9, 32.4, 30.9, 30.5, 29.6, 29.3, 26.3, 24.3, 23.5, 23.3, 20.3 (t, J = 1.5 Hz), 14.4, 14.3, 13.8; ^{11}B NMR (128 MHz, CD_3CN) δ 12.9 (s, 1B), 9.6 (br s, 1B), –26.3 (d, J = 130 Hz, 4B), –28.9 (d, J = 136 Hz, 4B); IR (ATR) ν 2918, 2850, 2454 (BH), 1630, 1508, 1457, 1302, 1200, 1034, 838 cm^{-1}; HRMS (ESI, –) *m/z* calcd. for $C_{20}H_{44}B_{10}NO$: 424.4353 [M]$^{+}$, found: 424.4393.

Preparation of ion pair [*closo*-$B_{10}H_8$-1-(NC_5H_4-4-OC_7H_{15})-10-C_8H_{17}][*N*-hexyl-4-($C_6H_{13}OCO$)pyridinium] (8b[PyrCOOC$_6$]). To a solution of [*closo*-$B_{10}H_8$-1-(NC_5H_4-4-OC_7H_{15})-10-C_8H_{17}][Bu_4N] (**8b[Bu$_4$N]**, 34.0 mg, 0.051 mmol) in MeOH (1 mL) *N*-hexyl-4-(hexyloxycarbonyl)pyridinium bromide (19.0 mg, 0.051 mmol) was added. The resulting orange solution was placed in a freezer (-20 °C) for 16 h to form an orange waxy material. The supernatant was decanted, the remaining solid was washed with cold MeOH and dried in vacuum giving **8b[PyrCOOC$_6$]** in 73% yield (26.7 mg) as an orange waxy solid. It was recrystallized again from MeOH (-20 °C). ^{1}H NMR (600 MHz, CD_3CN) δ 9.24 (d, J = 7.3 Hz, 2H), 8.82 (d, J = 6.7 Hz, 2H), 8.41 (d, J = 6.6 Hz, 2H), 7.17 (d, J = 7.4 Hz, 2H), 4.56 (t, J = 7.6 Hz, 2H), 4.41 (t, J = 6.6 Hz, 2H), 4.25 (t, J = 6.6 Hz, 2H), 2.00–1.95 (m, 2H), 1.85 (quint, J = 7.1 Hz, 2H), 1.82–1.75 (m, 4H), 1.71–1.64 (m, 2H), 1.53 (quint, J = 7.2 Hz, 2H), 1.51–1.43 (m, 4H), 1.43–1.37 (m, 4H), 1.37–1.27 (m, 20H), 0.93–0.86 (m, 12H), 0.85– -0.4 (br m, 8H); ^{13}C{^{1}H} NMR (151 MHz, CD_3CN) δ 169.2, 162.9, 150.2, 146.7 (t, J = 8.9 Hz), 128.6, 112.7, 70.9, 68.3, 63.3, 34.9, 34.7, 32.9, 32.4, 32.1, 31.9, 31.7, 30.9, 30.5, 29.6, 29.3, 29.0, 26.3, 26.18, 26.17, 26.21, 23.5, 23.3, 23.2, 23.0, 14.4, 14.32, 14.25, 14.1 (C nucleus attached to the cage was not observed); ^{11}B NMR (193 MHz, CD_3CN) δ 12.9 (s, 1B), 9.7 (br s, 1B), –26.1 (d, J = 132 Hz, 4B), –28.7 (d, J = 131 Hz, 4B); IR (ATR) ν 2919, 2851, 2451 (BH), 1734, 1629, 1507, 1457, 1285, 1198, 1120, 686 cm^{-1}. Anal. Calcd. for $C_{38}H_{74}B_{10}N_2O_3$: C, 63.82; H, 10.43; N, 3.92. Found: C, 63.48; H, 10.50; N, 3.97.

Preparation of ion pair [*closo*-$B_{10}H_8$-1-(NC_5H_4-4-OC_7H_{15})-10-C_8H_{17}][*N*-hexyl-4-($C_{11}H_{23}OCO$)pyridinium] (8b[PyrCOOC$_{11}$]). To a solution of [*closo*-$B_{10}H_8$-1-(NC_5H_4-4-

OC_7H_{15})-10-C_8H_{17}][Bu_4N] (**8b[Bu4N]**, 34.0 mg, 0.051 mmol) in MeOH (1 mL) *N*-hexyl-4-(undecyloxycarbonyl)pyridinium bromide (22.6 mg, 0.051 mmol) was added. Immediately, an orange precipitate was formed. The supernatant was decanted, the remaining solid was washed with cold MeOH and dried in vacuum giving **8b[PyrCOOC11]** in 66% yield (20.3 mg) as an orange waxy solid. It was recrystallized from MeOH avoiding excessive heating the sample (4 °C). ^{1}H NMR (600 MHz, CD_3CN) δ 9.24 (d, J = 7.4 Hz, 2H), 8.82 (d, J = 6.4 Hz, 2H), 8.41 (d, J = 6.1 Hz, 2H), 7.17 (d, J = 7.4 Hz, 2H), 4.57 (t, J = 7.6 Hz, 2H), 4.41 (t, J = 6.6 Hz, 2H), 4.25 (t, J = 6.6 Hz, 2H), 2.00–1.96 (m, 2H), 1.88–1.82 (m, 2H), 1.82–1.76 (m, 4H), 1.71–1.64 (m, 2H), 1.57–1.20 (m, 40H), 0.95–0.85 (m, 12H), 0.80– -0.4 (br m, 8H); ^{13}C{^{1}H} NMR (151 MHz, CD_3CN) δ 169.2, 163.4, 162.8, 150.2, 146.8 (q, J = 7.7 Hz), 128.6, 112.7, 70.9, 68.3, 63.3, 34.9, 34.7, 32.9, 32.6, 32.4, 31.9, 31.7, 30.9, 30.5, 30.28, 30.25, 30.19, 30.0, 29.9, 29.6, 29.2, 29.0, 26.5, 26.3, 26.2, 23.5, 23.34, 23.26, 23.0, 14.4, 14.34, 14.31, 14.1 (C nucleus attached to the cage was not observed); ^{11}B NMR (193 MHz, CD_3CN) δ 12.8 (s, 1B), 9.7 (br s, 1B), –26.0 (d, J = 129 Hz, 4B), –28.7 (d, J = 128 Hz, 4B); IR (ATR) ν 2919, 2851, 2451 (BH), 1734, 1629, 1507, 1457, 1285, 1198, 1120, 686 cm^{-1}. Anal. Calcd. for $C_{43}H_{84}B_{10}N_2O_3$: C, 65.77; H, 10.78; N, 3.57. Found: C, 63.08; H, 10.63; N, 3.63.

Preparation of [{(η5-Cp)(dppe)Fe}-*closo*-$B_{10}H_8$-1-CN-10-C_4H_9][Bu_4N] (9a[Bu4N]). The complex was prepared according to a literature procedure for a related complex.[4] Thus, a solution of [*closo*-$B_{10}H_8$-1-CN-10-C_4H_9][Bu_4N]$_2$ (**10a[Bu4N]**, 11.0 mg, 0.016 mmol) and (η5-Cp)(dppe)FeCl[5] (13.4 mg, 0.024 mmol) in dry CH_2Cl_2 (5 mL) was refluxed for 16 h under inert atmosphere. The resulting red solution was evaporated and the crude product was purified on a silica gel plug using CH_2Cl_2/MeCN, 9:1, as the eluent. The crude product was recrystallized from AcOEt/CH_2Cl_2 mixture giving **9a[Bu4N]** in 66% yield (10.1 mg) as a red oil solidyfying on standing. ^{1}H NMR (600 MHz, CD_2Cl_2) δ 7.97 (t, J = 8.4 Hz, 4H), 7.51 (t, J = 7.4 Hz, 4H), 7.47–7.41 (m, 4H), 7.34 (t, J = 7.5 Hz, 4H), 7.23 (t, J = 7.8 Hz, 4H), 4.33 (s, 5H), 3.05 (pseudo t, J = 8.5 Hz, 8H), 2.71–2.54 (m, 2H), 2.49–2.35 (m, 2H), 1.85–1.75 (m, 2H), 1.74–1.64 (m, 2H), 1.59–1.50 (m 10H), 1.41 (sext, J = 7.3 Hz, 8H), 1.00 (t, J = 7.3 Hz, 12H), 0.98 (t, J = 7.3 Hz, 3H), 0.70– -0.40 (br m, 8H); ^{13}C{^{1}H} NMR (126 MHz, CD_2Cl_2) δ 138.4 (t, J = 20 Hz), 135.6 (t, J = 21 Hz), 133.5 (t, J = 4.0 Hz), 132.6 (t, J = 4.0 Hz), 130.4, 130.2, 129.2 (t, J = 4.0 Hz), 128.7 (t, J = 3.9 Hz), 78.6, 59.2, 36.6, 27.5 (t, J = 21 Hz), 27.3, 24.4, 20.1, 14.8, 13.9 (C nucleus attached to the

cage was not observed); ^{11}B NMR (193 MHz, CD_2Cl_2) δ 20.2 (s, 1B), –16.4 (s, 1B), –25.8 (d, J = 134 Hz, 4B), –28.2 (d, J = 126 Hz, 4B); ^{31}P{^{1}H} NMR (243 MHz, CD_2Cl_2) δ 97.5; IR (ATR) v 2919, 2454 (BH), 2179 (CN), 1433, 1095, 881, 692 cm^{-1}; HRMS (ESI,–) *m/z* calcd. for $C_{36}H_{46}B_{10}FeNP_2$: 720.3385 [M]$^+$, found: 720.3423. Anal. Calcd. for $C_{52}H_{82}B_{10}FeN_2P_2$: C, 64.98; H, 8.60; N, 2.91. Found: C, 64.87; H, 8.56; N, 2.89.

Preparation of [{(η5-Cp)(dppe)Fe}-*closo*-$B_{10}H_8$-1-CN-10-C_4H_9][Bu_4N] (9a[Ph_4P]). To a solution of [{(η5-Cp)(dppe)Fe}-*closo*-$B_{10}H_8$-1-CN-10-C_4H_9][Bu_4N] (**10a[Bu_4N]**, 17.5 mg, 0.018 mmol) in CH_2Cl_2 (2 mL) [Ph_4P]Cl (6.8 mg, 0.018 mmol) was added and the solution was stirred for 30 min at rt. Then, H_2O (2 mL) was added and the resulting mixture was stirred vigorously for 30 min, organic layer was separated and dried (Na_2SO_4). The solvent was evaporated to give **9a[Ph_4P]** in 92% yield (17.7 mg) as a red solid. ^{1}H NMR (600 MHz, CD_2Cl_2) δ 7.97 (t, J = 8.6 Hz, 4H), 7.91 (t, J = 7.6 Hz, 4H), 7.75 (td, J_1 = 7.8 Hz, J_2 = 3.6 Hz, 8H), 7.61 (dd, J_1 = 13.4 Hz, J_2 = 7.7 Hz, 8H), 7.51 (t, J = 7.3 Hz, 4H), 7.48–7.40 (m, 4H), 7.34 (t, J = 8.8 Hz, 4H), 7.23 (t, J = 8.8 Hz, 4H), 4.32 (s, 5H), 2.71–2.57 (m, 2H), 2.49–2.37 (m, 2H), 1.79–1.70 (m, 2H), 1.69–1.62 (m, 2H), 1.46 (sext, J = 7.3 Hz, 2H), 0.92 (t, J = 7.4 Hz, 3H), 0.70– -0.50 (br m, 8H); ^{11}B NMR (193 MHz, CD_2Cl_2) δ 20.7 (s, 1B), –16.5 (s, 1B), –25.7 (d, J = 126 Hz, 4B), –28.1 (d, J = 122 Hz, 4B); ^{31}P{^{1}H} NMR (243 MHz, CD_2Cl_2) δ 98.1, 23.2.

Attempted recrystallization from hot solution in MeOH, EtOH, *i*PrOH, THF, MeOH/CH_2Cl_2, EtOH/CH_2Cl_2, *i*PrOH/CH_2Cl_2, EtOAc/MeCN or by slow evaporation of solution in CH_2Cl_2 and MeCN at or below ambient temperature led only to slow decomposition of the material as evident by ^{11}B and ^{31}P NMR spectroscopy.

Preparation of [*closo*-$B_{10}H_8$-1-CN-10-C_4H_9][Bu_4N]$_2$ (10a[Bu_4N]). In a glovebox, a pressure glass tube was charged with a solution of [*closo*-$B_{10}H_8$-1-IPh-10-C_4H_9][Bu_4N] (**7a[Bu_4N]**, 50.0 mg, 0.081 mmol) in dry MeCN (3 mL), freshly dried (overnight, high vacuum) [Bu_4N][CN] (130 mg, 0.484 mmol) was added, and the tube was sealed. The mixture was stirred at 55–60 °C for 2 days. The progress of the reaction was monitored by ^{11}B NMR. When the starting **7a[Bu_4N]** was no longer present, all volatiles were removed, the resulting crude product was washed with hexane (2×5 mL) and purified using a SiO_2 plug (SiO_2 passivated with [Bu_4N][HSO_4], starting with CH_2Cl_2/MeCN, 10:1 to elute all impurities, followed by

CH_2Cl_2/MeCN, 5:1 to elute the product). The eluate was concentrated giving **10a[Bu$_4$N]** in 30% yield (16.5 mg) as yellowish oil solidifying on standing. ^{1}H NMR (400 MHz, CD_3CN) δ 3.09 (pseudo t, J = 8.6 Hz, 16H), 1.83–1.70 (m, 2H), 1.66–1.49 (m, 20H), 1.36 (sext, J = 7.4 Hz, 16H), 0.97 (t, J = 7.4 Hz, 27H), 0.80– -0.80 (br m, 8H); ^{13}C{^{1}H} NMR (126 MHz, CD_3CN) δ 130.3, 59.3 (t, J = 2.9 Hz), 37.1, 27.4, 24.3, 20.3 (t, J = 1.4 Hz), 14.9, 13.8 (C nucleus attached to the cage was not observed); ^{11}B NMR (128 MHz, CD_3CN) δ 17.4 (s, 1B), -16.0 (s, 1B), -26.0 (d, J = 128 Hz, 4B), -28.0 (d, J = 132 Hz, 4B); IR (ATR) ν 2959, 2872, 2454 (BH), 2167 (CN), 1458, 1379, 877, 737 cm^{-1}; HRMS (ESI,–) *m/z* calcd. for $C_5H_{17}B_{10}N$: 201.2292 [M]$^{+}$, found: 201.2314.

N-Hexyl-4-(undecyloxycarbonyl)pyridinium bromide (**[PyrCOOC$_{11}$]Br**) and *N*-hexyl-4-(hexyloxycarbonyl)pyridinium bromide (**[PyrCOOC$_6$]Br**) were prepared by reaction of appropriate alkyl isonicotinate with *n*-hexyl bromide in MeCN according to a literature procedure for a closely related pyridinium derivative.[6]

N-Decyl-4-cyanopyridinium bromide ([**PyrCN]Br**) was reported recently.[6]

2. NMR spectra

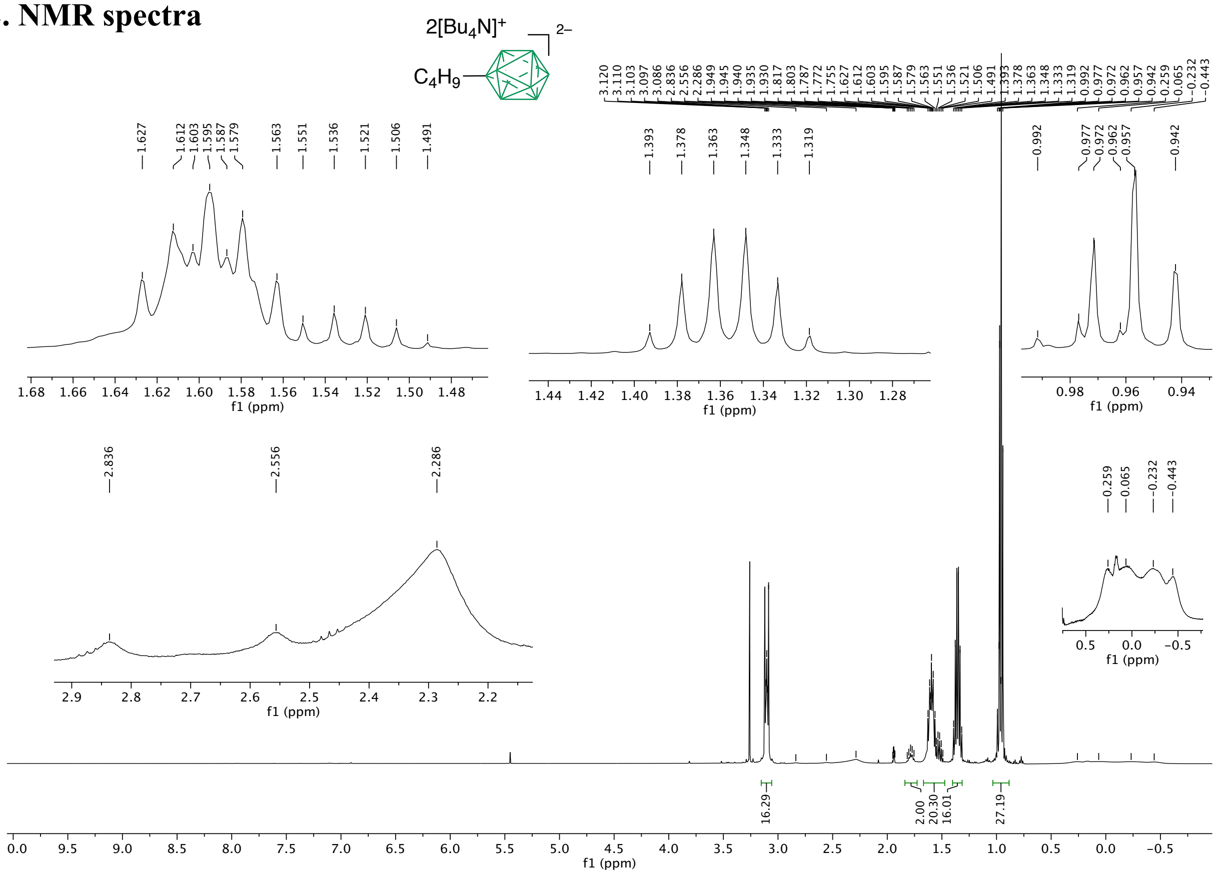

Figure S4. ^{1}H NMR spectrum of [*closo*-$B_{10}H_9$-1-C_4H_9][Bu_4N]$_2$ (**1a[Bu$_4$N]**, 500 MHz, CD_3CN).

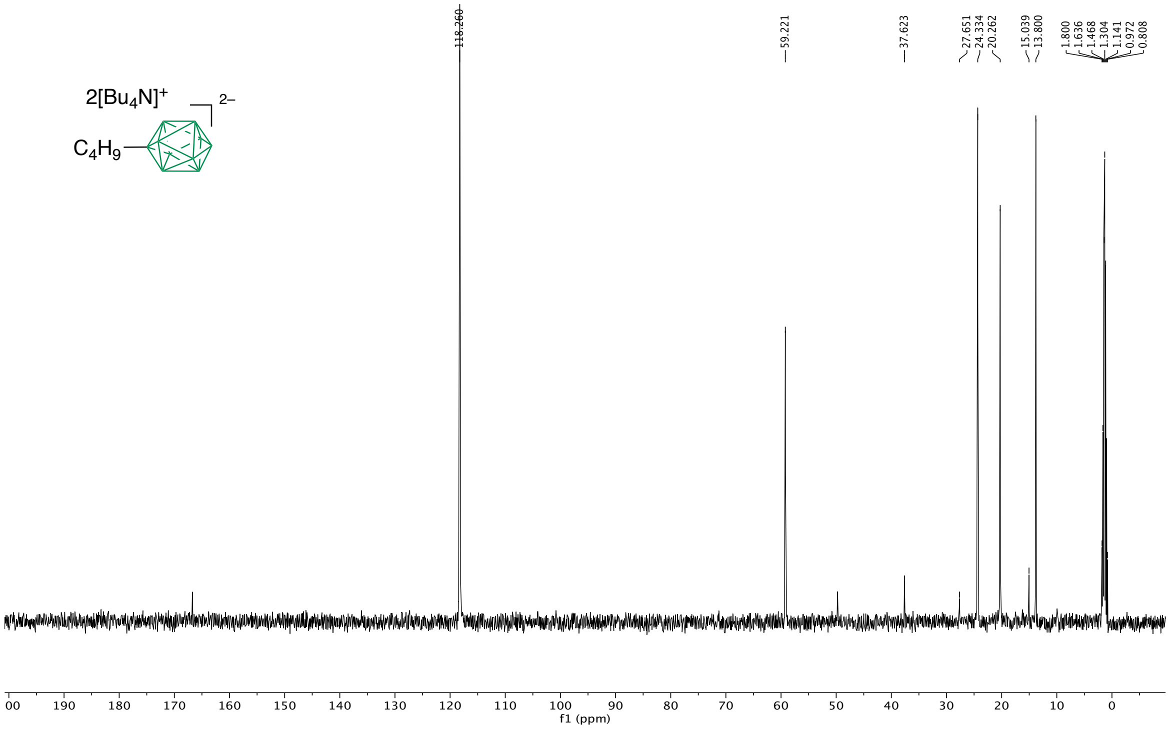

Figure S5. ^{13}C{^{1}H} NMR spectrum of [*closo*-$B_{10}H_9$-1-C_4H_9][Bu_4N]$_2$ (**1a[Bu$_4$N]**, 126 MHz, CD_3CN).

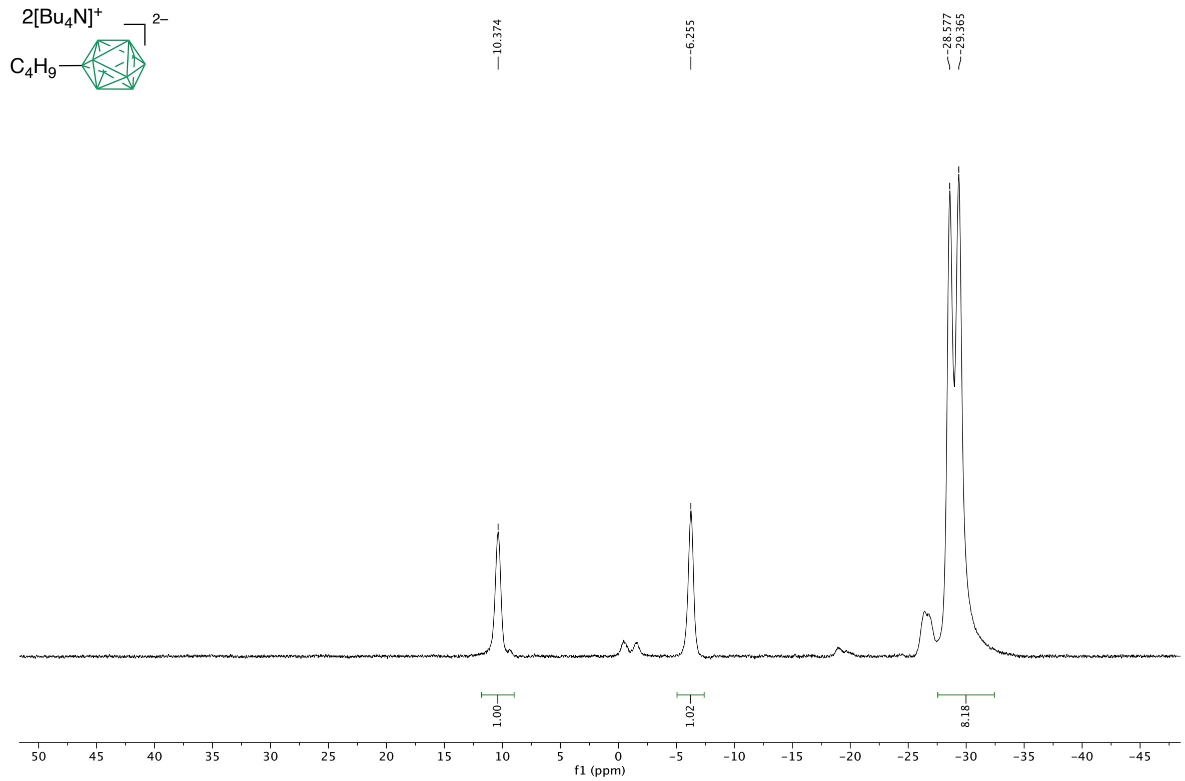

Figure S6. $^{11}B\{^1H\}$ NMR spectrum of purified [*closo*-$B_{10}H_9$-1-C_4H_9][Bu_4N]$_2$ (**1a[Bu$_4$N]**, 160 MHz, CD_3CN).

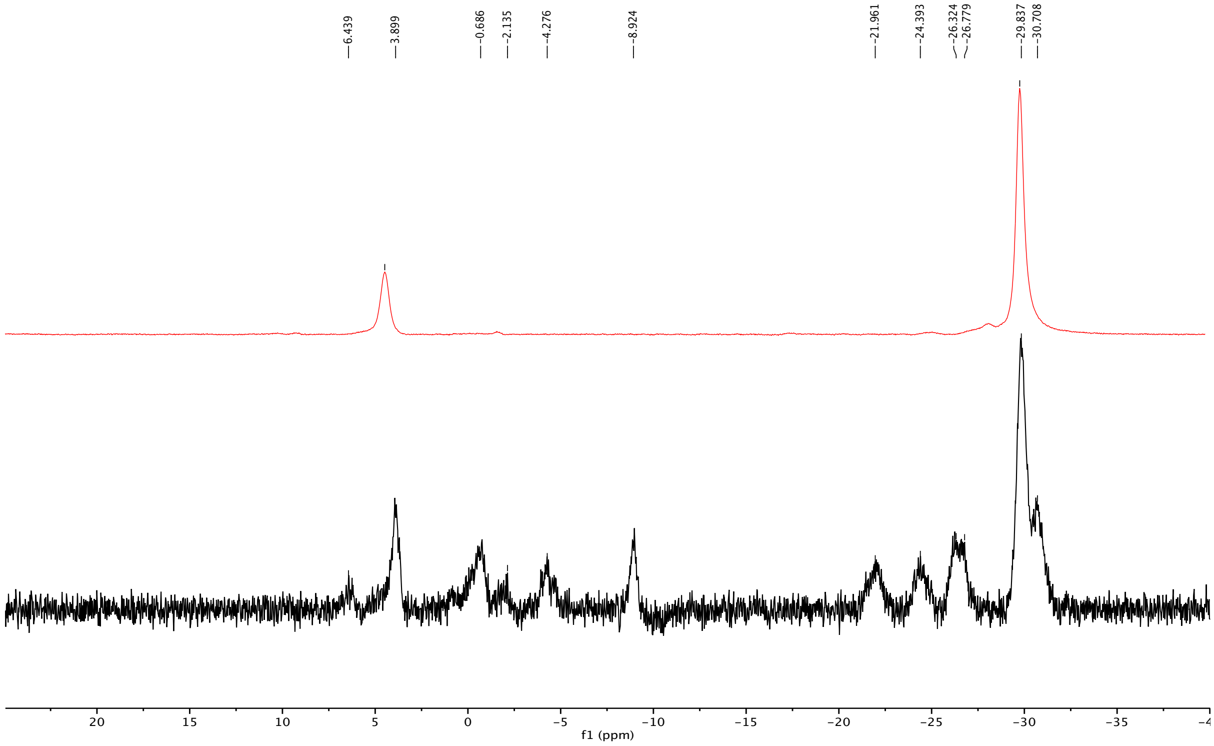

Figure S7. $^{11}B\{^1H\}$ NMR spectra of purified (top, red) and treated with 10% aq. HCl (bottom, black) [*closo*-$B_{10}H_9$-1-C_4H_9][Bu_4N]$_2$ (**1a[Bu$_4$N]**, 160 MHz, CD_3CN).

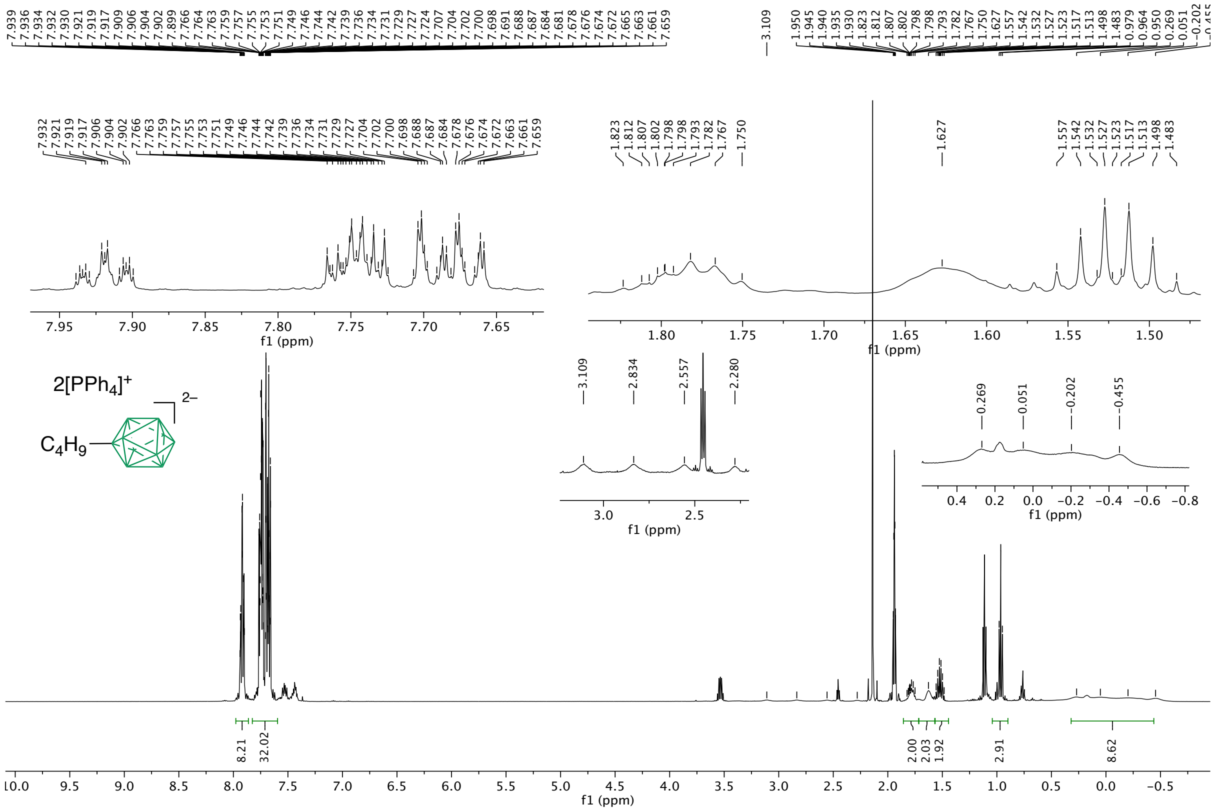

Figure S8. ^{1}H NMR spectrum of [*closo*-$B_{10}H_9$-1-C_4H_9][Ph_4P]$_2$ (**1a[Ph$_4$P]**, 500 MHz, CD_3CN).

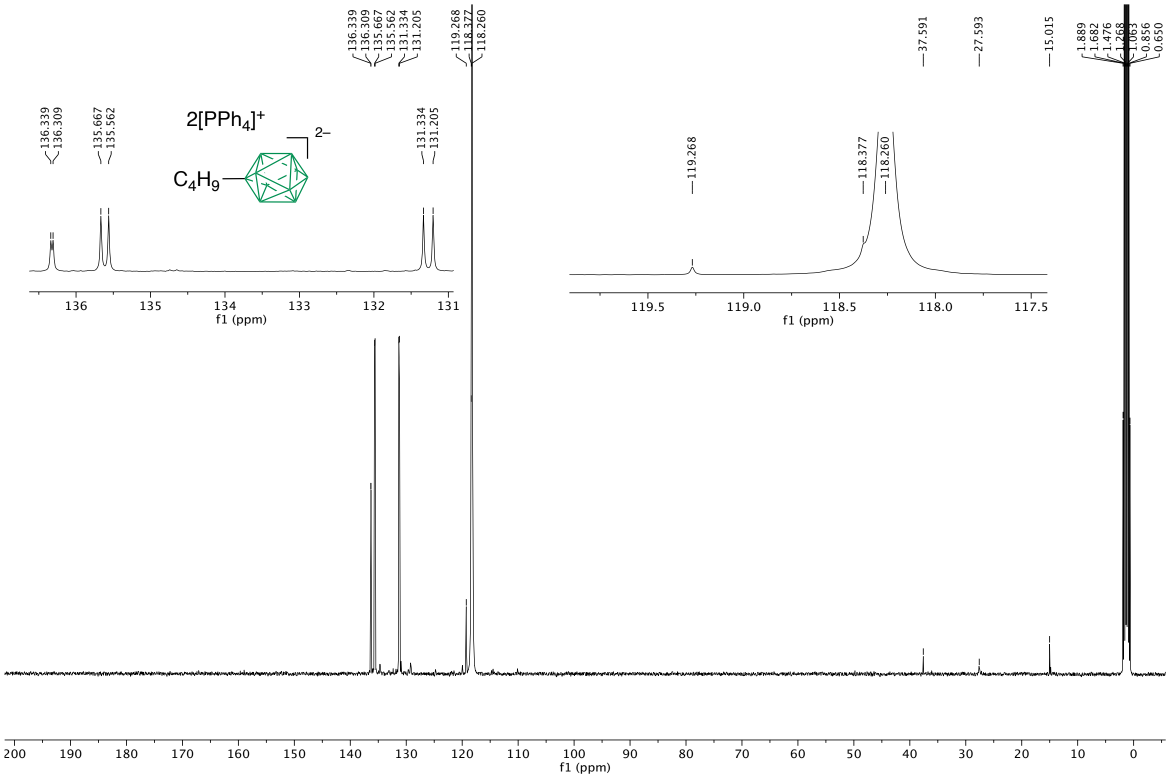

Figure S9. $^{13}C\{^{1}H\}$ NMR spectrum of [*closo*-$B_{10}H_9$-1-C_4H_9][Ph_4P]$_2$ (**1a[Ph$_4$P]**, 101 MHz, CD_3CN).

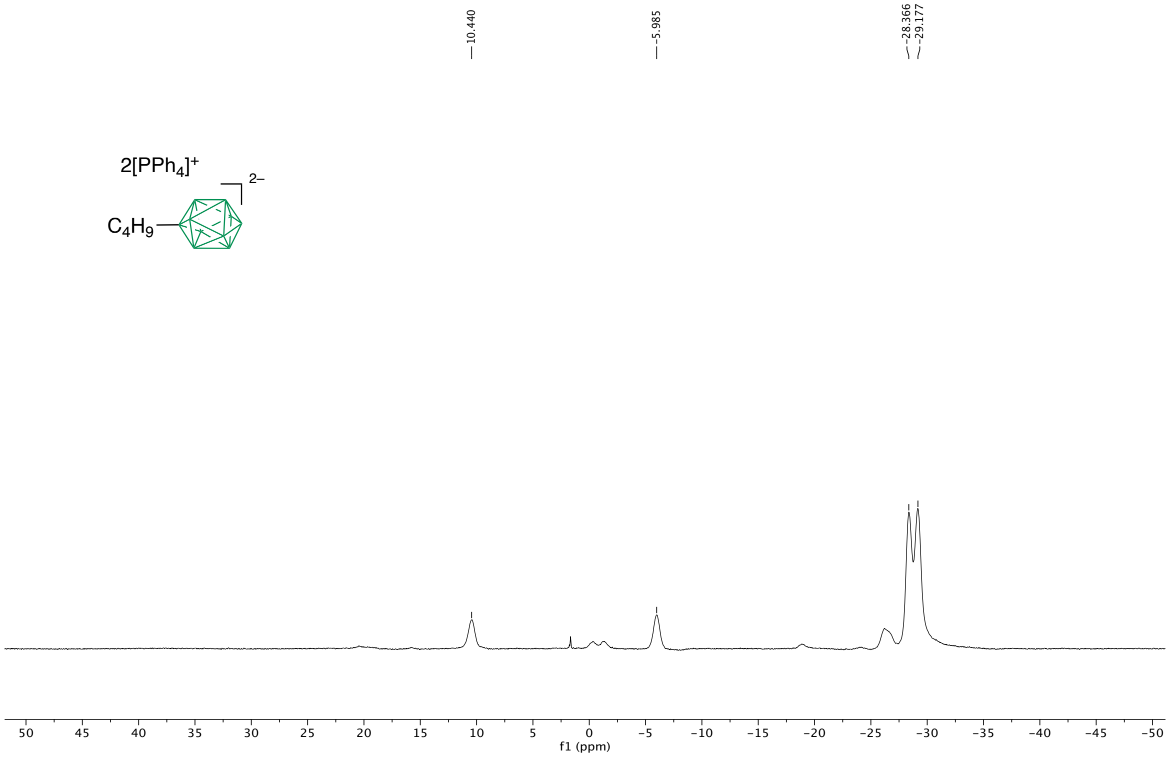

Figure S10. $^{11}B\{^1H\}$ NMR spectrum of [*closo*-$B_{10}H_9$-1-C_4H_9][PPh_4]$_2$ (**1a[Ph$_4$P]**, 128 MHz, CD_3CN).

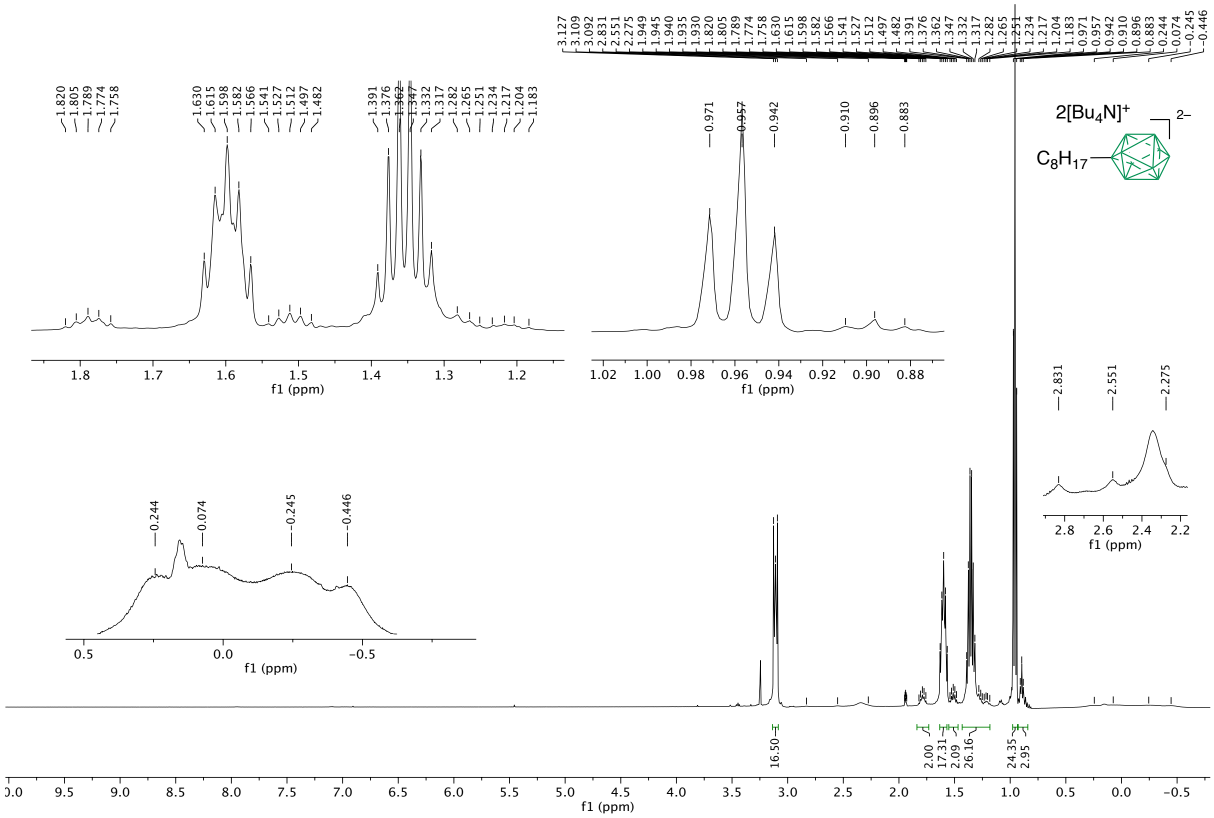

Figure S11. ^1H NMR spectrum of [*closo*-$B_{10}H_9$-1-C_8H_{17}][Bu_4N]$_2$ (**1b[Bu$_4$N]**, 500 MHz, CD_3CN).

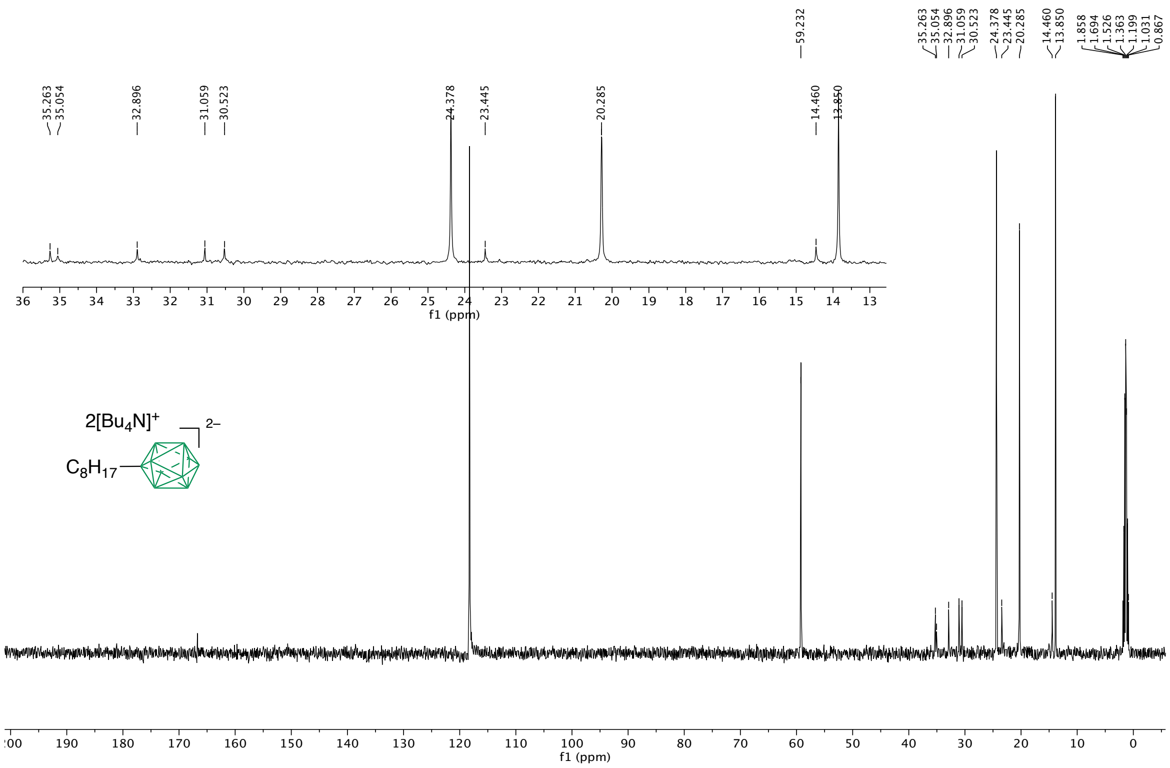

Figure S12. $^{13}C\{^{1}H\}$ NMR spectrum of [*closo*-$B_{10}H_9$-1-C_8H_{17}][Bu_4N]$_2$ (**1b[Bu$_4$N]**, 126 MHz, CD_3CN).

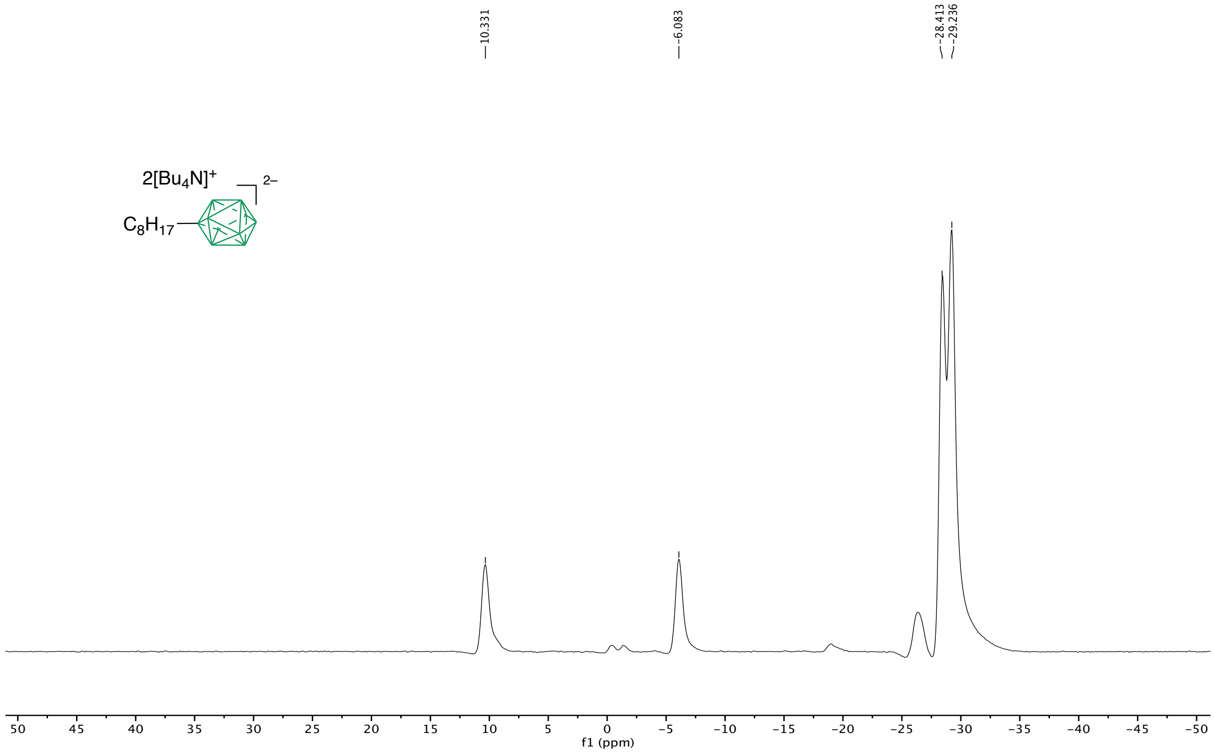

Figure S13. $^{11}B\{^{1}H\}$ NMR spectrum of [*closo*-$B_{10}H_9$-1-C_8H_{17}][Bu_4N]$_2$ (**1b[Bu$_4$N]**, 128 MHz, CD_3CN).

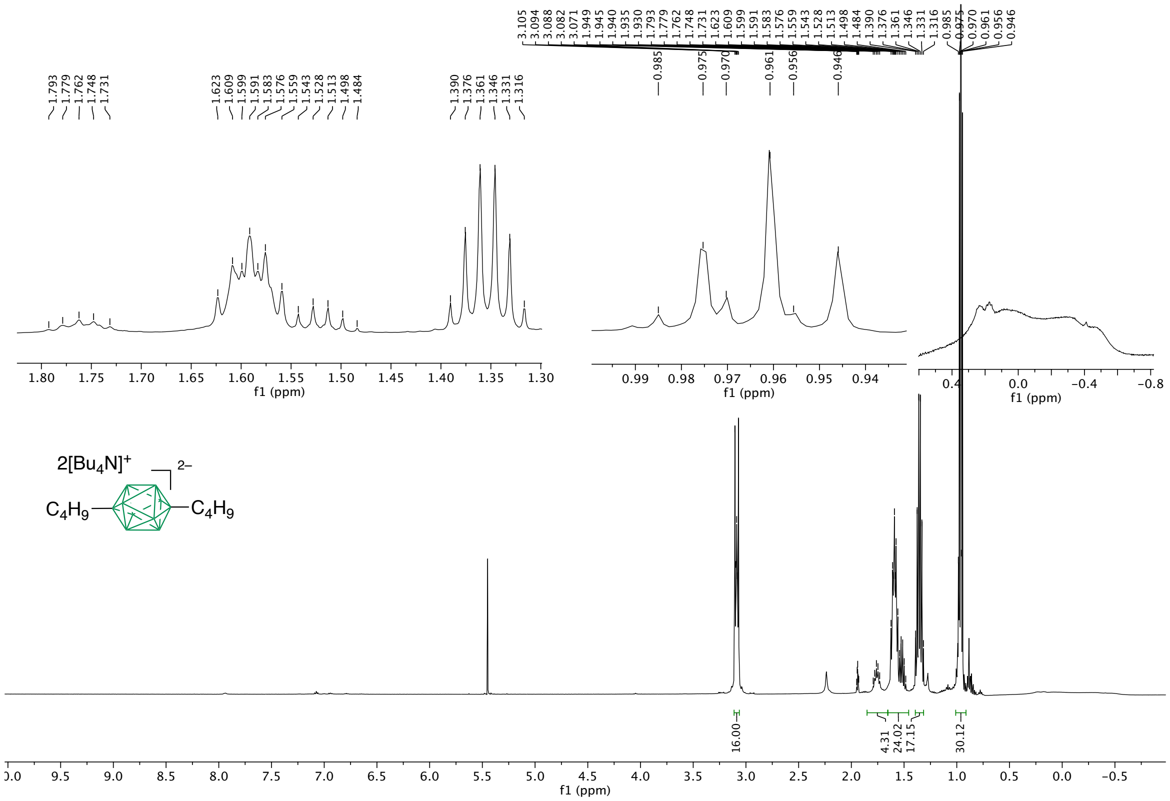

Figure S14. ^{1}H NMR spectrum of [*closo*-$B_{10}H_8$-1,10-$(C_4H_9)_2$][Bu_4N]$_2$ (**2a[Bu4N]**, 500 MHz, CD_3CN).

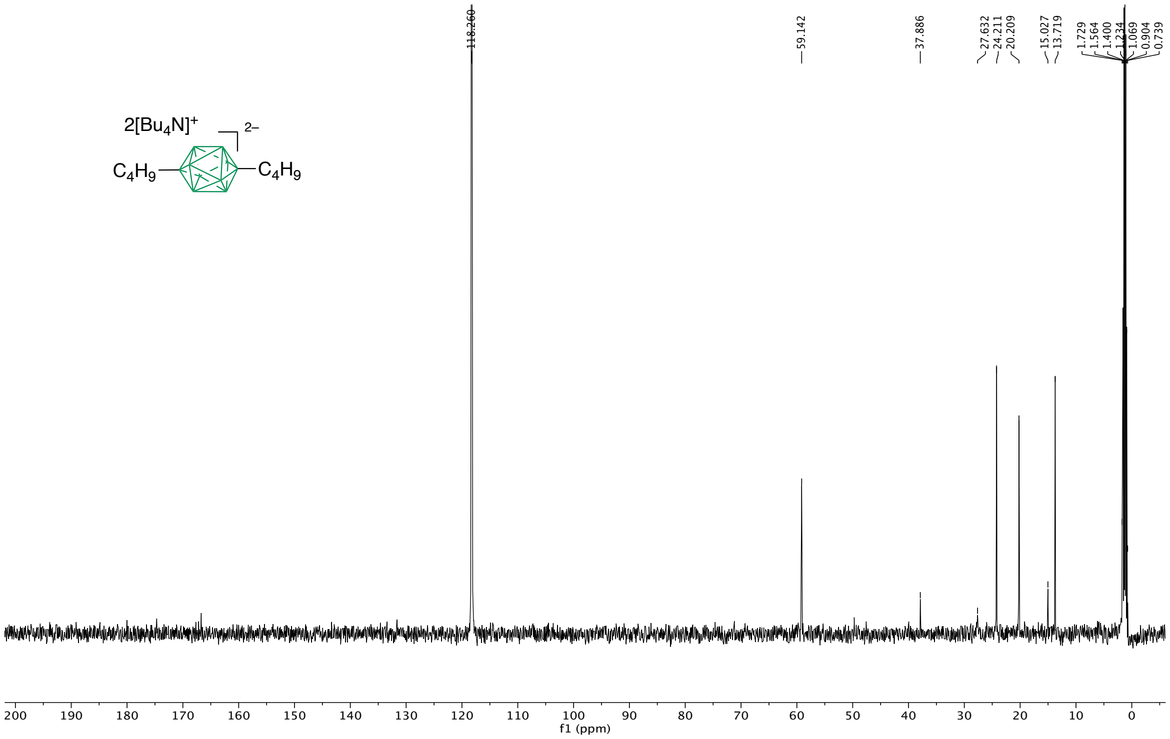

Figure S15. $^{13}C\{^{1}H\}$ NMR spectrum of [*closo*-$B_{10}H_8$-1,10-$(C_4H_9)_2$][Bu_4N]$_2$ (**2a[Bu4N]**, 126 MHz, CD_3CN).

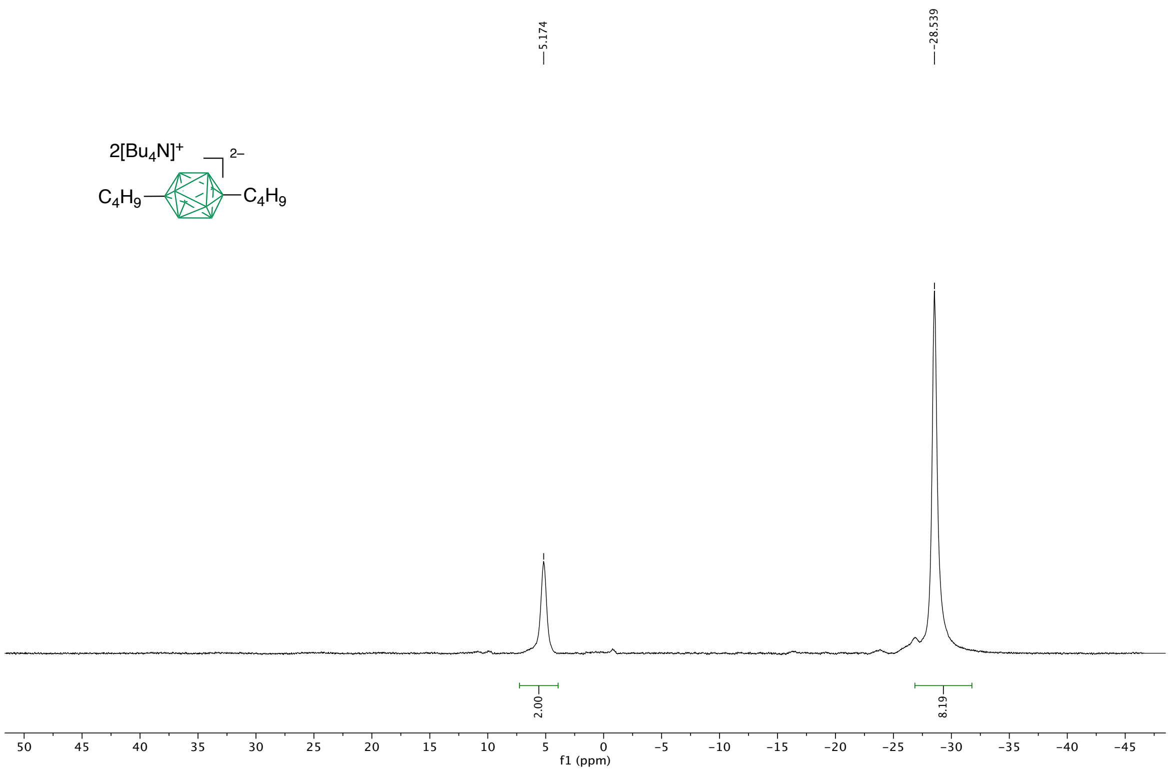

Figure S16. ^{11}B{^{1}H} NMR spectrum of [*closo*-$B_{10}H_8$-1,10-$(C_4H_9)_2$][Bu_4N]$_2$ (**2a[Bu₄N]**, 160 MHz, CD_3CN).

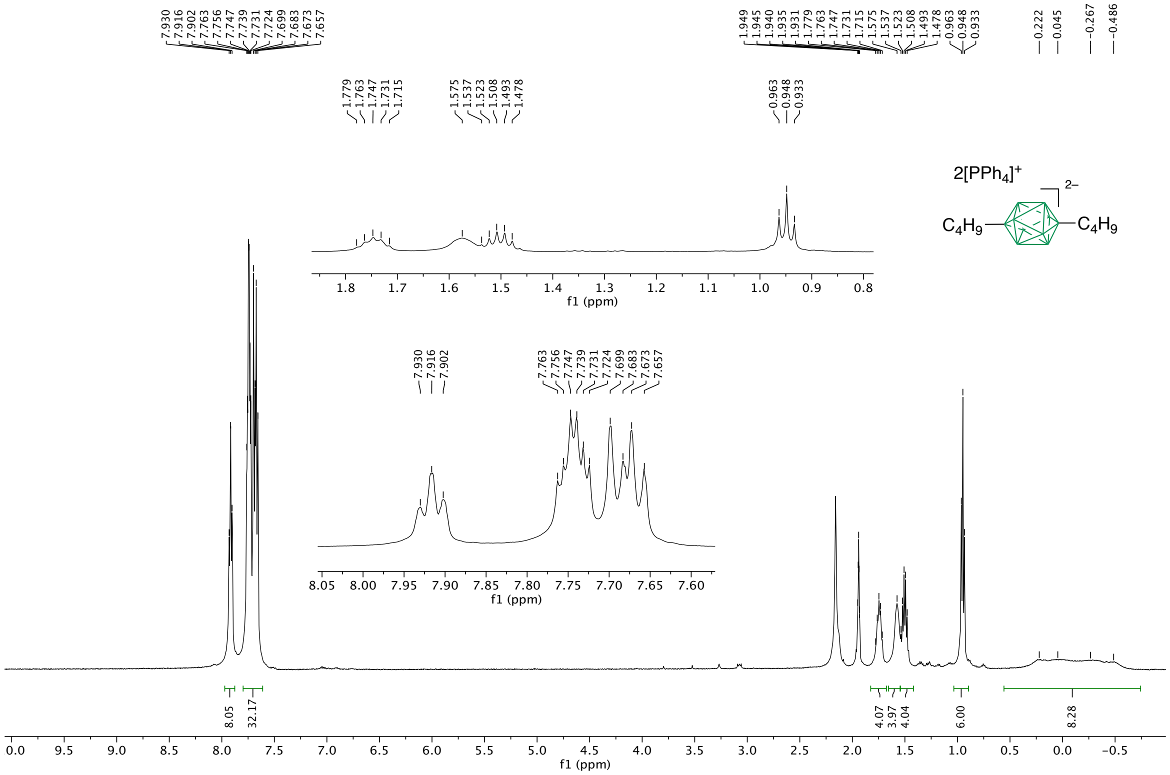

Figure S17. ^{1}H NMR spectrum of [*closo*-$B_{10}H_8$-1,10-$(C_4H_9)_2$][Ph_4P]$_2$ (**2a[Ph₄P]**, 500 MHz, CD_3CN).

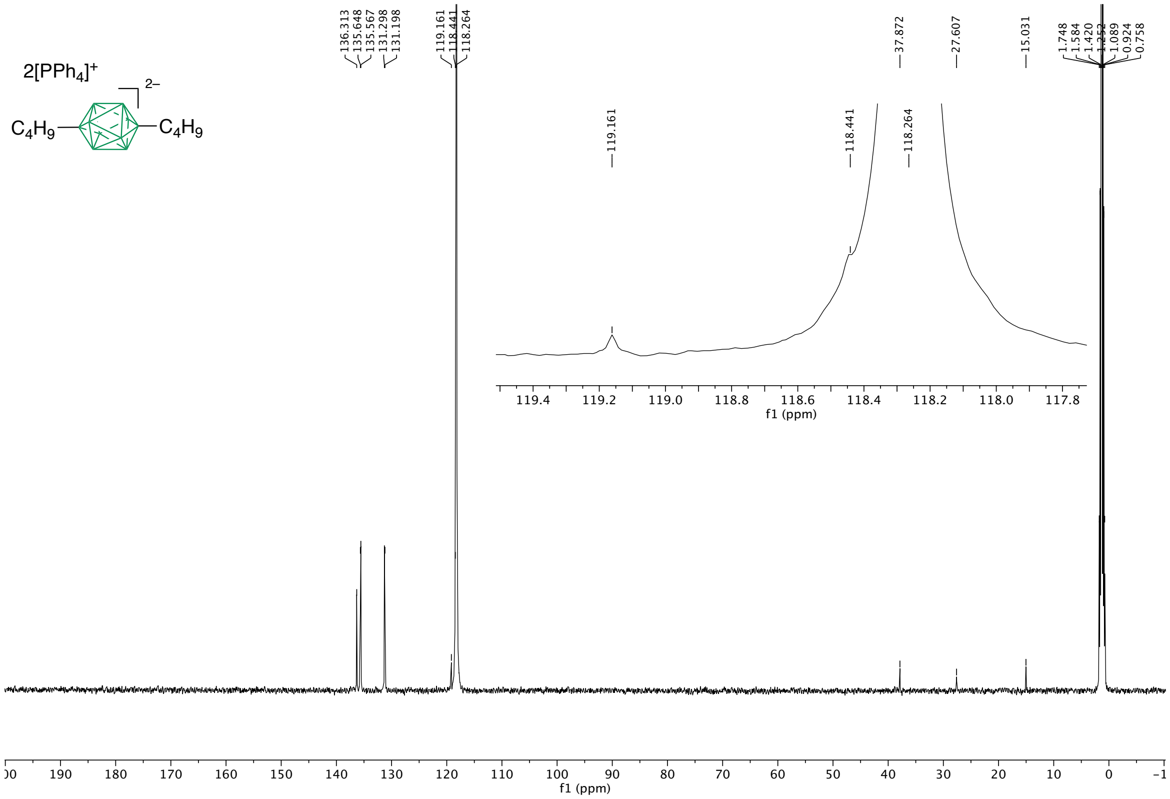

Figure S18. $^{13}C\{^{1}H\}$ NMR spectrum of [*closo*-$B_{10}H_8$-1,10-$(C_4H_9)_2$][Ph_4P]$_2$ (**2a[Ph4P]**, 126 MHz, CD_3CN).

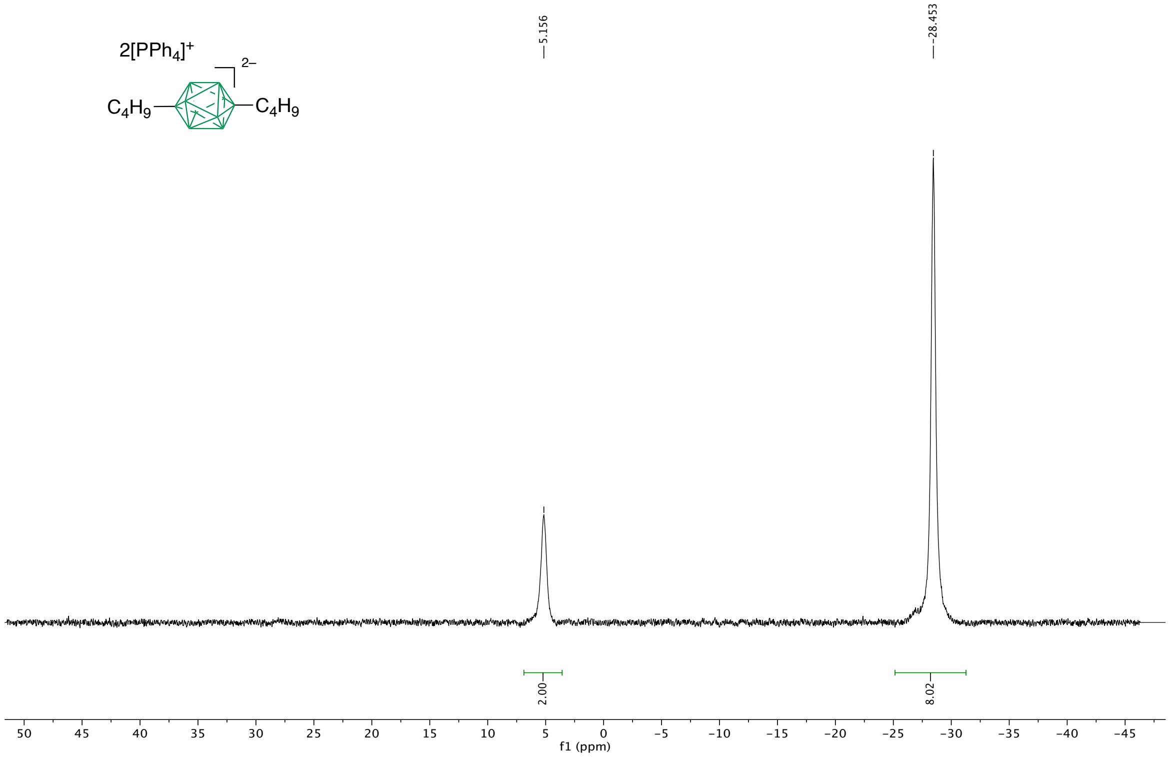

Figure S19. $^{11}B\{^{1}H\}$ NMR spectrum of [*closo*-$B_{10}H_8$-1,10-$(C_4H_9)_2$][PPh_4]$_2$ (**2a[Ph4P]**, 160 MHz, CD_3CN).

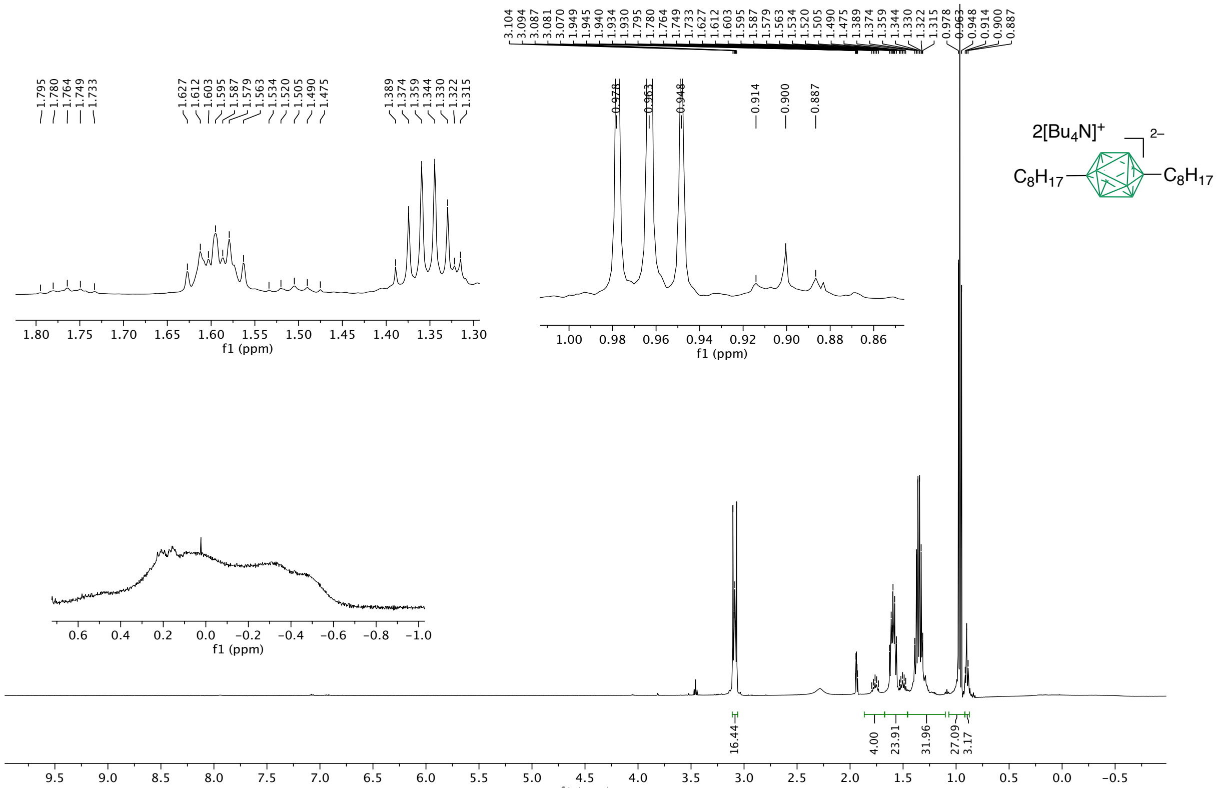

Figure S20. ^{1}H NMR spectrum of [*closo*-$B_{10}H_8$-1,10-$(C_8H_{17})_2$][Bu_4N]$_2$ (**2b[Bu$_4$N]**, 500 MHz, CD_3CN).

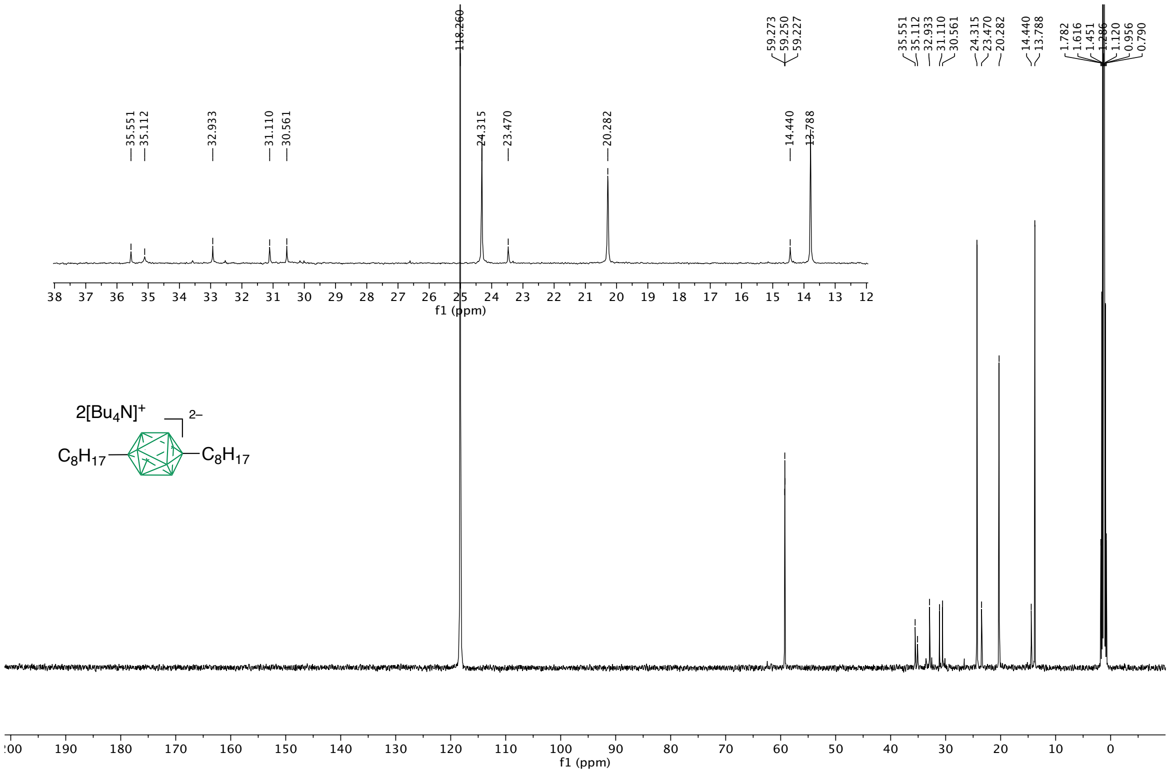

Figure S21. $^{13}C\{^{1}H\}$ NMR spectrum of [*closo*-$B_{10}H_8$-1,10-$(C_8H_{17})_2$][Bu_4N]$_2$ (**2b[Bu$_4$N]**, 126 MHz, CD_3CN).

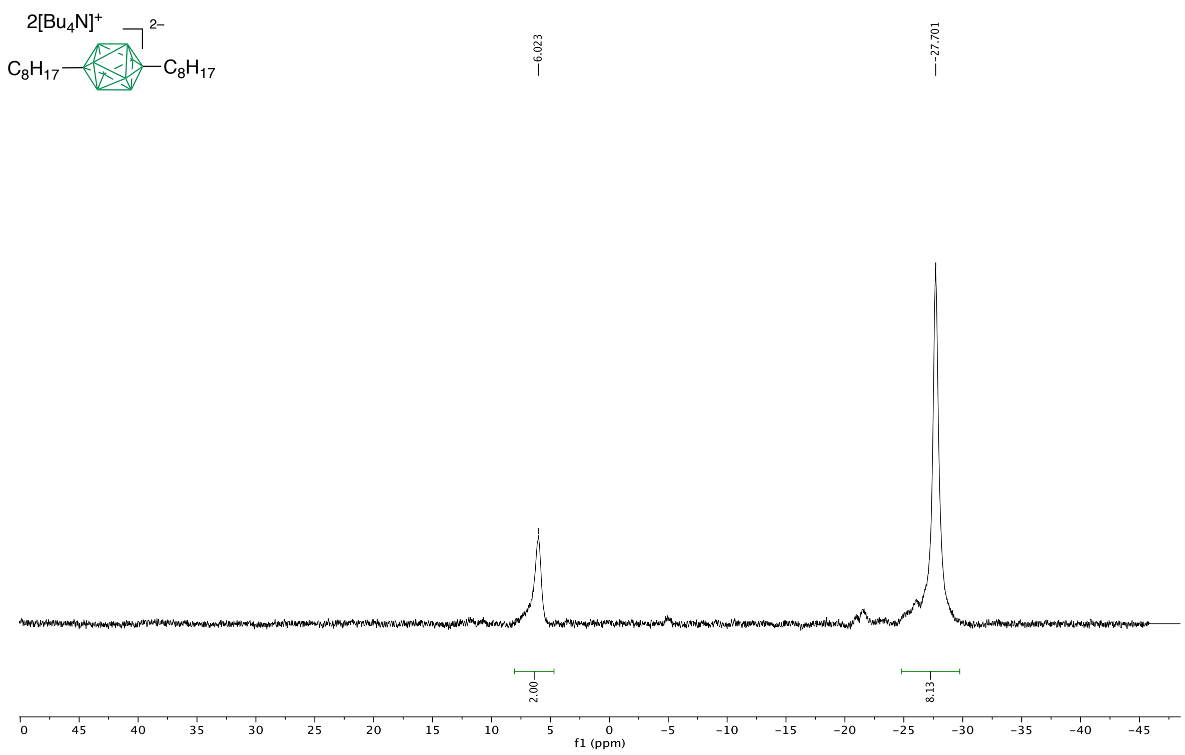

Figure S22. $^{11}B\{^{1}H\}$ NMR spectrum of [*closo*-$B_{10}H_8$-1,10-(C_8H_{17})$_2$][Bu_4N]$_2$ (**2b[Bu$_4$N]**, 160 MHz, CD_3CN).

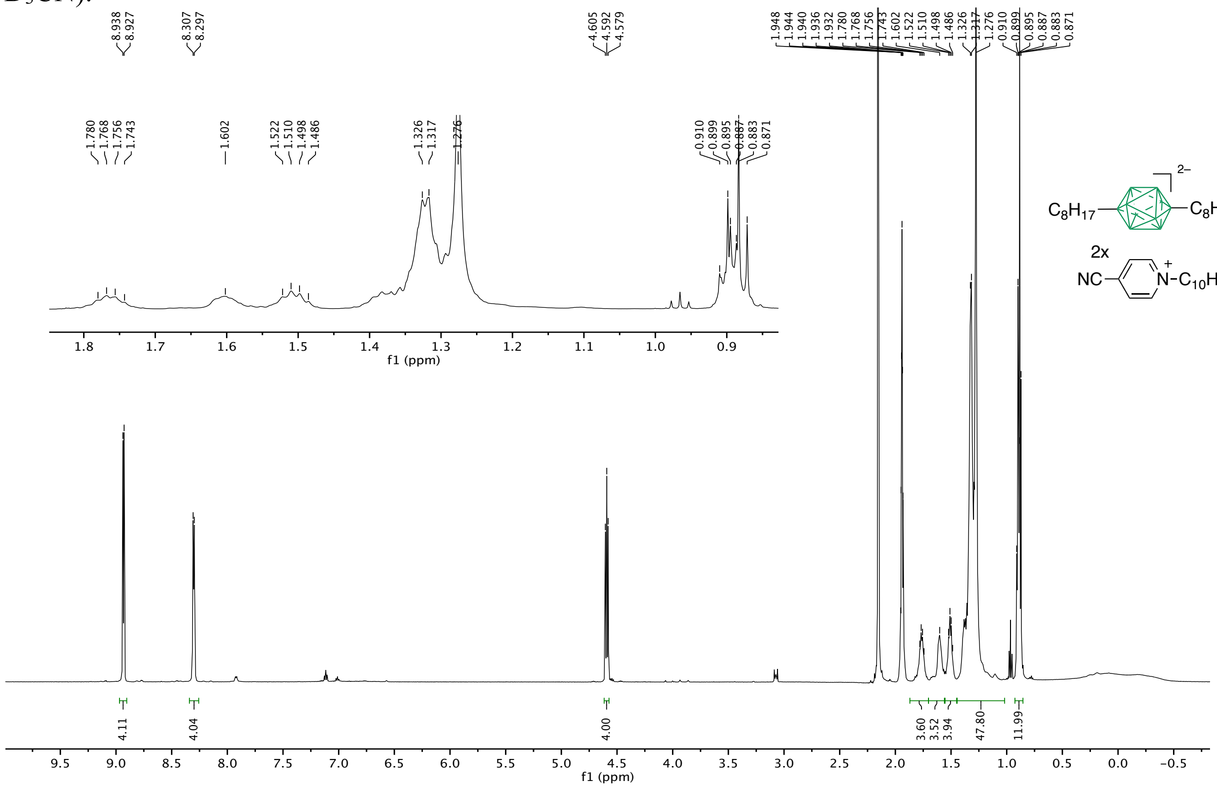

Figure S23. ^{1}H NMR spectrum of [*closo*-$B_{10}H_8$-1,10-(C_8H_{17})$_2$][*N*-decyl-4-cyanopyridinium]$_2$ (**2b[PyrCN]**, 600 MHz, CD_3CN).

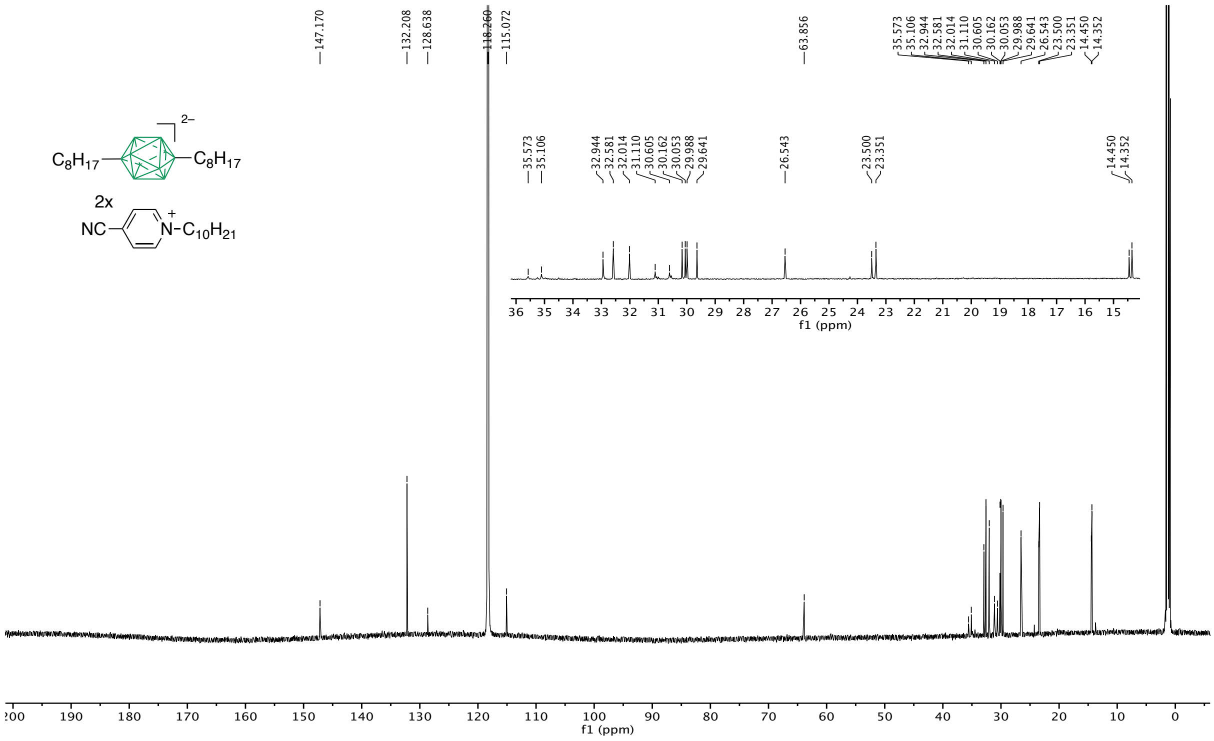

Figure S24. $^{13}C\{^{1}H\}$ NMR spectrum of [*closo*-$B_{10}H_8$-1,10-$(C_8H_{17})_2$][*N*-decyl-4-cyanopyridinium]$_2$ (**2b[PyrCN]**, 151 MHz, CD_3CN).

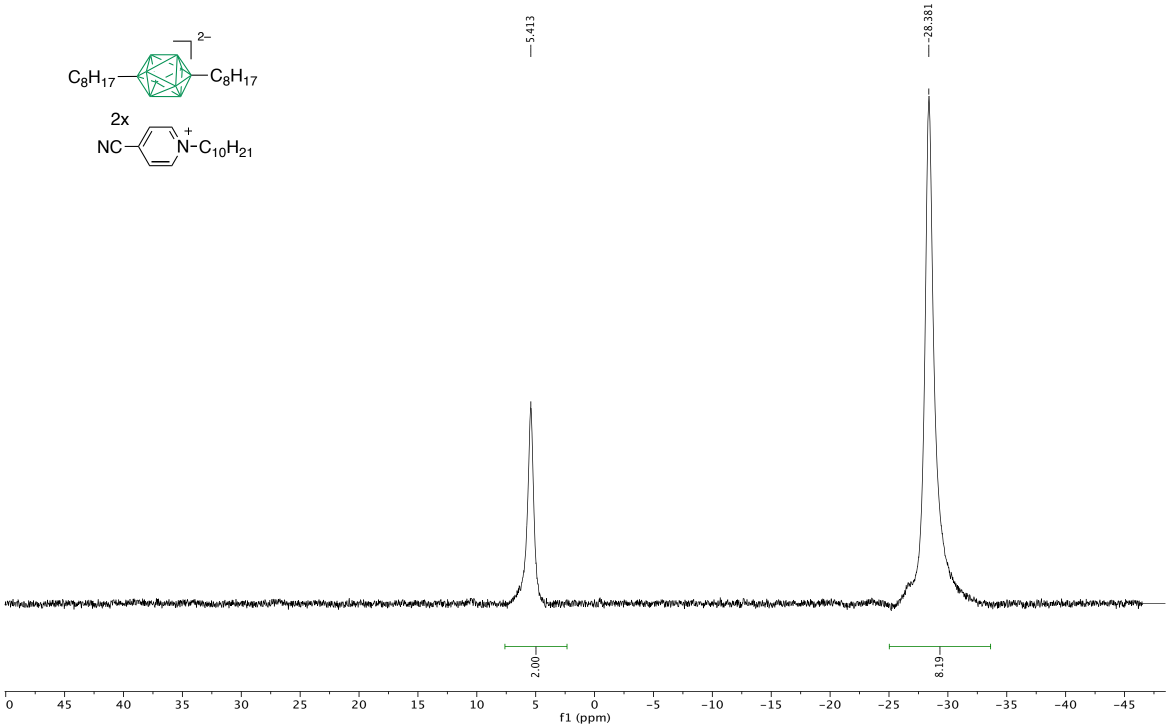

Figure S25. $^{11}B\{^{1}H\}$ NMR spectrum of [*closo*-$B_{10}H_8$-1,10-$(C_8H_{17})_2$][*N*-decyl-4-cyanopyridinium]$_2$ (**2b[PyrCN]**, 128 MHz, CD_3CN).

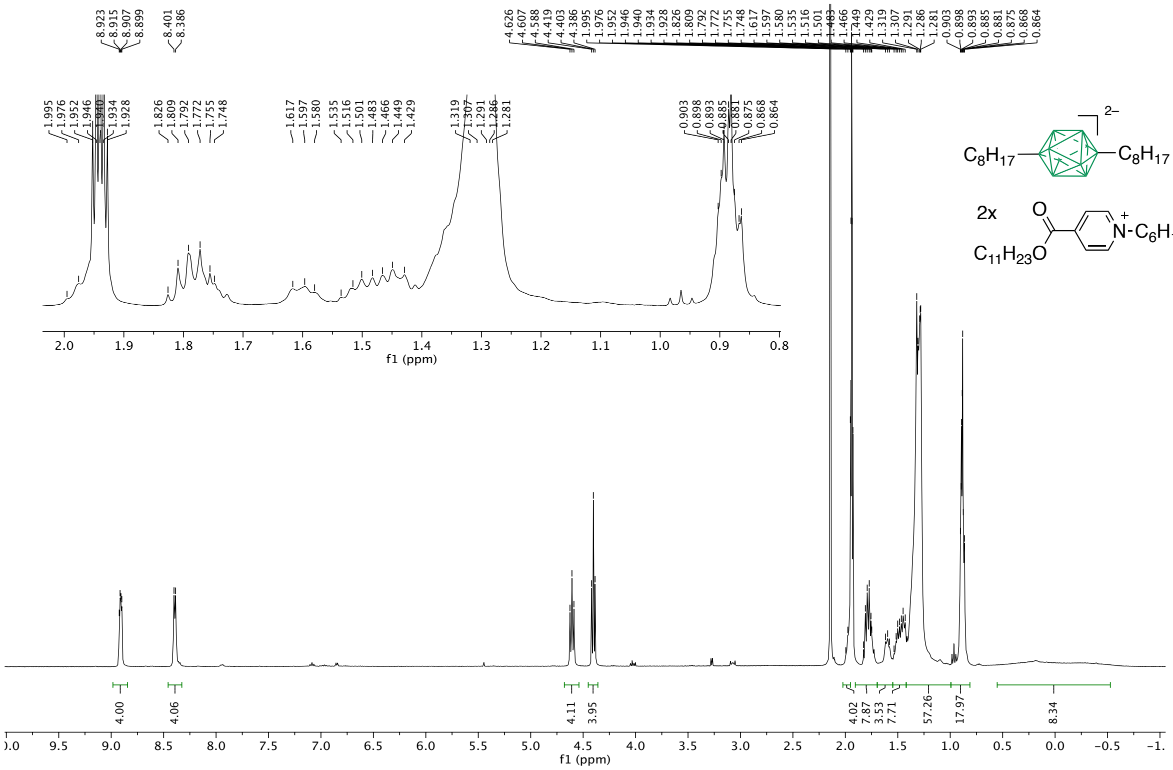

Figure S26. ^{1}H NMR spectrum of [*closo*-$B_{10}H_8$-1,10-$(C_8H_{17})_2$][*N*-hexyl-4-($C_{11}H_{23}$OCO)pyridinium]$_2$ (**2b[PyrCOOC$_{11}$]**, 400 MHz, CD_3CN).

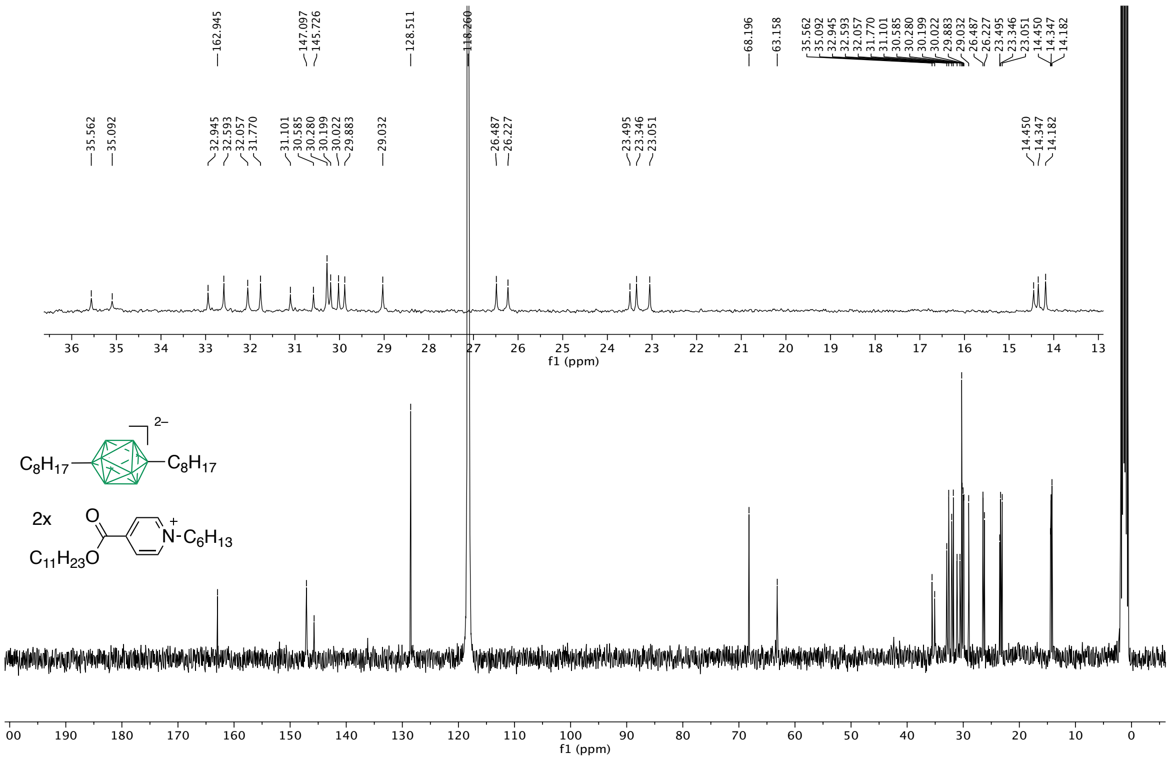

Figure S27. ^{13}C{^{1}H} NMR spectrum of [*closo*-$B_{10}H_8$-1,10-$(C_8H_{17})_2$][*N*-hexyl-4-($C_{11}H_{23}$OCO)pyridinium]$_2$ (**2b[PyrCOOC$_{11}$]**, 101 MHz, CD_3CN).

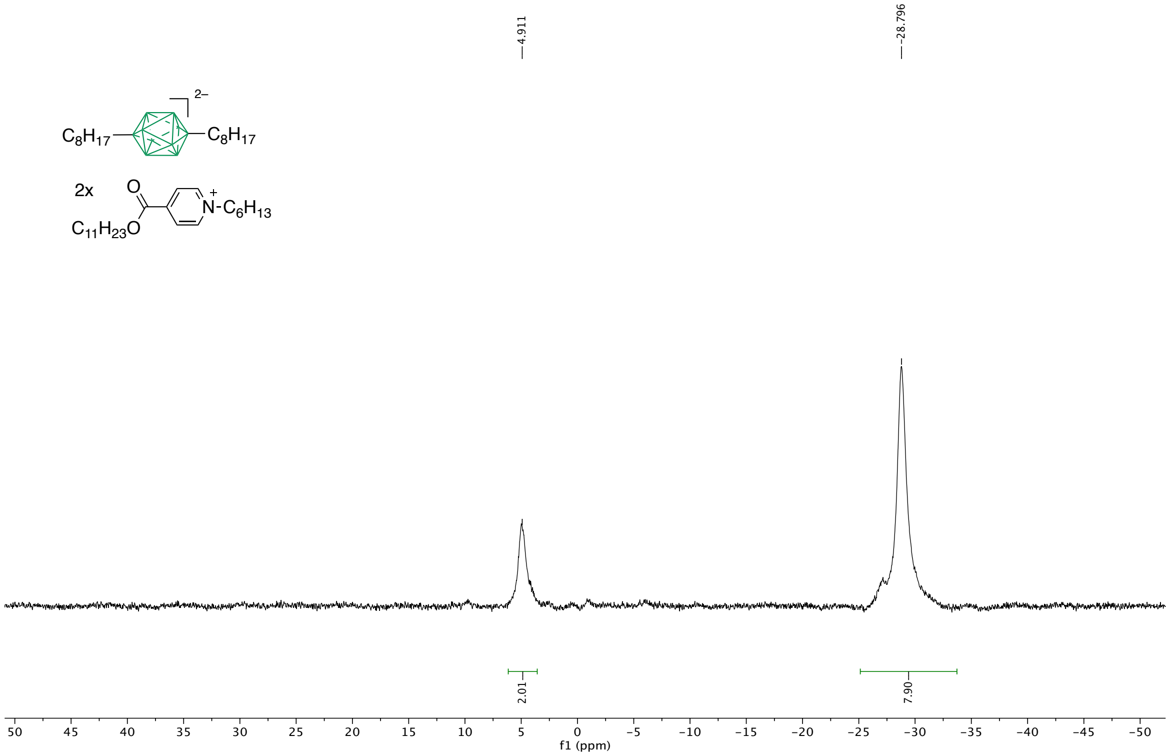

Figure S28. $^{11}B\{^1H\}$ NMR spectrum of [*closo*-$B_{10}H_8$-1,10-$(C_8H_{17})_2$][*N*-hexyl-4-($C_{11}H_{23}OCO$)pyridinium]$_2$ (**2b[PyrCOOC$_{11}$]**, 128 MHz, CD_3CN).

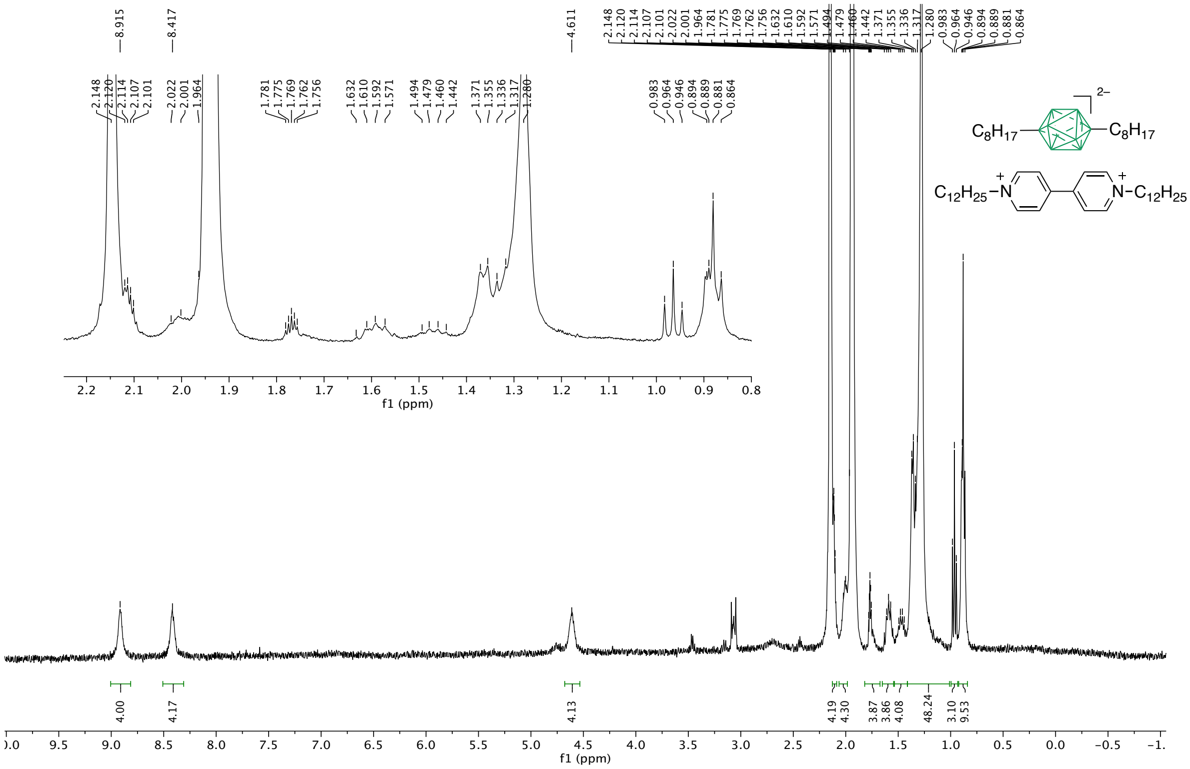

Figure S29. ^1H NMR spectrum of [*closo*-$B_{10}H_8$-1,10-$(C_8H_{17})_2$][*N,N'*-didodecyl-4,4′-bipyridinium] (**2b[Q12]**, 400 MHz, CD_3CN).

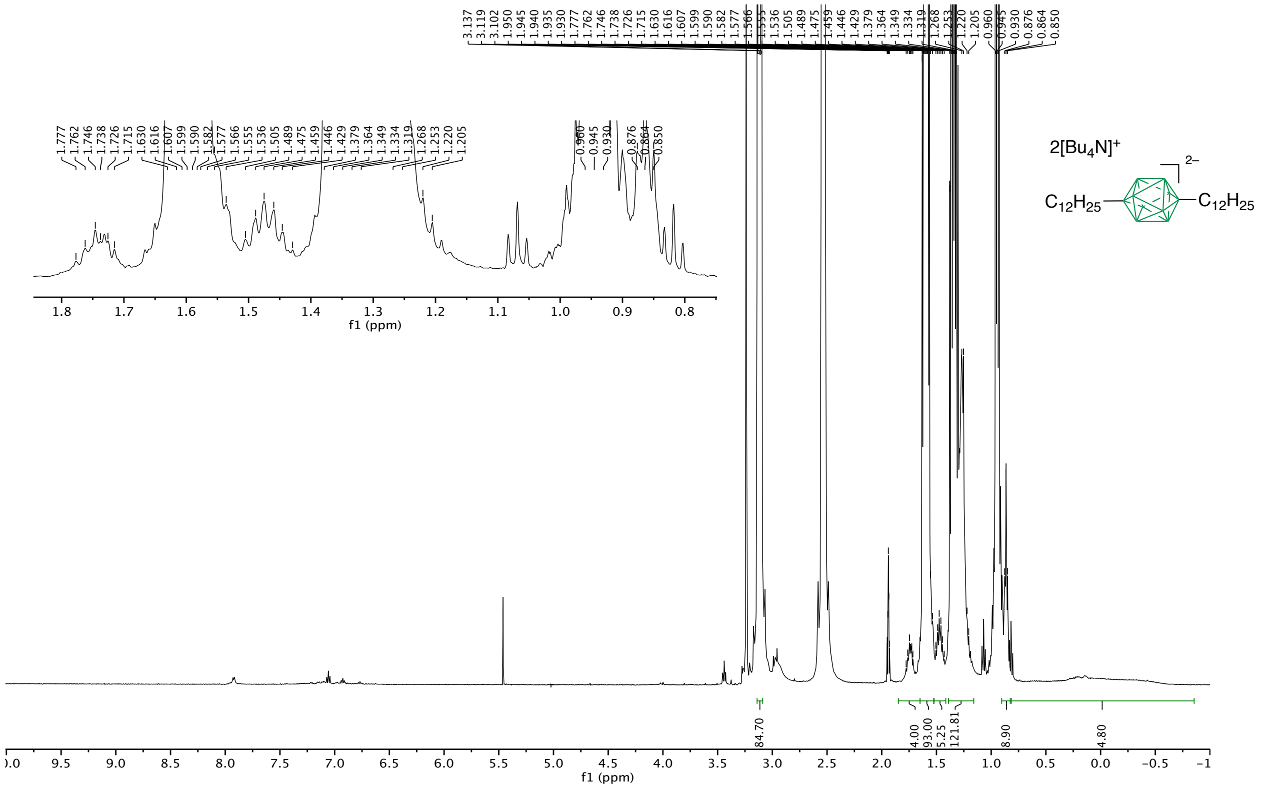

Figure S30. ^{1}H NMR spectrum of [*closo*-$B_{10}H_8$-1,10-($C_{12}H_{25}$)$_2$][Bu_4N]$_2$ (**2c[Bu$_4$N]**, 500 MHz, CD_3CN).

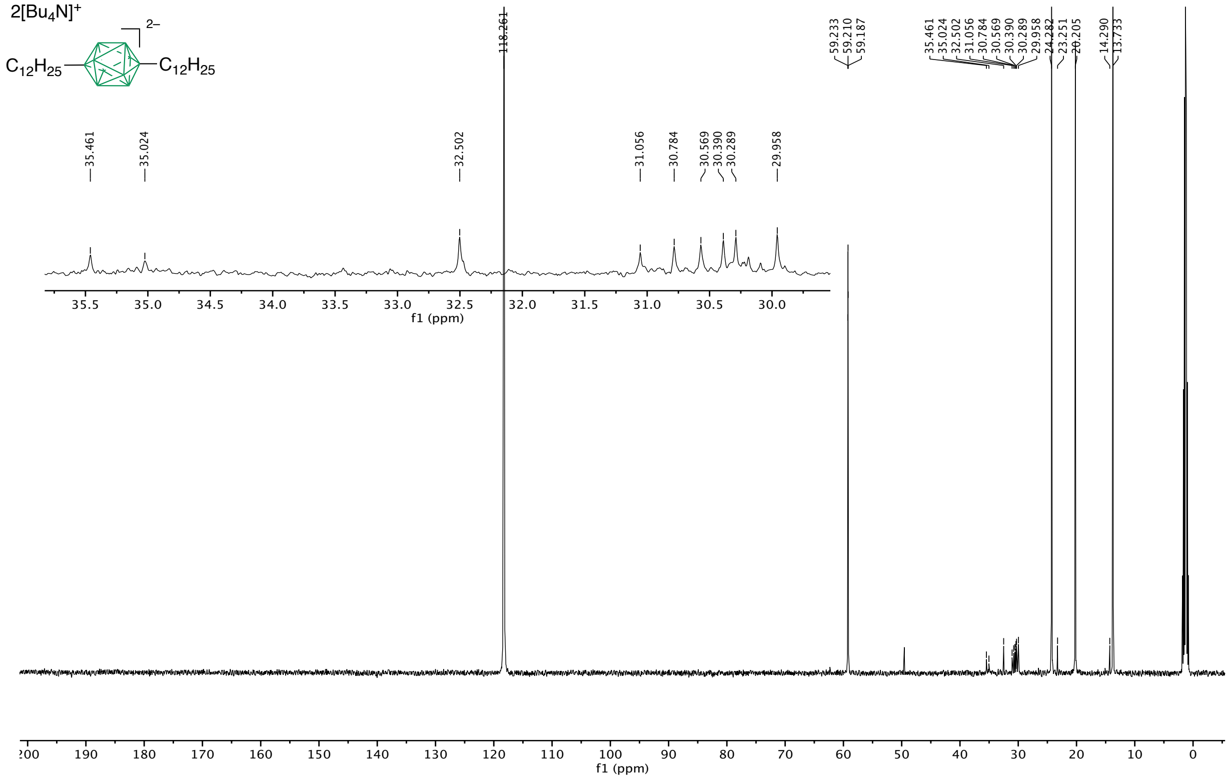

Figure S31. ^{13}C{^{1}H} NMR spectrum of [*closo*-$B_{10}H_8$-1,10-($C_{12}H_{25}$)$_2$][Bu_4N]$_2$ (**2c[Bu$_4$N]**, 126 MHz, CD_3CN).

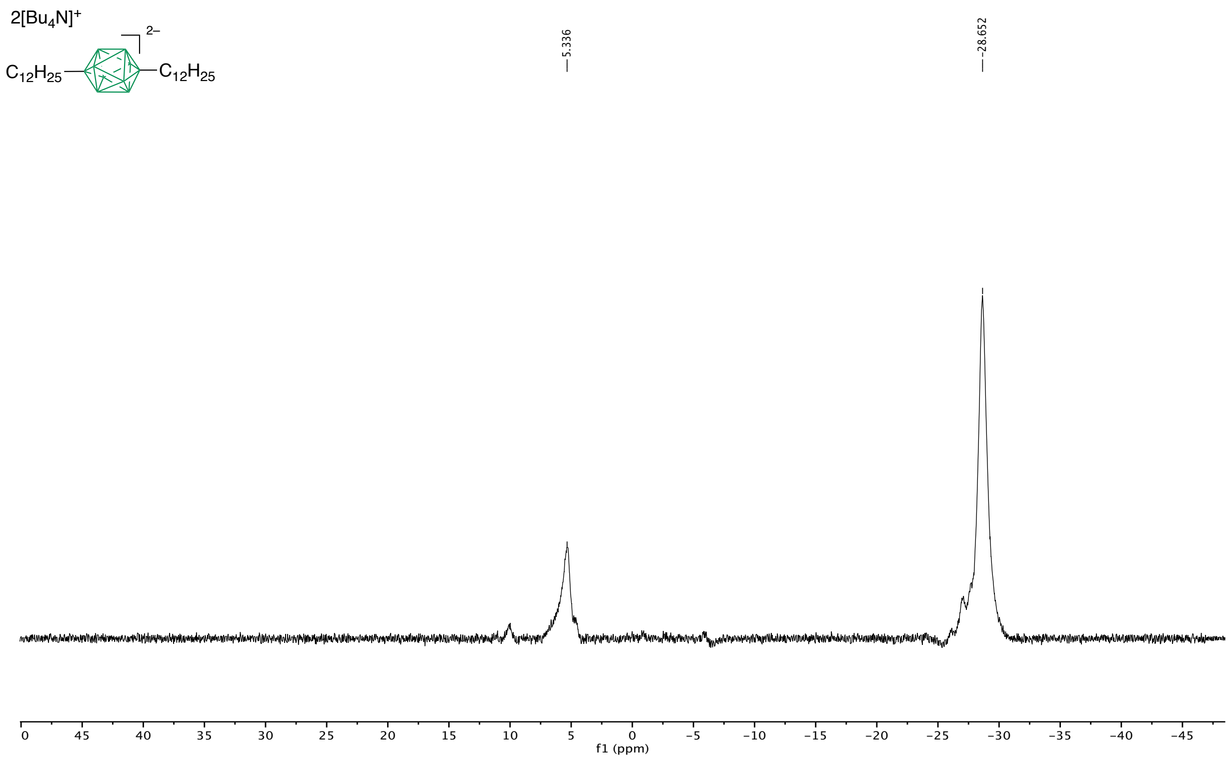

Figure S32. $^{11}B\{^1H\}$ NMR spectrum of [*closo*-$B_{10}H_8$-1,10-$(C_{12}H_{25})_2$][Bu_4N]$_2$ (**2c[Bu₄N]**, 160 MHz, CD_3CN).

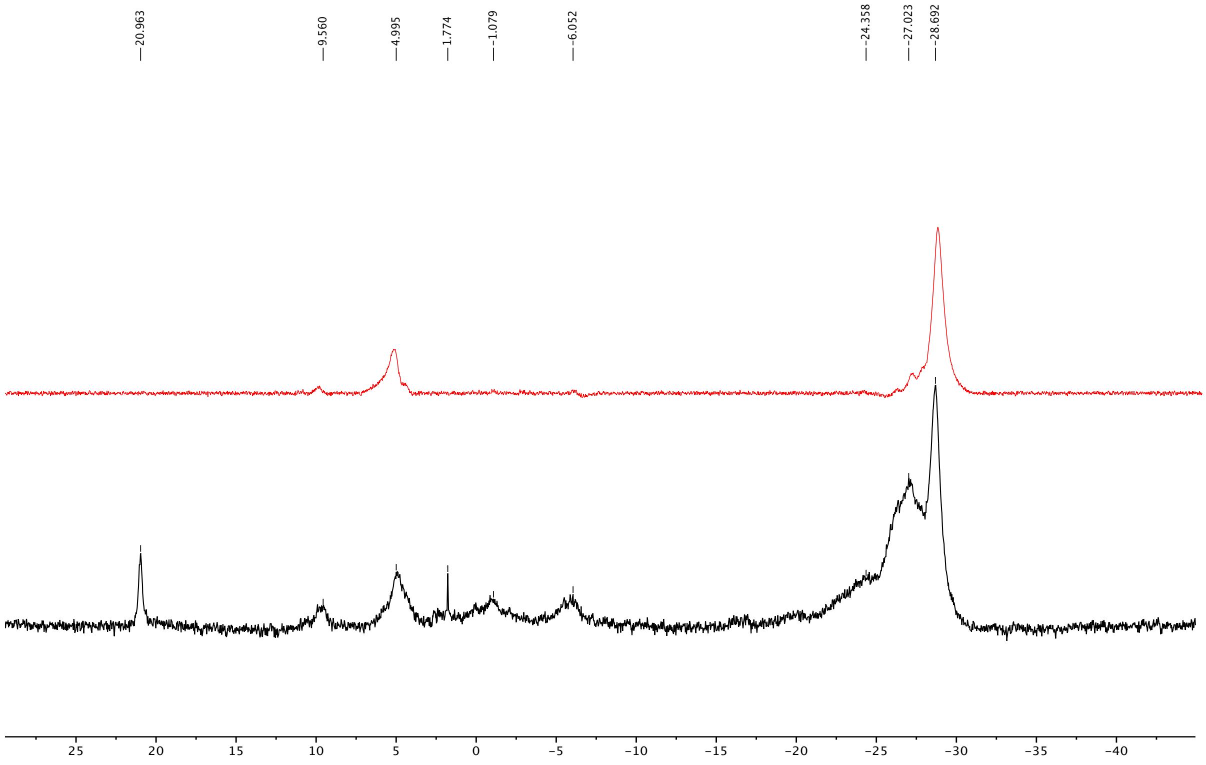

Figure S33. $^{11}B\{^1H\}$ NMR spectra of freshly purified (top, red) and stored for 4 weeks (black, bottom) [*closo*-$B_{10}H_8$-1,10-$(C_{12}H_{25})_2$][Bu_4N]$_2$ (**2c[Bu₄N]**, 160 MHz, CD_3CN).

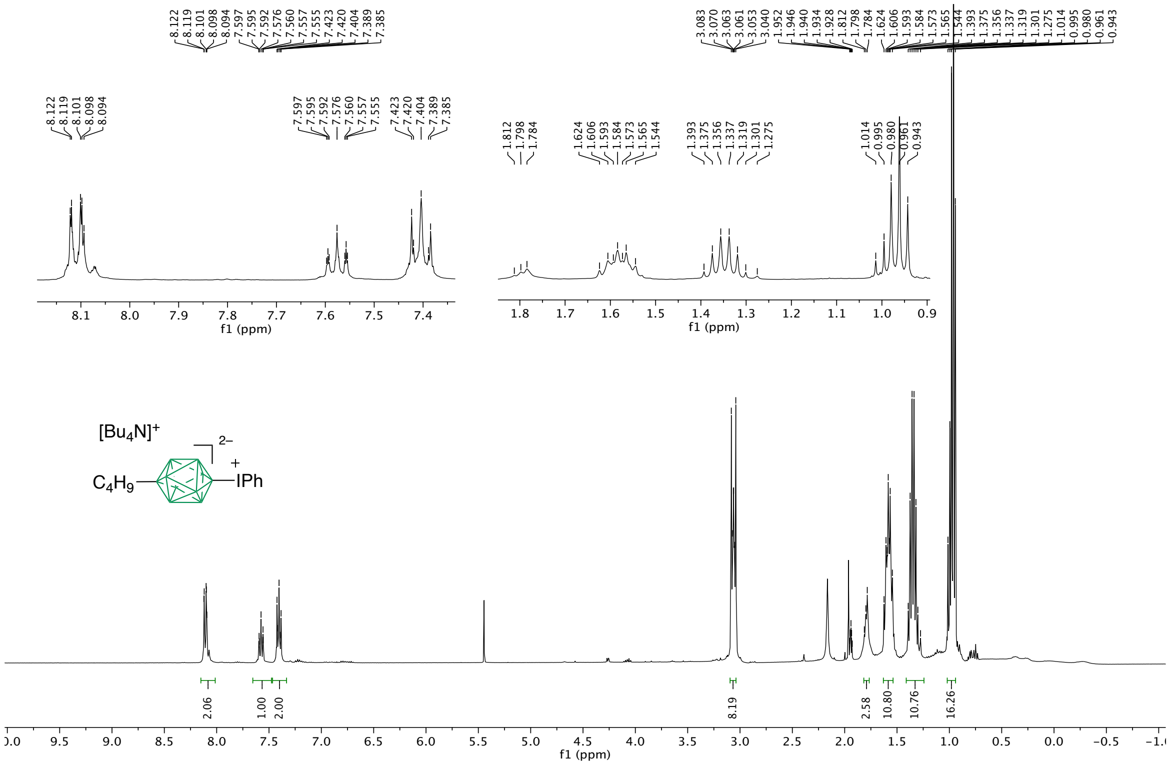

Figure S34. ^{1}H NMR spectrum of [*closo*-$B_{10}H_8$-1-IPh-10-C_4H_9][Bu_4N] (**7a[Bu$_4$N]**, 400 MHz, CD_3CN).

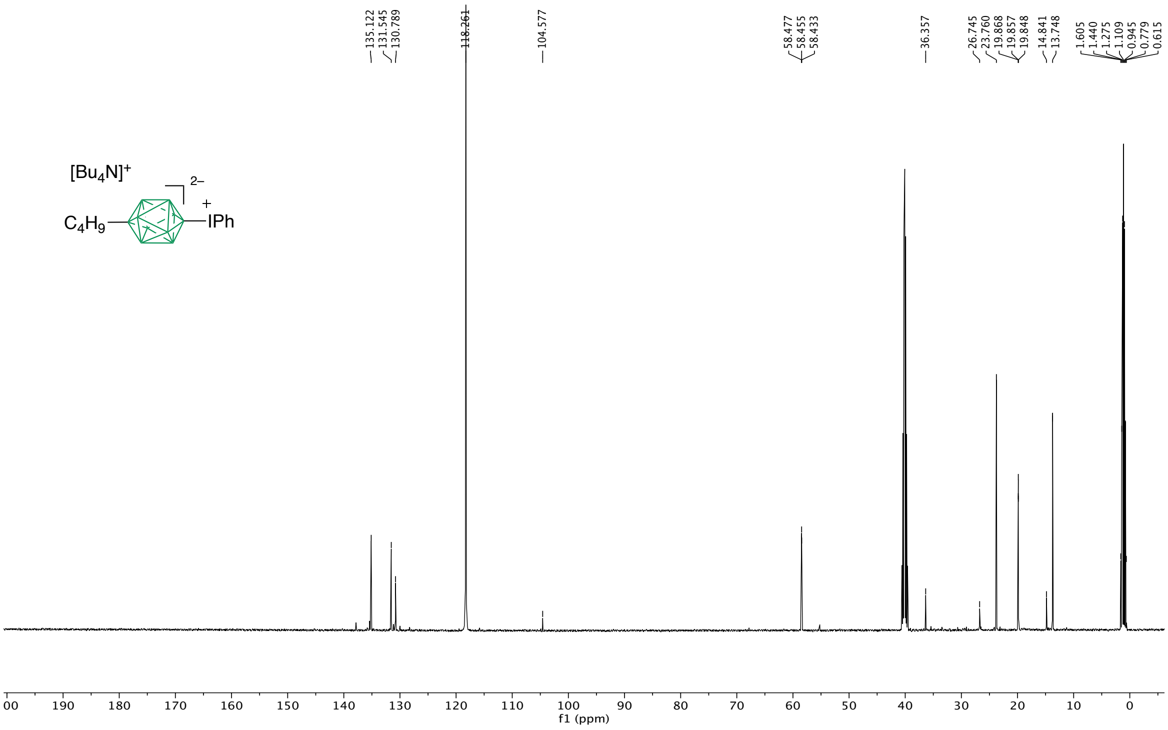

Figure S35. $^{13}C\{^{1}H\}$ NMR spectrum of [*closo*-$B_{10}H_8$-1-IPh-10-C_4H_9][Bu_4N] (**7a[Bu$_4$N]**, 126 MHz, CD_3CN).

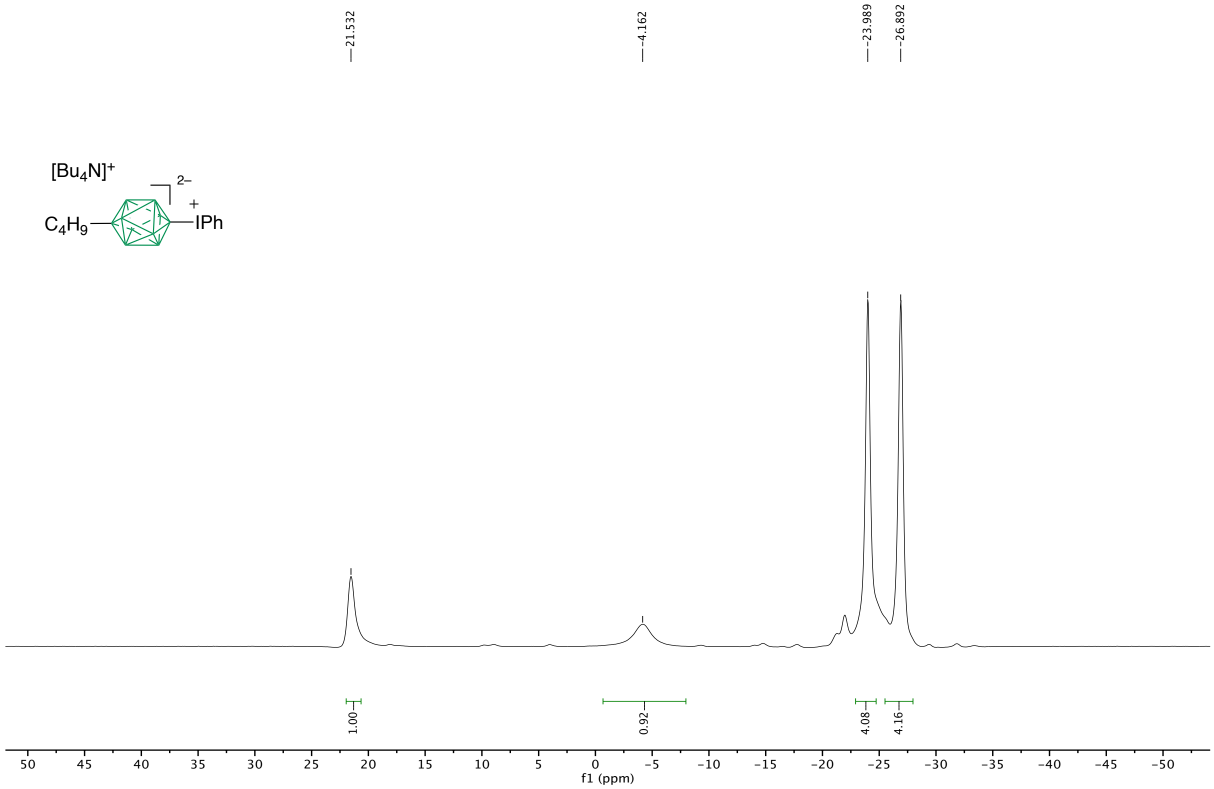

Figure S36. $^{11}B\{^1H\}$ NMR spectrum of [*closo*-$B_{10}H_8$-1-IPh-10-C_4H_9][Bu_4N] (**7a[Bu₄N]**, 161 MHz, CD_3CN).

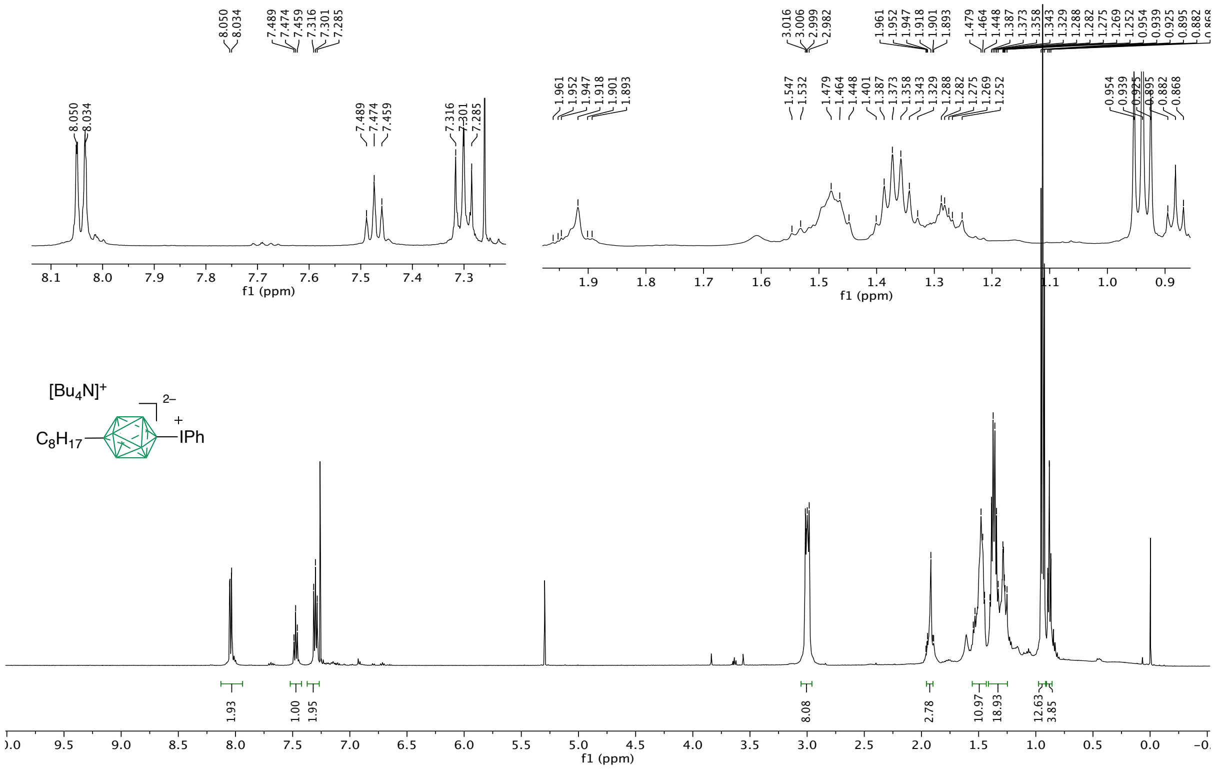

Figure S37. ^1H NMR spectrum of [*closo*-$B_{10}H_8$-1-IPh-10-C_8H_{17}][Bu_4N] (**7b[Bu₄N]**, 500 MHz, $CDCl_3$).

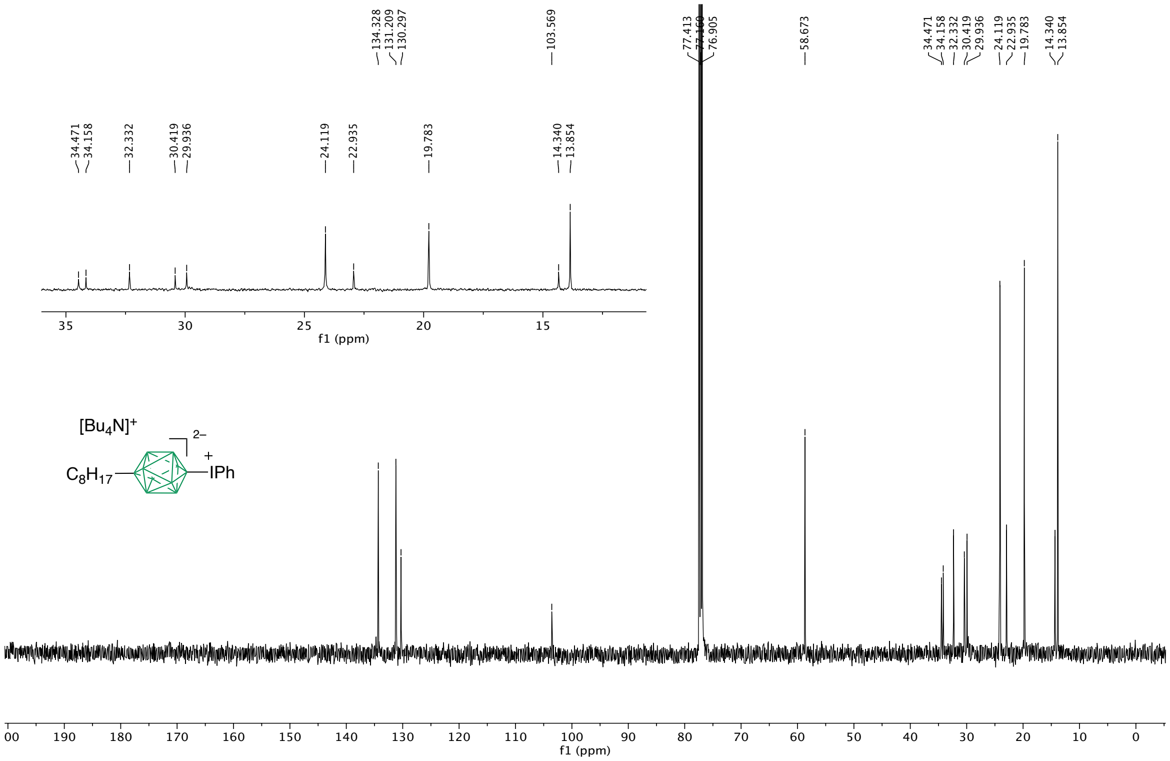

Figure S38. $^{13}C\{^{1}H\}$ NMR spectrum of [*closo*-$B_{10}H_8$-1-IPh-10-C_8H_{17}][Bu_4N] (**7b[Bu₄N]**, 126 MHz, $CDCl_3$).

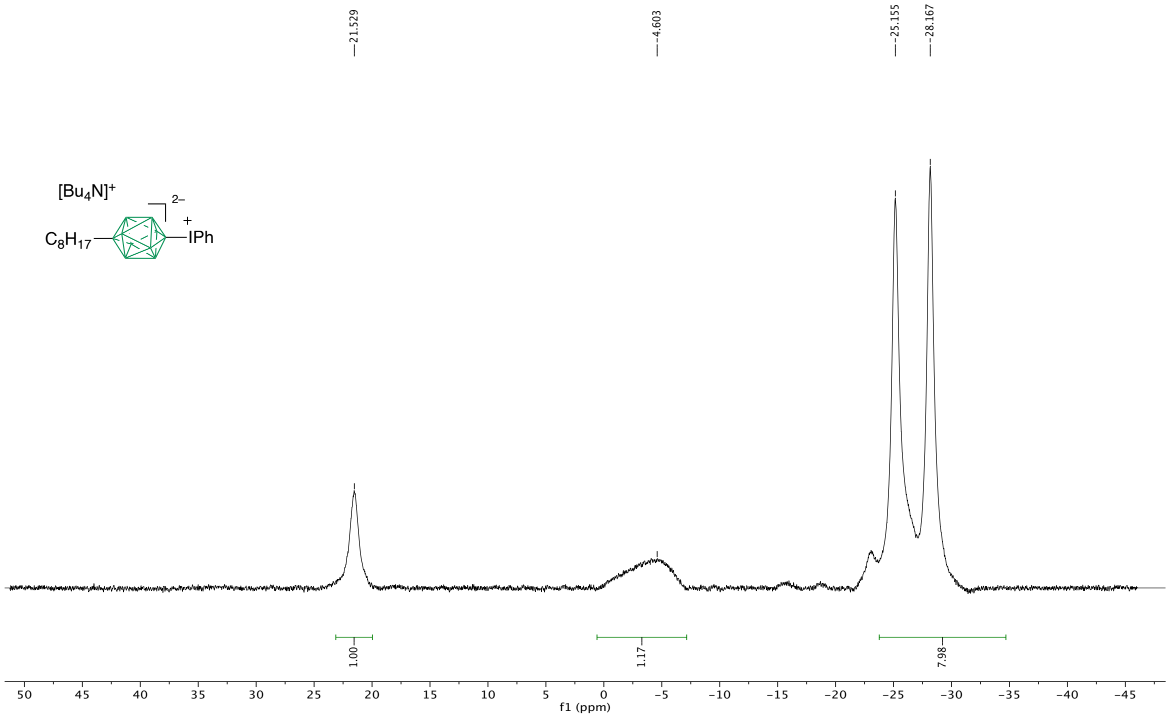

Figure S39. $^{11}B\{^{1}H\}$ NMR spectrum of [*closo*-$B_{10}H_8$-1-IPh-10-C_8H_{17}][Bu_4N] (**7b[Bu₄N]**, 161 MHz, $CDCl_3$).

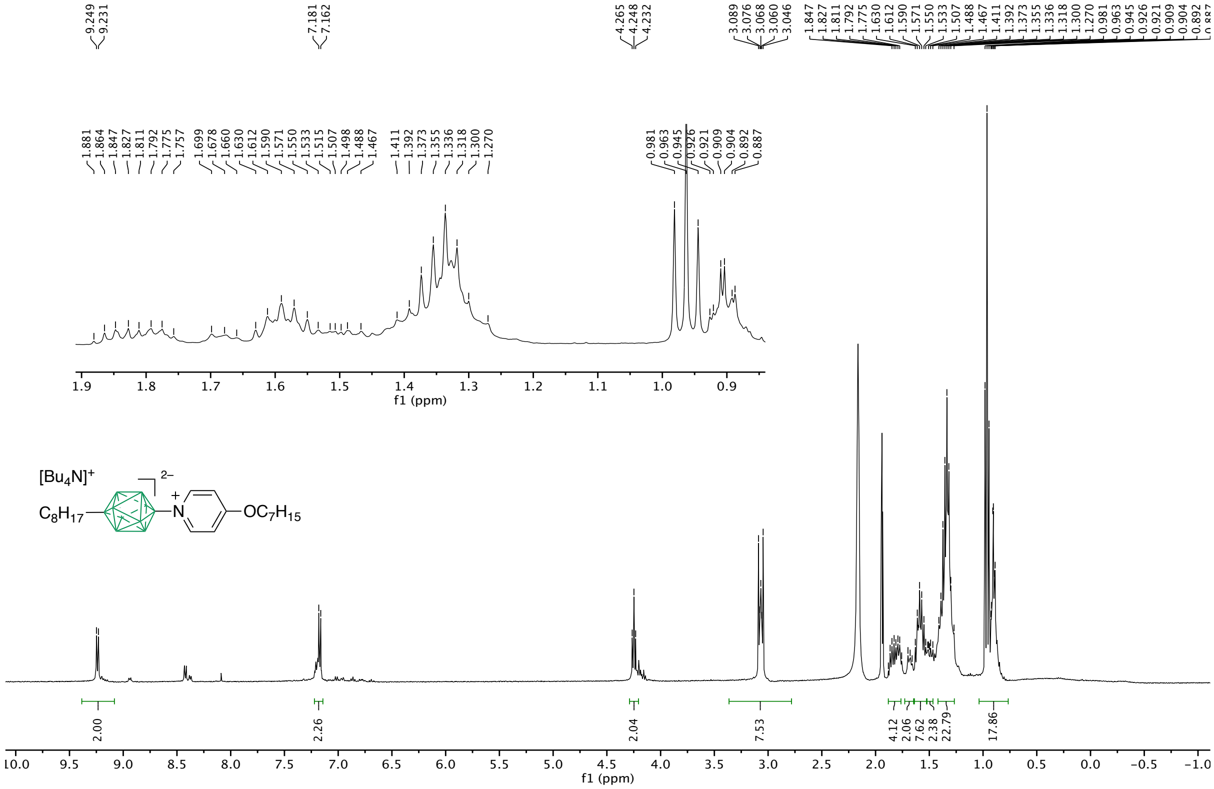

Figure S40. ^{1}H NMR spectrum of [*closo*-$B_{10}H_8$-1-(NC_5H_4-4-OC_7H_{15})-10-C_8H_{17}][Bu_4N] (**8b[Bu₄N]**, 400 MHz, CD_3CN).

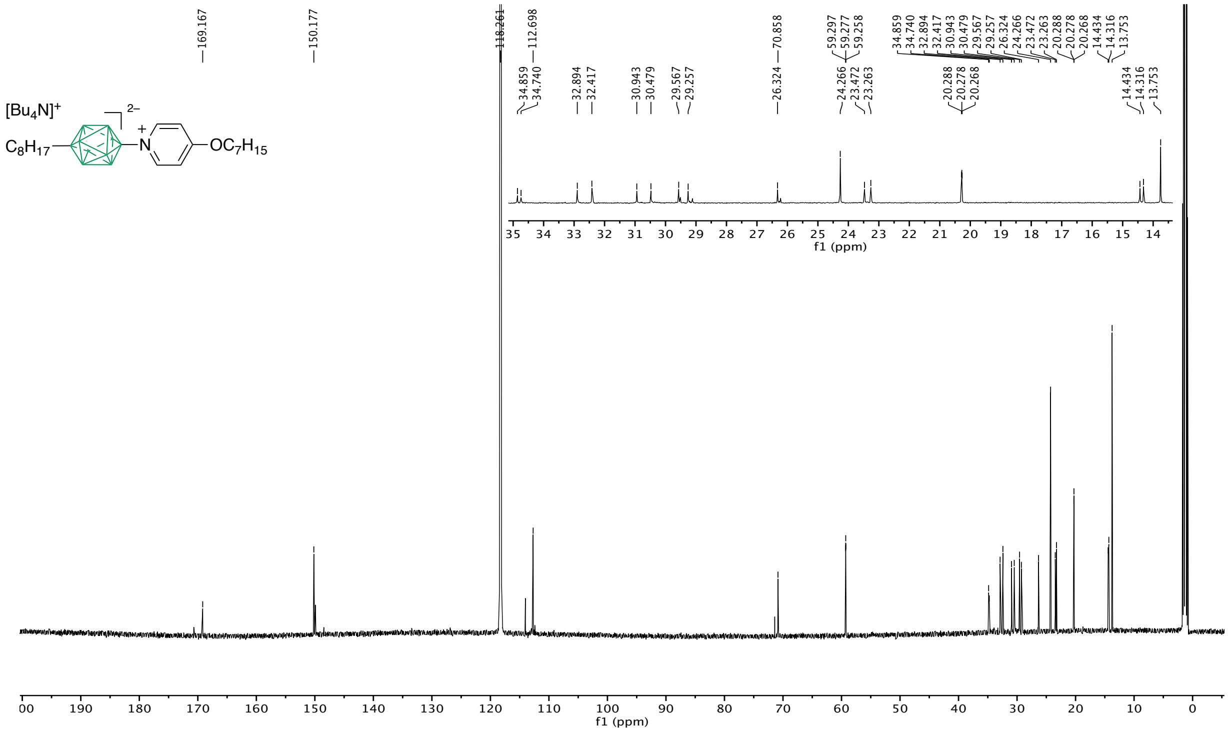

Figure S41.$^{13}C\{^1H\}$ NMR spectrum of [*closo*-$B_{10}H_8$-1-(NC_5H_4-4-OC_7H_{15})-10-C_8H_{17}][Bu_4N] (**8b[Bu_4N]**, 151 MHz, CD_3CN).

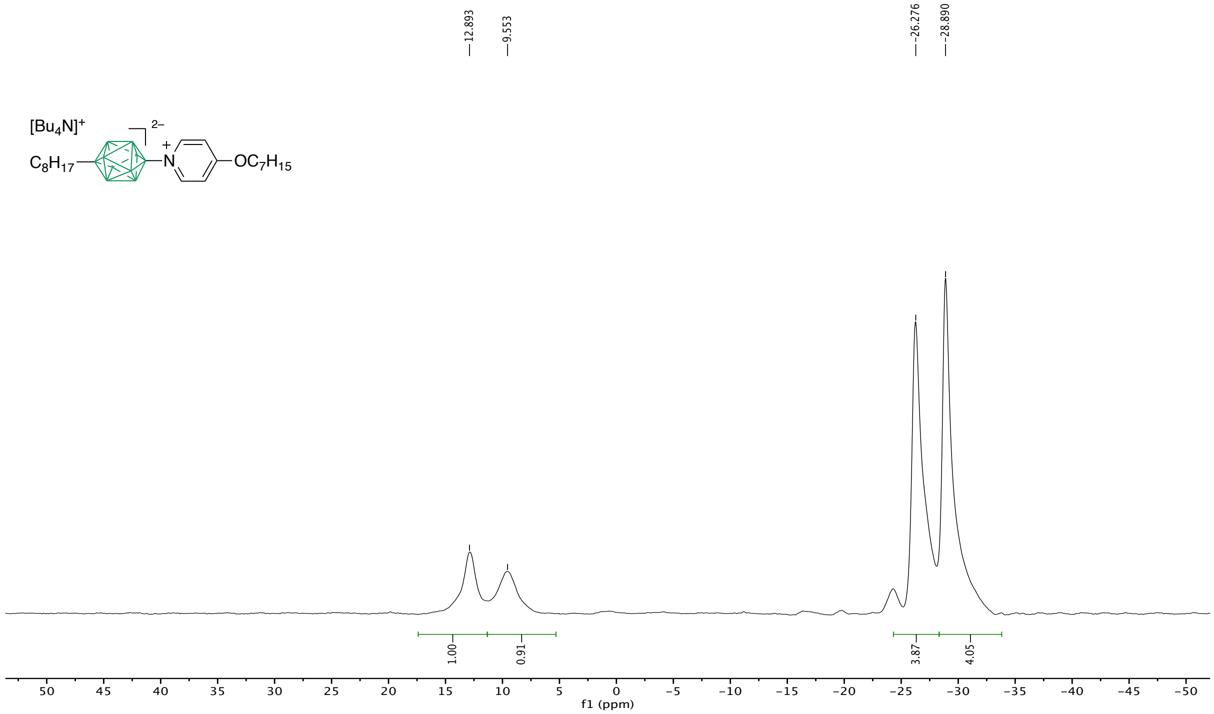

Figure S42. $^{11}B\{^{1}H\}$ NMR spectrum of [*closo*-$B_{10}H_8$-1-(NC_5H_4-4-OC_7H_{15})-10-C_8H_{17}][Bu_4N] (**8b[Bu$_4$N]**, 128 MHz, CD_3CN).

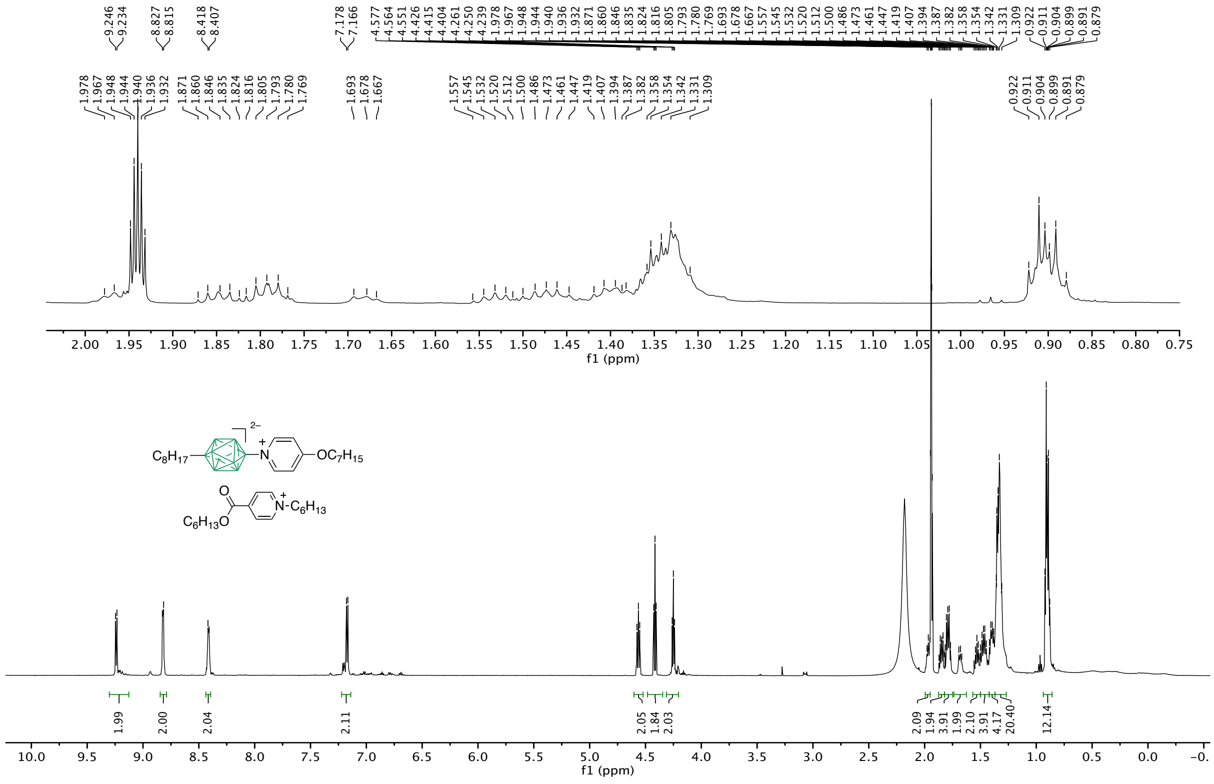

Figure S43. ^{1}H NMR spectrum of [*closo*-$B_{10}H_8$-1-(NC_5H_4-4-OC_7H_{15})-10-C_8H_{17}][*N*-hexyl-4-($C_6H_{13}OCO$)pyridinium] (**8b[PyrCOOC$_6$]**, 600 MHz, CD_3CN).

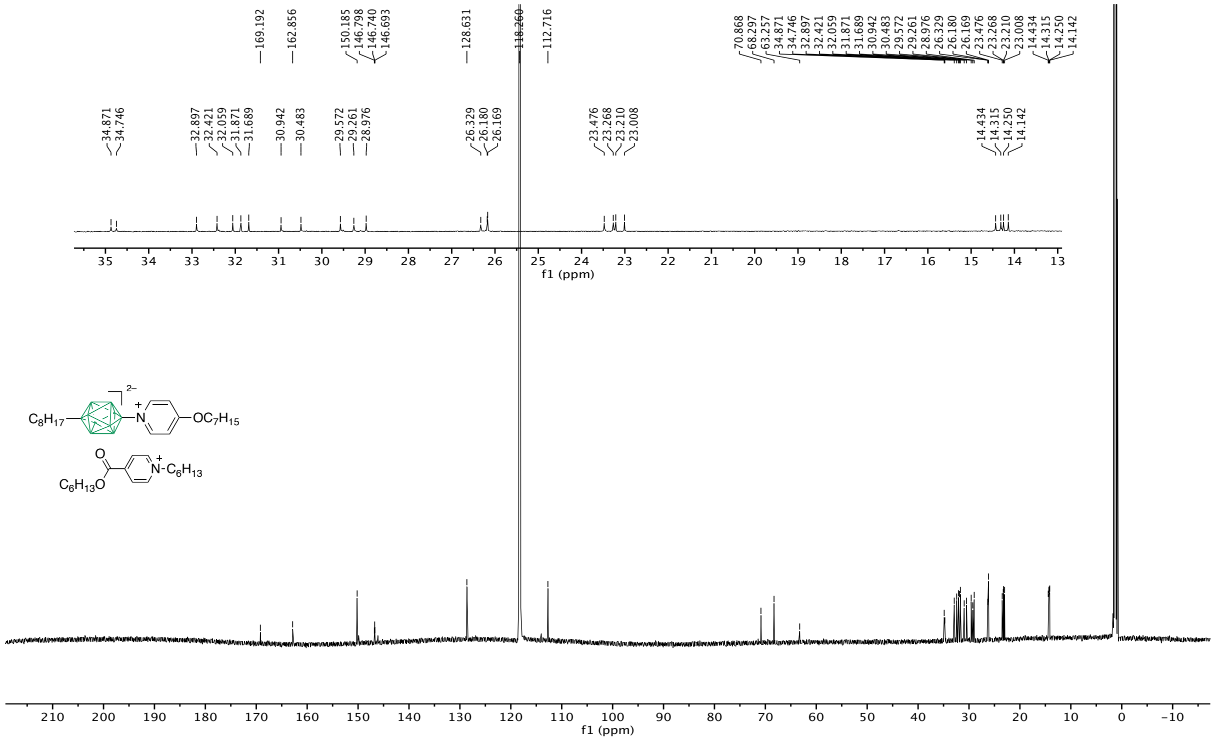

Figure S44. $^{13}C\{^{1}H\}$ NMR spectrum of [*closo*-$B_{10}H_8$-1-(NC_5H_4-4-OC_7H_{15})-10-C_8H_{17}][*N*-hexyl-4-($C_6H_{13}OCO$)pyridinium] (**8b[PyrCOOC$_6$]**, 151 MHz, CD_3CN).

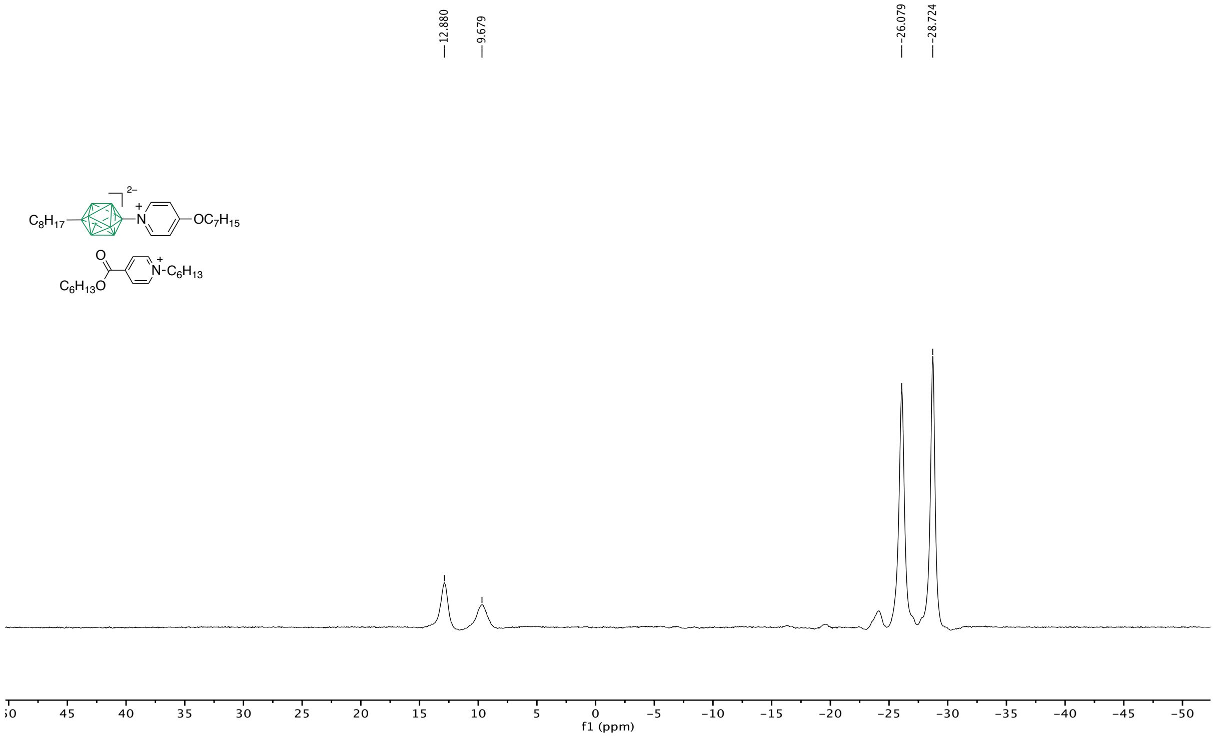

Figure S45. $^{11}B\{^{1}H\}$ NMR spectrum of [*closo*-$B_{10}H_8$-1-(NC_5H_4-4-OC_7H_{15})-10-C_8H_{17}][*N*-hexyl-4-($C_6H_{13}OCO$)pyridinium] (**8b[PyrCOOC$_6$]**, 193 MHz, CD_3CN).

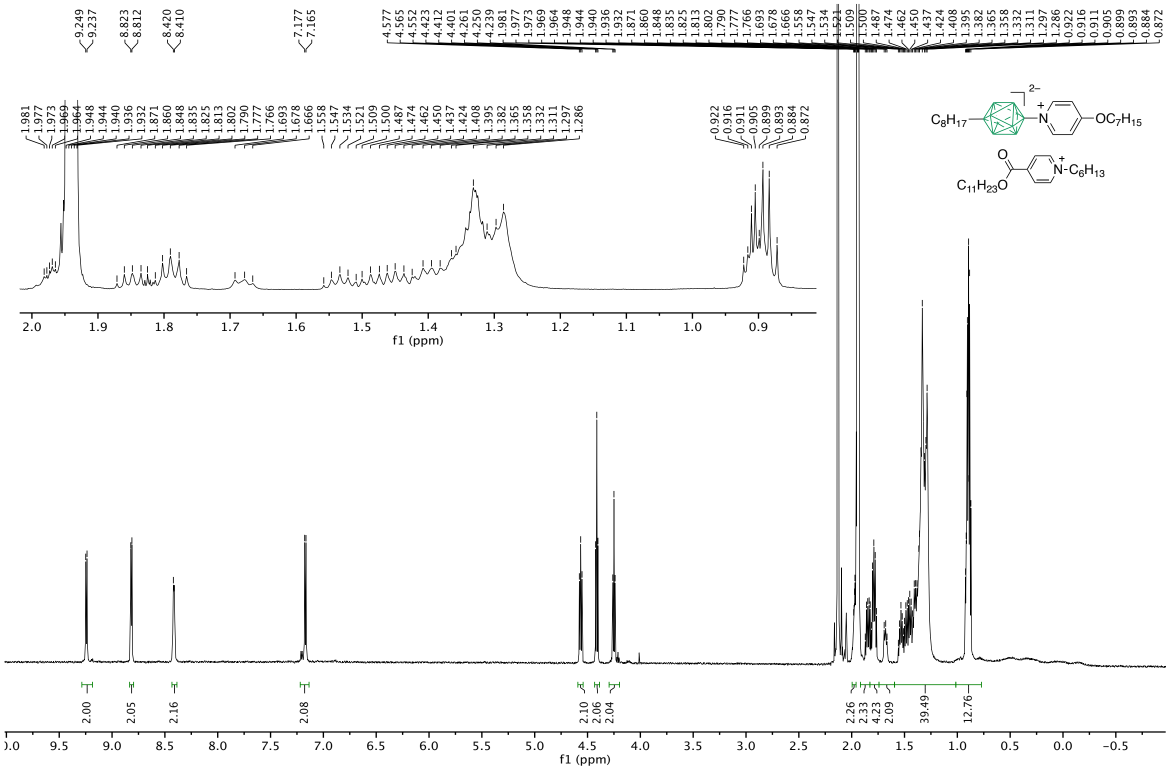

Figure S46. ^{1}H NMR spectrum of [*closo*-$B_{10}H_8$-1-(NC_5H_4-4-OC_7H_{15})-10-C_8H_{17}][*N*-hexyl-4-($C_{11}H_{23}OCO$)pyridinium] (**8b[PyrCOOC₁₁]**, 600 MHz, CD_3CN).

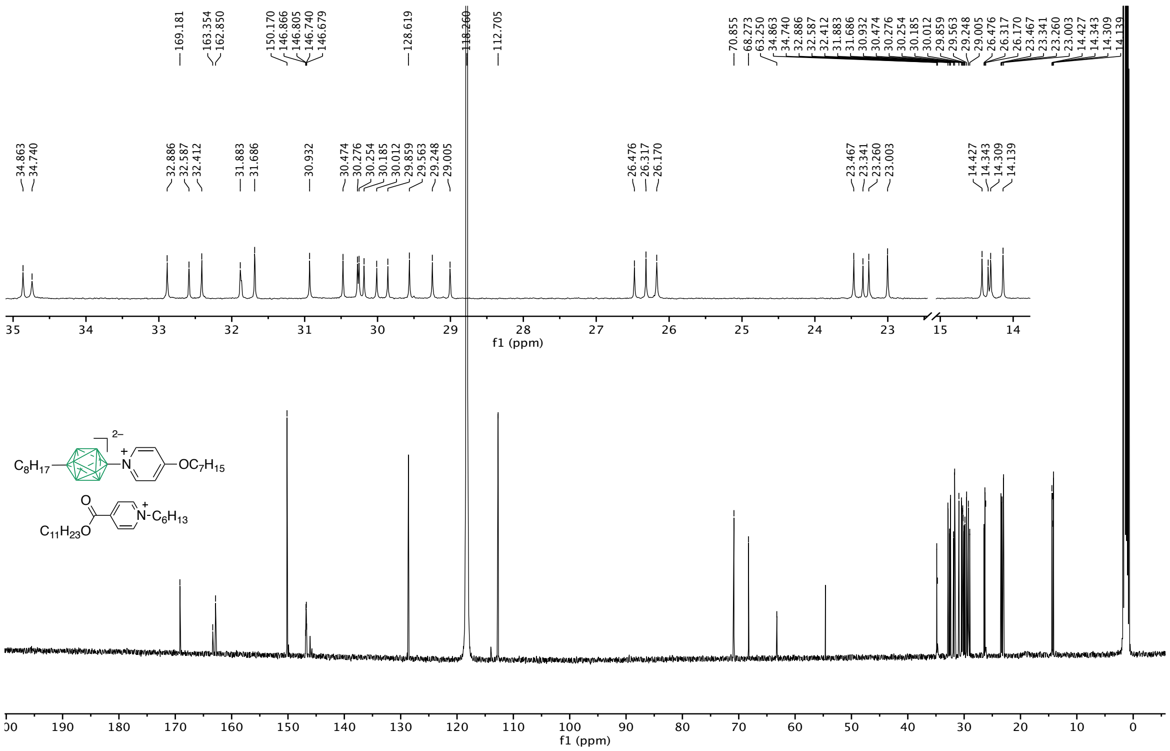

Figure S47. $^{13}C\{^1H\}$ NMR spectrum of [*closo*-$B_{10}H_8$-1-(NC_5H_4-4-OC_7H_{15})-10-C_8H_{17}][*N*-hexyl-4-($C_{11}H_{23}OCO$)pyridinium] (**8b[PyrCOOC₁₁]**, 151 MHz, CD_3CN).

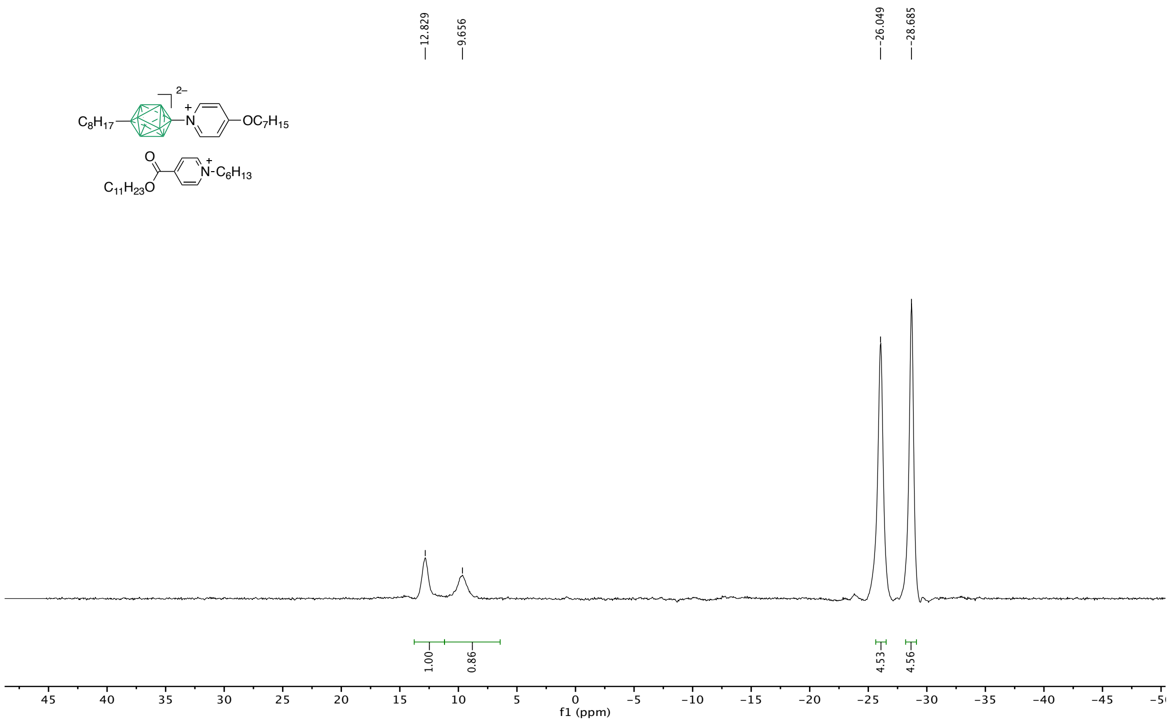

Figure S48. $^{11}B\{^1H\}$ NMR spectrum of [*closo*-$B_{10}H_8$-1-(NC_5H_4-4-OC_7H_{15})-10-C_8H_{17}][*N*-hexyl-4-($C_{11}H_{23}OCO$)pyridinium] (**8b[PyrCOOC₁₁]**, 193 MHz, CD_3CN).

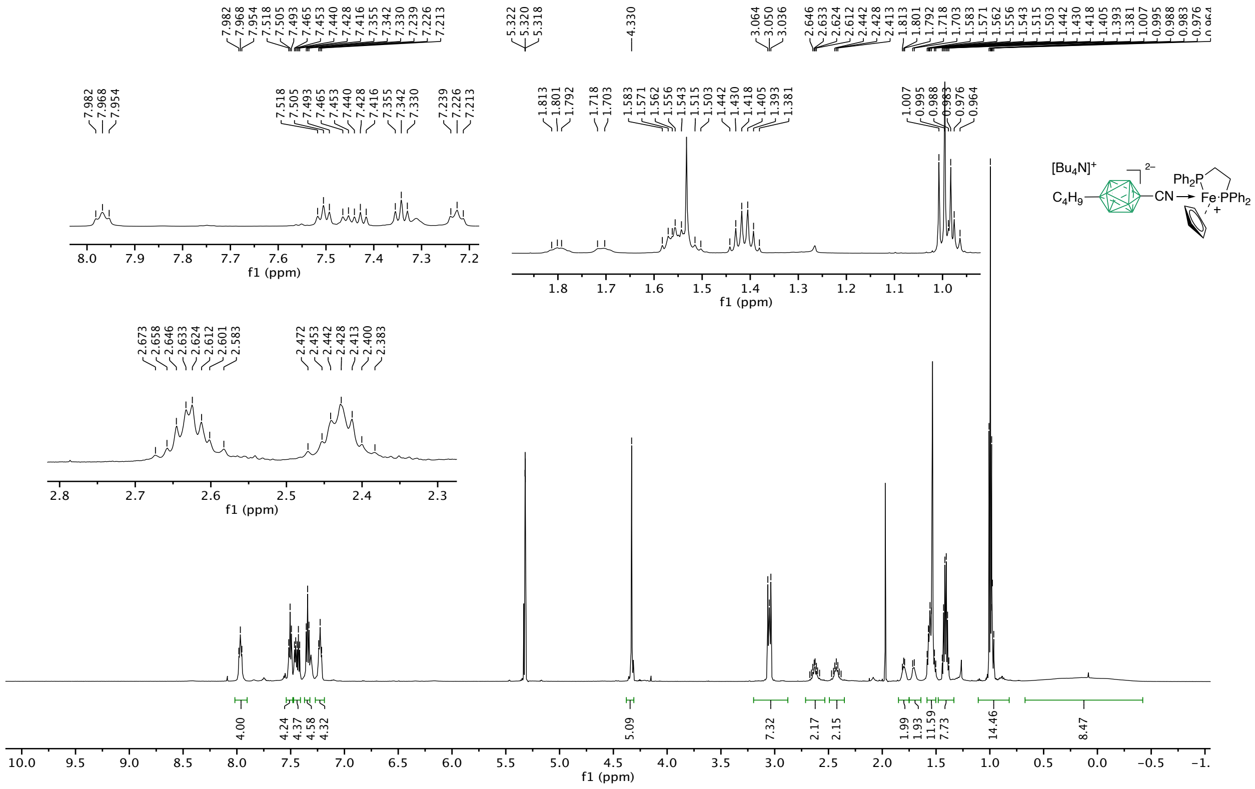

Figure S49. ^1H NMR spectrum of [{(η5-Cp)(dppe)Fe}-*closo*-$B_{10}H_8$-1-CN-10-C_4H_9][Bu_4N] (**9a[Bu₄N]**, 600 MHz, CD_2Cl_2).

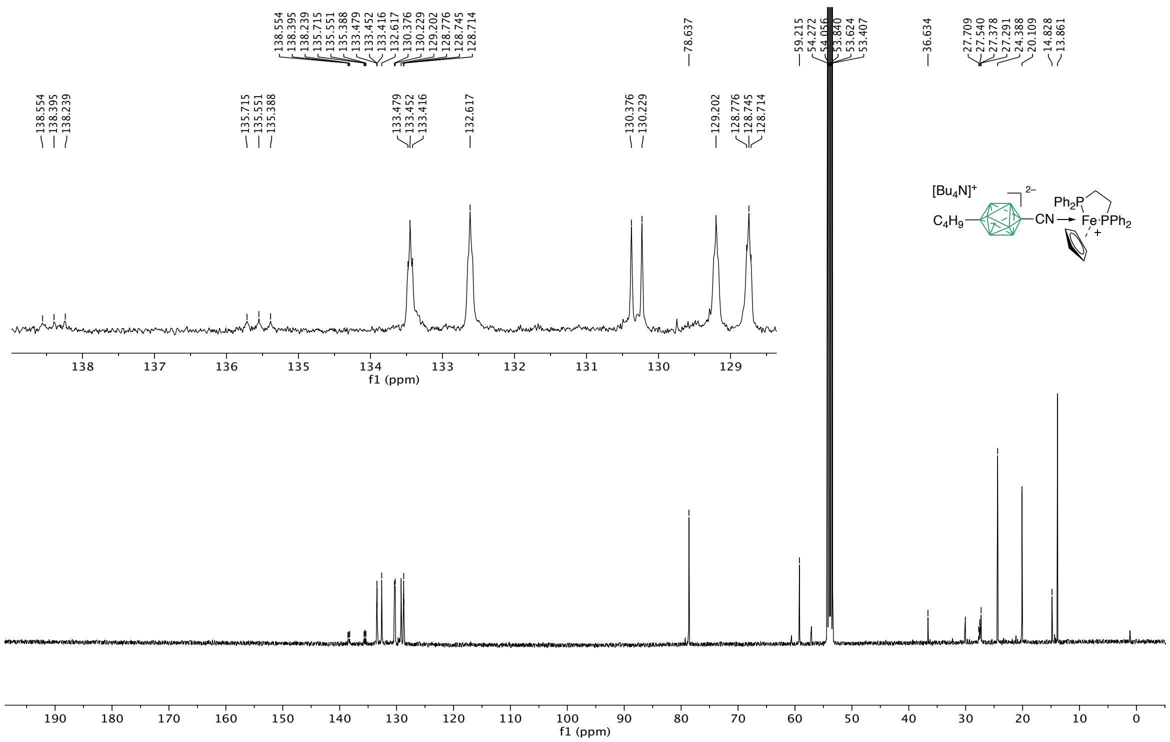

Figure S50. $^{13}C\{^1H\}$ NMR spectrum of [{(η5-Cp)(dppe)Fe}-*closo*-$B_{10}H_8$-1-CN-10-C_4H_9][Bu_4N] (**9a[Bu$_4$N]**, 126 MHz, CD_2Cl_2).

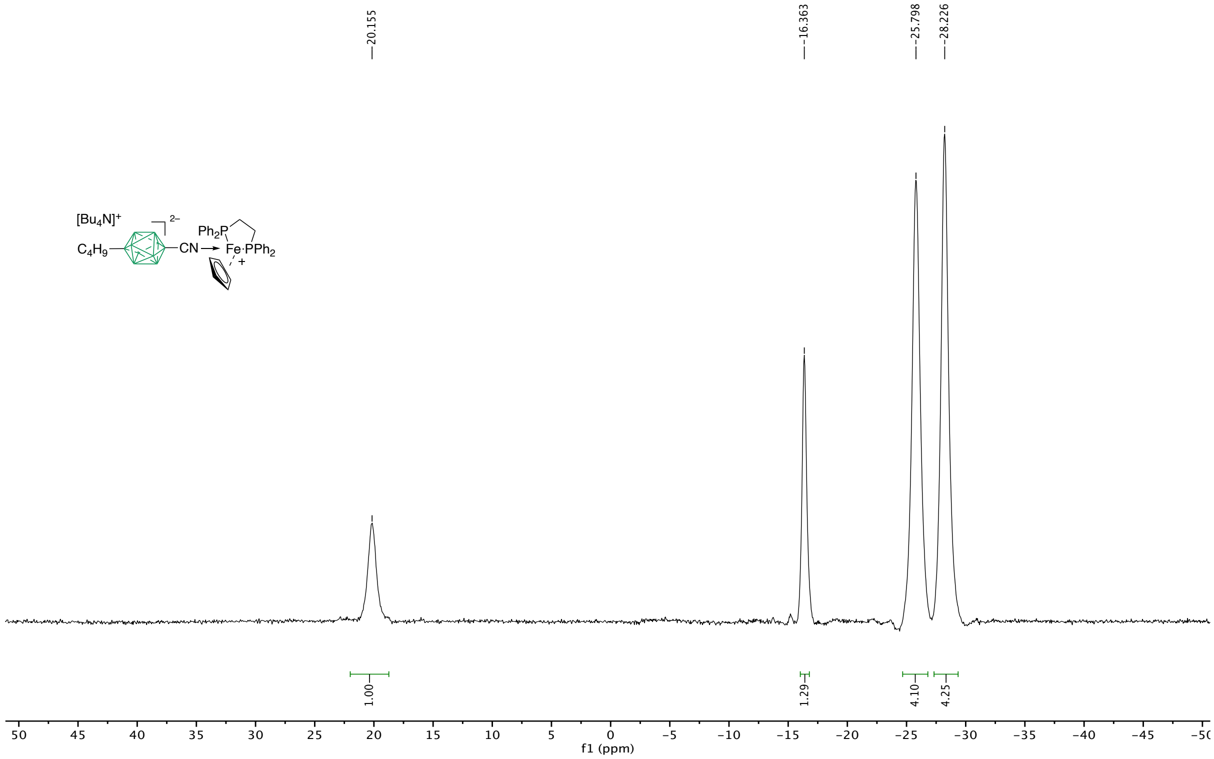

Figure S51. $^{11}B\{^1H\}$ NMR spectrum of [{(η5-Cp)(dppe)Fe}-*closo*-$B_{10}H_8$-1-CN-10-C_4H_9)][Bu_4N] (**9a[Bu$_4$N]**, 193 MHz, CD_2Cl_2).

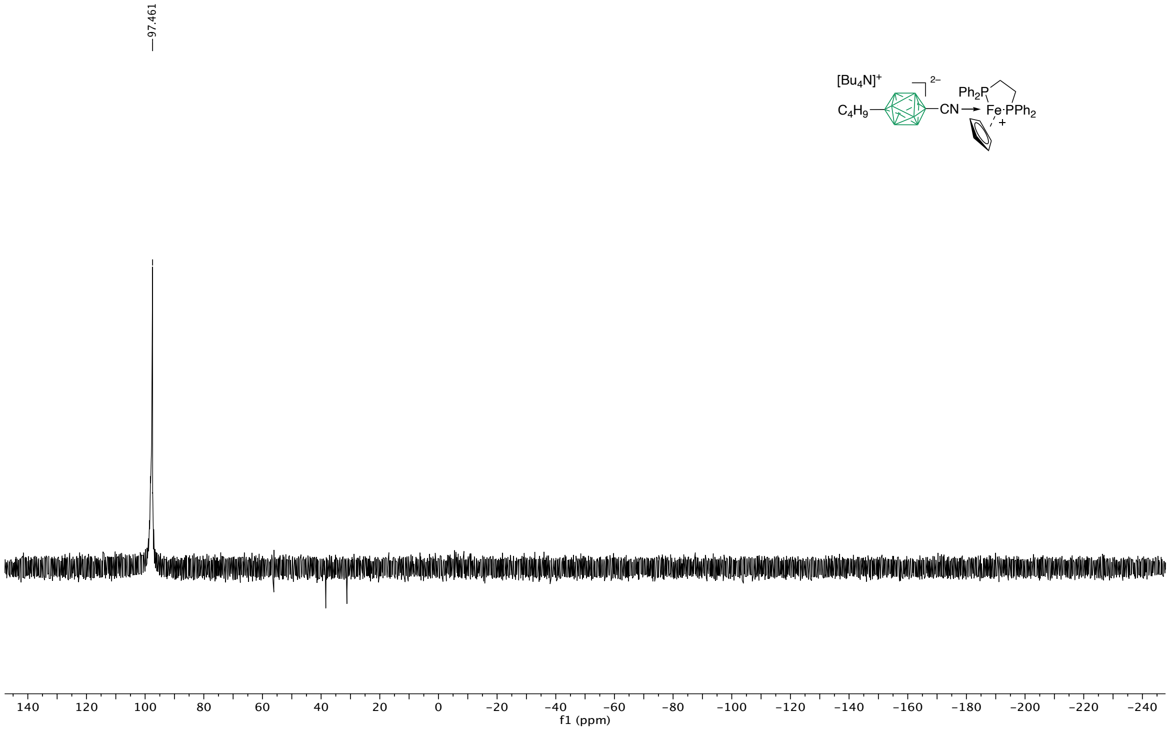

Figure S52. $^{31}P\{^{1}H\}$ NMR spectrum of [{(η5-Cp)(dppe)Fe}-*closo*-$B_{10}H_8$-1-CN-10-C_4H_9)][Bu_4N] (**9a[Bu$_4$N]**, 243 MHz, CD_2Cl_2).

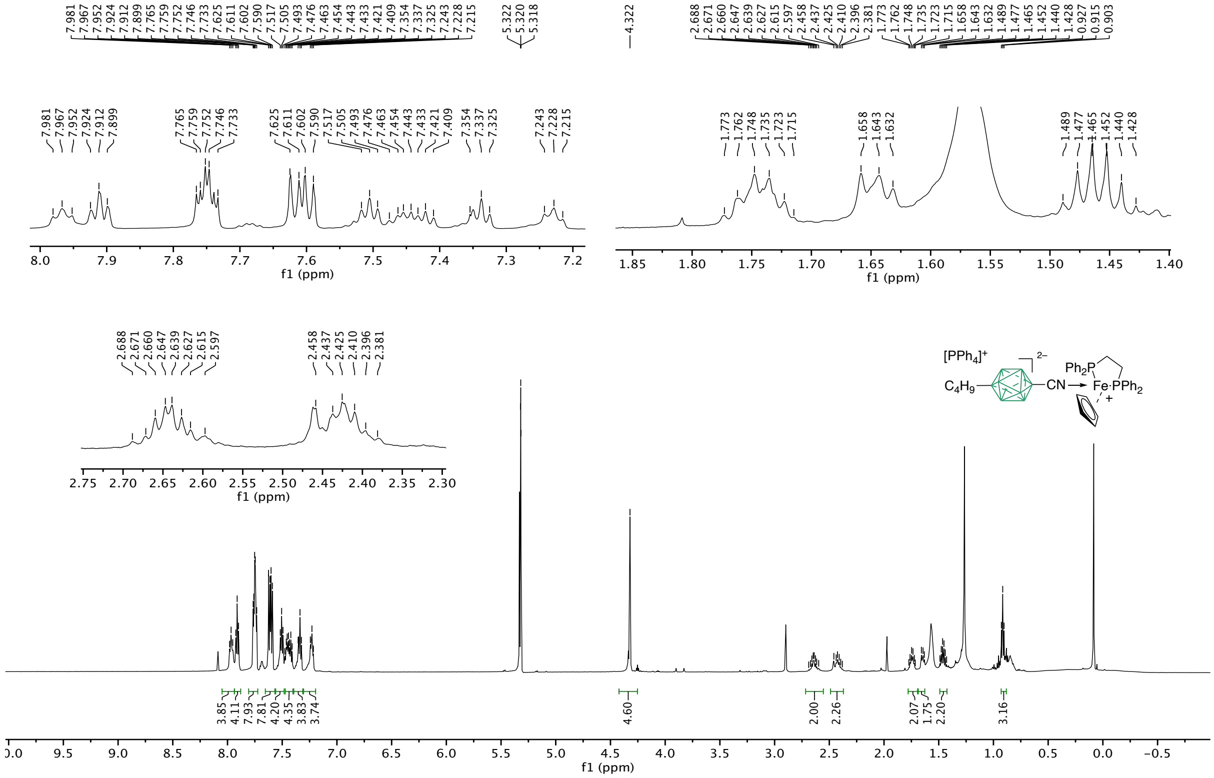

Figure S53. ^{1}H NMR spectrum of [{(η5-Cp)(dppe)Fe}-*closo*-$B_{10}H_8$-1-CN-10-C_4H_9)][Ph_4P] (**9a[Ph$_4$P]**, 600 MHz, CD_2Cl_2).

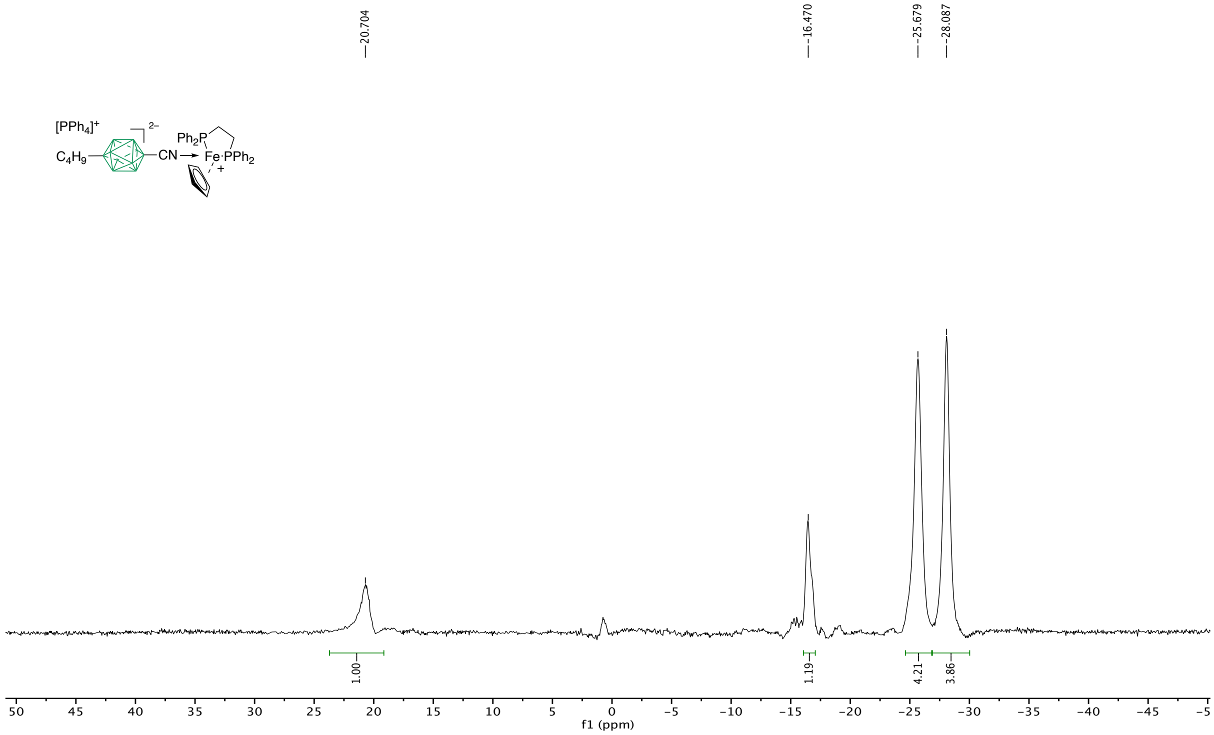

Figure S54. $^{11}B\{^{1}H\}$ NMR spectrum of $[\{(\eta5\text{-Cp})(\text{dppe})\text{Fe}\}\text{-}closo\text{-}B_{10}H_8\text{-1-CN-10-}C_4H_9)][Ph_4P]$ (**9a[Ph$_4$P]**, 193 MHz, CD_2Cl_2).

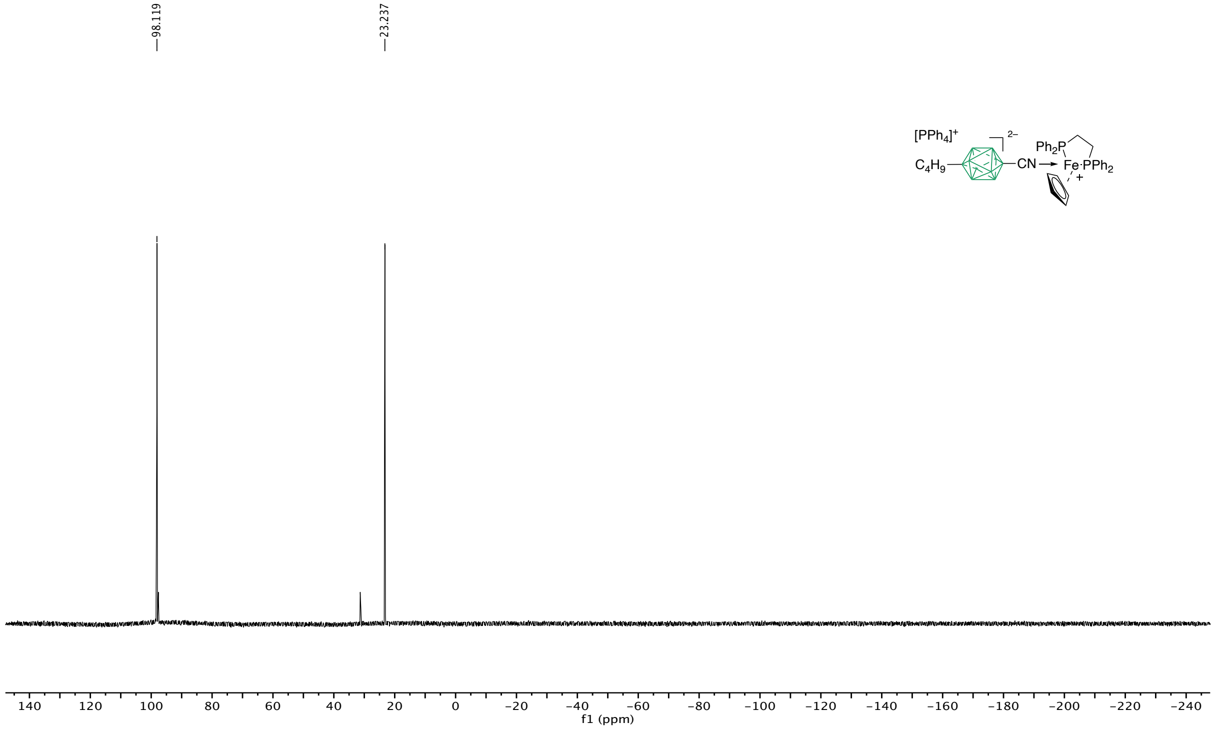

Figure S55. $^{31}P\{^{1}H\}$ NMR spectrum of $[\{(\eta5\text{-Cp})(\text{dppe})\text{Fe}\}\text{-}closo\text{-}B_{10}H_8\text{-1-CN-10-}C_4H_9)][Ph_4P]$ (**9a[Ph$_4$P]**, 243 MHz, CD_2Cl_2).

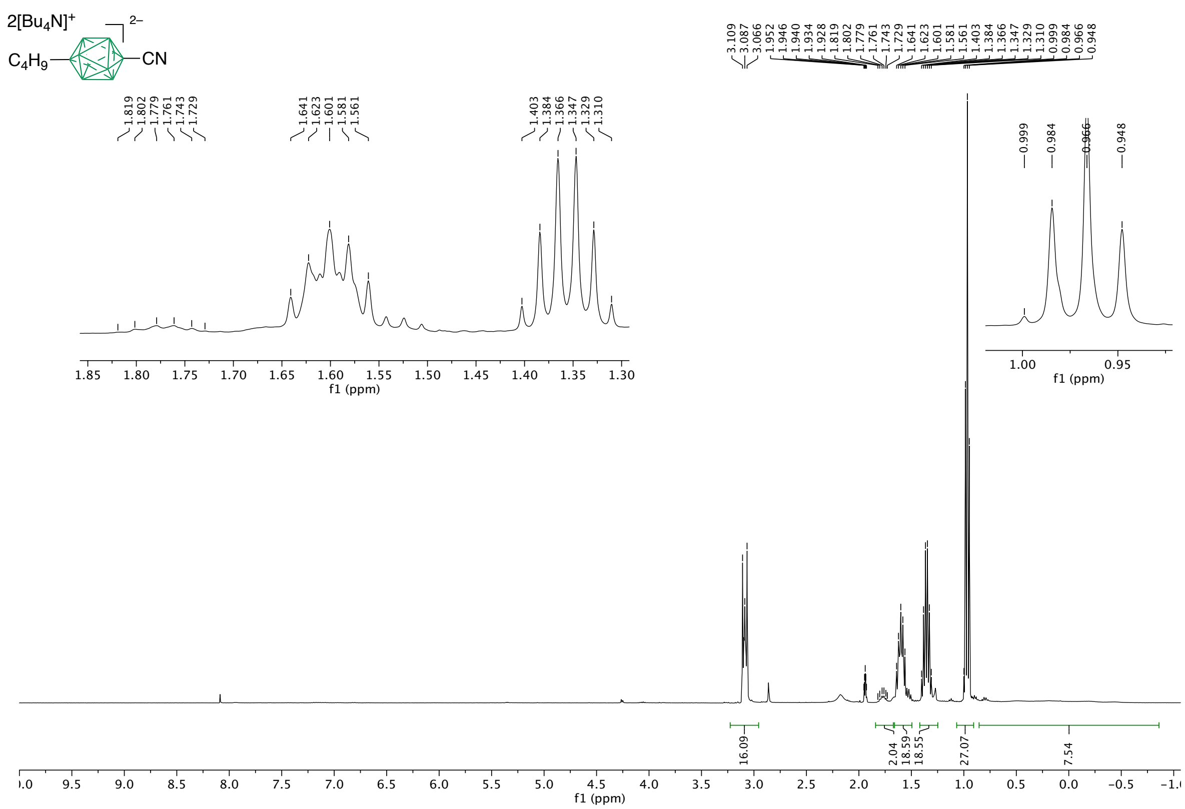

Figure S56. ^{1}H NMR spectrum of [*closo*-$B_{10}H_8$-1-CN-10-C_4H_9][Bu_4N]$_2$ (**10a[Bu$_4$N]**, 400 MHz, CD_3CN).

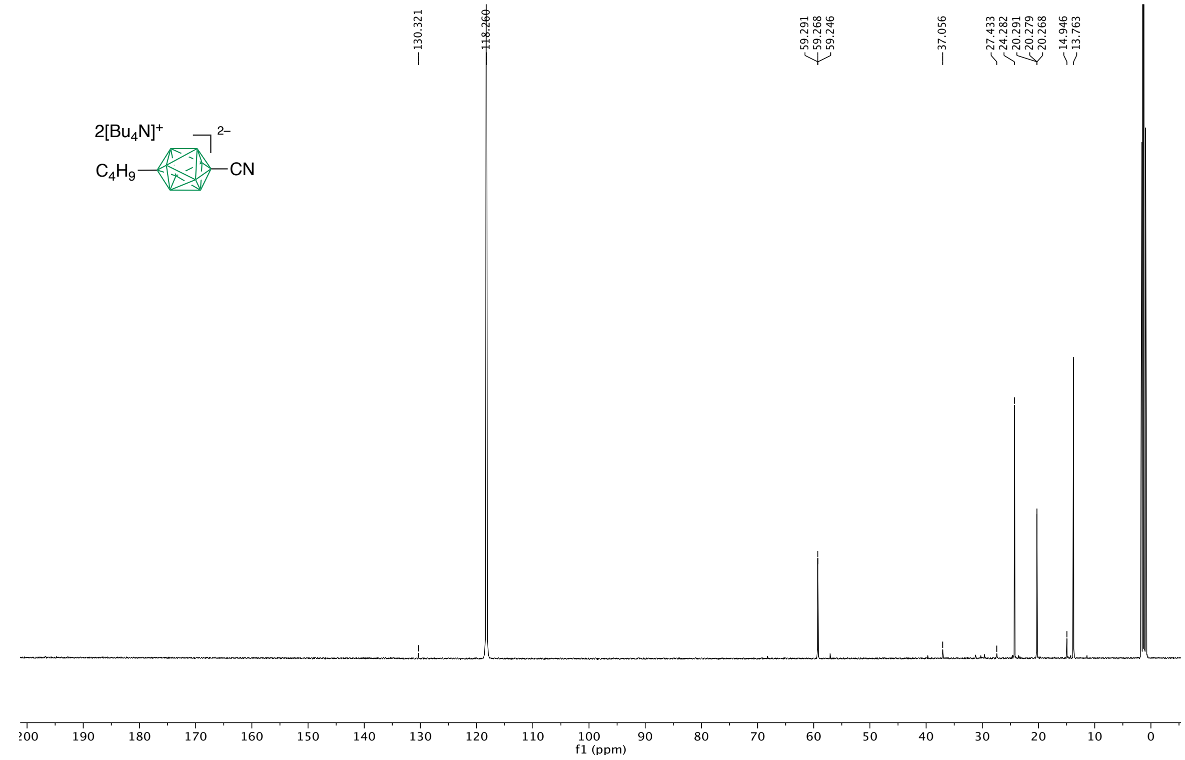

Figure S57. ^{13}C{^{1}H} NMR spectrum of [*closo*-$B_{10}H_8$-1-CN-10-C_4H_9][Bu_4N]$_2$ (**10a[Bu$_4$N]**, 126 MHz, CD_3CN).

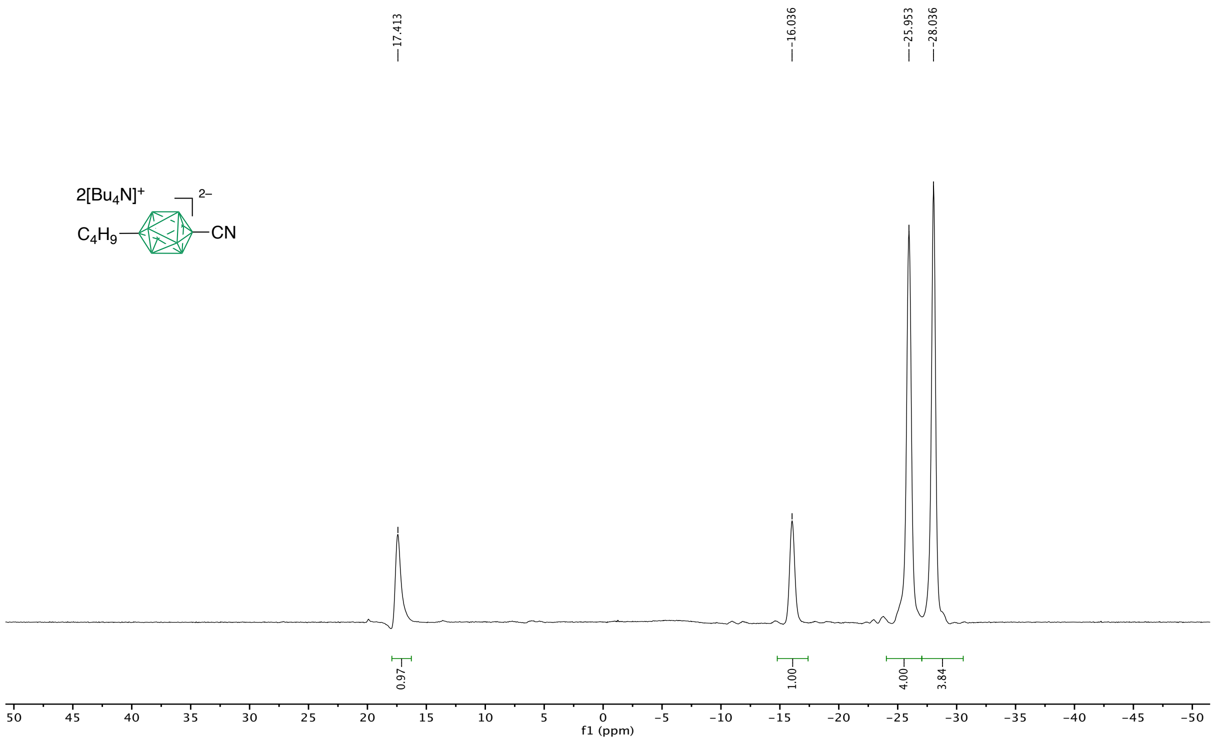

Figure S58. $^{11}B\{^{1}H\}$ NMR spectrum of [*closo*-$B_{10}H_8$-1-CN-10-C_4H_9][Bu_4N]$_2$ (**10a[Bu$_4$N]**, 128 MHz, CD_3CN).

3. IR spectrum of 9a[Bu$_4$N].

IR spectrum of Fe(II) complex **9a[Bu$_4$N]** was recorded for a neat sample using an ATR attachment and shown in Figure S59.

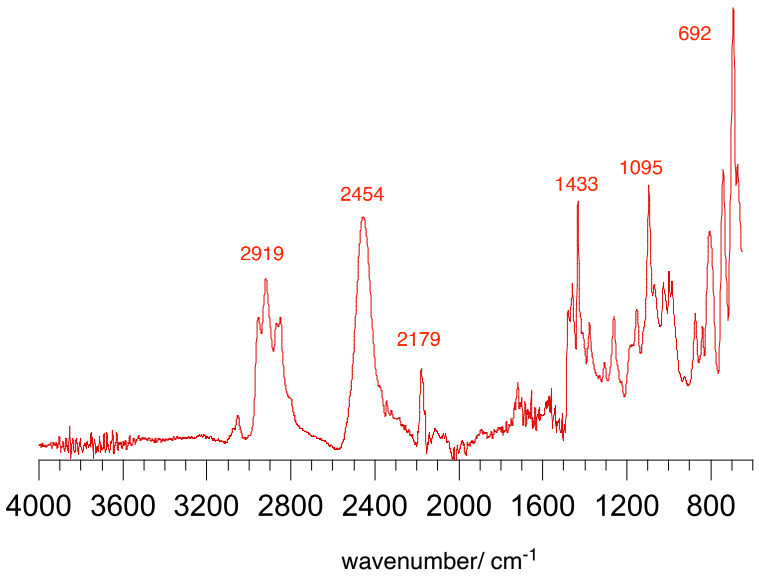

Figure S59. IR spectrum of **9a[Bu$_4$N]**.

4. XRD data collection and refinement details

a) general comments

All crystal data were collected on a dual source Rigaku SuperNova diffractometer with a Dectris Pilatus3 R 200 K-A detector, equipped with an Oxford Cryosystems Ltd. nitrogen flow apparatus (Cryostream 800 Series) at 100 K using micro-focus X-ray Source Cu K_α radiation, λ = 1.54184 Å. The data were integrated using CrysAlisPro program.[7] Intensities for absorption were corrected using gaussian method as in SCALE3 ABSPACK scaling algorithm implemented in CrysAlisPro program.[7] The crystal data and structure refinement descriptors for all structures are listed in Table S2.

Files CCDC 2362917 and 2362918 contain supplementary crystallographic data for this paper. These data can be obtained free of charge from the Cambridge Crystallographic Data Centre via www.ccdc.cam.ac.uk/structures

b) structure solution and refinement

Structures were solved with the ShelXT[8] structure solution program and refined in the ShelXL[9] by the full-matrix least-squares minimization on F^2 using OLEX2 software package.[10] All non-hydrogen atoms were refined anisotropically. All hydrogen atoms were included in idealized positions for structure factor calculations using a riding model. In **2a[Ph$_4$P]** one of the phenyl rings shows positional disorder. This was modelled by splitting three carbon atoms over two positions with the final refined occupancy ratio of 0.457(13):0.543(13).

In **1a[Ph$_4$P]•MeOH** two of the phenyl groups show positional disorder. This was modeled by splitting the six carbon atoms over two positions with the final refined occupancy ratio of 0.380(7):0.620(7) for one phenyl ring and by splitting the three carbon atoms over two positions with the final refined occupancy ratio of 0.199(6):0.801(6) for the second phenyl ring. The solvate methanol molecule in **1a[Ph$_4$P]•MeOH** is disordered over two overlapping positions. This was modelled by splitting the oxygen atom over two positions with the final refined occupancy ratio of 0.249(4):0.751(4). Constraints and restraints, such as EADP, SADI, RIGU, and SIMU were used to aid the disorder modelling.

c) data analysis

Analysis of the solid-state structure of **1a[Ph_4P]•MeOH** shows that the {B_{10}} cage is elongated (the B(1)···B(10) distance is 3.729(3) Å, Figure S59) relative to the parent anion **A** (3.717(4) Å, ref[11]), with the largest effect observed for the height of the tetragonal pyramid adjacent to the alkyl chain (1.114 Å in **1a** vs 1.100 Å in **A**). This is consistent with a general trend in the geometry of derivatives of **A**, that the less electron withdrawing the substituent, the larger separation of the apical positions.[6, 12]

The butyl chain adopts a nearly ideally staggered orientation relative to the cluster and a gauche conformation at the C(3) carbon atom. Partial packing diagram of **1a[Ph_4P]•MeOH** is shown in Figure S60.

Table S2. Crystallographic data for selected derivatives.

Compound	**1a[Ph$_4$P]•MeOH** 2362917	**2a[Ph$_4$P]** 2362918
Formula	$C_{53}H_{62}B_{10}OP_2$	$C_{56}H_{66}B_{10}P_2$
$D_{calc.}$/ g cm^{-3}	1.187	1.173
m/mm^{-1}	1.073	1.030
Formula Weight	885.06	909.12
Colour	yellow	yellow
Shape	plate	plate
Size/mm^3	0.40×0.36×0.14	0.21×0.14×0.06
T/K	99.8(8)	99.9(8)
Crystal System	monoclinic	triclinic
Space Group	$P2_1/n$	*P*-1
a/Å	11.5108(2)	10.61970(10)
b/Å	13.8768(2)	11.52740(10)
c/Å	31.3332(4)	21.5302(3)
a/°	90	92.6460(10)
b/°	98.3290(10)	92.8540(10)
g/°	90	101.7570(10)
V/Å^3	4952.16(13)	2572.90(5)
Z	4	2
Z'	1	1
Wavelength/Å	1.54184	1.54184
Radiation type	Cu K_a	Cu K_a
Θ_{min}/°	2.851	3.923
Θ_{max}/°	76.701	76.723
Measured Refl.	29114	27494
Independent Refl.	10117	10475
Reflections with I > 2(I)	9020	9753
R_{int}	0.0254	0.0208
Parameters	663	643
Restraints	318	91
Largest Peak	0.508	0.863
Deepest Hole	-0.525	-0.372
GooF	1.044	1.046
wR_2 (all data)	0.1682	0.1198
wR_2	0.1639	0.1177
R_1 (all data)	0.0638	0.0442
R_1	0.0583	0.0419

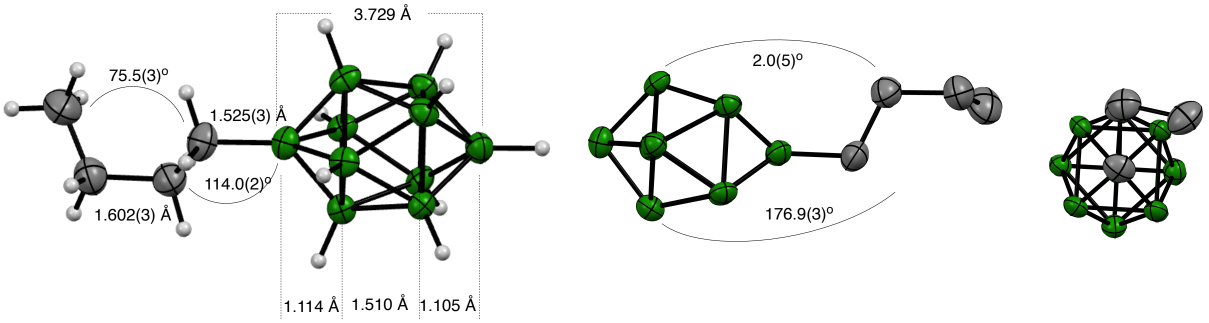

Figure S60. Interatomic dimensions (left), torsion angles defined as B(6)–B(1)–C(1)–(C2) and B(8)–B(1)–C(1)–(C2) (centre) and a Newman projection along the B(10)–B(1)–C(1) axis (right) for anion **1a**.

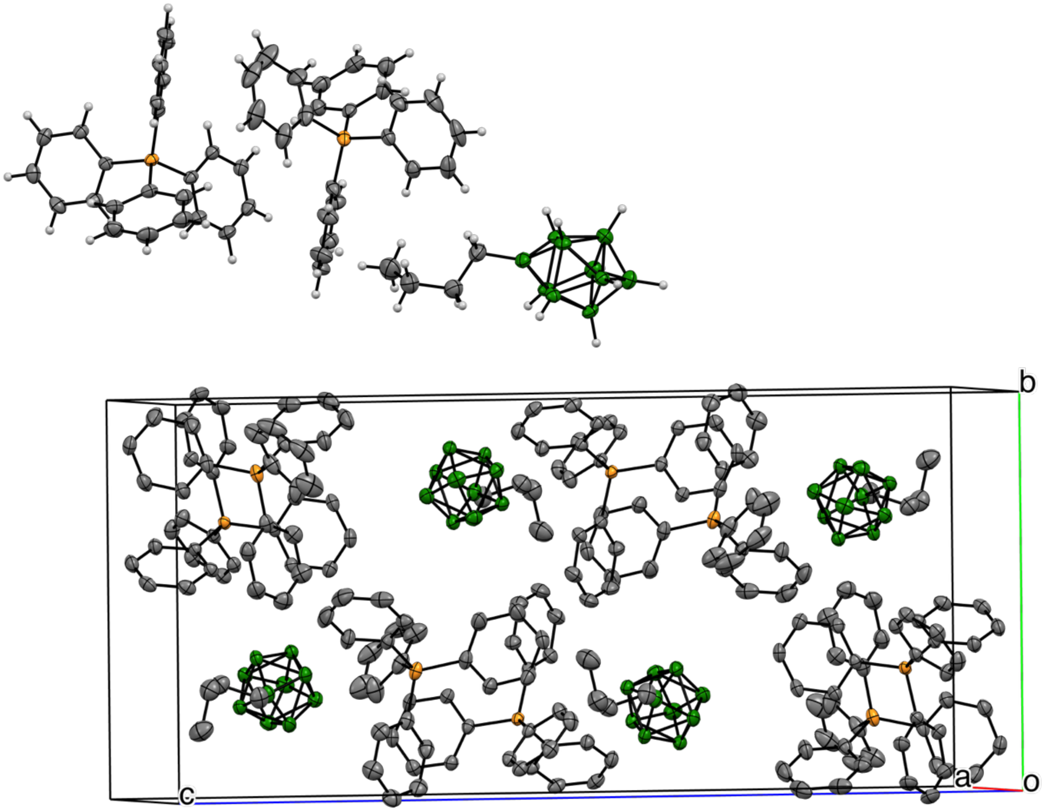

Figure S61. Asymmetric unit (top) and unit cell packing diagram (bottom) for **1a[Ph$_4$P]•MeOH** showing the main disordered structure. Hydrogen atoms and disordered MeOH molecule are omitted for clarity.

The {B_{10}} cage in **2a** is even more elongated (the B(1)···B(10) distance is 3.757(2) Å, Figure S62) relative to the parent anion **A** (3.717(4) Å, ref[11]), with the height of the tetragonal pyramid of 1.119 Å. The butyl chains are in all-*trans* conformation and adopt an eclipsed orientation relative to the {B_{10}} cage (Figure S62). The angle between the planes defined by the B–C–C–C atoms is 28.8° with a pseudo-*syn* relative orientation. Partial packing diagram for **2a[Ph$_4$P]** is shown in Figure S63.

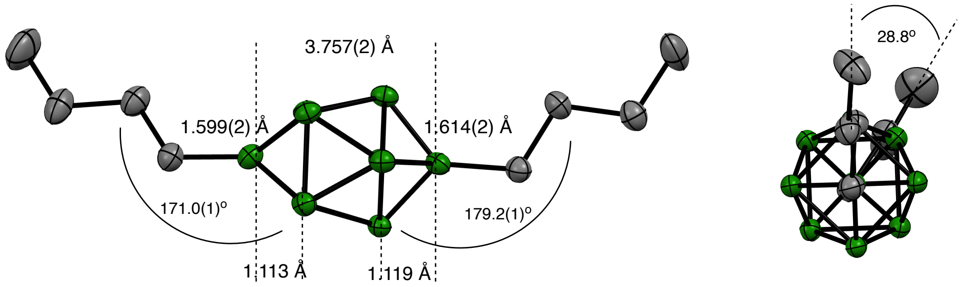

Figure S62. Interatomic dimensions and torsion angles defined as B(2)–B(1)–C(1)–(C2) and B(9)–B(10)–C(1')–(C2') (left) and a Newman projection along the C(1')–B(10)–B(1)–C(1) axis (right) for anion **2a**.

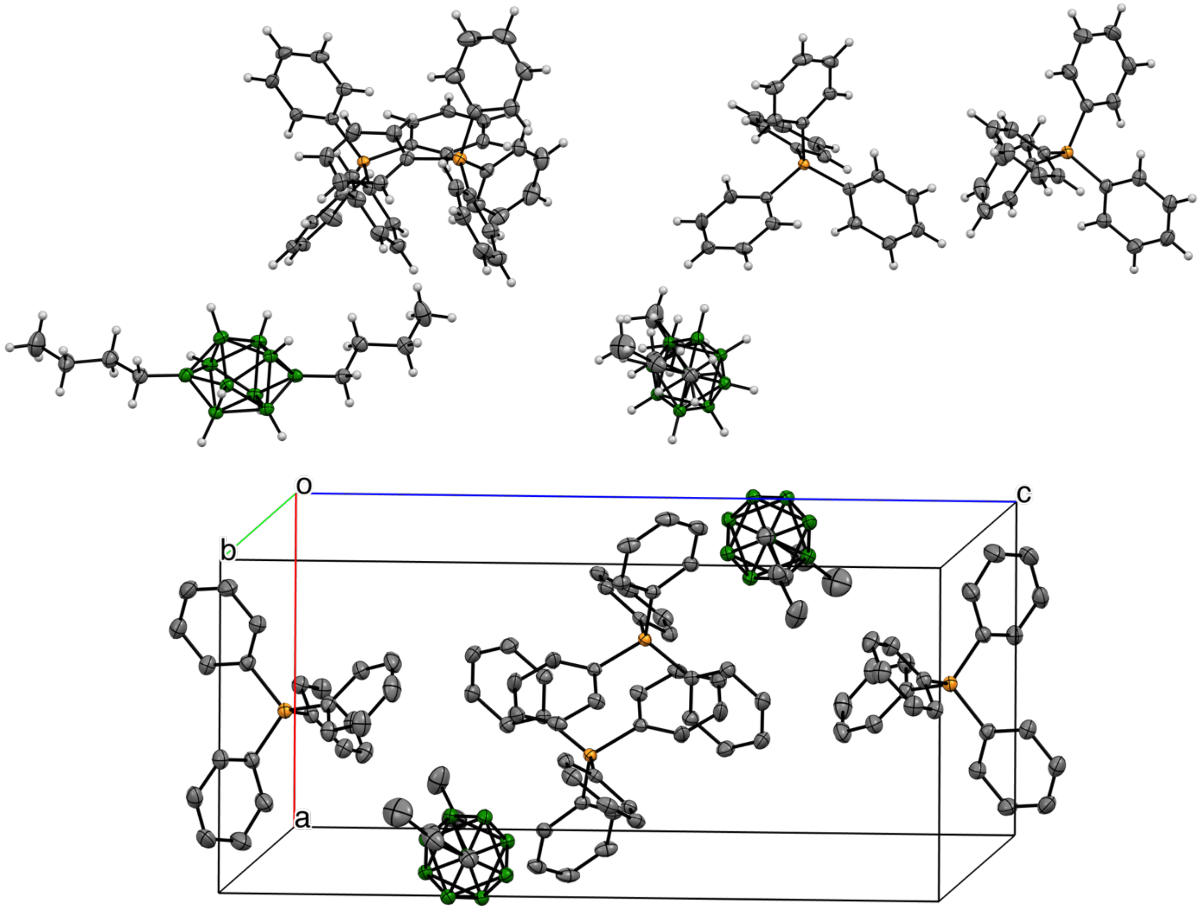

Figure S63. Two views of the asymmetric unit (top) and unit cell packing diagram (bottom) for **2a[Ph$_4$P]**. Hydrogen atoms are omitted for clarity.

The B(1)···B(10) distance, $d_{B1\ldots B10}$ and the height of the square pyramid, *h*, for a series of nine symmetric derivatives of the general structure [*closo*-$B_{10}H_8$-1,10-X_2]$^{2-}$ correlate well with the Hammett substituent parameters σ_p.[13] Results are shown in Figure S63. Structural data taken from literature: X = N_2^+,[14] PhI^+,[12a] CN,[12a] COOEt,[15] Me_2S^+,[16] Pyr^+,[12b] and ROPh.[6]

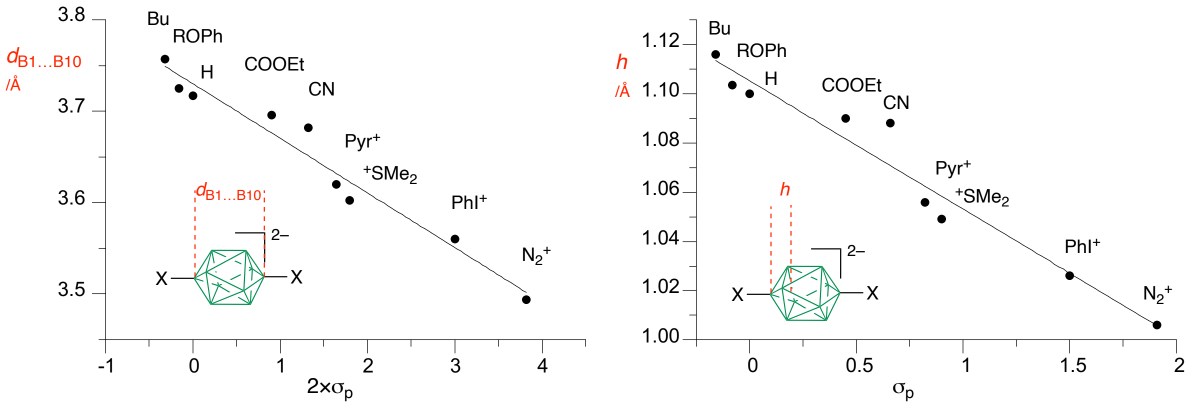

Figure S64. Left: Correlation of the B(1)···B(10) distance, $d_{B(1)...B(10)}$, with sum of the Hammett σ_p parameters in the parent **A** and its symmetric 1,10-disubtituted derivatives. Best fitting line: d = -0.060(5)×2σ_p+3.730(9); r^2 = 0.96. Right: Correlation of the height, h, of the square pyramid with the Hammett σ_p parameter in the parent **A** and its symmetric 1,10-disubtituted derivatives. Best fitting line: h = -0.052(4)×σ_p+1.105(4); r^2 = 0.95.

5. Electronic absorption spectra

Electronic absorption spectra were measured typically for four concentrations in a range of 10^{-5} to 10^{-6} M in spectrophotometric grade MeCN and CH_2Cl_2. The Beer's law plot slope was used to determine molar extinction coefficient ε with a typical correlation parameter r^2 >0.99 for each compound sample. Quantitative spectra are shown in Figures S65–S67.

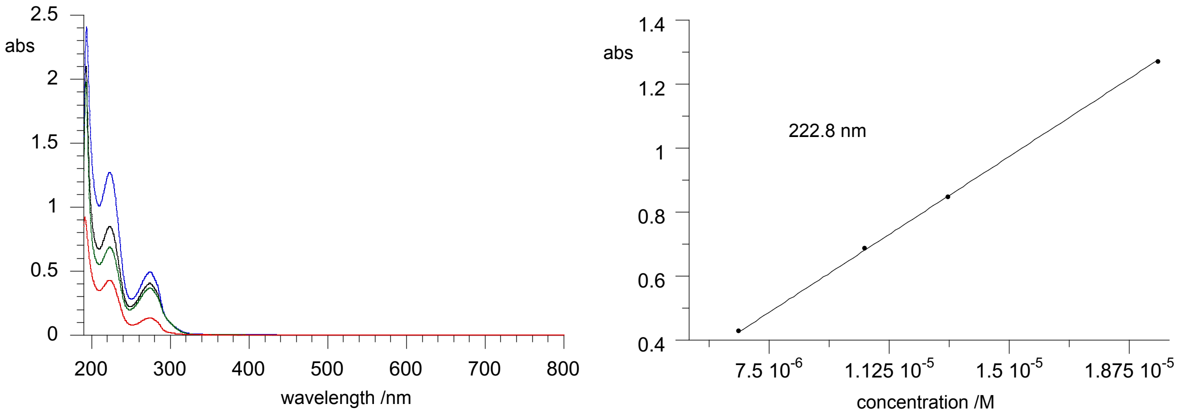

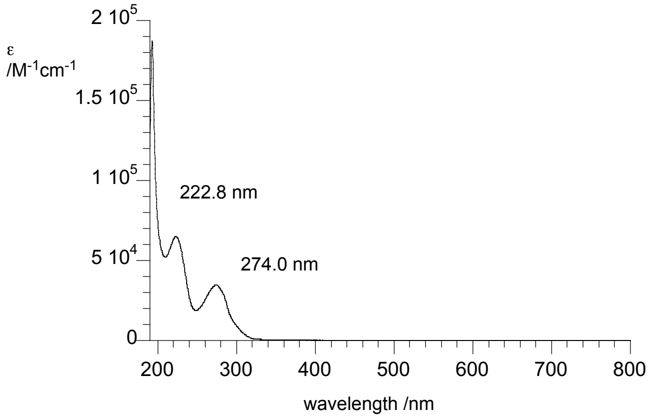

Figure S65. Clockwise: electronic absorption spectra for **2b[PyrCOOC$_{11}$]** in CH_3CN for 4 concentrations; determination of molar extinction coefficient ε at λ = 222.8 nm (best fit function: ε = 64925(201)×conc, r^2 = 0.9998); molar extinction.

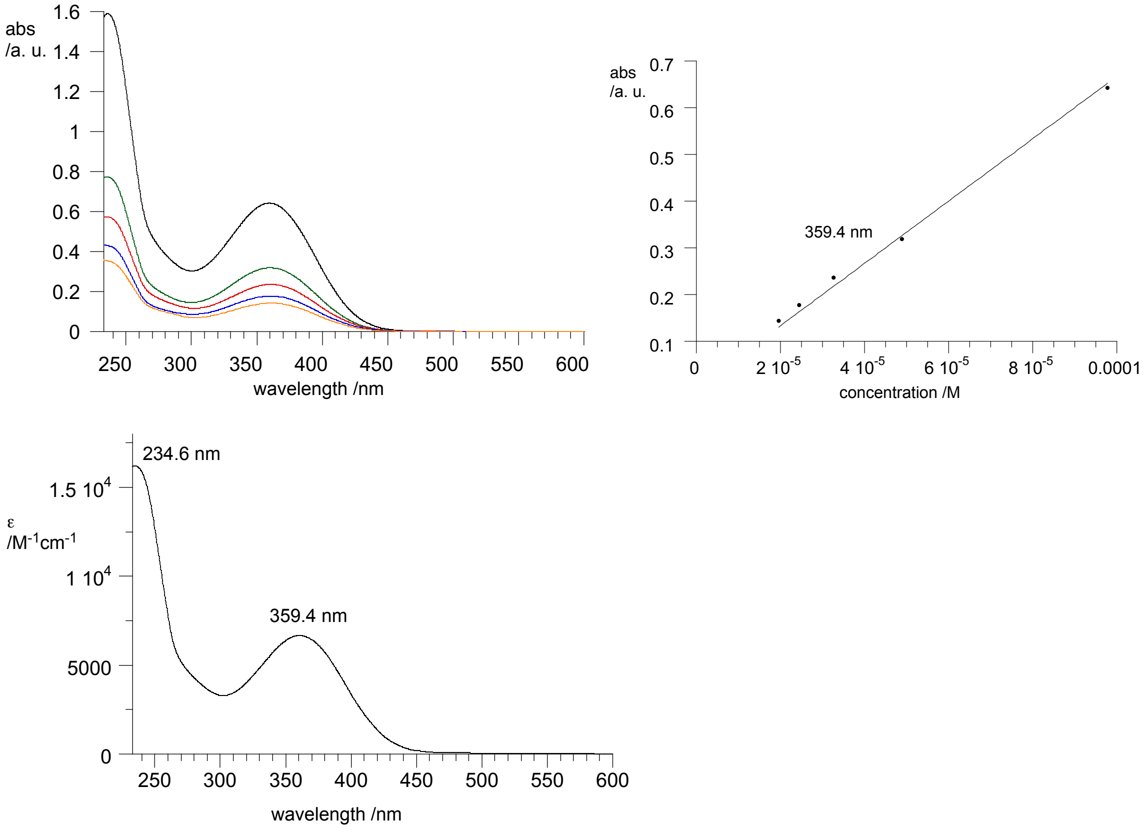

Figure S66. Clockwise: electronic absorption spectra for **8b[Bu$_4$N]** in CH_2Cl_2 for 5 concentrations; determination of molar extinction coefficient ε at λ = 359.4 nm (best fit function: ε = 6670(126)×conc, r^2 = 0.9945); molar extinction.

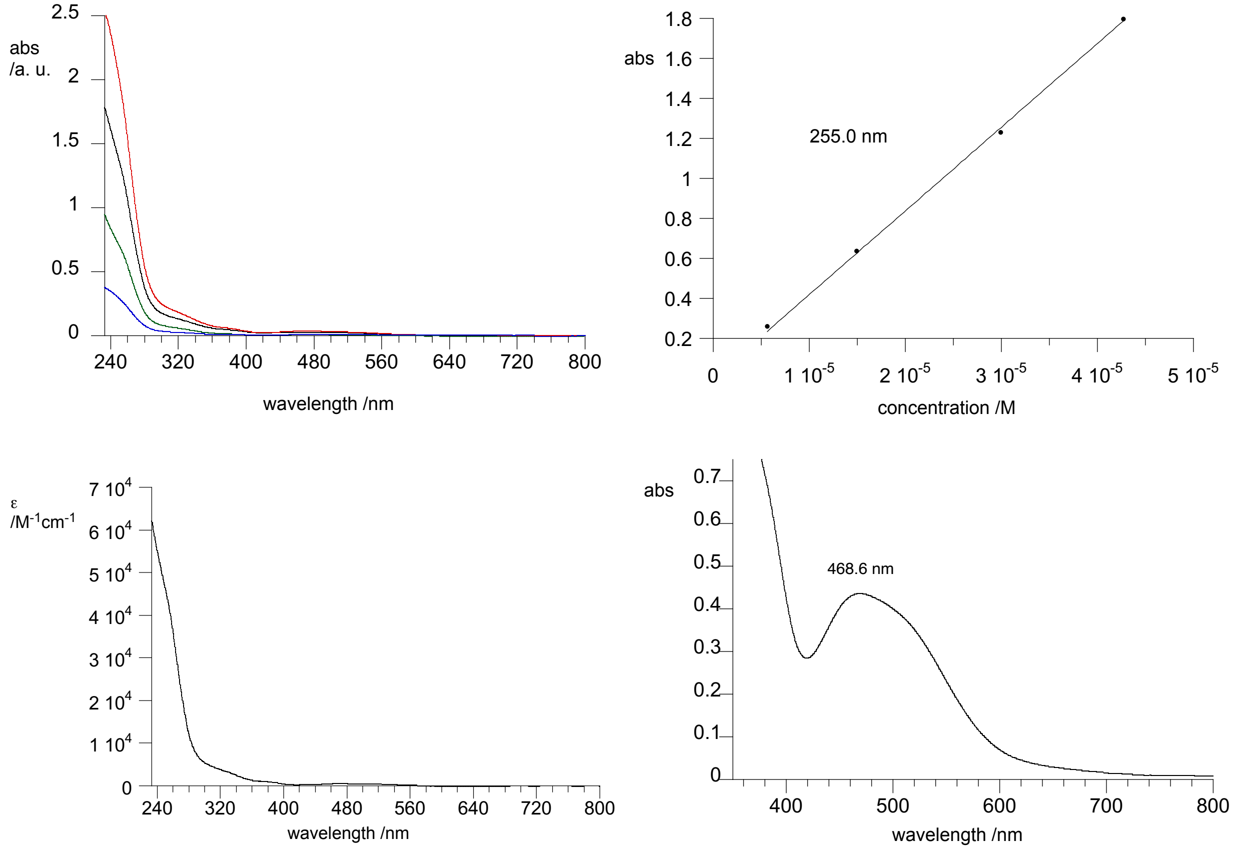

Figure S67. Clockwise: electronic absorption spectra for **9a[Bu$_4$N]** in CH_2Cl_2 for 4 concentrations; determination of molar extinction coefficient ε at λ = 255.0 nm (best fit function: ε = 41852(358)×conc, r^2 = 0.9991); molar extinction. Low energy absorption region of spectra is magnified (higher concentration).

For observation of the intermolecular CT band in pyridinium ion pairs of anions **2b** and **8b**, electronic absorption spectra were recorded in concentrated CH_2Cl_2 solutions using *ca*. 2 mg of the sample in 2 mL of the solvent. Results are shown in Figures S67–S70. Summary of the results is provided in Table S3.

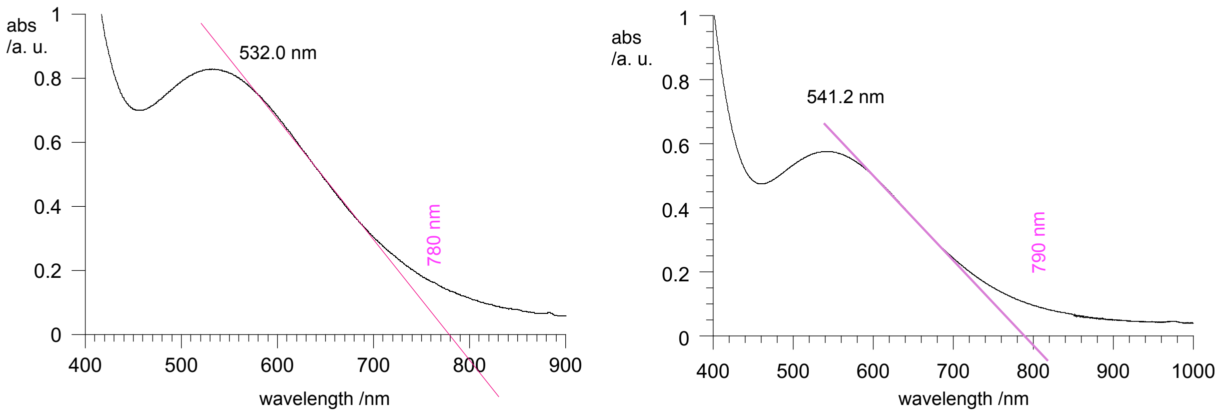

Figure S68. Normalized electronic absorption spectra obtained for **2b[PyrCOOC$_{11}$]** in CH_2Cl_2 (left) and MeCN (right) with indicated absorption maximum and absorption edge of the CT band.

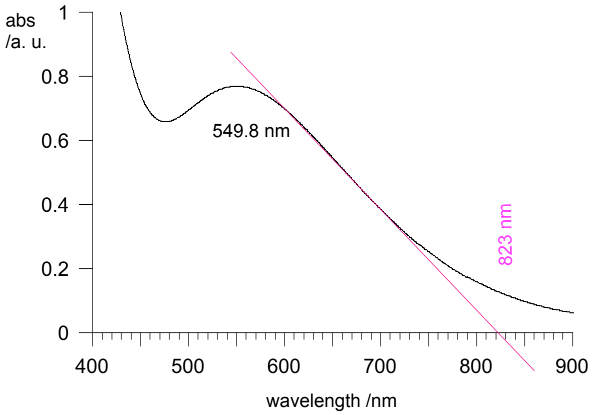

Figure S69. Electronic absorption spectrum obtained for **2b[PyrCN]** in CH_2Cl_2 with indicated absorption maximum and absorption edge of the CT band for high concentration.

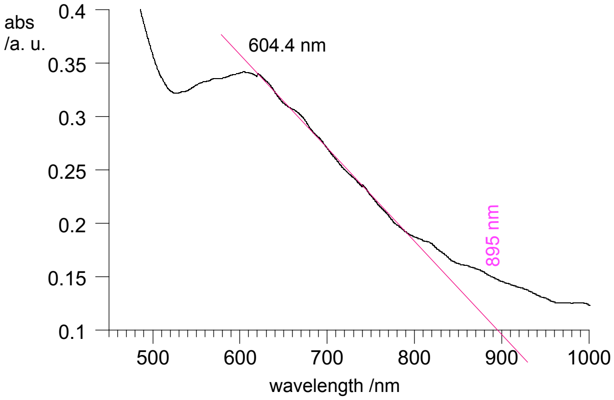

Figure S70. Electronic absorption spectrum obtained for **2b[Q12]** in CH_2Cl_2 with indicated absorption maximum and absorption edge of the CT band for high concentration.

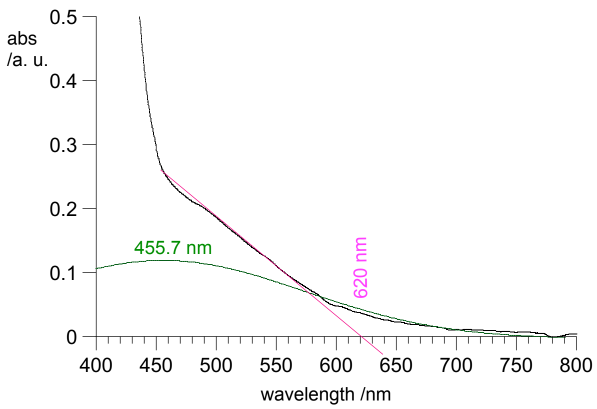

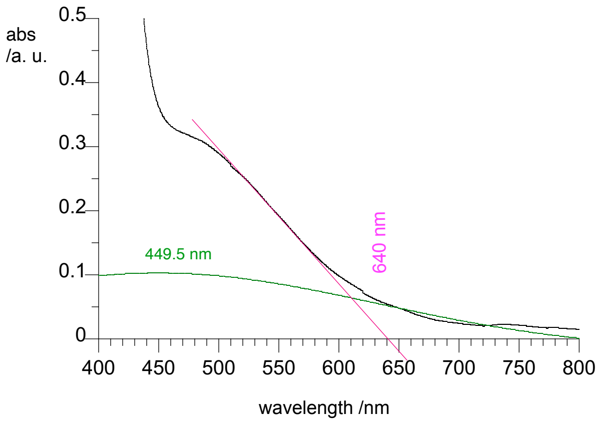

Figure S71. Electronic absorption spectrum obtained for **8b[PyrCOOC$_6$]** (left) and **8b[PyrCOOC$_{11}$]** (right) in CH_2Cl_2 with indicated absorption maximum and absorption edge of the CT band. The low energy absorption region of spectra is deconvoluted.

Table S3. Energies of CT absorption bands and absorption onset in CH_2Cl_2 for selected ion pairs.

Ion pair	λ_{max} /nm, eV	abs onset /nm, eV
**2b[PyrCOOC$_{11}$]**	532, 2.33	780, 1.59
**2b[PyrCN]**	550, 2.25	823, 1.51
**2b[Q12]**	604, 2.05	895, 1.385
**8b[PyrCOOC6]**	450, 2.756	640, 1.938

A correlation of the CT band energy with the difference between the E_{HOMO} and E_{LUMO} is shown in Figure S72.

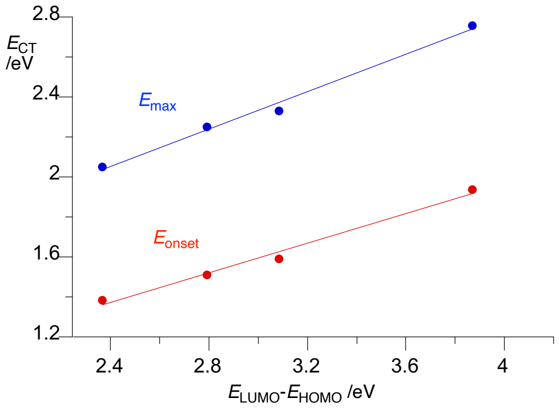

Figure S72. Correlation of maximum of absorption and onset of absorption for the CT bands. Best fitting functions: $E_{max} = 0.47(3)\times\Delta E_{FMO} +0.93(10)$, $r^2 = 0.991$; $E_{onset} = 0.37(3)\times\Delta E_{FMO} +0.48(10)$, $r^2 = 0.986$.

6. Electrochemical data

Electrochemical analysis was conducted using a Metrohm Autolab potentiostat. Cyclovoltammetric (CV) measurements were performed in degassed solutions of 0.5 mM analyte in 100 mM $[Bu_4N][PF_6]$ solution in MeCN (CH_2Cl_2 for **9a[Bu₄N]**) as the supporting electrolyte. The voltammograms were recorded at *ca* 22 °C using a glassy carbon working electrode (φ = 1 mm), a Pt-wire counter electrode and an Ag/AgCl wire as pseudo-reference electrode at a scan rate of 100 mV s^{-1} and typical range -0.5 to +1 V. The potentials are referenced to the Fc/Fc^+ couple (set as 0.0 V) using results from separate measurements (second scan) of solutions containing decamethylferrocene ($Me_{10}Fc$) as an internal reference. The oxidation potential for $Me_{10}Fc$ *vs* the Fc/Fc^+ couple was determined to be -0.509 V in MeCN (Figure S72) and -0.56 V in CH_2Cl_2. Electrochemical results for derivatives of **A** are shown in Figures S73–S80 and summarized in Table S4.

Derivatives [*closo*-$B_{10}H_9$-1-CN]$[Bu_4N]_2$, [*closo*-$B_{10}H_8$-1,10-CN]$[Bu_4N]_2$ used for measurements were described recently.[12] Electrochemical analysis of the parent anion **A** was investigated recently.[17]

Electrochemical analysis of the series using CH_2Cl_2 as the solvent gave $E_{1/2}^{0/+1}$ potentials which exhibit non-linear correlation with the DFT calculated E_{HOMO} energies and Hammett parameters σ_p, presumably due to aggregation of the ion pairs in solutions.

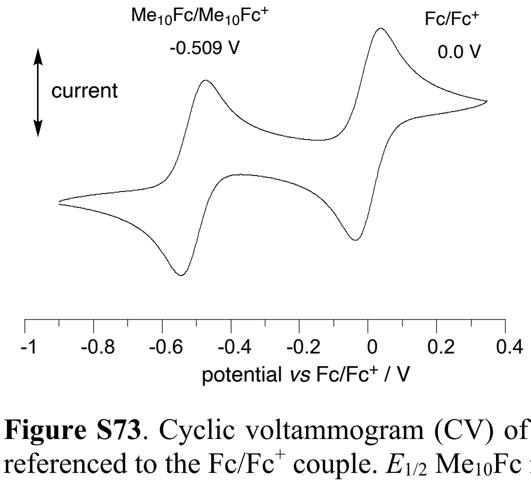

Figure S73. Cyclic voltammogram (CV) of ferrocene (Fc) and decamethylferrocene ($Me_{10}Fc$) in MeCN referenced to the Fc/Fc^+ couple. $E_{1/2}$ $Me_{10}Fc$ relative to Fc is -0.509 V.

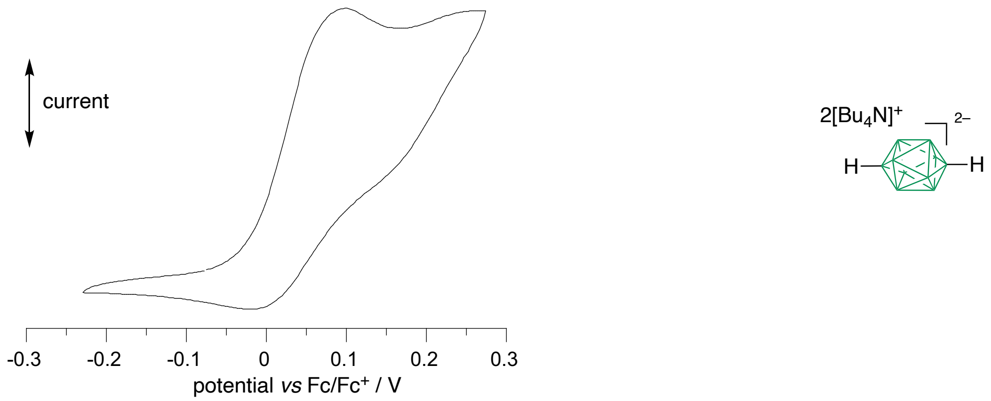

Figure S74. Cyclic voltammogram (CV) of [*closo*-$B_{10}H_{10}$][Bu_4N]$_2$ (**A[Bu_4N]**) in MeCN referenced to the Fc/Fc^+ couple. From CV: $E_{1/2}$ = 0.039 V.

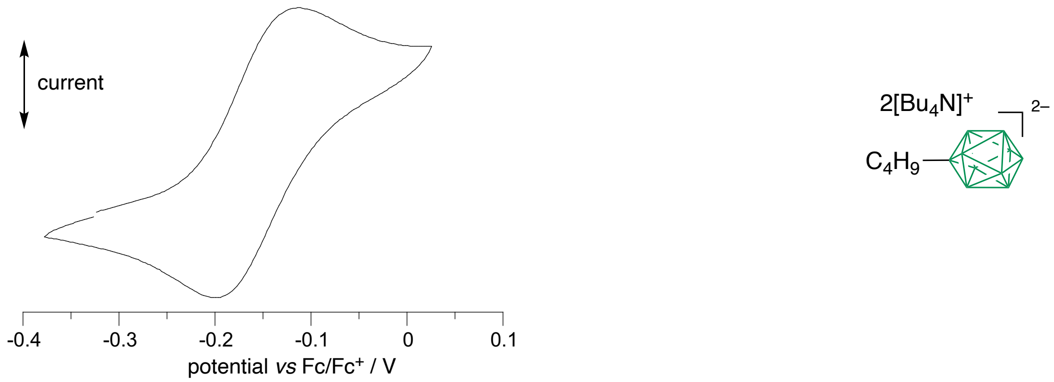

Figure S75. Cyclic voltammogram (CV) of [*closo*-$B_{10}H_9$-1-C_4H_9][Bu_4N]$_2$ (**1a[Bu_4N]**) in MeCN referenced to the Fc/Fc^+ couple. From CV: $E_{1/2}$ = -0.155 V.

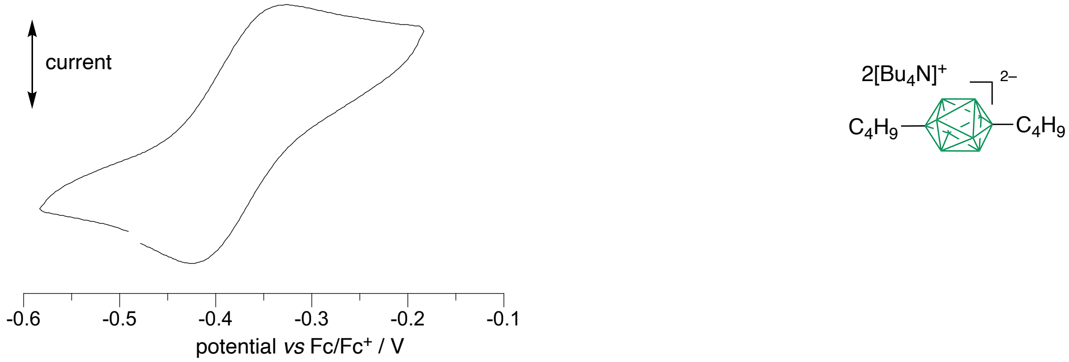

Figure S76. Cyclic voltammogram (CV) of [*closo*-$B_{10}H_8$-1,10-C_4H_9][Bu_4N]$_2$ (**2a[Bu_4N]**) in MeCN referenced to the Fc/Fc^+ couple. From CV: $E_{1/2}$ = -0.376 V.

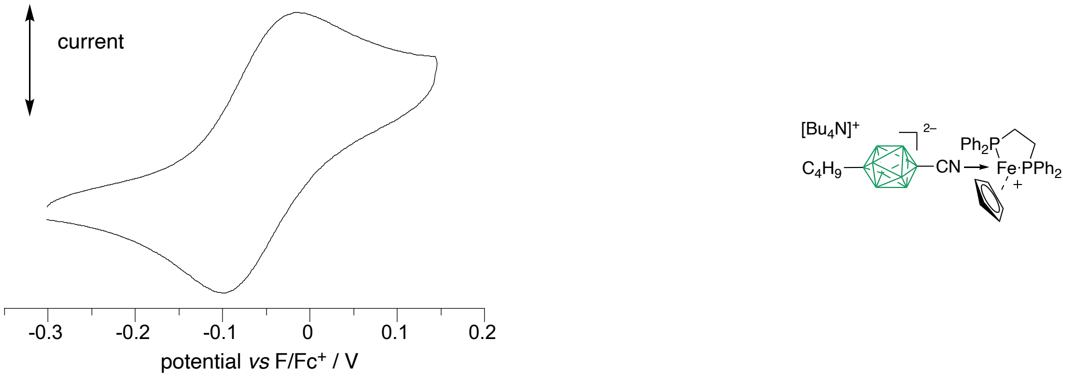

Figure S77. Cyclic voltammogram (CV) of [{(η5-Cp)(dppe)Fe}-*closo*-$B_{10}H_8$-1-CN-10-C_4H_9][Bu_4N] (**9a[Bu$_4$N]**) in CH_2Cl_2 referenced to the Fc/Fc$^+$ couple. From CV: $E_{1/2}$ = -0.054 V.

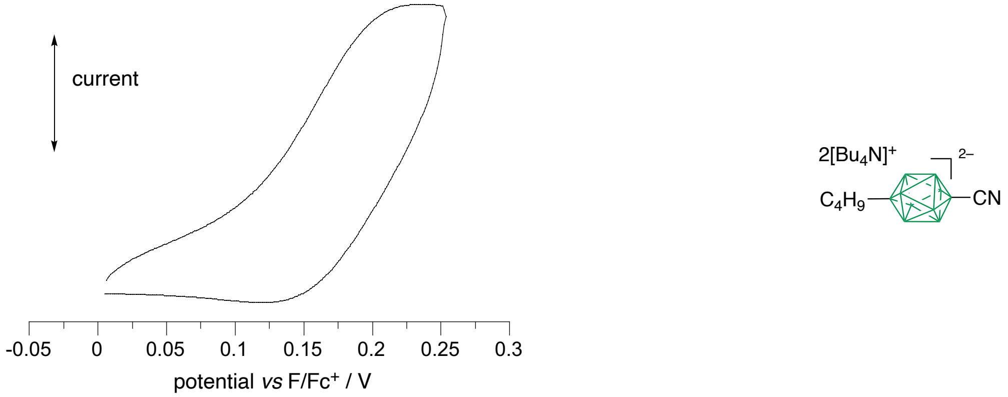

Figure S78. Cyclic voltammogram (CV) of [*closo*-$B_{10}H_9$-1-CN-10-C_4H_9][Bu_4N]$_2$ **(10a[Bu$_4$N])** in MeCN referenced to the Fc/Fc$^+$ couple. From CV: $E_{1/2}$ = 0.176 V.

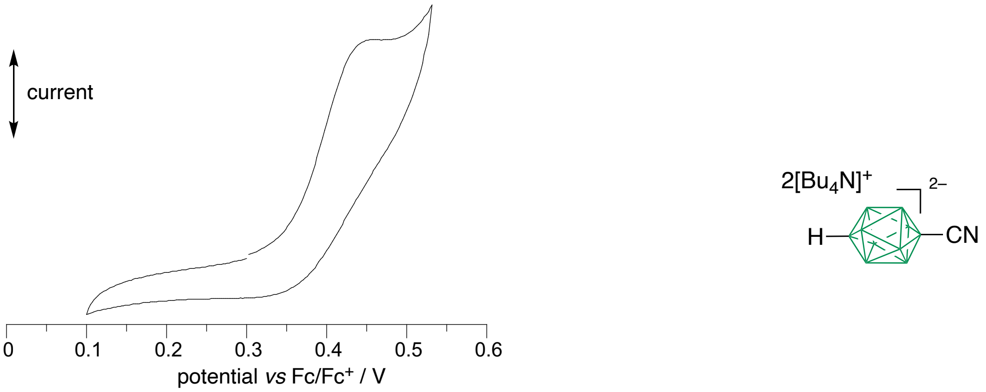

Figure S79. Cyclic voltammogram (CV) of [*closo*-$B_{10}H_9$-1-CN][Bu_4N]$_2$ in MeCN referenced to the Fc/Fc$^+$ couple. From CV: $E_{1/2}$ = 0.376 V.

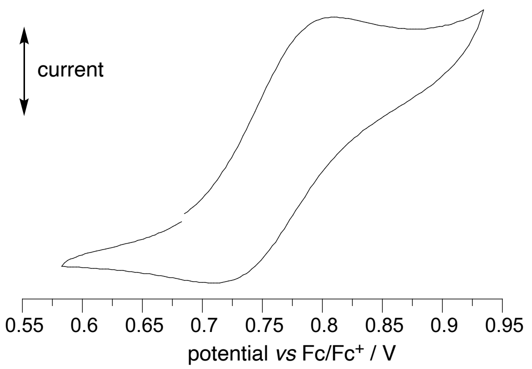

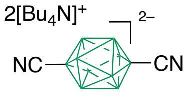

Figure S80. Cyclic voltammogram (CV) of [*closo*-$B_{10}H_8$-1,10-CN][Bu_4N]$_2$ in MeCN referenced to the Fc/Fc^+ couple. From CV: $E_{1/2}$ = 0.759 V.

Table S4. Electrochemical oxidation potentials and DFT energy of the HOMO for derivatives [*closo*-$B_{10}H_8$-1-X-10-Y]$^{2-}$

compound	X	Y	$E_{1/2}^{0/+1\ a}$ /V	HOMO [b] /eV	$\Sigma\sigma_p$ [c]
**A**	H	H	0.039	-5.263	0.00
**1a**	H	Bu	-0.155	-5.029	-0.16
**2a**	Bu	Bu	-0.376	-4.813	-0.32
**10a**	CN	Bu	0.176	-5.352	0.50
	CN	H	0.376	-5.587	0.66
	CN	CN	0.759	-5.903	1.32
	Ph	Ph	-0.140	-4.864	-0.02
	$MeOC_6H_4$	$MeOC_6H_4$	-0.254	-4.685	-0.12

[a] Recorded in MeCN [Bu_4N][PF_6] (100 mM), *ca*. 22 ºC, 100 mV s^{-1}, glassy carbon electrode and reported *vs* Fc/Fc^+ couple. [b] Calculated at the B3LYP/Def2TZVP (MeCN) // B3LYP/Def2TZVP (PhCl) level of theory. [c] Ref. [13]

7. Computational Details

a) general

Quantum-mechanical calculations were carried out with the B3LYP[18] method and Def2TZVP basis sets[19] using Gaussian 16 package[20] for all compounds except for the Fe complex, for which the Def2SVP basis set was used. In all cases geometry optimisations were conducted with tight convergence limits using appropriate symmetry constrains in PhCl dielectric medium requested with SCRF(PCM, Solvent=C6H5Cl).[21] The presence of a weak dielectric medium was

demonstrated to be particularly effective for obtaining accurate molecular geometries.[12a] The ground state nature of the stationary point was verified with vibrational frequency calculations. In model compounds the alkyl chains were replaced with an Et or Me group, while in complex **9a** the dppe ligand was replaced with two PH_3 groups.

b) homolytic bond dissociation energy

Dissociation energy of the Y–Hal bond in selected iodo derivatives and PhCl was calculated at the (U)B3LYP//Def2TZVP level of theory as a change of enthalpy in homolysis in vacuum. Results are shown in Table S5.

$$\text{R–Hal} \rightarrow \text{R}\bullet + \text{Hal}\bullet$$

c) molecular geometry of anions and cations

The ground state geometry of the alkyl derivatives **1**, **2**, and **8b**, has the C_1 symmetry, according to normal mode analysis (frequency calculations). In anion **8b** the heptyloxy chain was approximated as MeO, while the octyl was replaced with Et in the model derivative **8b'**. The structure of the model **8b'** shown in Figure S81 demonstrates that the alkyl chain and pyridine ring planes are nearly co-planar, which enhances the mesogenic derivatives of the ion pairs, such as **8b[PyrCOOC$_6$]**. The length of the core of the anion is 8.0 Å.

Models of cations di-C_{12}-paraquat, PyrCOO and PyrCN were calculated before[6] at the same level of theory.

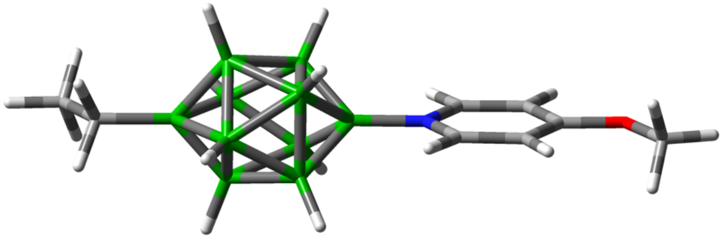

Figure S81. Molecular model of equilibrium geometry for model anion **8b'** obtained at the B3LYP/Def2TZVP level of theory in PhCl dielectric medium.

Table S5. Homolytic bond dissociation energy (HBDE) and energy of MO associated with $\sigma^*_{Y\text{-}I}$ obtained in vacuum.[a]

compound	*HBDE (ΔH)* /kcal mol^{-1}		σ_X $_{Y\text{-}I}$* [b] /eV	type of MO
	DFT	exp		
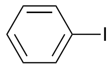	66.0	65.3±2 [c]<br>67±2 [d]<br>67.2 [e]	-1.104	LUMO
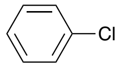	91.1	97.1(6) [d]<br>97.6 [e]		
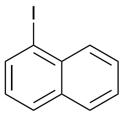	64.8	65.0 [e]		
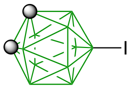	78.2	na	-0.623	LUMO+1
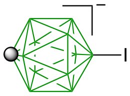	87.8	na	0.213	LUMO
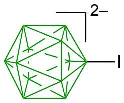	95.2	na	0.952	LUMO
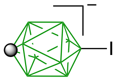	89.7	na	0.615	LUMO+2
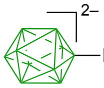 **3**	100.4	na	1.288	LUMO

[a] Obtained with B3LYP/Def2TZVP method in vacuum. [b] MO energy. [c] Ref. [22]. [d] Ref. [23]. [e] Ref. [24]

d) HOMO and ionization energies of anions

For a better analysis of electrochemical oxidation potentials, HOMO energies of anions were calculated at the B3LYP/Def2TZVP (MeCN)// B3LYP/Def2TZVP (PhCl) level of theory. Results are shown in Table S6. For comparison purposes, HOMO energies in PhCl dielectric medium are also provided in Table S6.

In addition, adiabatic ionisation energies I_E were obtained for all anions by comparing SCF energies (geometry optimized at the (U)B3LYP/Def2TZVP in PhCl medium) corrected for ZPE (geometry optimised at (U)B3LYP/Def2SVP in PhCl medium) for dianions of the corresponding

radical anions. Adiabatic I_E energies in MeCN medium, were obtained using B3LYP/def2TZVP (MeCN)//B3LYP/def2TZVP (PhCl) SCF energies corrected for (U)B3LYP/Def2SVP-derived ZPE. Results are shown in Table S6.

Table S6. HOMO energies, E_{HOMO}, and adiabatic ionisation energies, I_E, for anions obtained in PhCl and MeCN dielectric media.

Compound, symmetry	E_{HOMO} in PhCl [a] /eV	E_{HOMO} in MeCN [b] /eV	I_E in PhCl [c] /eV	I_E in MeCN [d] /eV	Sum of σ_p [e]
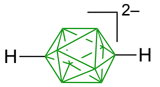 **A**, D_{4d}	-4.143	-5.263	4.069	4.911	0.0
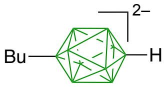 **1a**, C_1	-3.962	-5.029	3.811	4.609	-0.16
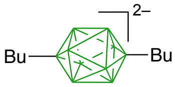 **2a**, C_1	-3.783	-4.813	3.6160	4.392	-0.32
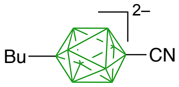 **10a**, C_1	-4.348	-5.352	4.203	4.944	0.50
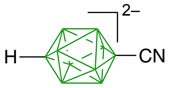 C_{4v}	-4.552	-5.587	4.454	5.221	0.66
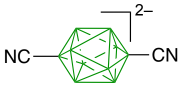 D_{4d}	-4.911	-5.903	4.806	5.537	1.32
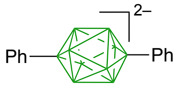 D_2	-3.914	-4.864	3.793	4.520	-0.02
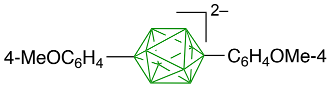 C_2	-3.724	-4.685	3.625	4.342	-0.12

[a] Obtained with B3LYP/def2TZVP method in PhCl dielectric medium. [b] Obtained with B3LYP/def2TZVP (MeCN)//B3LYP/def2TZVP (PhCl) method in MeCN dielectric medium. [c] Adiabatic ionisation energy obtained from E_{SCF}, obtained with (U)B3LYP/def2TZVP method in PhCl dielectric medium, and corrected for ZPE ((U)B3LYP/def2SVP method in PhCl). [d] Adiabatic ionisation energy obtained from E_{SCF}, obtained with (U)B3LYP/def2TZVP (MeCN)//(U)B3LYP/def2TZVP (PhCl) method, and corrected for ZPE ((U)B3LYP/def2SVP method in PhCl). [e] Ref. [13].

Correlation of the calculated E_{HOMO} and adiabatic ionisation energy, $I_{E(adiab)}$, in PhCl and MeCN dielectric media *vs* the Hammett parameters[13] are shown in Figure S82. Analysis demonstrates somewhat lower correlation factor for calculations in MeCN.

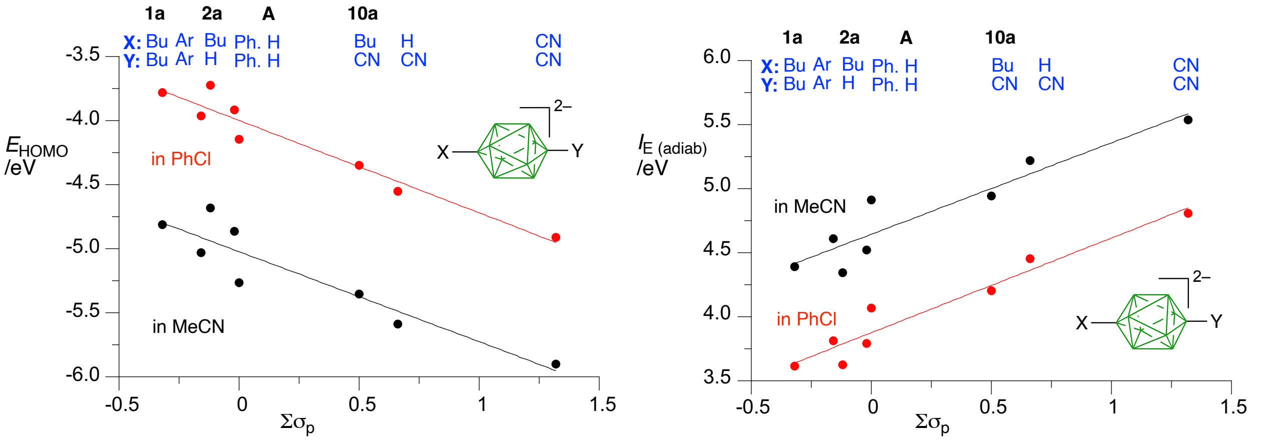

Figure S82. Correlation of HOMO energy level for derivatives of **A** in PhCl (left) and MeCN (right) dielectric medium with the sum of Hammett substituent parameters $\Sigma\sigma_p$. Best fit functions:
Left: E_{HOMO} (PhCl) = -4.00(4) – 0.72(8)×$\Sigma\sigma_p$, r^2 = 0.94; E_{HOMO} (MeCN) = -5.02(7) – 0.70(12)×$\Sigma\sigma_p$, r^2 = 0.86.
Right: $I_{E(adiab)}$ (PhCl) = 3.88(5) + 0.74(8)×$\Sigma\sigma_p$, r^2 = 0.93; $I_{E(adiab)}$ (MeCN) = 4.64(6) + 0.71(11)×$\Sigma\sigma_p$, r^2 = 0.88.

The correctness of DFT calculations was demonstrated with a correlation of E_{HOMO} and adiabatic ionisation energy, $I_{E(adiab)}$, in Figure S83.

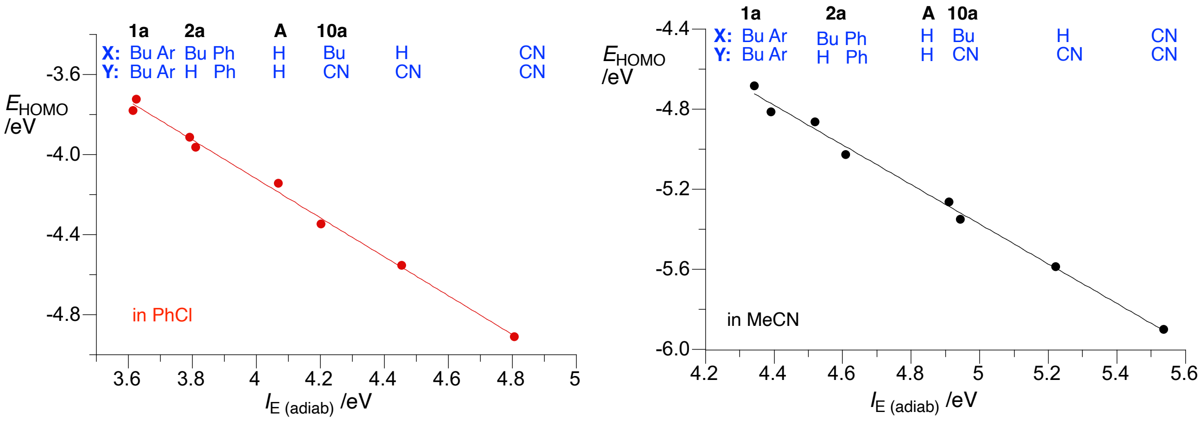

Figure S83. Correlation of HOMO energy level for derivatives of **A** in PhCl (left) and MeCN (right) dielectric medium with adiabatic ionisation energy (see text). Best fit functions: E_{HOMO} (PhCl) = -0.12(1) – $I_{E(adiab)}$, r^2 = 0.994; E_{HOMO} (MeCN) = -0.38(1) – $I_{E(adiab)}$, r^2 = 0.994.

Finally, adiabatic ionisation energy, $I_{E(adiab)}$, (and also E_{HOMO}, main text) shows an excellent correlation with experimental oxidation potentials (Figure S84).

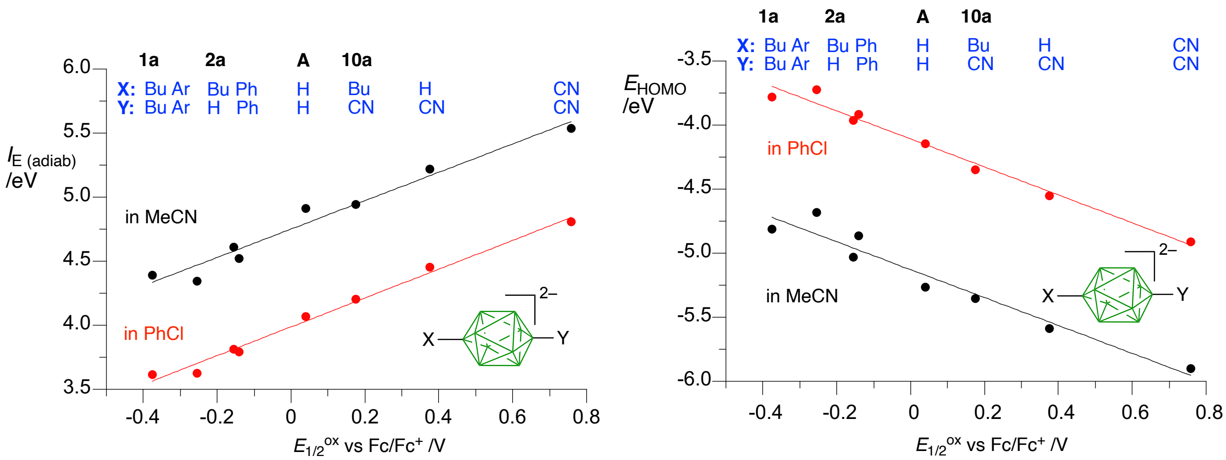

Figure S84. Correlation of experimental oxidation potential $E_{1/2}^{ox}$ of derivatives of **A** with DFT-derived adiabatic ionisation energy I_E (adiab) (left) and energy of the HOMO, E_{HOMO}, (right) calculated in PhCl (red) and MeCN (black) dielectric medium. Best fit functions: $I_{E(adiab)}$ (PhCl) = 3.99(2) + 1.12(5)×$E_{1/2}^{ox}$, r^2 = 0.988; $I_{E(adiab)}$ (MeCN) = 4.75(3) + 1.11(9)×$E_{1/2}^{ox}$, r^2 = 0.963; E_{HOMO} (PhCl) = -4.11(2) - 1.09(6)×$E_{1/2}^{ox}$, r^2 = 0.978; E_{HOMO} (MeCN) = -5.13(4) - 1.09(11)×$E_{1/2}^{ox}$, r^2 = 0.944.

e) FMO energies for model cations and anions

To analyse the intermolecular charge transfer observed in CH_2Cl_2 solutions, energies of the FMOs were calculated at the CAM-B3LYP/Def2TZVP level of theory using SCRF(PCM, Solvent=CH2Cl2) keyword as a single point at the geometry determined with the B3LYP/Def2TZVP method in PhCl dielectric medium. The results are shown in Table S7.

Table S7. FMO energies for model anions and cations obtained in CH_2Cl_2 dielectric medium.[a]

Ion	E_{HOMO} [b] /eV	E_{LUMO} [b] /eV
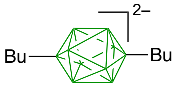 **2a**, C1 symmetry	-5.597	2.437
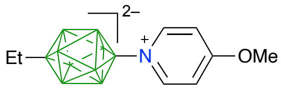 **8b'**, C1 symmetry	-6.381	-0.272
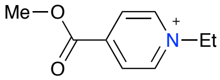 **PyrCOOC$_1$**, [c]	-10.692	-2.513
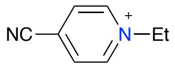 **PyrCN'**, [c]	-10.991	-2.804
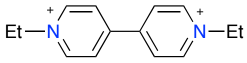 **Q2**, [c]	-11.015	-3.230

[a] Obtained with CAM-B3LYP/def2TZVP//B3LYP/def2TZVP method in CH_2Cl_2 dielectric medium for model compounds. [b] FMO energy. [c] Ref. [6]

The wavelength of the peak and edge absorption of the ion pairs in CH_2Cl_2 was predicted using the previously determined correlations for similar ion pairs using the calculated differences of HOMO and LUMO energies, ΔE_{FMO} (equations S1 and S2).[6] The results are collected in Table S8.

CT band peak:	$E_{peak}(DCM) = 0.783(7)\times\Delta E_{FMO}$	eq S1
CT band onset:	$E_{edge}(DCM) = -0.74(11) + 0.805(30)\times\Delta E_{FMO}$	eq S2

Table S8. Difference in energy $\Delta E_{FMO} = E_{LUMO}–E_{HOMO}$ (eV) of the LUMO of the cation (E_{LUMO}) and the HOMO of the anion (E_{HOMO}) in CH_2Cl_2 dielectric medium.[a]

anion	cation	ΔE_{FMO} /eV	λ_{max} [b] /nm	λ_{edge} [c] /nm
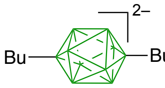 **2a**	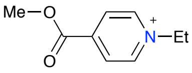	3.084	514	711
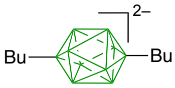 **2a**	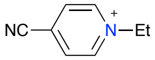	2.793	570	820
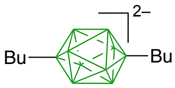 **2a**	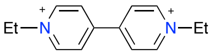	2.367	670	1060
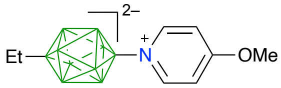	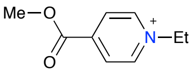	3.868	410	520

[a] FMO energies obtained with CAM-B3LYP/def2TZVP//B3LYP/def2TZVP method in CH_2Cl_2 dielectric medium. See Table S2. [b] Determined from correlation: $E_{peak}(DCM) = 0.783(7)\times\Delta E_{FMO}$; accuracy ±18 nm. Ref.[6]. [c] Determined from empirical correlation: $E_{edge}(DCM) = -0.74(11) + 0.805(30)\times \Delta E_{FMO}$; accuracy ±26 nm. Ref.[6].

f) models of the Fe(II) complexes

DFT analysis of the Fe(II) complex **9a** was performed for its model **9a'** in which the bidentate dppe ligand was replaced with two PH_3 ligands. The equilibrium structure optimized at the B3LYP/Def2SVP method without symmetry constraints in CH_2Cl_2 dielectric medium is shown in Figure S85. The solvent effects were implemented with the COSMO method[25] using the SCRF(COSMO, Solvent=CH2CL2) keyword. The HOMO is mainly localized on the $\{B_{10}\}$ cluster (Figure S85). For comparison purposes similar calculations were performed for models of structurally analogous compounds recently studied experimentally. The natural charges for all compounds were analysed by the NBO method. Results are shown in Table S9.

A correlation of the calculated E_{HOMO} for models and experimental $E_{1/2}^{0/+1}$ is shown in Figure S86.

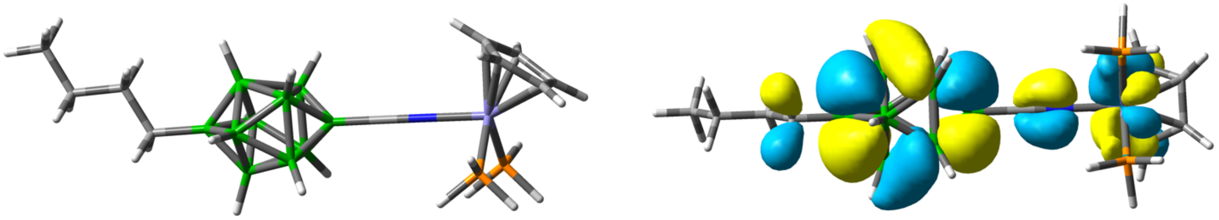

Figure S85. Left: Molecular model of equilibrium geometry for model complex **9a'** obtained at the B3LYP/Def2SVP level of theory in CH_2Cl_2 dielectric medium. Right: the HOMO of **9a'**. MO isovalue =0.020.

Table S9. Energy of the HOMO, E_{HOMO}, stretching frequency for the CN group, ν_{CN}, and natural charge, q_N, of the nitrile N atom, and experimental oxidation potential, $E_{1/2}^{ox}$, for models and oxidation potentials for **9a** and related complexes.[a]

complex	E_{HOMO} /eV	ν_{CN} /cm^{-1}	q_N /e	$E_{1/2}^{ox\ b}$ /V
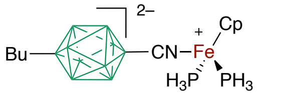 **9a'**	-5.081	2284.8	-0.394	-0.054
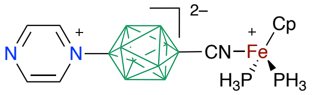 *c*	-5.685	2300.2	-0.368	0.101
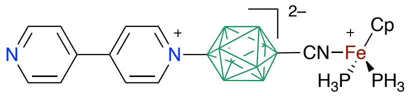 *c]*	-5.657	2298.8	-0.372	0.080
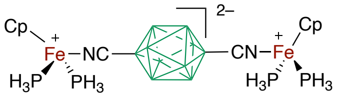 *c,d*	-5.510	2297.6	-0.287	0.056

[a] Obtained with B3LYP/Def2SVP in CH_2Cl_2 dielectric medium. [b] Potential relative to the Fc/Fc$^+$ couple. [c] Ref.[4]. [d] Ref. [26].

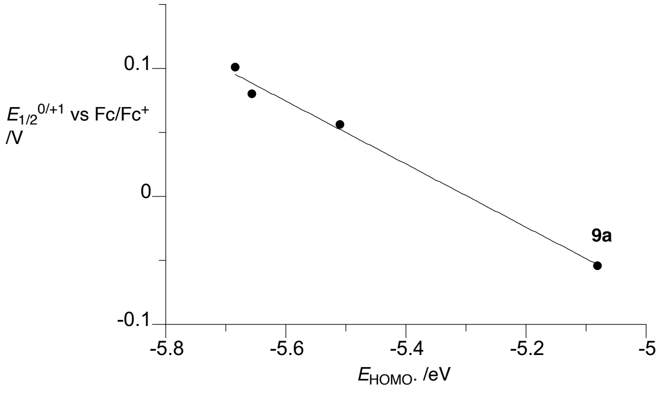

Figure S86. Correlation of DFT HOMO energy for model compounds with experimental $E_{1/2}^{0/+1}$ for **9a** and related Fe(II) complexes. Best fitting function: E_{HOMO} for models and experimental $E_{1/2}^{0/+1}$ = 0.246(15)×E_{HOMO}–1.30(9), r^2 = 0.992.

8. Archive for DFT results.

A


```
1\1\GINC-LOCALHOST\FOpt\RB3LYP\def2TZVP\B10H10(2-)\PIOTR\10-Jun-2020\0
 \\#P B3LYP/Def2TZVP FOpt(tight) geom(noangle, nodistance) fcheck #P SC
 RF(Solvent=C6H5Cl)\\B10H10 anion, D4d sym\\-2,1\B,-0.0000000006,1.2957
 240704,-0.7581610836\B,0.0000000006,-1.2957240703,-0.7581610836\B,-1.2
 957240703,-0.0000000006,-0.7581610836\B,1.2957240703,0.0000000006,-0.7
 581610836\B,0.915966411,0.9159664119,0.758390132\B,-0.9159664119,0.915
 9664111,0.758390132\B,-0.915966411,-0.9159664118,0.758390132\B,0.91596
 64119,-0.915966411,0.758390132\B,0.,0.,1.8524476948\B,0.,0.,-1.8531518
 459\H,-0.0000000011,2.4276697171,-1.1607416045\H,0.0000000011,-2.42766
 97171,-1.1607416045\H,-2.4276697171,-0.0000000011,-1.1607416045\H,2.42
 76697171,0.0000000011,-1.1607416045\H,1.7165238536,1.7165238552,1.1608
 451813\H,-1.7165238552,1.7165238536,1.1608451813\H,-1.7165238536,-1.71
 65238551,1.1608451813\H,1.7165238552,-1.7165238536,1.1608451813\H,0.,0
 .,-3.049567886\H,0.,0.,3.0489415151\\Version=ES64L-G09RevD.01\State=1-
 A1\HF=-254.9669623\RMSD=7.342e-09\RMSF=2.286e-06\Dipole=0.,0.,0.004994
 4\Quadrupole=2.1626917,2.1626917,-4.3253833,0.,0.,0.\PG=C04V [C4(H1B1B
 1H1),2SGV(B2H2),2SGD(B2H2)]\\
```


A(rad-anion)


```
1\1\GINC-LOCALHOST\FOpt\UB3LYP\def2TZVP\B10H10(1-,2)\PIOTR\25-Oct-2025
 \0\\#P UB3LYP/Def2TZVP FOpt geom(noangle, nodistance) fcheck #P SCRF(S
 olvent=C6H5Cl)\\B10H10 rad anion, D4d sym\\-1,2\B,-0.0000000009,1.3112
 296958,-0.7724830689\B,0.0000000006,-1.3112296962,-0.7724830691\B,-1.3
 082509865,-0.000000001,-0.7094462491\B,1.3082509862,0.0000000006,-0.70
 94462496\B,0.8838858053,0.9780325427,0.7336306344\B,-0.8838858061,0.97
 80325416,0.7336306347\B,-0.883885805,-0.9780325433,0.7336306346\B,0.88
 38858064,-0.9780325422,0.7336306342\B,0.0000000004,-0.0000000004,1.858
 5797719\B,-0.0000000003,-0.0000000001,-1.8603590018\H,-0.0000000016,2.
 4230710294,-1.2002860941\H,0.0000000012,-2.4230710297,-1.2002860944\H,
 -2.4118307525,-0.0000000016,-1.1558537442\H,2.4118307521,0.0000000012,
 -1.1558537451\H,1.6788975916,1.7394644338,1.186519854\H,-1.6788975931,
 1.7394644318,1.1865198546\H,-1.6788975911,-1.7394644344,1.1865198544\H
 ,1.6788975936,-1.7394644325,1.1865198537\H,-0.0000000006,0.,-3.0444636
 572\H,0.0000000006,-0.0000000005,3.041779234\\Version=ES64L-G16RevC.01
 \State=2-B1\HF=-254.8179017\S2=0.757207\S2-1=0.\S2A=0.750033\RMSD=5.05
 7e-09\RMSF=1.036e-04\Dipole=0.,0.,0.1392914\Quadrupole=1.3824995,-0.19
 01927,-1.1923069,0.,0.,0.\PG=C02V [C2(H1B1B1H1),SGV(B2H2),SGV'(B2H2),X
 (B4H4)]\\@
```


1a


```
1\1\GINC-LOCALHOST\FOpt\RB3LYP\def2TZVP\C4H18B10(2-)\PIOTR\24-Apr-2025
 \0\\#P B3LYP/Def2TZVP FOpt(tight) geom(noangle, nodistance) fcheck #P
 SCRF(Solvent=C6H5Cl) freq\\H-B10-Bu Cs\\-2,1\B,-1.0933965966,0.7744403
 114,0.037409266\B,1.3974042335,1.4527600953,-0.0203932202\B,0.12615423
 85,1.0988983381,-1.2835207382\B,0.1776685142,1.1292595313,1.2996983713
 \B,-1.1072432206,2.3482286879,0.9362086812\B,-1.1454444398,2.326661722
 1,-0.8983024498\B,0.6215075248,2.8077269104,-0.9387914541\B,0.65885552
 56,2.8300848486,0.8929290081\B,-0.5306415945,3.6341318738,-0.009249266
```

7\B,0.4441619064,0.0432833177,0.0142952207\H,-2.0808300207,0.091195339
7,0.0639991946\H,2.5956553108,1.3457897825,-0.0433851634\H,0.217740609
9,0.6846834068,-2.4094692978\H,0.3121450359,0.7411978882,2.4304108776\
H,-1.9703301403,2.5392084331,1.7511223818\H,-2.0410782118,2.4964710402
,-1.681936656\H,1.2737304772,3.3942505625,-1.7603630674\H,1.3414925131
,3.4377394274,1.6738551193\C,-0.1600698396,-2.5707017957,0.0003197091\
H,-0.8164838538,-2.44602897,0.8697746021\H,-0.8028418173,-2.4312879152
,-0.8772340013\C,0.9288886667,-1.4894568367,0.0180477501\H,1.564455340
1,-1.6699931013,0.8969597601\H,1.5867323397,-1.6642548449,-0.845512744
1\C,0.3797318301,-4.0036288054,-0.0075149664\H,1.027841338,-4.13721265
19,-0.881613192\H,1.0231710949,-4.1486447104,0.8682634232\C,-0.7106990
518,-5.075141777,-0.0173785328\H,-0.2883562582,-6.0836428166,-0.024175
2297\H,-1.3531057462,-4.990913232,0.8636112198\H,-1.3501915795,-4.9779
027142,-0.8991450767\H,-0.8496254088,4.7880669443,-0.0156743285\\Versi
on=ES64L-G16RevC.01\State=1-A\HF=-412.2826863\RMSD=2.749e-09\RMSF=1.06
6e-05\Dipole=0.2320769,-6.7983964,-0.0030846\Quadrupole=11.122778,-23.
7983141,12.6755361,1.9989593,-0.0189104,-0.0065309\PG=C01 [X(C4H18B10)
]\\@

1a (rad-anion)

1\1\GINC-LOCALHOST\FOpt\UB3LYP\def2TZVP\C4H18B10(1-,2)\PIOTR\25-Oct-20
25\0\\#P UB3LYP/Def2TZVP FOpt geom(noangle, nodistance) fcheck #P SCRF
(Solvent=C6H5Cl)\\H-B10-Bu starts at dianion geom, C1\\-1,2\B,-1.13417
61282,0.8095356775,-0.2708416149\B,1.3996732081,1.4408436656,0.0256515
715\B,0.3696424793,1.3248396513,-1.397328058\B,-0.1041364431,0.9302891
71,1.1569919779\B,-1.2593477692,2.2321217961,0.7883831846\B,-0.9959220
689,2.427720569,-1.0297932361\B,0.7641300437,2.9245920304,-0.722478577
6\B,0.5358410168,2.6023080712,1.0827493926\B,-0.5185574801,3.618447348
2,0.1444311999\B,0.4188542309,0.0183892036,-0.2369319654\H,-2.06704794
26,0.1140796091,-0.5245764004\H,2.5640857776,1.2468740008,0.1859313912
\H,0.6051521155,1.0212574915,-2.5249757318\H,-0.102425469,0.3381236417
,2.1908503042\H,-2.2252622348,2.3477078342,1.4773264381\H,-1.752666080
6,2.7628740474,-1.886599658\H,1.51639206,3.6266139211,-1.3245216366\H,
1.076898271,3.0876839961,2.0265270464\C,-0.1403099858,-2.5492709273,0.
1046057053\H,-0.3686701146,-2.3667934915,1.160291815\H,-1.0902744812,-
2.4416864582,-0.429500489\C,0.8488941791,-1.4875906699,-0.3946143361\H
,1.807711075,-1.6246455627,0.1269054934\H,1.0837856853,-1.6839913414,-
1.4498235289\C,0.362018666,-3.9851041768,-0.0567847862\H,0.5901359039,
-4.1687491864,-1.1127174812\H,1.3101894177,-4.0962683528,0.4811294408\
C,-0.6304080001,-5.0358022646,0.4391237059\H,-0.2420100326,-6.04862025
33,0.3087885982\H,-0.8509145608,-4.8988121533,1.501131647\H,-1.5769626
696,-4.970664421,-0.1040755099\H,-0.8173139489,4.7589658234,0.26798929
8\\Version=ES64L-G16RevC.01\State=2-A\HF=-412.1410674\S2=0.756425\S2-1
=0.\S2A=0.750027\RMSD=5.516e-09\RMSF=5.755e-06\Dipole=0.26606,-3.92553
2,-0.0156795\Quadrupole=5.5747634,-12.1694492,6.5946858,0.4130263,0.93
02684,0.3981217\PG=C01 [X(C4H18B10)]\\@

2a

1\1\GINC-LOCALHOST\FOpt\RB3LYP\def2TZVP\C8H26B10(2-)\PIOTR\25-Apr-2025
\0\\#P B3LYP/Def2TZVP FOpt geom(check, noangle, nodistance) fcheck #P
SCRF(Solvent=C6H5Cl) guess=check\\Bu-B10-Bu C1\\-2,1\B,-1.0290010587,0
.9552880235,-0.258454516\B,-0.4438794599,-1.2868118928,0.8796713228\B,
-0.614766579,-0.7208299615,-0.8515178826\B,-0.8551924091,0.3905098815,
1.4682536978\B,0.4454604521,1.385319624,0.7037694709\B,0.6150499358,0.
5976877343,-0.9387078417\B,1.0297899588,-0.9865859122,-0.1318664023\B,
0.857371021,-0.2008531043,1.5063313641\B,1.8125313733,0.471321915,0.26
61785461\B,-1.8114366844,-0.4379169221,0.327476585\H,-1.6685594088,1.8

46936294,-0.7478503294\H,-0.5903332322,-2.3697969035,1.3838197503\H,-0
.9110968805,-1.3129994935,-1.8564232743\H,-1.3607593506,0.7743225677,2
.490714886\H,0.5922424621,2.5248077958,1.0622034282\H,0.9101569861,1.0
534240774,-2.0127301736\H,1.6691474664,-1.9345313015,-0.5009403958\H,1
.3639261702,-0.4476469391,2.5697219556\C,-4.3664259494,0.0827939995,-0
.2680878029\H,-4.2805907132,1.0715675298,0.1981542643\H,-4.1042819403,
0.232202801,-1.3224855128\C,4.367281175,-0.1226139767,-0.2576139647\H,
4.1045383917,-0.4080799994,-1.283413356\H,4.2817537745,-1.0422616185,0
.3334585619\C,-3.3580100409,-0.8759038275,0.379442939\H,-3.6632076845,
-1.0264045748,1.4256038372\H,-3.4782370617,-1.8614448185,-0.0940939682
\C,3.3591628839,0.9122320288,0.2601888667\H,3.6646686874,1.1974594151,
1.2778088502\H,3.4793018892,1.827853473,-0.3374698909\C,-5.8236908958,
-0.379150776,-0.1806667201\H,-5.9166798105,-1.3646852239,-0.6520578715
\H,-6.0898564278,-0.5239242405,0.872987648\C,-6.8189039361,0.586289382
6,-0.8250808969\H,-7.8474190825,0.2249583939,-0.7407730402\H,-6.773928
2144,1.5712156851,-0.3518872879\H,-6.6025758594,0.7245287844,-1.888121
4782\C,5.824578497,0.346739243,-0.2319000813\H,6.0912960754,0.62740260
44,0.7938099988\H,5.9172680958,1.2625351729,-0.8275910763\C,6.81950287
23,-0.6943634902,-0.7457817907\H,7.8480720313,-0.3252594374,-0.7095393
08\H,6.7746306997,-1.6094305889,-0.1485817683\H,6.602762469,-0.9695146
613,-1.7817800147\\Version=ES64L-G16RevC.01\State=1-A\HF=-569.597375\R
MSD=5.800e-09\RMSF=8.341e-06\Dipole=-0.0004087,-0.0652072,-0.9968543\Q
uadrupole=-12.7774006,5.8924387,6.8849619,-0.8397,0.0614277,0.0655302\
PG=C01 [X(C8H26B10)]\\@

2a (rad-anion)

1\1\GINC-LOCALHOST\FOpt\UB3LYP\def2TZVP\C8H26B10(1-,2)\PIOTR\24-Oct-20
25\0\\#P UB3LYP/Def2TZVP FOpt geom(noangle, nodistance) fcheck #P SCRF
(Solvent=C6H5Cl)\\Bu-B10-Bu C1 rad ion\\-1,2\B,-1.055590315,0.95629632
83,-0.4191752713\B,-0.4052964035,-1.3458719506,0.6471286572\B,-0.55119
99574,-0.7775959111,-1.0424108249\B,-0.8316017873,0.4094914246,1.27126
92978\B,0.4064457697,1.4137671716,0.4658921661\B,0.551208256,0.6311017
66,-1.134805461\B,1.0559948544,-1.0081492989,-0.2912926545\B,0.8343604
834,-0.2462881211,1.3131149832\B,1.8128279672,0.4965639319,0.067892240
1\B,-1.811687748,-0.4883994887,0.1340603961\H,-1.7405436051,1.79850206
05,-0.910049986\H,-0.5611381137,-2.4142285877,1.1540147121\H,-0.881833
8893,-1.3593889479,-2.028886045\H,-1.3763756086,0.7463880343,2.2766152
468\H,0.5629518735,2.5388654508,0.8295243348\H,0.8810912409,1.07940441
45,-2.1889070997\H,1.7407277187,-1.9068180779,-0.6693696454\H,1.380215
0727,-0.449325341,2.3532164021\C,-4.3784961444,0.1190506252,-0.1600644
71\H,-4.2568413906,0.9846266541,0.5006044772\H,-4.1993863986,0.4928141
131,-1.1740958724\C,4.3793066402,-0.1446099167,-0.1456759035\H,4.19966
20373,-0.6477571209,-1.1020262934\H,4.257813048,-0.9162928298,0.622592
803\C,-3.3237263403,-0.9403135939,0.1863787846\H,-3.5323164313,-1.3255
588338,1.195093424\H,-3.4609318801,-1.8082875805,-0.472704781\C,3.3248
726198,0.9512473867,0.0596354204\H,3.5340019995,1.4650006057,1.0092588
844\H,3.4619247003,1.7255241113,-0.7073453523\C,-5.8189479869,-0.38650
51016,-0.0619709148\H,-5.9412872417,-1.2517063044,-0.7233065185\H,-6.0
015079688,-0.75475689,0.9538916629\C,-6.8620770423,0.6733715998,-0.413
6099022\H,-7.8782644129,0.2800274892,-0.3333423305\H,-6.7865113637,1.5
365871598,0.2531112283\H,-6.7260753646,1.0355113873,-1.4362085433\C,5.
8198801345,0.3691257604,-0.1151998531\H,6.0030183107,0.8669019862,0.84
37531732\H,5.9420038529,1.1404782454,-0.8839663192\C,6.862660312,-0.72
78382977,-0.3258753551\H,7.8789477426,-0.3276012559,-0.2981601406\H,6.
7872945079,-1.4965507461,0.4479254205\H,6.7260889402,-1.2204167479,-1.
2923448482\\Version=ES64L-G16RevC.01\State=2-A\HF=-569.4623225\S2=0.75
6872\S2-1=0.\S2A=0.75003\RMSD=8.098e-09\RMSF=3.555e-05\Dipole=0.002233

8,-0.0132165,-0.2047368\Quadrupole=-3.8380131,0.4145847,3.4234284,1.46
09384,-0.0938889,0.2028967\PG=C01 [X(C8H26B10)]\\@

3

1\1\GINC-LOCALHOST\FOpt\RB3LYP\def2TZVP\B10H9I1(2-)\PIOTR\08-Jun-2024\
0\\#P B3LYP/Def2TZVP FOpt(tight) geom(noangle, nodistance) fcheck freq
\\B10H9-1-I, D4d sym optimized in vaccum\\-2,1\B,0.,1.3086827097,0.054
7940256\B,0.,-1.3086827097,0.0547940256\B,1.3086827097,0.,0.0547940256
\B,-1.3086827097,0.,0.0547940256\B,-0.9201686739,0.9201686739,-1.46024
77239\B,0.9201686739,0.9201686739,-1.4602477239\B,0.9201686739,-0.9201
686739,-1.4602477239\B,-0.9201686739,-0.9201686739,-1.4602477239\B,0.,
0.,-2.5496367419\B,0.,0.,1.1093307061\H,0.,2.4323551165,0.4739279627\H
,0.,-2.4323551165,0.4739279627\H,2.4323551165,0.,0.4739279627\H,-2.432
3551165,0.,0.4739279627\H,-1.7199798021,1.7199798021,-1.8677149932\H,1
.7199798021,1.7199798021,-1.8677149932\H,1.7199798021,-1.7199798021,-1
.8677149932\H,-1.7199798021,-1.7199798021,-1.8677149932\H,0.,0.,-3.748
20163\I,0.,0.,3.3644584996\\Version=ES64L-G16RevC.01\State=1-A1\HF=-55
2.0056935\RMSD=1.894e-09\RMSF=6.720e-06\Dipole=0.,0.,4.8214586\Quadrup
ole=13.1661765,13.1661765,-26.3323531,0.,0.,0.\PG=C04V [C4(H1B1B1I1),2
SGV(B2H2),2SGD(B2H2)]\\@

8b′

1\1\GINC-LOCALHOST\FOpt\RB3LYP\def2TZVP\C8H20B10N1O1(1-)\PIOTR\26-Apr-
2025\0\\#P B3LYP/Def2TZVP FOpt(tight) geom(noangle, nodistance) fcheck
#P SCRF(Solvent=C6H5Cl)\\Et-B10-PyrOMe, C1\\-1,1\B,0.3396798339,2.981
004349,0.0163654368\B,2.5380953251,1.6093154626,-0.0113551667\B,1.4476
557016,2.2845429675,1.2981107254\B,1.4261755122,2.3025521275,-1.292353
5131\B,-0.1509164701,1.5113724307,-0.9111154258\B,-0.1348676265,1.4977
091395,0.9310422608\B,1.4346633817,0.5193454769,0.9107691094\B,1.41866
14977,0.5326931561,-0.9304341296\B,0.0827311706,0.1184050165,-0.001633
0746\B,2.0246231533,3.2314310513,0.0050549339\C,-2.8265036846,-2.30481
92114,0.0948132147\C,-0.7962611906,-3.5690928252,-0.1051757974\C,-2.07
37494663,-1.159347718,0.0921714118\C,-0.1097262134,-2.3741211259,-0.10
05504428\C,-2.1904061759,-3.548933844,-0.0052826926\H,-0.3989573699,3.
9229570613,0.0293871507\H,3.7124969363,1.3689211716,-0.0228228786\H,1.
6770208665,2.6272029577,2.4237226883\H,1.6386949965,2.6626790663,-2.41
56333064\H,-1.0566992891,1.5624076113,-1.6934507274\H,-1.0266818878,1.
5399084371,1.7300621009\H,1.8770868431,-0.2742739868,1.691766692\H,1.8
494873439,-0.2474878054,-1.7313319508\H,-3.9025946989,-2.250772269,0.1
735016895\H,-0.237385046,-4.4879162897,-0.1859460366\H,-2.5200098116,-
0.1798494154,0.1680562375\H,0.9663058967,-2.3395368003,-0.1768919198\O
,-2.9764194709,-4.631752813,0.0026401392\C,-2.3769217642,-5.9290051231
,-0.0984347989\H,-3.2017627266,-6.6345780881,-0.0699032397\H,-1.835419
6736,-6.0333649639,-1.0399637418\H,-1.7060311915,-6.1126112035,0.74225
98355\C,2.1197522964,5.8847402194,0.1656959556\H,2.7762566417,6.762225
783,0.1671133485\H,1.3986957534,6.014022543,-0.6457541234\H,1.55148274
97,5.8976061842,1.0997159187\C,2.9022893918,4.572517949,0.0100933956\H
,3.4903839151,4.6301893121,-0.9152579606\H,3.6461799034,4.5184051222,0
.8156811596\N,-0.7237734632,-1.1801294628,-0.0038661677\\Version=ES64L
-G16RevC.01\State=1-A\HF=-695.9090811\RMSD=4.607e-09\RMSF=5.119e-06\Di
pole=-4.6256384,-8.8047513,-0.0729119\Quadrupole=-2.9372115,5.266881,-
2.3296695,-7.3463122,-0.279818,0.8378697\PG=C01 [X(C8H20B10N1O1)]\\@

10a

1\1\GINC-LOCALHOST\FOpt\RB3LYP\def2TZVP\C5H17B10N1(2-)\PIOTR\25-Apr-20
25\0\\#P B3LYP/Def2TZVP FOpt(tight) geom(noangle, nodistance) fcheck #
P SCRF(Solvent=C6H5Cl) freq\\NC-B10-Bu C1\\-2,1\B,-0.9456320684,0.1637

088103,0.0314044653\B,1.5916489296,0.665091998,0.00232403\B,0.31189436
94,0.3953835532,-1.2761974229\B,0.3341606143,0.4357519356,1.3110426336
\B,-0.8596196444,1.7310101259,0.9297468809\B,-0.8763201533,1.703036199
2,-0.9149382275\B,0.9298506378,2.0597497073,-0.9357794155\B,0.94695341
27,2.0881921034,0.9068793622\B,-0.1742960865,2.9554204743,-0.017633168
3\B,0.5386282161,-0.6723484059,0.0326967076\H,-1.9793632481,-0.4433987
028,0.0495363759\H,2.7760305262,0.4692471629,-0.0066934763\H,0.3872001
128,-0.0323824202,-2.3952502602\H,0.4276024149,0.0426251627,2.44134794
97\H,-1.7130049641,2.0013941412,1.7251797134\H,-1.7434951123,1.9493940
653,-1.7033927603\H,1.6224868794,2.6072489811,-1.74466149\H,1.65251942
4,2.6610841349,1.6863628035\C,-0.259722532,-3.2238157049,0.0043655713\
H,-0.9179471279,-3.053755906,0.8644060702\H,-0.8740264947,-3.021953223
3,-0.8809742321\C,0.9106878373,-2.2329152948,0.0506561194\H,1.50891558
04,-2.4565193104,0.945454367\H,1.5749525482,-2.4553436202,-0.796465653
7\C,0.159740589,-4.6959070753,-0.0102768938\H,0.807824408,-4.875578015
8,-0.8759472924\H,0.7737367321,-4.9027422855,0.8740371294\C,-1.0179854
738,-5.6697489871,-0.0484277864\H,-0.6834875235,-6.7104774906,-0.06021
11556\H,-1.6646898157,-5.5391224377,0.8236897856\H,-1.6326435286,-5.50
87251956,-0.9384639432\C,-0.4760276771,4.4559581666,-0.0355428765\N,-0
.7024564318,5.5934479341,-0.0486389004\\Version=ES64L-G16RevC.01\State
=1-A\HF=-504.6102409\RMSD=1.096e-09\RMSF=1.441e-06\Dipole=-0.0486618,-
6.9498411,0.0041909\Quadrupole=19.2997567,-40.9458599,21.6461032,5.158
3516,-0.1081413,0.3438727\PG=C01 [X(C5H17B10N1)]\\@

10a (rad-anion)

1\1\GINC-LOCALHOST\SP\UB3LYP\def2TZVP\C5H17B10N1(1-,2)\PIOTR\26-Oct-20
25\0\\#P UB3LYP/Def2TZVP SCF=tight geom(check, noangle, nodistance) fc
heck #P SCRF(Solvent=CH3CN) guess=check\\NC-B10-Bu C1 in MeCN, rad ani
on at the dianion geom\\-1,2\B,0,0.833581094,0.8840456233,-0.754924982
7\B,0,1.4374674917,-1.2005367846,0.732357426\B,0,1.381397575,-0.969661
0692,-1.0149051079\B,0,0.8910032322,0.653772544,0.9966505366\B,0,2.210
6801668,1.5180602281,0.1746341884\B,0,2.4711374335,0.42105088,-1.30511
66234\B,0,2.9517374034,-0.9806420904,-0.176597123\B,0,2.555062633,0.07
52410595,1.3033210928\B,0,3.5954046814,0.566646212,0.0064816983\B,0,0.
0273404095,-0.4798985803,-0.0176905547\H,0,0.1447906781,1.5644310613,-
1.4456812693\H,0,1.2199527881,-2.1474508526,1.4184585169\H,0,1.1110251
938,-1.7101306216,-1.9056226805\H,0,0.2500736161,1.1229636434,1.882239
4464\H,0,2.3177339442,2.6926600866,0.3260295057\H,0,2.8412325983,0.685
3311405,-2.4030904798\H,0,3.6792792121,-1.9097144049,-0.3228016522\H,0
,2.9976777045,0.0500727136,2.4059677184\C,0,-2.5415642322,0.151041413,
-0.0153657607\H,0,-2.3839798609,0.8140720159,0.8420026305\H,0,-2.41816
77221,0.7773294687,-0.9050789657\C,0,-1.4705979472,-0.9480219592,-0.01
32192346\H,0,-1.6226519307,-1.5921942232,0.8653363761\H,0,-1.636501871
9,-1.6164740994,-0.8694277458\C,0,-3.9733714797,-0.3854034322,0.022975
3731\H,0,-4.1312384237,-1.0484011111,-0.8349520878\H,0,-4.0989833283,-
1.0093143098,0.9148738355\C,0,-5.0336366058,0.7146644954,0.0178959199\
H,0,-6.0433953358,0.2988000242,0.0462445027\H,0,-4.9212972364,1.373670
9264,0.8829096387\H,0,-4.9544713498,1.3338108881,-0.8796386793\C,0,5.0
586175701,0.9978075976,0.0173341751\N,0,6.1679930788,1.3238322569,0.02
55921964\\Version=ES64L-G16RevC.01\State=2-A\HF=-504.4640836\S2=0.7561
4\S2-1=0.\S2A=0.750028\RMSD=7.173e-09\Dipole=-5.2048009,-0.7489688,-0.
0055365\Quadrupole=-27.9363803,12.2366819,15.6996984,-7.2693166,-0.125
5793,-0.1458312\PG=C01 [X(C5H17B10N1)]\\@

[*closo*-$B_{10}H_9$-1-CN]$^{2-}$

1\1\GINC-LOCALHOST\FOpt\RB3LYP\def2TZVP\C1H9B10N1(2-)\PIOTR\24-Apr-202
5\0\\#P B3LYP/Def2TZVP FOpt(tight) geom(noangle, nodistance) fcheck #P

SCRF(Solvent=C6H5Cl) freq(noraman, readIso)\\B10H9-1-CN, D4d sym\\-2,
1\B,0.,1.3031949629,0.0615703072\B,0.,-1.3031949629,0.0615703072\B,1.3
031949629,0.,0.0615703072\B,-1.3031949629,0.,0.0615703072\B,-0.9173128
534,0.9173128534,-1.4467379797\B,0.9173128534,0.9173128534,-1.44673797
97\B,0.9173128534,-0.9173128534,-1.4467379797\B,-0.9173128534,-0.91731
28534,-1.4467379797\B,0.,0.,-2.5398138382\B,0.,0.,1.1414288696\H,0.,2.
4242256433,0.4816701873\H,0.,-2.4242256433,0.4816701873\H,2.4242256433
,0.,0.4816701873\H,-2.4242256433,0.,0.4816701873\H,-1.7145744027,1.714
5744027,-1.8541085199\H,1.7145744027,1.7145744027,-1.8541085199\H,1.71
45744027,-1.7145744027,-1.8541085199\H,-1.7145744027,-1.7145744027,-1.
8541085199\H,0.,0.,-3.7328883038\C,0.,0.,2.6732078815\N,0.,0.,3.832662
6936\\Version=ES64L-G16RevC.01\State=1-A1\HF=-347.2943312\RMSD=3.275e-
09\RMSF=3.714e-06\Dipole=0.,0.,-0.145441\Quadrupole=10.8855808,10.8855
808,-21.7711617,0.,0.,0.\PG=C04V [C4(H1B1B1C1N1),2SGV(H2B2),2SGD(H2B2)
]\\@

[*closo*-$B_{10}H_9$-1-CN]$^{-\bullet}$

1\1\GINC-LOCALHOST\SP\UB3LYP\def2TZVP\C1H9B10N1(1-,2)\PIOTR\26-Oct-202
5\0\\#P UB3LYP/Def2TZVP SCF=tight geom(check, noangle, nodistance) fch
eck #P SCRF(Solvent=CH3CN) guess=check\\B10H9-1-CN, D4d sym, rad at th
e PhCl geom\\-1,2\B,0,0.0000000001,1.3175714093,0.0185665859\B,0,0.,-1
.3175714093,0.0185665858\B,0,1.316394233,-0.0000000001,0.0870123899\B,
0,-1.3163942329,0.0000000001,0.0870123901\B,0,-0.9790109724,0.88501302
9,-1.4175164021\B,0,0.9790109724,0.8850130289,-1.4175164022\B,0,0.9790
109723,-0.885013029,-1.4175164022\B,0,-0.9790109725,-0.8850130289,-1.4
175164021\B,0,-0.0000000001,0.0000000001,-2.5475918991\B,0,0.000000000
1,-0.0000000001,1.1494316847\H,0,0.0000000002,2.4221528865,0.455329449
9\H,0,-0.0000000001,-2.4221528866,0.4553294498\H,0,2.4209116707,-0.000
0000002,0.5262220633\H,0,-2.4209116706,0.0000000001,0.5262220635\H,0,-
1.7311468111,1.6786214647,-1.8841640459\H,0,1.7311468111,1.6786214645,
-1.8841640461\H,0,1.7311468109,-1.6786214646,-1.8841640462\H,0,-1.7311
468113,-1.6786214644,-1.884164046\H,0,-0.0000000002,0.0000000001,-3.72
8808108\C,0,0.0000000002,-0.0000000001,2.6737303124\N,0,0.0000000002,-
0.0000000001,3.8298720788\\Version=ES64L-G16RevC.01\State=2-B2\HF=-347
.1393183\S2=0.757058\S2-1=0.\S2A=0.750035\RMSD=6.386e-09\Dipole=0.,0.,
-1.251211\Quadrupole=6.7265535,7.9298169,-14.6563704,0.,0.,0.\PG=C02V
[C2(H1B1B1C1N1),SGV(H2B2),SGV'(H2B2),X(H4B4)]\\@

[*closo*-$B_{10}H_9$-1,10-(CN)$_2$]$^{2-}$

1\1\GINC-LOCALHOST\FOpt\RB3LYP\def2TZVP\C2H8B10N2(2-)\PIOTR\30-Nov-202
0\0\\#P B3LYP/Def2TZVP FOpt(tight) geom(noangle, nodistance) fcheck #P
SCRF(Solvent=C6H5Cl) guess=check freq(noraman, readIso)\\B10H01,10-(C
N)2, D4d\\-2,1\B,-0.0000438252,1.3051963072,0.7508880076\B,0.000043824
4,-1.3051963068,0.7508880076\B,1.3051963066,0.000043825,0.7508880076\B
,-1.3051963074,-0.0000438246,0.7508880076\B,-0.9226089021,0.9225812293
,-0.7508037712\B,0.9225812287,0.9226089019,-0.7508037712\B,0.922608901
3,-0.9225812289,-0.7508037712\B,-0.9225812295,-0.9226089015,-0.7508037
712\B,-0.0000000004,0.0000000002,-1.8282020846\B,-0.0000000004,0.00000
00002,1.8276166664\H,0.0000107758,2.4236390263,1.1715472522\H,-0.00001
07766,-2.4236390259,1.1715472522\H,2.4236390257,-0.000010776,1.1715472
522\H,-2.4236390265,0.0000107764,1.1715472522\H,-1.7136397659,1.713672
2452,-1.1711313593\H,1.7136722446,1.7136397657,-1.1711313593\H,1.71363
97651,-1.7136722448,-1.1711313593\H,-1.7136722454,-1.7136397653,-1.171
1313593\C,-0.0000000004,0.0000000002,3.3599257401\N,-0.0000000004,0.00
00000002,4.5183536076\C,-0.0000000004,0.0000000002,-3.3606401374\N,-0.
0000000004,0.0000000002,-4.5190543159\\Version=ES64L-G09RevD.01\State=
1-A\HF=-439.6175758\RMSD=5.357e-09\RMSF=4.188e-06\Dipole=0.,0.,-0.0057

249\Quadrupole=21.4948807,21.4948807,-42.9897613,0.,0.,0.\PG=C04 [C4(N
1C1B1B1C1N1),X(H8B8)]\\@

[*closo*-$B_{10}H_9$-1,10-(CN)$_2$]$^{-•}$

1\1\GINC-LOCALHOST\FOpt\UB3LYP\def2TZVP\C2H8B10N2(1-,2)\PIOTR\25-Oct-2
025\0\\#P UB3LYP/Def2TZVP FOpt(tight) geom(noangle, nodistance) fcheck
#P SCRF(Solvent=C6H5Cl)\\B10H01,10-(CN)2, D4d, rad anion\\-1,2\B,-0.0
000095141,1.3189560213,0.7665313985\B,0.0000095133,-1.3189560209,0.766
5313985\B,1.3196982416,-0.000009171,0.7051411733\B,-1.3196982424,0.000
0091714,0.7051411733\B,-0.8917735687,0.9836547914,-0.7300270764\B,0.89
17804398,0.9836472246,-0.7300169793\B,0.8917735679,-0.9836547909,-0.73
00270764\B,-0.8917804406,-0.9836472241,-0.7300169793\B,-0.0000000004,0
.0000000002,-1.8343252665\B,-0.0000000004,0.0000000002,1.8355732412\H,
-0.0000172335,2.4206108121,1.2090812008\H,0.0000172327,-2.4206108117,1
.2090812007\H,2.4158393049,-0.0000010946,1.1587236076\H,-2.4158393057,
0.000001095,1.1587236076\H,-1.6803375435,1.7400269868,-1.1920790715\H,
1.6803423269,1.7400156439,-1.1920782613\H,1.6803375427,-1.7400269864,-
1.1920790715\H,-1.6803423277,-1.7400156434,-1.1920782614\C,-0.00000000
04,0.0000000002,3.3574680779\N,-0.0000000004,0.0000000002,4.5133043735
\C,-0.0000000004,0.0000000002,-3.353216139\N,-0.0000000004,0.000000000
2,-4.5093562904\\Version=ES64L-G16RevC.01\State=2-B\HF=-439.4387328\S2
=0.7584\S2-1=0.\S2A=0.750056\RMSD=7.523e-09\RMSF=4.146e-07\Dipole=0.,0
.,-0.184361\Quadrupole=16.7947119,15.4979803,-32.2926923,0.0001049,0.,
0.\PG=C02 [C2(N1C1B1B1C1N1),X(H8B8)]\\@

[*closo*-$B_{10}H_9$-1,10-Ph_2]$^{2-}$

1\1\GINC-LOCALHOST\FOpt\RB3LYP\def2TZVP\C12H18B10(2-)\PIOTR\17-Apr-202
5\0\\#P B3LYP/Def2TZVP FOpt=tight geom(noangle, nodistance) fcheck #P
SCRF(Solvent=C6H5Cl) freq(noraman)\\1-(4-FPh)-B10-10-(Ph-4-F), D2, sta
ggered in PhCl\\-2,1\B,0.,0.,1.8576684618\B,-0.4924209998,1.1983433515
,-0.7573262518\B,1.194388709,0.5006987059,-0.7540367625\B,0.4924209998
,-1.1983433515,-0.7573262518\B,-1.194388709,-0.5006987059,-0.754036762
5\B,-1.194388709,0.5006987059,0.7540367625\B,0.4924209998,1.1983433515
,0.7573262518\B,1.194388709,-0.5006987059,0.7540367625\B,-0.4924209998
,-1.1983433515,0.7573262518\B,0.,0.,-1.8576684618\C,0.,0.,3.4356465836
\C,-0.4082638401,1.1271101744,5.5754877473\C,0.4082638401,-1.127110174
4,5.5754877473\C,-0.4059393757,1.1204301861,4.1835113172\C,0.405939375
7,-1.1204301861,4.1835113172\C,0.,0.,6.2841514031\H,-0.927763099,2.235
228545,-1.1767569141\H,2.2364468673,0.9283087714,-1.1683029451\H,0.927
763099,-2.235228545,-1.1767569141\H,-2.2364468673,-0.9283087714,-1.168
3029451\H,-0.7294763581,2.0149012049,6.1100172787\H,0.7294763581,-2.01
49012049,6.1100172787\H,-2.2364468673,0.9283087714,1.1683029451\H,0.92
7763099,2.235228545,1.1767569141\H,2.2364468673,-0.9283087714,1.168302
9451\H,-0.927763099,-2.235228545,1.1767569141\H,-0.7283895443,2.010759
0862,3.6545922421\H,0.7283895443,-2.0107590862,3.6545922421\C,0.,0.,-3
.4356465836\C,-0.4059393757,-1.1204301861,-4.1835113172\C,0.4059393757
,1.1204301861,-4.1835113172\C,-0.4082638401,-1.1271101744,-5.575487747
3\C,0.4082638401,1.1271101744,-5.5754877473\C,0.,0.,-6.2841514031\H,-0
.7283895443,-2.0107590862,-3.6545922421\H,0.7283895443,2.0107590862,-3
.6545922421\H,-0.7294763581,-2.0149012049,-6.1100172787\H,0.7294763581
,2.0149012049,-6.1100172787\H,0.,0.,-7.368041295\H,0.,0.,7.368041295\\
Version=ES64L-G16RevC.01\State=1-A\HF=-717.2621925\RMSD=6.141e-09\RMSF
=2.054e-07\Dipole=0.,0.,0.\Quadrupole=7.0904844,13.8043581,-20.8948425
,0.,0.,0.\PG=D02 [C2(H1C1C1B1.B1C1C1H1),X(C8H16B8)]\\@

[*closo*-$B_{10}H_9$-1,10-Ph_2]$^{-•}$

1\1\GINC-LOCALHOST\FOpt\UB3LYP\def2TZVP\C12H18B10(1-,2)\PIOTR\17-Apr-2

```
025\0\\#P UB3LYP/Def2TZVP FOpt=tight geom(noangle, nodistance) fcheck
 #P SCRF(Solvent=C6H5Cl)\\1-Ph-B10-10-Ph, D2, staggered in PhCl\\-1,2\B
 ,0.,0.,1.8652942137\B,-0.4586348968,1.2281412431,-0.7571410953\B,1.195
 5775908,0.5364480189,-0.719530331\B,0.4586348968,-1.2281412431,-0.7571
 410953\B,-1.1955775908,-0.5364480189,-0.719530331\B,-1.1955775908,0.53
 64480189,0.719530331\B,0.4586348968,1.2281412431,0.7571410953\B,1.1955
 775908,-0.5364480189,0.719530331\B,-0.4586348968,-1.2281412431,0.75714
 10953\B,0.,0.,-1.8652942137\C,0.,0.,3.4173018903\C,-0.1875569119,1.188
 9585552,5.5440523319\C,0.1875569119,-1.1889585552,5.5440523319\C,-0.18
 36560229,1.1853444534,4.1553687208\C,0.1836560229,-1.1853444534,4.1553
 687208\C,0.,0.,6.2453918777\H,-0.8970115513,2.2465513872,-1.1943534736
 \H,2.2232804534,0.9445184922,-1.1618985999\H,0.8970115513,-2.246551387
 2,-1.1943534736\H,-2.2232804534,-0.9445184922,-1.1618985999\H,-0.33420
 96105,2.1175383482,6.0829759152\H,0.3342096105,-2.1175383482,6.0829759
 152\H,-2.2232804534,0.9445184922,1.1618985999\H,0.8970115513,2.2465513
 872,1.1943534736\H,2.2232804534,-0.9445184922,1.1618985999\H,-0.897011
 5513,-2.2465513872,1.1943534736\H,-0.325541574,2.117819952,3.622251147
 4\H,0.325541574,-2.117819952,3.6222511474\C,0.,0.,-3.4173018903\C,-0.1
 836560229,-1.1853444534,-4.1553687208\C,0.1836560229,1.1853444534,-4.1
 553687208\C,-0.1875569119,-1.1889585552,-5.5440523319\C,0.1875569119,1
 .1889585552,-5.5440523319\C,0.,0.,-6.2453918777\H,-0.325541574,-2.1178
 19952,-3.6222511474\H,0.325541574,2.117819952,-3.6222511474\H,-0.33420
 96105,-2.1175383482,-6.0829759152\H,0.3342096105,2.1175383482,-6.08297
 59152\H,0.,0.,-7.3286905146\H,0.,0.,7.3286905146\\Version=ES64L-G16Rev
 C.01\State=2-B3\HF=-717.1220162\S2=0.759305\S2-1=0.\S2A=0.750079\RMSD=
 3.524e-09\RMSF=4.872e-07\Dipole=0.,0.,0.\Quadrupole=-5.8641354,3.25904
 55,2.6050899,0.,0.,0.\PG=D02 [C2(H1C1C1B1.B1C1C1H1),X(C8H16B8)]\\@
```

[*closo*-$B_{10}H_9$-1,10-(C_6H_4-OMe)$_2$]$^{2-}$

```
1\1\GINC-LOCALHOST\FOpt\RB3LYP\def2TZVP\C14H22B10O2(2-)\PIOTR\04-Oct-2
 020\0\\#P B3LYP/Def2TZVP FOpt(tight, RCFC) geom(noangle, nodistance) f
 check #P guess=check SCRF(Solvent=C6H5Cl)\\MeOPh-B10-PhOMe, C2 start a
 t Def2SVP in PhCl\\-2,1\B,-0.7993402102,-1.170257711,0.540449667\B,-0.
 7144522318,1.2238573529,-0.4450114832\B,-0.7370113838,0.5253409293,1.2
 422272671\B,-0.7722373175,-0.4717775039,-1.146738973\B,0.7144522318,-1
 .2238573529,-0.4450114832\B,0.7370113838,-0.5253409293,1.2422272671\B,
 0.7993402102,1.170257711,0.540449667\B,0.7722373175,0.4717775039,-1.14
 6738973\B,1.8578486536,-0.0657688188,0.0477749161\B,-1.8578486536,0.06
 57688188,0.0477749161\C,5.5346490266,-1.3220877873,0.4805884796\C,5.62
 52653627,0.9135960181,-0.3928774688\C,4.1480338322,-1.2588535344,0.478
 3488112\C,4.228666187,0.948663805,-0.3831378496\C,6.2865904359,-0.2315
 484905,0.0423009249\C,3.4357328344,-0.123352786,0.0470303448\C,-3.4357
 328344,0.123352786,0.0470303448\C,-4.1480338322,1.2588535344,0.4783488
 112\C,-4.228666187,-0.948663805,-0.3831378496\C,-5.5346490266,1.322087
 7873,0.4805884796\C,-5.6252653627,-0.9135960181,-0.3928774688\C,-6.286
 5904359,0.2315484905,0.0423009249\H,-1.2531131013,-2.1926443938,0.9766
 691248\H,-1.0945235239,2.2758633785,-0.8811836821\H,-1.1358529305,0.96
 83885302,2.2841268643\H,-1.2018357103,-0.8851149191,-2.1886513007\H,1.
 0945235239,-2.2758633785,-0.8811836821\H,1.1358529305,-0.9683885302,2.
 2841268643\H,1.2531131013,2.1926443938,0.9766691248\H,1.2018357103,0.8
 851149191,-2.1886513007\H,6.053054847,-2.2121654392,0.8186963028\H,6.1
 757979077,1.778045182,-0.7386225025\H,3.5926426703,-2.1239513803,0.823
 533488\H,3.7410855784,1.854303825,-0.7271179443\H,9.4882646608,0.38144
 66564,-0.2566000229\H,8.2601394206,0.9465129792,-1.4132890033\H,8.2908
 707507,1.5980663524,0.2443583782\H,-3.5926426703,2.1239513803,0.823533
 488\O,7.6546405923,-0.380521104,0.076484211\C,8.4540946369,0.703133075
 9,-0.3640754882\H,-3.7410855784,-1.854303825,-0.7271179443\H,-6.053054
```

847,2.2121654392,0.8186963028\H,-6.1757979077,-1.778045182,-0.73862250
25\O,-7.6546405923,0.380521104,0.076484211\C,-8.4540946369,-0.70313307
59,-0.3640754882\H,-9.4882646608,-0.3814466564,-0.2566000229\H,-8.2601
394206,-0.9465129792,-1.4132890033\H,-8.2908707507,-1.5980663524,0.244
3583782\\Version=ES64L-G09RevD.01\State=1-A\HF=-946.4002699\RMSD=6.584
e-09\RMSF=8.639e-07\Dipole=0.,0.,-0.5667747\Quadrupole=-16.0556652,11.
0670448,4.9886204,17.1229318,0.,0.\PG=C02 [X(C14H22B10O2)]\\@

[*closo*-$B_{10}H_9$-1,10-(C_6H_4-OMe)$_2$]$^{-\bullet}$

1\1\GINC-LOCALHOST\FOpt\UB3LYP\def2TZVP\C14H22B10O2(1-,2)\PIOTR\19-Oct
-2024\0\\#P UB3LYP/Def2TZVP FOpt geom(noangle, nodistance) fcheck #P S
CRF(Solvent=C6H5Cl)\\MeOPh-B10-PhOMe, rad anion C2 in PhCl\\-1,2\B,1.2
21060697,-0.7622670735,0.4514197345\B,-1.2279431909,-0.7511939007,-0.4
732396746\B,-0.5356361464,-0.7208885793,1.1843895453\B,0.5290140009,-0
.726019232,-1.205982323\B,1.2279431909,0.7511939007,-0.4732396746\B,0.
5356361464,0.7208885793,1.1843895453\B,-1.221060697,0.7622670735,0.451
4197345\B,-0.5290140009,0.726019232,-1.205982323\B,0.0087628375,1.8656
285607,-0.0108071237\B,-0.0087628375,-1.8656285607,-0.0108071237\C,1.2
169473015,5.53878431,0.158637559\C,-1.1750784588,5.553235574,-0.175348
5163\C,1.2048158843,4.1574491645,0.1529901933\C,-1.1623081835,4.163380
4302,-0.171097584\C,0.0228984782,6.251239455,-0.0073103283\C,0.0156912
356,3.4137929306,-0.0099079929\C,-0.0156912356,-3.4137929306,-0.009907
9929\C,-1.2048158843,-4.1574491645,0.1529901933\C,1.1623081835,-4.1633
804302,-0.171097584\C,-1.2169473015,-5.53878431,0.158637559\C,1.175078
4588,-5.553235574,-0.1753485163\C,-0.0228984782,-6.251239455,-0.007310
3283\H,2.2397615066,-1.2008531408,0.8903880926\H,-2.250663305,-1.18067
75378,-0.9118575016\H,-0.9481128541,-1.1565720661,2.2141902726\H,0.937
5893374,-1.1648085118,-2.2360282278\H,2.250663305,1.1806775378,-0.9118
575016\H,0.9481128541,1.1565720661,2.2141902726\H,-2.2397615066,1.2008
531408,0.8903880926\H,-0.9375893374,1.1648085118,-2.2360282278\H,2.138
8154398,6.0922341307,0.2883659254\H,-2.1105353121,6.0779843157,-0.3064
946416\H,2.1411702259,3.6273561978,0.2793252679\H,-2.1024117156,3.6400
242394,-0.2983612799\H,-0.7309656554,9.4256856479,-0.09995706\H,-1.524
9610496,8.1990826644,-1.1153473812\H,-1.7674893798,8.1903425588,0.6522
153306\H,-2.1411702259,-3.6273561978,0.2793252679\O,0.128921562,7.6074
256171,0.0092340647\C,-1.0508037466,8.3877216621,-0.1489323511\H,2.102
4117156,-3.6400242394,-0.2983612799\H,-2.1388154398,-6.0922341307,0.28
83659254\H,2.1105353121,-6.0779843157,-0.3064946416\O,-0.128921562,-7.
6074256171,0.0092340647\C,1.0508037466,-8.3877216621,-0.1489323511\H,0
.7309656554,-9.4256856479,-0.09995706\H,1.5249610496,-8.1990826644,-1.
1153473812\H,1.7674893798,-8.1903425588,0.6522153306\\Version=ES64L-G1
6RevC.01\State=2-A\HF=-946.2678043\S2=0.758069\S2-1=0.\S2A=0.750059\RM
SD=4.657e-09\RMSF=7.405e-06\Dipole=0.,0.,-0.1813603\Quadrupole=-3.1230
135,16.7565353,-13.6335218,-18.7517984,0.,0.\PG=C02 [X(C14H22B10O2)]\\
@

Fe complexes

9a′

1\1\GINC-LOCALHOST\FOpt\RB3LYP\def2SVP\C10H28B10Fe1N1P2(1-)\PIOTR\26-A
pr-2025\0\\#P B3LYP/Def2SVP FOpt=tight SCF(Direct, tight) SCRF(COSMO,
Solvent=CH2CL2) #P Geom=(NoDistance,NoAngle) fcheck freq(noRaman)\\H-B
10CN-Fe(PH3)2Cp\\-1,1\B,-4.9038511613,-0.01865336,1.3253868998\B,-4.92
32062686,0.3287022153,-1.2503308711\B,-4.9342097491,-1.1328093779,-0.1
361218716\B,-4.8946620273,1.4414021014,0.2108199223\B,-3.383610752,0.9
263512363,1.0658255232\B,-3.4118838836,-0.9084017358,0.817462037\B,-3.
42580884,-0.6607889694,-1.0188026308\B,-3.3972418056,1.1742028103,-0.7

726622167\B,-2.3173855713,0.1179074503,0.0127362588\B,-6.0340598077,0.
1684796264,0.0478943857\H,-5.318537021,-0.1669045759,2.4578859759\H,-5
.3558340774,0.4848483803,-2.3749889836\H,-5.3728877344,-2.2564140148,-
0.2842122874\H,-5.2917943397,2.5774824369,0.3669136101\H,-2.9411036289
,1.6094096569,1.9649044687\H,-2.9921737006,-1.8180550499,1.5013696574\
H,-3.0181577356,-1.3559276049,-1.925381925\H,-2.9669704123,2.071858114
,-1.4653147887\C,-0.7882878717,0.0862029775,-0.0004508716\N,0.37730867
79,0.0557742712,-0.0088934281\C,2.3801244461,2.1264520826,0.0892389502
\H,1.4853050789,2.7479204035,0.1083847558\C,3.0146406884,1.561740803,1
.2286817816\C,3.044385632,1.676767993,-1.0828314475\H,2.7340379156,1.7
231427868,2.2684517426\C,4.1113059315,0.7609318027,0.7537876526\H,2.79
0086333,1.9405994139,-2.108350657\C,4.1298043957,0.8306028784,-0.66204
36205\H,4.8139305536,0.2032383231,1.3728928331\H,4.8496595131,0.336928
807,-1.3147022893\Fe,2.2993733783,-0.0043227452,-0.0163063178\P,2.2591
846589,-1.446118179,-1.7340714657\H,2.4136956741,-0.9205666771,-3.0433
736482\H,3.2165589955,-2.4919460807,-1.8283332412\H,1.0869145122,-2.21
63008674,-1.9474546939\P,2.258587524,-1.6326880232,1.5245700091\H,3.23
34590014,-2.6664879188,1.5159025632\H,2.383193252,-1.2563754582,2.8874
284461\H,1.097275058,-2.4410493684,1.6290792625\C,-7.6408634739,0.1605
200343,0.0592014058\C,-8.3261893729,1.5170363955,0.2880182813\H,-8.000
9021281,-0.5434861917,0.8354392639\H,-8.015267307,-0.2588250764,-0.895
6698889\C,-9.8586468691,1.4620085205,0.2968307006\H,-7.9790252628,1.94
99475755,1.2452217552\H,-7.9990180631,2.2329473083,-0.489393354\C,-10.
526701515,2.8193784433,0.5273486226\H,-10.2106369095,1.0368650931,-0.6
618358954\H,-10.1901938207,0.7516137265,1.0772827522\H,-11.6266136519,
2.7400753509,0.5274229018\H,-10.2243579088,3.2542944302,1.4955613807\H
,-10.2452355895,3.5419517761,-0.2579306558\\Version=ES64L-G16RevC.01\S
tate=1-A\HF=-2647.1215174\RMSD=5.098e-09\RMSF=1.523e-06\Dipole=13.2297
042,-1.5684313,-0.1219608\Quadrupole=-18.0303593,7.55938,10.4709792,-1
4.8334701,-1.1792683,-0.1605223\PG=C01 [X(C10H28B10Fe1N1P2)]\\@

[*closo*-$B_{10}H_8$-1-Pyrazine-10-{Fe}]$^{2-}$

1\1\GINC-LOCALHOST\FOpt\RB3LYP\def2SVP\C10H23B10Fe1N3P2\PIOTR\14-Oct-2
023\0\\#P B3LYP/Def2SVP FOpt SCF(Direct, tight) SCRF(COSMO, Solvent=CH
2CL2) #P Geom=(NoDistance,NoAngle) fcheck freq(noRaman)\\Pyrazine-B10C
N-Fe(PH3)2Cp\\0,1\B,3.195061705,-0.7283421998,1.0899704326\B,3.2040677
872,0.6590063962,-1.1426217893\B,3.196833744,1.0884015104,0.6576382425
\B,3.2014714928,-1.1578375512,-0.7102353117\B,1.6931806883,-1.31306976
56,0.2727918467\B,1.6953743289,0.2686416207,1.2458330693\B,1.696294365
5,1.2440582794,-0.3369554954\B,1.7049058029,-0.337437419,-1.3103203866
\B,0.616916167,-0.03436754,-0.0365023674\B,4.2613250228,-0.0350014933,
-0.0222035943\H,3.6363170798,-1.3141905432,2.0517422589\H,3.6528277796
,1.2448168713,-2.1009099434\H,3.6421800556,2.0431390461,1.2518551785\H
,3.6509562294,-2.1126953601,-1.3009879226\H,1.2601969626,-2.4113894939
,0.5330758572\H,1.2635929653,0.5311051167,2.3442001369\H,1.2656441394,
2.3427450142,-0.6002481766\H,1.281902363,-0.5991956329,-2.4121033833\C
,-0.9161975409,-0.0309328256,-0.0377285365\N,-2.080412652,-0.027484028
9,-0.0348571566\C,-4.019358921,-1.8301810899,-1.1700478412\H,-3.109581
1573,-2.3127828333,-1.5261183196\C,-4.6300024345,-2.0535521439,0.09463
21212\C,-4.7343826582,-0.814582109,-1.8574738349\H,-4.3117062563,-2.77
47344716,0.8458570639\C,-5.7633561266,-1.1732661253,0.1817614031\H,-4.
5080955484,-0.427373776,-2.849866971\C,-5.82773622,-0.4119892865,-1.01
22621631\H,-6.4594999176,-1.1050715594,1.0177749975\H,-6.5821852272,0.
3373881467,-1.250549081\Fe,-3.9984104697,-0.0290910421,-0.0237308981\P
,-4.0352984724,2.1330430274,-0.6247270843\H,-4.2752891077,2.4324958671
,-1.9905256502\H,-4.979618617,3.0292591244,-0.0559841079\H,-2.86573844
47,2.9134140173,-0.4333074533\P,-3.9976295505,0.4428501411,2.169453905

8\H,-4.9949276977,1.2873580614,2.7259524602\H,-4.1196234637,-0.6384838
988,3.0800876761\H,-2.8542283566,1.0699716748,2.728223824\C,6.47932324
38,-1.1272168066,0.3778989142\C,6.4829479701,1.0574309935,-0.401027783
7\C,7.8718282995,-1.1062713314,0.3787767728\H,5.9030078062,-1.99909747
2,0.6855953856\C,7.8753747784,1.0373585809,-0.3865355437\H,5.909526903
2,1.9290073338,-0.7149120352\H,8.4218488952,-1.9958464526,0.6996578928
\H,8.4283681286,1.9272620668,-0.7013469583\N,5.7881945989,-0.035043968
6,-0.0152091805\N,8.572485537,-0.0342136699,0.00005553\\Version=ES64L-
G09RevD.01\State=1-A\HF=-2753.4082294\RMSD=3.742e-09\RMSF=6.661e-06\Di
pole=-3.9982269,1.293214,0.8147416\Quadrupole=53.0315733,-23.7799037,-
29.2516696,-11.2767639,-6.6138551,-4.5793285\PG=C01 [X(C10H23B10Fe1N3P
2)]\\@

[*closo*-$B_{10}H_9$-1,10-(CN{Fe})$_2$]$^{2-}$

1\1\GINC-LOCALHOST\FOpt\RB3LYP\def2SVP\C12H30B10Fe2N2P4\PIOTR\19-Dec-2
020\0\\#P B3LYP/Def2SVP FOpt SCRF(COSMO, Solvent=CH2CL2) #P Geom=(NoDi
stance,NoAngle) fcheck\\CpFe(PH3)2-CNB10CN-Fe(PH3)2Cp\\0,1\B,-0.745676
0109,0.2976010233,-1.2897271919\B,-0.7568324905,-0.2667449905,1.273669
5075\B,-0.7247551097,1.2965992175,0.2746050444\B,-0.7780187108,-1.2655
710609,-0.2906060927\B,0.7366367581,-0.721422945,-1.1152456861\B,0.774
2378064,1.08993474,-0.7145057953\B,0.7662607339,0.6892793783,1.0983718
448\B,0.7286305551,-1.1220596933,0.6979983121\B,1.8342348961,-0.038799
5743,-0.0086106304\B,-1.8342418134,0.0382033253,-0.0078127582\H,-1.169
6112969,0.5503179371,-2.3941863933\H,-1.190706862,-0.5018749335,2.3781
939934\H,-1.1297534425,2.4101135844,0.518561913\H,-1.2296548088,-2.361
1190229,-0.5345371551\H,1.151746598,-1.3393446474,-2.0687803018\H,1.22
18001413,2.0345167378,-1.3236738389\H,1.2073881597,1.2895308941,2.0516
108276\H,1.1367823695,-2.0842776303,1.3070464359\C,-3.3670441571,0.065
9519597,-0.0054716504\N,-4.5313010021,0.0855201586,-0.0020670478\C,3.3
671831413,-0.0667152576,-0.0068032341\N,4.5314345214,-0.0863640438,-0.
0037765251\C,-6.4283678548,2.2517827071,-0.0666344742\H,-5.5054039641,
2.8308849785,-0.0843614459\C,-7.0988875401,1.7360994177,-1.2086072561\
C,-7.1036753671,1.8159296606,1.10466508\H,-6.820681625,1.9007791782,-2
.2485208636\C,-8.2293930259,0.9823622631,-0.736018073\H,-6.82934805,2.
0514749853,2.1319121377\C,-8.2322745307,1.0303767102,0.6806959956\H,-8
.9637854381,0.4693323813,-1.3569065231\H,-8.9698998696,0.56207625,1.33
21883639\C,6.4299347652,-2.2516743843,-0.0704962696\H,5.5074271961,-2.
8314876695,-0.0885679167\C,7.0999026935,-1.7345542369,-1.2121428832\C,
7.105150307,-1.8163765053,1.1010596214\H,6.8216284475,-1.8985291388,-2
.2521493681\C,8.229970213,-0.9804462282,-0.7391015875\H,6.8311708804,-
2.0530074094,2.1281501834\C,8.2331390654,-1.0296860614,0.6775704074\H,
8.9638885762,-0.4663678711,-1.3596846598\H,8.9705578202,-0.5614472471,
1.3293403154\Fe,6.4503621606,-0.118777196,0.0044403863\Fe,-6.450229995
4,0.1188462278,0.0064588615\P,6.4879498531,1.353231723,1.698041421\H,6
.6264946962,0.8422222472,3.0145172336\H,7.4955662412,2.3521961038,1.76
43826016\H,5.3555182032,2.1827090571,1.9046389356\P,6.4959537854,1.486
0141234,-1.562660233\H,7.5127849986,2.4778623738,-1.5553732206\H,6.618
9344667,1.0840713713,-2.9179413881\H,5.3699995732,2.339772233,-1.69027
90799\P,-6.4888152165,-1.3546269535,1.6987775591\H,-6.6279896778,-0.84
47071451,3.0156060183\H,-7.4966844635,-2.3534362788,1.763701183\H,-5.3
567537849,-2.184609781,1.9053566553\P,-6.4971642728,-1.4844953708,-1.5
62126431\H,-7.5137014806,-2.4766530776,-1.5545629275\H,-6.6219196081,-
1.0812902661,-2.9168670816\H,-5.3711857738,-2.3378935284,-1.6919297055
\\Version=ES64L-G09RevD.01\State=1-A\HF=-4725.2142892\RMSD=4.404e-09\R
MSF=4.431e-06\Dipole=-0.003106,0.0012181,0.160448\Quadrupole=127.13886
84,-67.7196663,-59.4192021,37.4816205,-0.0121354,-0.0

[*closo*-$B_{10}H_8$-1-Bipyridyl-10-{Fe}]$^{2-}$

1\1\GINC-LOCALHOST\FOpt\RB3LYP\def2SVP\C16H27B10Fe1N3P2\PIOTR\16-Nov-2
023\0\\#P B3LYP/Def2SVP FOpt(ModRedundant) SCF(Direct) SCRF(COSMO, Sol
vent=CH2CL2) #P Geom=(NoDistance,NoAngle, Step=9) fcheck freq(noRaman)
\\Bipyr-B10CN-Fe(PH3)2Cp with frozen bipoyridyl two angles\\0,1\B,1.34
15639959,0.62478178,-1.1492048561\B,1.339485244,-0.6169696013,1.164240
8059\B,1.3418028317,-1.1555364662,-0.6067240916\B,1.3395207571,1.16469
72964,0.6224925329\B,-0.1629044804,1.2609744405,-0.3768209978\B,-0.160
1482441,-0.3791353009,-1.2496803346\B,-0.1627300655,-1.2538164022,0.38
89879009\B,-0.1627767417,0.3862079595,1.26227323\B,-1.2426313125,0.003
2810408,0.0053125517\B,2.4069355134,0.0054846357,0.0094304334\H,1.7866
232216,1.1522028903,-2.1433646798\H,1.7831520306,-1.1442142009,2.15904
84196\H,1.7871063302,-2.1468403824,-1.1390825951\H,1.7817262196,2.1567
272217,1.1559931407\H,-0.5954169569,2.3412960506,-0.7064235279\H,-0.58
65621124,-0.70893718,-2.3323612277\H,-0.5948517833,-2.3345575631,0.717
5683043\H,-0.5916380572,0.715625523,2.3439354057\C,-2.7754748433,0.002
4981021,0.0067294686\N,-3.9398713721,0.0013013527,0.0133445583\C,-5.83
12651614,0.171778669,2.1727966323\H,-4.9065789274,0.208846922,2.748130
6962\C,-6.4940170559,1.2941378618,1.6061840162\C,-6.5174766876,-1.0127
361553,1.7940679925\H,-6.206446247,2.3381412057,1.7214206141\C,-7.6308
425338,0.7962937866,0.8789157968\H,-6.2509518516,-2.029933228,2.077366
5714\C,-7.6449212533,-0.6165555729,0.9924175884\H,-8.3618431393,1.3987
918882,0.3397121946\H,-8.389523963,-1.28305112,0.5575593139\Fe,-5.8586
507349,0.0001345678,0.0450445554\P,-5.9111882544,-1.7611906362,-1.3445
035104\H,-6.0843112573,-3.0474677617,-0.7712565422\H,-6.9077680846,-1.
8560696237,-2.3522920744\H,-4.7739327216,-2.0332673436,-2.1481349791\P
,-5.9068493585,1.4905598821,-1.6290772092\H,-6.9140108476,1.4244015629
,-2.6287345313\H,-6.0515433299,2.8608574787,-1.2905787019\H,-4.7751934
831,1.5949104387,-2.4785887102\C,4.6260479268,1.1173990708,-0.32545965
01\C,4.6205416726,-1.1122638996,0.3597918349\C,6.0106640595,1.14853016
62,-0.3238571079\H,4.0303777023,1.9866112022,-0.6030245899\C,6.0049856
251,-1.144080623,0.3780151282\H,4.0205520284,-1.9809230801,0.629560259
1\H,6.5087548268,2.0709506883,-0.6234320077\H,6.4982039458,-2.06669684
04,0.6849562519\N,3.9361487682,0.0026600588,0.0119272619\C,6.744311028
4,0.0023402449,0.0334300823\C,8.227638128,0.0020662271,0.0440311945\C,
8.9583585943,1.1617613393,0.3544803482\C,8.9620127468,-1.1575676715,-0
.2579826141\C,10.3539988079,1.1074058124,0.348481031\H,8.4571326406,2.
0944077277,0.6198108351\C,10.3575022588,-1.1015034643,-0.2420150834\H,
8.4639876408,-2.0907950611,-0.5272463286\H,10.9318213604,2.0051380866,
0.5965118168\H,10.9381749183,-1.9986390383,-0.4855037961\N,11.05070403
78,0.0036260358,0.0547749795\\Version=ES64L-G09RevD.01\State=1-A\HF=-2
984.3144537\RMSD=6.783e-09\RMSF=6.370e-05\Dipole=-3.2591403,-0.138573,
-1.4364792\Quadrupole=71.8531979,-27.4108835,-44.4423144,1.6677061,18.
2325521,-1.1320539\PG=C01 [X(C16H27B10Fe1N3P2)]\\@

Pyridinium cations

[PyrCN]′

1\1\GINC-LOCALHOST\FOpt\RB3LYP\def2TZVP\C8H9N2(1+)\PIOTR\11-Oct-2020\0
\\#P B3LYP/Def2TZVP FOpt(tight) geom(noangle, nodistance) fcheck #P fr
eq(noraman, readIso) SCRF(Solvent=C6H5Cl) guess=check\\4-CN-Pyridinium
-N-Et, Cs\\1,1\C,0.0960834539,0.8507468963,-1.2049773922\C,0.096083453
8,0.8507468992,1.2049773903\C,0.247515048,-0.5172699195,-1.170804331\C
,0.2475150479,-0.5172699167,1.1708043323\C,0.0187632168,1.551170789,-0
.0000000018\N,0.3213943984,-1.1771034116,0.0000000014\H,0.0441004212,1
.3581119782,-2.1561900685\H,0.044100421,1.3581119833,2.1561900653\H,0.
3177558343,-1.1122847889,-2.0685624813\H,0.3177558341,-1.112284784,2.0

68562484\C,0.4492683588,-2.664425061,0.0000000032\C,-0.9099136323,-3.3
469320109,0.0000000039\H,1.030788573,-2.9263096698,-0.8807168633\H,1.0
307885729,-2.9263096677,0.8807168704\H,-0.7537813599,-4.425563739,0.00
00000052\H,-1.4870136755,-3.0856410238,0.8870623922\H,-1.4870136754,-3
.0856410259,-0.887062385\C,-0.1335099783,2.9735858366,-0.0000000035\N,
-0.2559472151,4.1174999807,-0.0000000049\\Version=ES64L-G09RevD.01\Sta
te=1-A'\HF=-419.7392617\RMSD=3.212e-09\RMSF=4.496e-06\Dipole=0.5061479
,-3.0657204,0.\Quadrupole=-3.9928044,-2.8759577,6.8687621,0.0204764,0.
,0.\PG=CS [SG(C4H1N2),X(C4H8)]\\@

[PyrCOOC$_1$]’

1\1\GINC-LOCALHOST\FOpt\RB3LYP\def2TZVP\C9H12N1O2(1+)\PIOTR\07-Oct-202
0\0\\#P B3LYP/Def2TZVP FOpt(tight, RCFC) geom(noangle, nodistance) fch
eck #P guess=check SCRF(Solvent=C6H5Cl)\\4-MeOCO-Pyridinium-N-Et, C1 o
pt at Def2SVP in PhCl\\1,1\C,-0.5476762605,1.2961932447,-0.0249535941\
C,-0.3028379356,-1.0891045616,0.0102691075\C,-1.9140128877,1.129953658
3,-0.0187649696\C,-1.6767325061,-1.199384705,0.0165085884\C,0.27742738
49,0.1758836483,-0.0092942076\N,-2.4572017445,-0.1027176333,0.00186693
26\H,-0.1243051534,2.2891665824,-0.0458011241\H,0.3005565078,-1.983252
1302,0.0171134537\H,-2.6010445308,1.9625915227,-0.0352883639\H,-2.1824
053813,-2.153125957,0.0290432721\C,-3.9403473075,-0.250968244,0.051271
7599\C,-4.4638159931,-0.221708106,1.4790742983\H,-4.3558033281,0.55828
83145,-0.5447951094\H,-4.1772539807,-1.1903592842,-0.4424818155\H,-4.2
338358779,0.7240944019,1.9700568042\H,-5.5473082027,-0.3368425324,1.45
31270524\H,-4.0482610631,-1.03673125,2.0714529488\C,1.768434794,0.3857
647142,-0.0174655307\O,2.2691924552,1.4824111361,-0.0392491193\O,2.429
8259304,-0.7644765192,0.0034341834\C,3.8748739207,-0.681095479,0.00038
99301\H,4.2155370917,-0.1421904174,0.882048905\H,4.2210836883,-1.70887
50113,0.0172703732\H,4.2143298003,-0.1719800925,-0.8992621856\\Version
=ES64L-G09RevD.01\State=1-A\HF=-555.4425592\RMSD=9.452e-09\RMSF=8.531e
-06\Dipole=-2.739243,-0.9965546,-0.0823055\Quadrupole=19.457926,-6.766
0533,-12.6918727,-5.6241835,-0.691688,0.1194001\PG=C01 [X(C9H12N1O2)]\
\@

Et-Pyr-Pyr-Et, [Q2]

1\1\GINC-LOCALHOST\FOpt\RB3LYP\def2TZVP\C14H18N2(2+)\PIOTR\07-Oct-2020
\0\\#P B3LYP/Def2TZVP FOpt(tight, RCFC) geom(noangle, nodistance) fche
ck #P guess=check SCRF(Solvent=C6H5Cl)\\Paraquat-N-Et, C2 opt at Def2S
TVP in PhCl\\2,1\C,-0.5012735636,1.4196290094,-1.0908761565\C,0.224894
4885,1.4904541016,1.1880091409\C,-0.6282446195,2.7899524371,-1.0625066
455\C,0.0843204389,2.8598398788,1.1607191164\C,-0.0690916446,0.7370026
507,0.0487123438\N,-0.3385740141,3.4893289696,0.0491261742\C,0.0690916
446,-0.7370026507,0.0487123438\C,-0.2248944885,-1.4904541016,1.1880091
409\C,0.5012735636,-1.4196290094,-1.0908761565\C,-0.0843204389,-2.8598
398788,1.1607191164\C,0.6282446195,-2.7899524371,-1.0625066455\N,0.338
5740141,-3.4893289696,0.0491261742\H,-0.7662112885,0.9001134813,-1.999
43874\H,0.5801489319,1.0302145274,2.0981514533\H,-0.9670310125,3.35686
49802,-1.9162869917\H,0.3005869012,3.4802137041,2.0174978354\H,-1.2424
90431,5.2224386493,-0.6674466511\H,-0.7606982714,5.2813370217,1.027373
0211\H,-0.5801489319,-1.0302145274,2.0981514533\H,0.7662112885,-0.9001
134813,-1.99943874\C,0.445976431,-4.9777757639,0.0315116873\C,-0.86814
2792,-5.6295606152,-0.3690853321\H,-0.3005869012,-3.4802137041,2.01749
78354\H,0.9670310125,-3.3568649802,-1.9162869917\H,-1.1808758206,-5.31
02004226,-1.363484372\H,-0.727828297,-6.7101338345,-0.3853652416\H,-1.
6613260087,-5.3999754013,0.342314187\C,-0.445976431,4.9777757639,0.031
5116873\C,0.868142792,5.6295606152,-0.3690853321\H,1.242490431,-5.2224
386493,-0.6674466511\H,0.7606982714,-5.2813370217,1.0273730211\H,1.180

8758206,5.3102004226,-1.363484372\H,0.727828297,6.7101338345,-0.385365
2416\H,1.6613260087,5.3999754013,0.342314187\\Version=ES64L-G09RevD.01
\State=1-A\HF=-653.7277058\RMSD=6.991e-09\RMSF=6.193e-06\Dipole=0.,0.,
0.1423679\Quadrupole=-38.6485088,61.0978994,-22.4493906,-5.6571167,0.,
0.\PG=C02 [X(C14H18N2)]\\@

Aryl and *closo*-borane halides

[*closo*-$B_{12}H_{11}$-1-I]$^{2-}$

1\1\GINC-LOCALHOST\FOpt\RB3LYP\def2TZVP\B12H11I1(2-)\PIOTR\08-Jun-2024
\0\\#P B3LYP/Def2TZVP FOpt(tight) geom(noangle, nodistance) fcheck fre
q\\1-I-B12, symmetry in vaccum\\-2,1\B,1.5165948121,-2.4100489319,0.\B
,0.4687846432,-2.4105124167,1.442109021\B,0.4687846432,-2.4105124167,-
1.442109021\B,-1.226524827,-2.4108232126,0.8913634126\B,-1.226524827,-
2.4108232126,-0.8913634126\B,-1.5235667981,-0.8977808031,0.\B,-0.47095
69899,-0.8975165717,1.4489050264\B,-0.4709569899,-0.8975165717,-1.4489
050264\B,1.2322399227,-0.8971614508,-0.8956048637\B,1.2322399227,-0.89
71614508,0.8956048637\B,-0.0003403455,-0.0046696013,0.\I,-0.0009388934
,2.2941860542,0.\B,0.0003805071,-3.3439849635,0.\H,2.5916389638,-2.941
2028281,0.\H,0.8011067096,-2.9418774276,2.4643908991\H,0.8011067096,-2
.9418774276,-2.4643908991\H,-2.0960027633,-2.9425558682,1.5231163443\H
,-2.0960027633,-2.9425558682,-1.5231163443\H,-2.5847559877,-0.34724723
79,0.\H,-0.7990191852,-0.3468821861,2.4580547703\H,-0.7990191852,-0.34
68821861,-2.4580547703\H,2.0904691563,-0.3460893834,-1.5192808339\H,2.
0904691563,-0.3460893834,1.5192808339\H,0.0007884016,-4.5432656393,0.\
\Version=ES64L-G16RevC.01\State=1-A'\HF=-603.0559334\RMSD=1.509e-09\RM
SF=5.931e-06\Dipole=-0.0009865,3.973696,0.\Quadrupole=9.5728447,-19.14
59869,9.5731422,0.0068738,0.,0.\PG=CS [SG(B4H3I1),X(B8H8)]\\@

[*closo*-$CB_{11}H_{11}$-12-I]$^{-}$

1\1\GINC-LOCALHOST\FOpt\RB3LYP\def2TZVP\C1H11B11I1(1-)\PIOTR\09-Jun-20
24\0\\#P B3LYP/Def2TZVP FOpt(tight) geom(noangle, nodistance) fcheck f
req\\12-I-CB11, symmetry in vaccum\\-1,1\B,0.0000000003,1.5104822148,-
2.3671880733\B,-1.4365539531,0.4667646743,-2.3671880733\B,1.4365539533
,0.4667646737,-2.3671880733\B,-0.88783917,-1.2220057814,-2.3671880733\
B,0.8878391695,-1.2220057818,-2.3671880733\B,-0.0000000003,-1.52305723
24,-0.8671468687\B,-1.4485135056,-0.470650568,-0.8671468687\B,1.448513
5054,-0.4706505686,-0.8671468687\B,0.8952305798,1.2321791841,-0.867146
8687\B,-0.8952305793,1.2321791845,-0.8671468687\B,0.,-0.0000000001,0.0
423189621\I,0.,-0.0000000001,2.2772522045\C,0.,-0.0000000001,-3.151336
8311\H,0.0000000005,2.5033172489,-3.0161912568\H,-2.3807961819,0.77356
75727,-3.0161912568\H,2.3807961822,0.7735675717,-3.0161912568\H,-1.471
4129612,-2.0252261965,-3.0161912568\H,1.4714129604,-2.0252261972,-3.01
61912568\H,-0.0000000005,-2.5907126896,-0.3491494199\H,-2.4639141854,-
0.8005742482,-0.3491494199\H,2.4639141851,-0.8005742492,-0.3491494199\
H,1.5227827123,2.095930593,-0.3491494199\H,-1.5227827114,2.0959305936,
-0.3491494199\H,0.,-0.0000000001,-4.2305009869\\Version=ES64L-G16RevC.
01\State=1-A1\HF=-616.3339767\RMSD=2.376e-09\RMSF=1.645e-06\Dipole=0.,
0.,0.4496043\Quadrupole=1.2245074,1.2245074,-2.4490148,0.,0.,0.\PG=C05
V [C5(H1C1B1I1),5SGV(H2B2)]\\@

[*closo*-$CB_{9}H_{9}$-10-I]$^{-}$

1\1\GINC-LOCALHOST\FOpt\RB3LYP\def2TZVP\C1H9B9I1(1-)\PIOTR\08-Jun-2024
\0\\#P B3LYP/Def2TZVP FOpt(tight) geom(noangle, nodistance) fcheck fre
q\\1-CB9-10-I, C4v in vaccum\\-1,1\B,-1.3078070328,0.,-0.0819475673\B,
0.,1.3078070328,-0.0819475673\B,1.3078070328,0.,-0.0819475673\B,0.,-1.
3078070328,-0.0819475673\B,-0.921755725,-0.921755725,1.4200305877\B,-0
.921755725,0.921755725,1.4200305877\B,0.921755725,0.921755725,1.420030

5877\B,0.921755725,-0.921755725,1.4200305877\C,0.,0.,2.3465005275\B,0.
,0.,-1.1477587282\I,0.,0.,-3.3460451015\H,0.,0.,3.4229687732\H,-2.4248
902374,0.,-0.4914259845\H,0.,2.4248902374,-0.4914259845\H,2.4248902374
,0.,-0.4914259845\H,0.,-2.4248902374,-0.4914259845\H,-1.6820218945,-1.
6820218945,1.9250670116\H,-1.6820218945,1.6820218945,1.9250670116\H,1.
6820218945,1.6820218945,1.9250670116\H,1.6820218945,-1.6820218945,1.92
50670116\\Version=ES64L-G16RevC.01\State=1-A1\HF=-565.3305741\RMSD=7.8
80e-09\RMSF=2.555e-06\Dipole=0.,0.,-0.8069593\Quadrupole=2.3389747,2.3
389747,-4.6779493,0.,0.,0.\PG=C04V [C4(H1C1B1I1),2SGV(H2B2),2SGD(H2B2)
]\\@

$C_2B_{10}H_{11}$-9-I

1\1\GINC-LOCALHOST\FOpt\RB3LYP\def2TZVP\C2H11B10I1\PIOTR\08-Jun-2024\0
\\#P B3LYP/Def2TZVP FOpt(tight) geom(noangle, nodistance) fcheck freq\
\9-I-C2B10, Cs symmetry in vaccum\\0,1\C,-2.2550635271,-1.3757600807,0
.\B,-2.3438241059,-0.467184328,1.4527591193\B,-2.3438241059,-0.4671843
28,-1.4527591193\B,-2.3844761924,1.2148005243,0.8901668221\B,-2.384476
1924,1.2148005243,-0.8901668221\B,-0.8832959591,1.5349969967,0.\B,-0.8
656493203,0.4874871177,1.4526413433\B,-0.8656493203,0.4874871177,-1.45
26413433\B,-0.828716433,-1.2013114351,-0.8920609039\B,-0.828716433,-1.
2013114351,0.8920609039\B,0.0770424058,0.038108526,0.\I,2.265779263,0.
0286095526,0.\C,-3.1352700515,-0.0135263198,0.\H,-2.8074480575,-2.3019
168983,0.\H,-3.0013219421,-0.8940321313,2.3311526496\H,-3.0013219421,-
0.8940321313,-2.3311526496\H,-3.0665368791,1.9771181178,1.4770937957\H
,-3.0665368791,1.9771181178,-1.4770937957\H,-0.3914273945,2.6083811915
,0.\H,-0.3632920797,0.8052894387,2.4722600933\H,-0.3632920797,0.805289
4387,-2.4722600933\H,-0.4105767758,-2.1361166874,-1.4751394446\H,-0.41
05767758,-2.1361166874,1.4751394446\H,-4.2061739964,-0.138233838,0.\\V
ersion=ES64L-G16RevC.01\State=1-A'\HF=-629.4281908\RMSD=3.691e-09\RMSF
=2.449e-06\Dipole=-2.1995707,-0.8794198,0.\Quadrupole=6.8961133,-1.913
028,-4.9830853,4.6079796,0.,0.\PG=CS [SG(C2H3B2I1),X(H8B8)]\\@

PhCl

1\1\GINC-LOCALHOST\Freq\UB3LYP\def2TZVP\Cl1(2)\PIOTR\08-Jun-2024\0\\#P
Geom=AllCheck Guess=TCheck SCRF=Check GenChk UB3LYP/def2TZVP Freq\\Cl
atom, in vacuum\\0,2\Cl,0.,1.3068100629,0.0537536324\\Version=ES64L-G
16RevC.01\HF=-460.1669788\S2=0.753113\S2-1=0.\S2A=0.750003\RMSD=8.004e
-10\RMSF=0.000e+00\Thermal=0.0014163\ETot=-460.1655625\HTot=-460.16461
83\GTot=-460.182656\Dipole=0.,0.,0.\DipoleDeriv=0.,0.,0.,0.,0.,0.,0.,0
.,0.\Polar=8.5825105,0.,8.636855,0.,0.,8.636855\Quadrupole=1.0547241,-
0.5273621,-0.5273621,0.,0.,0.\PG=OH [O(Cl1)]\NImag=0\\0.,0.,0.,0.,0.,0
.\\0.,0.,0.\\\@

PhI

1\1\GINC-LOCALHOST\FOpt\RB3LYP\def2TZVP\C6H5I1\PIOTR\08-Jun-2024\0\\#P
B3LYP/Def2TZVP FOpt(tight) geom(noangle, nodistance) fcheck freq\\PhI
, C2v in vaccum\\0,1\C,0.,1.2020822689,-2.6270220264\C,0.,-1.202082268
9,-2.6270220264\C,0.,1.2095721683,-1.2357062767\C,0.,-1.2095721683,-1.
2357062767\C,0.,0.,-3.3248553164\C,0.,0.,-0.5512416585\I,0.,0.,1.56364
33944\H,0.,2.1432709683,-3.1624224882\H,0.,-2.1432709683,-3.1624224882
\H,0.,2.1465184867,-0.6964037529\H,0.,-2.1465184867,-0.6964037529\H,0.
,0.,-4.4071990509\\Version=ES64L-G16RevC.01\State=1-A1\HF=-529.534161\
RMSD=3.697e-09\RMSF=4.790e-07\Dipole=0.,0.,-0.6626065\Quadrupole=-4.78
17146,1.6618943,3.1198202,0.,0.,0.\PG=C02V [C2(H1C1C1I1),SGV(C4H4)]\\@

9. References

1. M. F. Hawthorne, R. L. Pilling and W. H. Knoth, *Inorg. Synth.*, 1967, **9**, 16–19.
2. E. Rzeszotarska, I. Novozhilova and P. Kaszyński, *Inorg. Chem.*, 2017, **56**, 14351–14356.
3. G. R. Fulmer, A. J. M. Miller, N. H. Sherden, H. E. Gottlieb, A. Nudelman, B. M. Stoltz, J. E. Bercaw and K. I. Goldberg, *Organometallics*, 2010, **29**, 2176–2179.
4. R. Jakubowski, M. B. Abdulmojeed, O. Hietsoi, A. C. Friedli and P. Kaszynski, *Inorg. Chem.*, 2024, **63**, 17774−17784.
5. J. B. G. Gluyas, N. J. Brown, J. D. Farmer and P. J. Low, *Aust. J. Chem.*, 2017, **70**, 113-119.
6. L. Jacob, E. Rzeszotarska, M. Koyioni, R. Jakubowski, D. Pociecha, A. Pietrzak and P. Kaszyński, *Chem. Mater.*, 2022, **34**, 6476–6491.
7. CrysAlisPro, Rigaku Oxford Diffraction, 2020 and 2022, Yarnton, Oxfordshire, England.
8. G. M. Sheldrick, *Acta Cryst., Sect. A*, 2015, **A71**, 3-8.
9. G. M. Sheldrick, *Acta Cryst., Sect. C*, 2015, **C71**, 3-8.
10. O. V. Dolomanov, L. J. Bourhis, R. J. Gildea, J. A. K. Howard and H. Puschmann, *J. Appl. Cryst.*, 2009, **42**, 339-341.
11. S. Mebs, R. Kalinowski, S. Grabowsky, D. Förster, R. Kickbusch, E. Justus, W. Morgenroth, C. Paulmann, P. Luger, D. Gabel and D. Lentz, *Inorg. Chem.*, 2011, **50**, 90-103.
12. a) L. Jacob, E. Rzeszotarska, A. Pietrzak, V. G. Young, Jr. and P. Kaszyński, *Eur. J. Inorg. Chem.*, 2020, DOI: 10.1002/ejic.202000456, 3083–3093; b) S. Kapuściński, M. B. Abdulmojeed, T. E. Schafer, A. Pietrzak, O. Hietsoi, A. C. Friedli and P. Kaszyński, *Inorg. Chem. Front.*, 2021, **8**, 1066–1082.
13. C. Hansch, A. Leo and R. W. Taft, *Chem. Rev.*, 1991, **91**, 165–195.
14. T. Whelan, P. Brint, T. R. Spalding, W. S. McDonald and D. R. Lloyd, *J. Chem. Soc. Dalton Trans.*, 1982, 2469-2473.
15. S. Kapuściński, O. Hietsoi, A. Pietrzak, A. C. Friedli and P. Kaszyński, *Chem. Commun.*, 2022, **58**, 851–854.
16. H. D. Hall, B. D. Ulrich, R. G. Kultyshev, J. Liu, S. Liu, E. A. Meyers, S. Gréau and S. G. Shore, *Collect. Czech. Chem. Commun.*, 2002, **67**, 1007-1024.
17. V. V. Voinova, I. N. Klyukin, A. S. Novikov, A. Y. Kozmenkova, A. P. Zhdanov, K. Y. Zhizhin and N. T. Kuznetsov, *Russ. J. Inorg. Chem.*, 2021, **66**, 295–304.
18. a) A. D. Becke, *J. Chem. Phys.*, 1993, **98**, 5648-5652; b) C. Lee, W. Yang and R. G. Parr, *Phys. Rev. B,*, 1988, **37**, 785-789.
19. a) F. Weigend, *Phys. Chem. Chem. Phys.*, 2006, **8**, 1057-1065; b) F. Weigend and R. Ahlrichs, *Phys. Chem. Chem. Phys.*, 2005, **7**, 3297-3305.
20. M. J. Frisch, G. W. Trucks, H. B. Schlegel, G. E. Scuseria, M. A. Robb, J. R. Cheeseman, G. Scalmani, V. Barone, G. A. Petersson, H. Nakatsuji, X. Li, M. Caricato, A. V. Marenich, J. Bloino, B. G. Janesko, R. Gomperts, B. Mennucci, H. P. Hratchian, J. V. Ortiz, A. F. Izmaylov, J. L. Sonnenberg, Williams, F. Ding, F. Lipparini, F. Egidi, J. Goings, B. Peng, A. Petrone, T. Henderson, D. Ranasinghe, V. G. Zakrzewski, J. Gao, N. Rega, G. Zheng, W. Liang, M. Hada, M. Ehara, K. Toyota, R. Fukuda, J. Hasegawa, M. Ishida, T. Nakajima, Y. Honda, O. Kitao, H. Nakai, T. Vreven, K. Throssell, J. A. Montgomery Jr., J. E. Peralta, F. Ogliaro, M. J. Bearpark, J. J. Heyd, E. N. Brothers, K. N. Kudin, V. N. Staroverov, T. A. Keith, R. Kobayashi, J. Normand, K. Raghavachari, A. P. Rendell, J. C. Burant, S. S. Iyengar, J. Tomasi, M. Cossi, J. M. Millam, M. Klene, C.

Adamo, R. Cammi, J. W. Ochterski, R. L. Martin, K. Morokuma, O. Farkas, J. B. Foresman and D. J. Fox, *Journal*, 2016.

21. M. Cossi, G. Scalmani, N. Rega and V. Barone, *J. Chem. Phys.*, 2002, **117**, 43–54.
22. D. F. McMillen and D. M. Golden, *Ann. Rev. Phys. Chem.* , 1982, **33**, 493–532.
23. S. J. Blanksby and G. B. Ellison, *Acc. Chem. Res.*, 2003, **36**, 255-263, and references therein.
24. C. Galli and T. Pau, *Tetrahedron*, 1998, **54**, 2893-2904.
25. A. Klamt and G. Schüürmann, *J. Chem. Soc. Perkin Trans. 2*, 1993, DOI: 10.1039/P29930000799, 799–805.
26. J. Guschlbauer, K. H. Shaughnessy, A. Pietrzak, M.-C. Chung, M. B. Sponsler and P. Kaszyński, *Organometallics*, 2021, **40**, 2504−2515.
